# Pd-Catalyzed De Novo Skeletal Editing of Bicyclic Carbamates Delivers 2‑Pyrimidinones

**DOI:** 10.1021/acs.orglett.6c02359

**Published:** 2026-07-06

**Authors:** Wangyu Shi, Yue Ren, Jordi Benet-Buchholz, Maria Vicent Morales, Arjan W. Kleij

**Affiliations:** † 202569 Institute of Chemical Research of Catalonia (ICIQ-Cerca), the Barcelona Institute of Science and Technology (BIST), Tarragona 43007, Spain; ‡ Departament de Química Física i Inorgànica, Universitat Rovira i Virgili, Tarragona 43007, Spain; § Catalan Institute of Research and Advanced Studies (ICREA), Barcelona,08010, Spain

## Abstract

A newly designed Pd-catalyzed decarboxylative coupling between bicyclic carbamates and isocyanates allows to produce a wide range of 2-pyrimidinone mimics in good yields under user-friendly conditions and applying simple catalyst components. This catalytic transformation represents a formal and *de novo* skeletal editing that creates a novel and practical approach for potential 3D bioisosteric, nitrogen-based functionalized heterocycles. The utilization of these scaffolds is demonstrated in postsynthetic diversifications and through the design and construction of 3D drug-like analogues.

## Graphical abstract

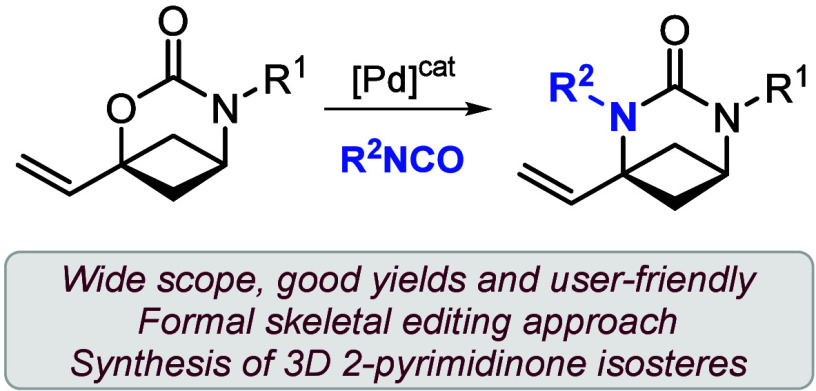

Nitrogen-containing heterocycles are fundamental structural components in drug discovery.^1^ Among the 321 novel small-molecule drugs approved by the FDA between 2013 and 2023, 82% contain at least one nitrogen-containing heterocycle (Scheme 1a).^2^ In this regard, the pyrimidine scaffold ranks as the fifth most frequently occurring heterocycle and should thus be considered as a privileged scaffold in small-molecule drug design,^3^ offering key advantages such as favorable target engagement,^4^ good biocompatibility,^5^ and ease of functionalization.^6^ Consequently, pyrimidine-based compounds have been successfully employed across nearly all major therapeutic areas, giving rise to development and commercialization of numerous blockbuster drugs.^1,7^ Therefore, the development of novel therapeutics toward the incorporation of a pyrimidine subunit is a major focus in medicinal chemistry.

**Scheme 1:**
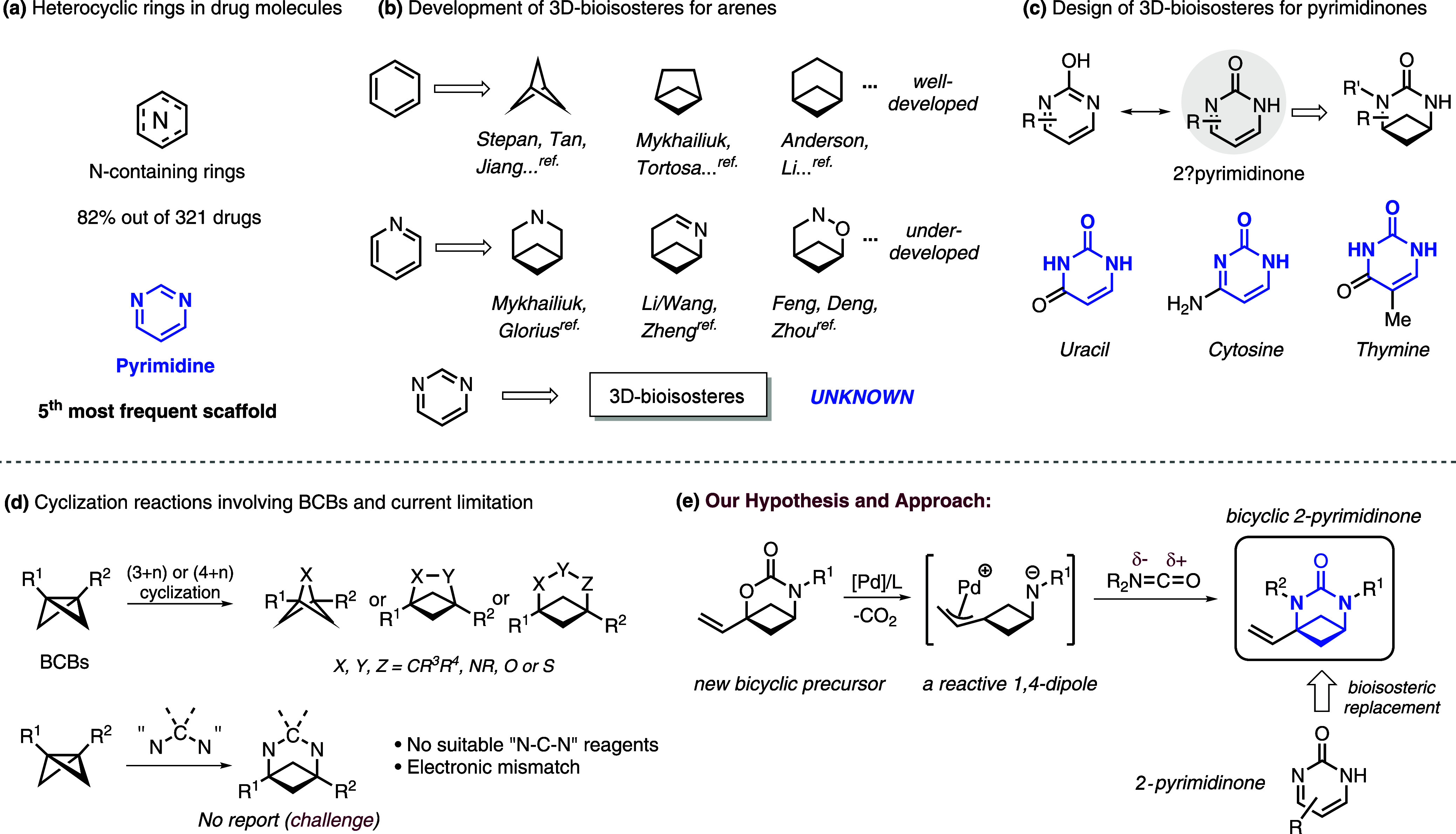
(a) Importance of Pyrimidine Scaffolds for Drug Development, (b) State of the Art in the Development of 3D Bioisosteres, (c) Designing Pyrimidinone Analogues, (d) Use of BCB Chemistry to Access Bioisosteric Small Molecules, and (e) Our Approach Offering New Pyrimidinone Analogues Using a Newly Designed Substrate

The ″Escape from Flatland″ concept emphasizes to increase the proportion of sp^3^-hybridized carbon atoms in (drug) molecules to improve their aqueous solubility, membrane permeability, and metabolic stability.^8^ Based on this concept, in recent years the use of sp^3^-rich, three-dimensional bioisosteres such as bicyclo­[*n*.1.1]­alkanes (*n* = 1, 2, 3) as replacements for planar aromatic rings has become a mainstream strategy for improving the physicochemical properties and pharmacological performance of bioactive molecules. Significant success has been achieved in the advancement of benzene- and pyridine-isosteres as illustrated by the work from Stepan,^9^ Tan,^10^ Jiang,^11^ Mykhailiuk,^12,13^ Tortosa,^14^ Anderson,^15^ Li,^16^ Glorius,^17^ Li and Wang,^18^ Zheng,^19^ Feng,^20^ Deng,^21^ Zhou,^22^ and others.^23,24^ Despite these remarkable advances, little progress on the exploration of potential structural 3D-bioisosteres for pyrimidines (Scheme 1b) has been reported.^25,26^ 2-Pyrimidinones represent highly important and relevant pyrimidine derivatives and actually constitute one of the most common forms of the pyrimidine scaffold in nature (Scheme 1c). Drugs based on this structure tend to exhibit superior biocompatibility.^5^ Therefore, developing 3D-bioisosteres reminiscent of pyrimidines is of great importance providing new directions and options for the optimization of new types of pyrimidine-containing drugs.

Bicyclic butanes (BCBs) are a highly versatile class of synthons employed for the construction of bicyclo­[*n*.1.1] scaffolds using photocatalysis, radical relay processes, boronyl radical catalysis, palladium catalysis, Lewis/Brønsted acid catalysis, and Lewis base catalysis (Scheme 1d).^27–33^ Despite the versatility of BCBs, 2,4-dinitrogen-containing bicyclo[3.1.1]­heptanes cannot be easily accessed from them (Scheme 1d). Given the limited chemical space of such diaza-bicyclics, we present a novel approach that does not depend on BCB precursors, while using newly designed bicyclic carbamate precursors (Scheme 1e). We anticipated these to undergo facile decarboxylation under Pd-catalysis,^34–36^ providing π-allyl Pd intermediates that should have the potential to undergo cycloaddition with readily available isocyanate reagents delivering pyrimidinones through a (formal) skeletal editing approach.^37–39^ Together with the synthesis of the 3D diaza-bicycles, we also demonstrate downstream transformations providing access to a wider variety of synthons. The potential of these novel pyrimidinone derivatives is also illustrated by the creation of various drug analogues.

Key to our investigation was the access to a precursor that would enable an easy entry to 2,4-diazabicyclo[3.1.1]­heptan-3-ones (2-pyrimidinone analogues), and commercially available *tert*-butyl (3-oxocyclobutyl)­carbamate **A** (a Boc-protected aminocyclobutanone) provided such a starting point allowing to prepare the initial target in just two steps (see SI for details). Treatment of **A** with vinyl magnesium bromide (giving intermediate **B**) followed by NaH-assisted annulation afforded the free NH-version of a bicyclic carbamate in typical yields around 60% in repeated experiments. N-protection (≥85% yield, repeated experiments) gave access to vinyl N-tosyl bicyclic carbamate **1a**. Next, **1a** was subjected to 4-chloro-arylisocyanate **2a** under Pd catalysis and varying conditions optimizing the solvent, Pd precursor and ancillary ligand (Figure 1, Table S1). Based on previous experience,^34–36^ we surmised that a Pd­(allyl) intermediate would easily form that engages with the isocyanate through the basic *N*-atom of the pi-allyl fragment (see the scheme to Table S1). Attack on the electrophilic carbon center of the isocyanate and subsequent ring-closure should then deliver the target 2,4-diazabicyclo[3.1.1]­heptan-3-one **3a**.

**Figure 1:**
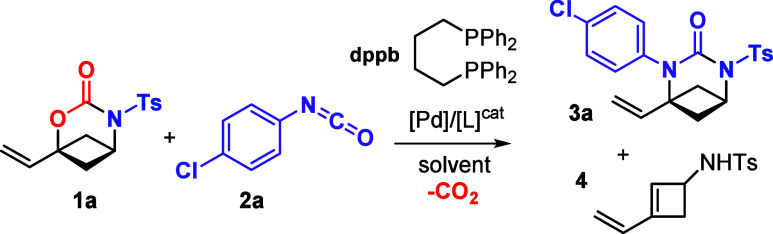
Optimized process conditions for the formation of 2,4-diazabicyclo[3.1.1]­heptan-3-one **3a**. Optimized conditions: Pd_2_(dba)_3_·CHCl_3_ (2.5 mol%), dppb (5 mol%), 1.0 mL CH_2_Cl_2_, N_2_ atmosphere, 15 min. Conv. (**1a**) >99%, NMR yield of **3a** is 90% (88% isolated).

Initially, we screened a few *N*- and *P*-based bidentate ligands (Table S1, entries 1–3) and found that these reactions provided access to product **3a** in different amounts (10–67%) and varying chemo-selectivities. A disubstituted cyclobutene derivative **4** ^40^ was identified as the major byproduct in these experiments, whose formation can be rationalized by an undesired β-hydride elimination reaction occurring from the Pd­(allyl) intermediate favored by the conjugated nature of the 1,3-diene in **4**.^41^ The use of simple and cheap triphenylphosphine (Table S1, entry 4) was also productive leading to product **3a** in 51% yield with a relatively low amount of byproduct **4** (6%). To further increase the chemo-selectivity toward **3a** and improving its yield, various less and more flexible bidentate diphosphines were examined (Table S1, entries 5–8), which led to the identification of dppb [1,4-bis­(diphenylphosphino)­butane] as a ligand favoring the rapid formation **3a** in high yield (Table S1, entry 5:15 min reaction time, conversion of **1a** > 99%, yield of **3a** 90% determined by ^1^H NMR, 88% isolated) with only trace amounts of byproduct **4** being observed. At this stage, we believe that the relative large bite angle in the Pd-dppb complex results in less stable and thus more reactive Pd intermediates propelling the formation of **3a**.

The presence of a ligand is crucial, as without it (Table S1, entry 9) even after 24 h (1440 min) hardly any conversion of **1a** was noted. In the absence of the isocyanate **2a**, conversion of **1a** takes place (entry 10, > 99%) but under these conditions the major identified product was **4** (26%). The presence of [Pd] is also required as virtually no conversion without it (Table S1, entry 11) occurs. Different solvents were scrutinized (Table S1, entries 12–15, vs entry 5) showing rather similar results when using DCM, DCE and THF and a somewhat inferior product yield in the case of ACN. The final experiments (Table S1, entries 16–18) were dedicated to revealing the influence of the reaction stoichiometry and the amount/type of Pd precursor. When inverting the ratio between **1a** and **2a** (from 1:1.2 to 1.2:1, Table S1, entry 16 vs entry 5), product **3a** was formed with a very similar yield of 88%. Lowering the amount of [Pd] and dppb to 1.0 and 2.0 mol %, respectively, effectively produced **3a** in high selectivity but with a reduced yield (Table S1, entry 17, 71%). Finally, replacing Pd_2_dba_3_·CHCl_3_ by Pd­(PPh_3_)_4_ as the metal precursor was not beneficial as it did not lead to any observable amounts of **3a**. While product **3a** can be easily assigned on the basis of specific ^1^H/^13^C NMR and IR spectroscopic signatures, an X-ray diffraction study of a single crystal obtained for **3a** (see insert in the scheme to Table S1; SI for details) unambiguously confirmed the proposed atom connectivity.

The conditions reported in entry 5 of Table S1 were then utilized to investigate the scope of this catalytic and formal skeletal editing process (Scheme 2), and we first varied the isocyanate reagent **2**. With product **3a** isolated in good yield (88%), we further varied the aryl-substituent R^2^ in the reagent **2**, allowing to introduce potentially useful functionalities including aryl halide (Br, I; synthesis of **3c** and **3d**), nitrile (**3g**), ester (**3h**), and acyl (**3i**) groups. Notably, within the series of products **3a**–**3l**, good-to-excellent yields of 61–97% were achieved with R^2^ having an electron-withdrawing character, while isocyanates with a (more) donating R^2^ ability gave rise to 2,4-diazabicyclo[3.1.1]­heptan-3-one products in much lower yields (**3j**–**3l**; 39–55%; note that in these cases, **4** was observed as a major side-product). This can be rationalized by assuming a lower electrophilic nature of the carbon atom of the isocyanate group, thereby attenuating the reactivity of it in the presence of the Pd­(allyl) species.

**Scheme 2:**
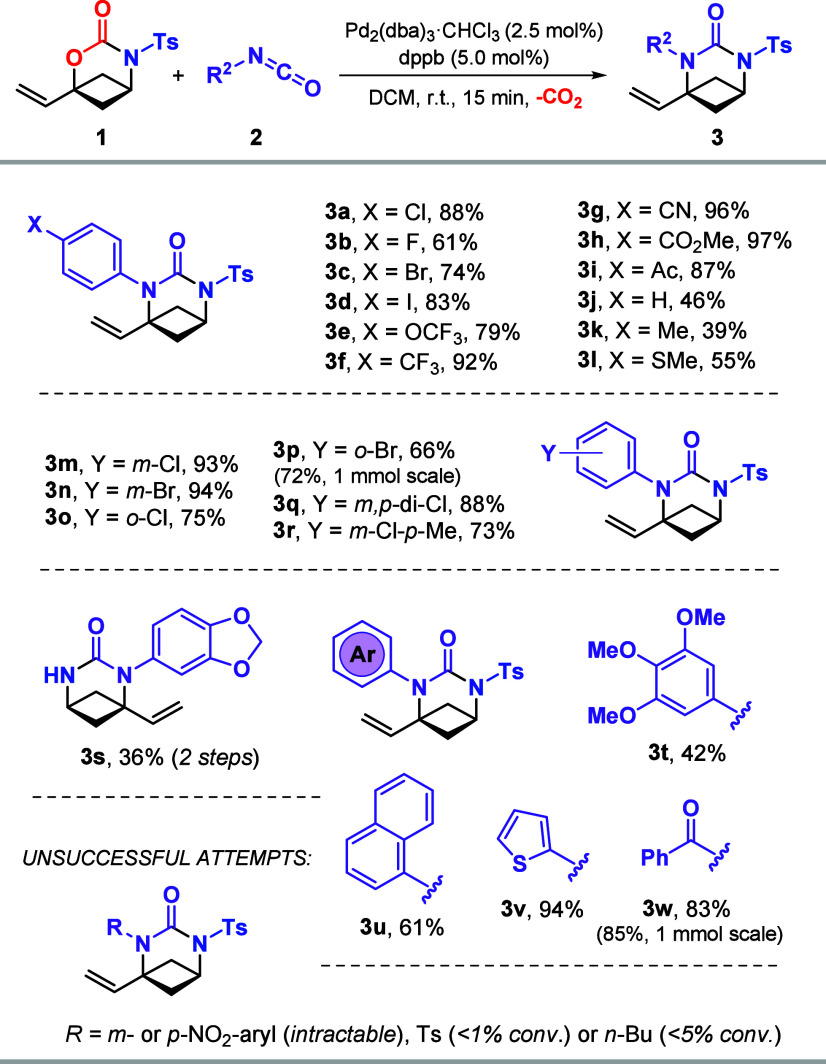
Product Scope for **3** Varying the Isocyanate Reagent **2**

Positional variation of the aryl-substituents (**3m**–**3r**; *ortho*, *meta* and *meta*,*para*) was feasible and the use of these isocyanate reagents **2** resulted in the formation of the desired 2,4-diazabicyclo[3.1.1]­heptan-3-one products in high yields (73–94%). Product **3p** could be easily scaled up 10 times with a slightly improved yield (72 vs 66%). Products with more complex aryl groups having multiple electron-donating substituents could also be prepared (**3s**: 36%, and **3t**: 42%) but in modest yield as noted for **3j**–**3l**. In the case of **3s**, the NTs product could not be purified well-enough by column chromatography and, therefore, the initial 2,4-diazabicyclo[3.1.1]­heptan-3-one was deprotected by magnesium powder in MeOH at 60 °C. The yield presented is thus of the two-step sequence without isolating the NTs intermediate. Larger aromatic (**3u**, 61%), heteroaromatic (**3v**, 94%) and an aryl acyl (**3w**, 83%; 85% when scaled up) in **2** were successfully probed providing the products in good to excellent yield. The use of aryl isocyanate reagents with a nitro-aryl, tosyl or alkyl group proved to be unproductive.

Second, we varied the substituent on the *N*-atom of the bicyclic carbamate precursor and combined it with two different isocyanates having either a *p*-chloro-aryl or a benzoyl substituent (Scheme 3). Compared to **3a** (88%), 2,4-diazabicyclo[3.1.1]­heptan-3-one **5a** (63%) and **5b** (40%) feature other aryl sulfonyl groups. In the case of **5c**, the lower yield is a result of a lower reactivity of the starting bicyclic carbamate (**5b**) as much lower conversion was noted for it even after 24 h. Larger aryl sulfonyl groups as those present in **5c** (2-naphthyl sulfonyl, 55%) and alkyl sulfonyls (**5d**–**f**; 75–92%) were also endorsed, with the synthesis of compound **5f** successfully scaled up ten times with a slightly improved yield of 80%. More complex heteroaryl sulfonyl fragments (products **5g**–**i**) are also tolerated in this protocol allowing to isolate the corresponding 2,4-diazabicyclo[3.1.1]­heptan-3-ones in 60–70% yield. Lastly, when we subjected bicyclic carbamates with *N*-Bz (Bz = benzoyl) or *N*-PMB (*p*-methoxy-benzyl) fragments to the optimized conditions, (hardly) no substrate conversion was observed indicating a privileged nature of *N*-aryl sulfonyl groups in the bicyclic carbamate substrate.

**Scheme 3:**
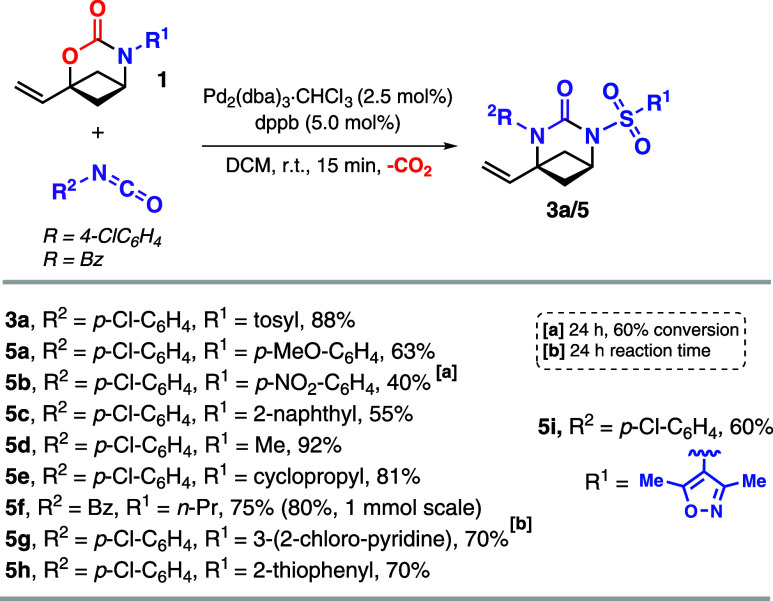
Product Scope for **3** while Varying the N-Substituent of the Bicyclic Carbamate Precursor **1**

The synthetic utility of various 2,4-diazabicyclo[3.1.1]­heptan-3-one was then examined (Scheme 4). Compound **3p** undergoes a deaminative borylation under Cu-catalysis in the presence of B_2_pin_2_ affording cyclobutane **6** in 58% yield (Scheme 4a). When **3a** is treated with Mg powder at 60 °C in MeOH, clean *N*-deprotection takes place delivering the free NH-based 2,4-diazabicyclo[3.1.1]­heptan-3-one **7** in 84% (Scheme 4b). Combining both *N*-deprotection and *N*-alkylation using BnBr (Scheme 4c) in a one-pot sequence provides access to **8** in an 80% overall yield.

**Scheme 4:**
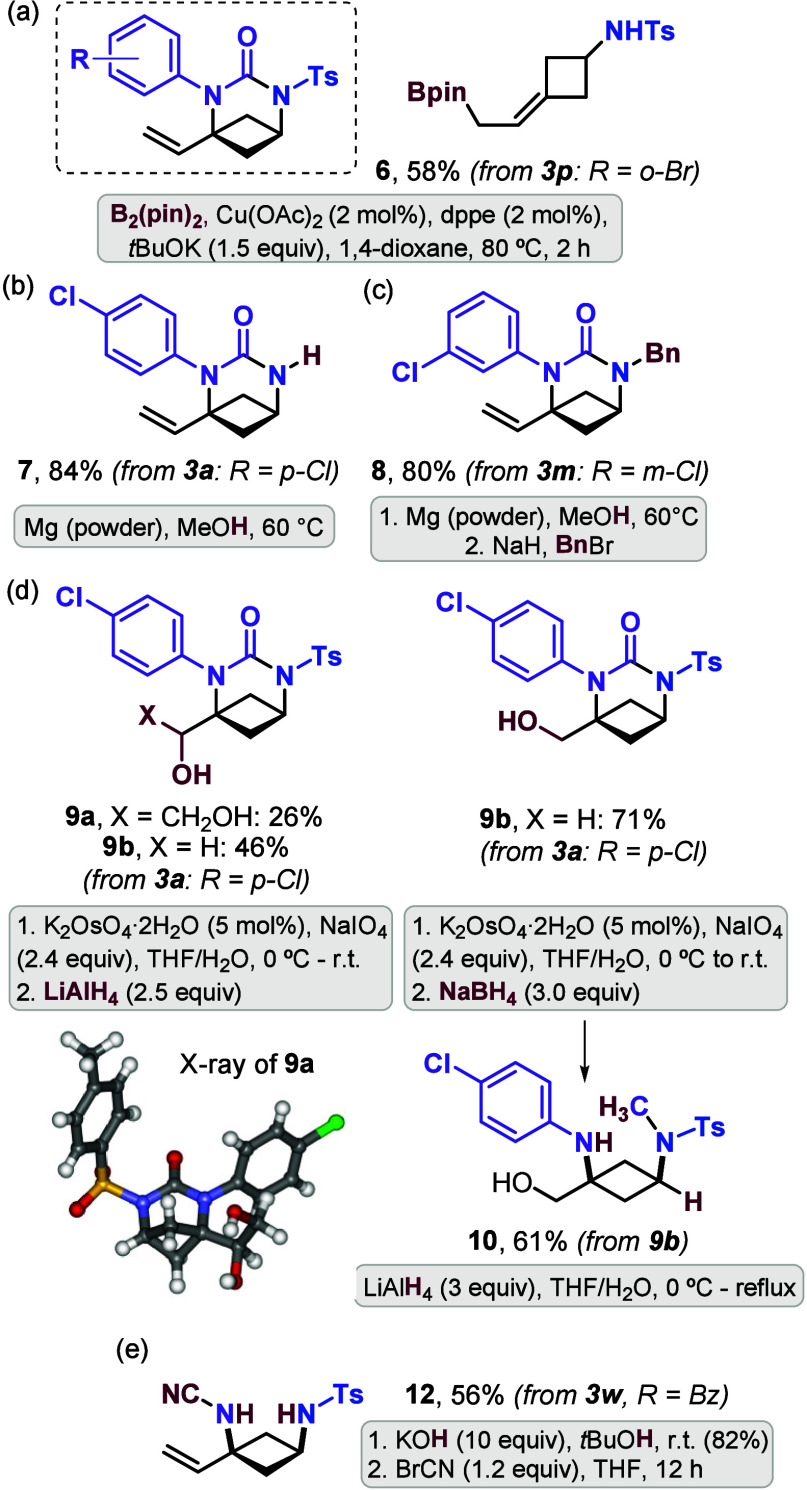
Post-Synthetic Utility Studies

2,4-Diazabicyclo­[3.1.1]­heptan-3-one **3a** was further subjected to several different oxidation and/or reduction conditions (Scheme 4d). Treatment of **3a** under adapted Sharpless dihydroxylation conditions followed by reduction of the mixture in the presence of (excess) LiAlH_4_ produces both diol **9a** (26% yield, X-ray structure provided below under Scheme 4d), and monoalcohol **9b** (46% yield). When LiAlH_4_ is replaced by NaBH_4_ following a similar sequence, **9b** is isolated in 71% with only traces of diol **9a** being formed. Finally, addition of (excess) of LiAlH_4_ to **9b** yielded *syn*-1,3-diaminated cyclobutane derivative **10** in 61% yield. Finally, product **5f** was converted into *syn*-1,3-diaminated cyclobutane **11** (82%, Scheme 4e) featuring a primary amine by basic hydrolysis, and the latter product could be further advanced to *N*-cyanated **12** (56%, in 2 steps from **3w**) by combining it with cyanogen bromide.

To demonstrate that 2,4-diazabicyclo[3.1.1]­heptan-3-ones can be readily introduced into more relevant scaffolds, three multistep sequences were performed (Figure S1, Scheme 5). Similar to the synthesis of **11** (Scheme 4e), 2,4-diazabicyclo[3.1.1]­heptan-3-one **5f** was converted into a diaminated cyclobutane (isolated but not purified) and subsequently reduced in the presence of LiAlH_4_ to provide **13** in 63% overall yield (Figure S1, *top*). The latter was then coupled to chlorinated heterocycle **X** at the more nucleophilic *N*-center of **13** to afford, in two steps, a structural mimic of Abrocitinib (a compound used against eczema) with a diversification handle through the presence of a vinyl group. A second example is shown in Scheme 5 and starts with 2,4-diazabicyclo[3.1.1]­heptan-3-one **3p**. An intramolecular Heck reaction in **3p** under suitable Pd catalysis conditions furnished tetracyclic **15** in 70% yield. *N*-deprotection similar to **7** (Scheme 4b) gave access to **16** in 92% yield. Finally, treatment of **16** with imidazole **Y** under basic conditions provided a 3D drug analogue (**17**: 48%, 2 steps) of a 5-HT3 receptor antagonist^42^ after removal of the trityl *N*-protecting group. In a third case, 2,4-diazabicyclo[3.1.1]­heptan-3-one **3a** was first converted into hydroxymethylene derivative 9b (71%), then transformed into the mesyl-compound 18 (95%), and finally coupled to piperidine **Z** via nucleophilic substitution to give a Rupatadine mimic (**19**) in 52% yield (Figure S1, *below*). These combined examples (Figure S1, and Scheme 5) demonstrate the versatile synthetic nature of the 2,4-diazabicyclo[3.1.1]­heptan-3-one synthons that are available through skeletal editing of bicyclic carbamate precursors.

**Scheme 5:**
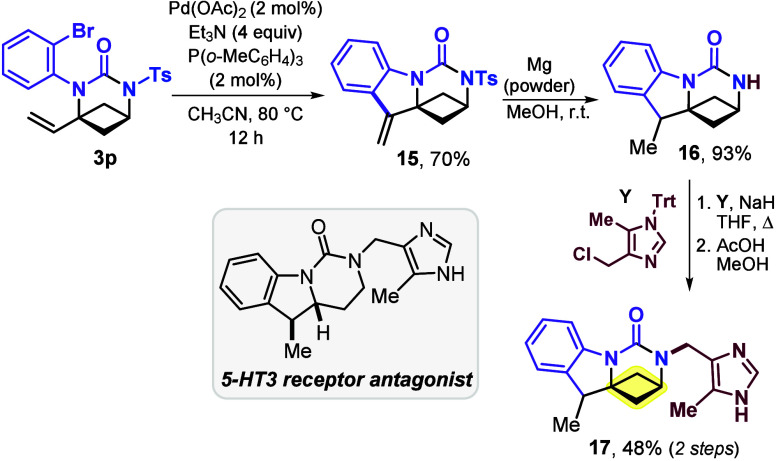
Synthesis of Potential 3D Bioisosteres

In summary, we present an effective strategy to prepare 2,4-diazabicyclo[3.1.1]­heptan-3-ones from carbamate precursors via a formal Pd-catalyzed skeletal editing approach using modular bicyclic carbamates as *de novo* substrates for bioisosteric development. The process combines fast reaction times, user-friendly reaction conditions, structural diversity in the products and application potential in the synthesis of structural pyrimidine/2-pyrimidinone drug mimics.

## Supplementary Material



## Data Availability

The data underlying this study are available in the published article and its Supporting Information.

## Notes

Experimental procedures, full spectroscopic data for all new compounds, and copies of NMR and IR spectra, and X-ray crystal structure data (PDF)

## References

[1] Ertl P., Altmann E., McKenna J. M. (2020). The Most Common Functional Groups
in Bioactive Molecules and How Their Popularity Has Evolved over Time. J. Med. Chem..

[2] Marshall C. M., Federice J. G., Bell C. N., Cox F. B., Njardarson J. T. (2024). An Update
on the Nitrogen Heterocycle Compositions and Properties of U.S. FDA-Approved
Pharmaceuticals (2013–2023). J. Med.
Chem..

[3] Kumar S., Narasimhan B. (2018). Therapeutic Potential of Heterocyclic Pyrimidine Scaffolds. Chem. Cent. J..

[4] Kang D., Sun Y., Feng D., Gao S., Wang Z., Jing L., Zhang T., Jiang X., Lin H., De Clercq E., Pannecouque C., Zhan P., Liu X. (2022). Development
of Novel
Dihydrofuro­[3,4-d]­pyrimidine Derivatives as HIV-1 NNRTIs to Overcome
the Highly Resistant Mutant Strains F227L/V106A and K103N/Y181C. J. Med. Chem..

[5] Zhuang Z., Pan R., Zhang Q., Huang H. (2015). Molecular
recognition of pyrimidine
nucleobases by triplex DNA receptors. Bioorg.
Med. Chem. Lett..

[6] Uhlenbruck B. J. H., Josephitis C. M., de Lescure L., Paton R. S., McNally A. (2024). A Deconstruction-Reconstruction Strategy
for Pyrimidine Diversification. Nature.

[7] Venugopala K. N., Kamat V. (2024). Pyrimidines: A New
Versatile Molecule in the Drug Development Field,
Scope, and Future Aspects. Pharmaceuticals.

[8] Lovering F., Bikker J., Humblet C. (2009). Escape from
Flatland: Increasing
Saturation as an Approach to Improving Clinical Success. J. Med. Chem..

[9] Stepan A. F., Subramanyam C., Efremov I. V., Dutra J. K., O’Sullivan T. J., DiRico K. J., McDonald W. S., Won A., Dorff P. H., Nolan C. E., Becker S. L., Pustilnik L. R., Riddell D. R., Kauffman G. W., Kormos B. L., Zhang L., Lu Y., Capetta S. H., Green M. E., Karki K., Sibley E., Atchison K. P., Hallgren A. J., Oborski C. E., Robshaw A. E., Sneed B., O’Donnell C. J. (2012). Application of the Bicyclo[1.1.1]­pentane
Motif as a Nonclassical Phenyl Ring Bioisostere in the Design of a
Potent and Orally Active γ-Secretase Inhibitor. J. Med. Chem..

[10] Che J.-T., Ding W.-Y., Zhang H.-B., Wang Y.-B., Xiang S.-H., Tan B. (2025). Enantioselective Synthesis of 2-Substituted Bicyclo[1.1.1]­pentanes
via Sequential Asymmetric Imine Addition of Bicyclo[1.1.0]­butanes
and Skeletal Editing. Nat. Chem..

[11] Tian D., Pan Y., Zhao X., Yin Y., Jiang Z. (2025). Chiral Lewis Acid-Catalyzed
Intramolecular [2 + 2] Photocycloaddition: Enantioselective Synthesis
of Azaarene-Functionalized Azabicyclo[2.1.1]­hexanes and Bicyclo[1.1.1]­pentanes. J. Am. Chem. Soc..

[12] Denisenko A., Garbuz P., Shishkina S. V., Voloshchuk N. M., Mykhailiuk P. K. (2020). Saturated Bioisosteres of ortho-Substituted Benzenes. Angew. Chem., Int. Ed..

[13] Dibchak D., Snisarenko M., Mishuk A., Shablykin O., Bortnichuk L., Klymenko-Ulianov O., Kheylik Y., Sadkova I. V., Rzepa H. S., Mykhailiuk P. K. (2023). General Synthesis of 3-Azabicyclo[3.1.1]­heptanes
and Evaluation of Their Properties as Saturated Isosteres. Angew. Chem., Int. Ed..

[14] Garrido-García P., Quirós I., Milán-Rois P., Ortega-Gutiérrez S., Martín-Fontecha M., Campos L. A., Somoza Á., Fernández I., Rigotti T., Tortosa M. (2025). Enantioselective
Photocatalytic Synthesis of Bicyclo[2.1.1]­hexanes as Ortho-Disubstituted
Benzene Bioisosteres with Improved Biological Activity. Nat. Chem..

[15] Frank N., Nugent J., Shire B. R., Pickford H. D., Rabe P., Sterling A. J., Zarganes-Tzitzikas T., Grimes T., Thompson A. L., Smith R. C., Schofield C. J., Brennan P. E., Duarte F., Anderson E. A. (2022). Synthesis of Meta-Substituted
Arene Bioisosteres from
[3.1.1]­Propellane. Nature.

[16] Yu T., Yang J., Wang Z., Ding Z., Xu M., Wen J., Xu L., Li P. (2023). Selective [2σ+2σ] Cycloaddition
Enabled by Boronyl Radical Catalysis: Synthesis of Highly Substituted
Bicyclo[3.1.1]­heptanes. J. Am. Chem. Soc..

[17] Liang Y., Nematswerani R., Daniliuc C. G., Glorius F. (2024). Silver-Enabled Cycloaddition
of Bicyclobutanes with Isocyanides for the Synthesis of Polysubstituted
3-Azabicyclo[3.1.1]­heptanes. Angew. Chem., Int.
Ed..

[18] Liu Y., Lin S., Ding Z., Li Y., Tang Y.-J., Xue J.-H., Li Q., Li P., Wang H. (2024). Pyridine-Boryl Radical-Catalyzed
[3π + 2σ] Cycloaddition for the Synthesis of Pyridine
Isosteres. Chem..

[19] Lin Z., Ren H., Lin X., Yu X., Zheng J. (2024). Synthesis of Azabicyclo[3.1.1]­heptenes
Enabled by Catalyst-Controlled Annulations of Bicyclo[1.1.0]­butanes
with Vinyl Azide. J. Am. Chem. Soc..

[20] Wu W.-B., Xu B., Yang X.-C., Wu F., He H.-X., Zhang X., Feng J.-J. (2024). Enantioselective
Formal (3 + 3) Cycloaddition of Bicyclobutanes
with Nitrones Enabled by Asymmetric Lewis Acid Catalysis. Nat. Commun..

[21] Zhang J., Su J.-Y., Zheng H., Li H., Deng W.-P. (2024). Eu­(OTf)_3_-Catalyzed Formal Dipolar [4π+2σ]
Cycloaddition
of Bicyclo-[1.1.0]­butanes with Nitrones: Access to Polysubstituted
2-Oxa-3-azabicyclo[3.1.1]­heptanes. Angew. Chem.,
Int. Ed..

[22] Zhang X.-G., Zhou Z.-Y., Li J.-X., Chen J.-J., Zhou Q.-L. (2024). Copper-Catalyzed
Enantioselective [4π + 2σ] Cycloaddition of Bicyclobutanes
with Nitrones. J. Am. Chem. Soc..

[23] Wang X., Gao R., Li X. (2024). Catalytic
Asymmetric Construction of Chiral Polysubstituted
3-Azabicyclo[3.1.1]­heptanes by Copper-Catalyzed Stereoselective Formal
[4π+2σ] Cycloaddition. J. Am. Chem.
Soc..

[24] Jiang Q., Dong J., Zhou X., Liao H., Zhou J., Xue D. (2024). Lewis-Acid-Catalyzed Dearomative [4π + 2σ] Cycloaddition
of Bicyclobutanes with Isoquinolinium Methylides for the Synthesis
of Ring-Fused Azabicyclo[3.1.1]­heptanes. Org.
Lett..

[25] Chintawar C. C., Laskar R., Rana D., Schäfer F., van Wyngaerden N., Dutta S., Daniliuc C. G., Glorius F. (2024). Photoredox-catalysed
amidyl radical insertion to bicyclo[1.1.0]­butanes. Nat. Catal..

[26] Chen Y., Zhou Y.-T., Ge Z., Qin L., Zhu S.-E., Xu H.-J., Xu J. (2026). Iodide-Catalyzed Intermolecular
Formal
(3 + 3) Cycloaddition of Bicyclo[1.1.0]­butanes and Amides: Modular
Access to Heteroatom-Enriched Bicyclo[3.1.1]­heptane Scaffolds. Org. Lett..

[27] Revie R. I., Ragus J., Anderson E. A. (2026). Synthesis of Heterobicyclo­[*n*.1.1]­alkanes. Chem. Soc. Rev..

[28] Shire B. R., Anderson E. A. (2023). Conquering the Synthesis
and Functionalization of Bicyclo[1.1.1]­pentanes. JACS Au.

[29] Xu M., Wang Z., Sun Z., Ouyang Y., Ding Z., Yu T., Xu L., Li P. (2022). Diboron­(4)-Catalyzed Remote [3 +
2] Cycloaddition of Cyclopropanes via Dearomative/Rearomative Radical
Transmission through Pyridine. Angew. Chem.,
Int. Ed..

[30] Zhou J.-L., Zhan X., He H.-X., Xu Y., Peng Q., Huang G., Feng J.-J. (2025). Catalytic Activation of Acceptor–Acceptor
Bicyclobutanes Enabled by Lewis Base Catalysis. Angew. Chem., Int. Ed..

[31] Li T., Wang Y., Xu Y., Ren H., Lin Z., Li Z., Zheng J. (2024). Zwitterionic π**-**Allyl-Pd Species
Enabled [2σ+2π] Cycloaddition Reactions of Vinylbicyclo[1.1.0]­butanes
(VBCBs) with Alkenes, Carbonyls, and Imines. ACS Catal..

[32] Liu Y., Wu Z., Shan J.-R., Yan H., Hao E.-J., Shi L. (2024). Titanium Catalyzed
[2σ+2π] Cycloaddition of Bicyclo[1.1.0]-Butanes with 1,3-Dienes
for efficient Synthesis of Stilbene Bioisosteres. Nat. Commun..

[33] Fu Q., Cao S., Wang J., Lv X., Wang H., Zhao X., Jiang Z. (2024). Enantioselective [2π+2σ]
Cycloadditions of Bicyclo[1.1.0]­butanes
with Vinylazaarenes through Asymmetric Photoredox Catalysis. J. Am. Chem. Soc..

[34] Guo W., Gómez J. E., Cristòfol À., Xie J., Kleij A. W. (2018). Catalytic
Transformations of Functionalized Cyclic Organic Carbonates. Angew. Chem., Int. Ed..

[35] Guo W., Kuniyil R., Gómez J. E., Maseras F., Kleij A. W. (2018). A Domino
Process toward Functionally Dense Quaternary Carbons through Pd-Catalyzed
Decarboxylative C­(sp^3^)–C­(sp^3^) Bond Formation. J. Am. Chem. Soc..

[36] Cai A., Guo W., Martínez-Rodríguez L., Kleij A. W. (2016). Palladium-Catalyzed
Regio- and Enantioselective Synthesis of Allylic Amines Featuring
Tetrasubstituted Tertiary Carbons. J. Am. Chem.
Soc..

[37] Wang C., Tunge J. A. (2008). Asymmetric Cycloadditions of Palladium-Polarized Aza-o-xylylenes. J. Am. Chem. Soc..

[38] Yi Z., Xiao W., Jie J., Yang H., Fu H. (2025). Ligand-Enabled
Palladium-Catalyzed Asymmetric Synthesis of Cyclic Sulfamidate-Fused
Imidazolidinones. Org. Lett..

[39] Spielmann K., van der Lee A., Marcia de Figueiredo R., Campagne J.-M. (2018). Diastereoselective
Palladium-Catalyzed (3 + 2)-Cycloadditions from Cyclic Imines and
Vinyl Aziridines. Org. Lett..

[40] Dutta S., Lu Y.-L., Erchinger J. E., Shao H., Studer E., Schäfer F., Wang H., Rana D., Daniliuc C. G., Houk K. N., Glorius F. (2024). Double Strain-Release [2π+2σ]-Photocycloaddition. J. Am. Chem. Soc..

[41] Wang W., Xiao J. A., Zheng L., Liang W.-J., Yang L., Huang X.-X., Lin C., Chen K., Su W., Yang H. (2024). Structure-Dependent, Switchable Alder-Ene/[2π + 2σ] Cycloadditions
of Vinyl Bicyclo[1.1.0]­butanes with α-Ketoesters Enabled by
Palladium Catalysis. Org. Lett..

[42] Kato M., Nishino S., Ito K., Yamakuni H., Takasugi H. (1994). New 5-HT_3_ (Serotonin-3)
Receptor Antagonists. II. Synthesis and Structure-Activity
Relationships of Pyrimido­[1,6-α]­indoles. Chem. Pharm. Bull..

