# Supplementary material for: Pd-Catalyzed De Novo Skeletal Editing of Bicyclic Carbamates Delivers 2‑Pyrimidinones
Source: Org Lett. 2026 Jul 6;28(28):8952–7. doi: 10.1021/acs.orglett.6c02359 (PMC13386539; doi:10.1021/acs.orglett.6c02359)
Supplement: Supplementary file 1 [file ol6c02359_si_001.pdf]

Supplementary Information:

Pd-Catalyzed De Novo Skeletal Editing of Bicyclic Carbamates delivers 2-Pyrimidinones

Wangyu Shi,[†,‡] Yue Ren,[†,‡] Jordi Benet-Buchholz,[†] Maria Vicent Morales[†] and Arjan W. Kleij*[†,#]

[†] Institute of Chemical Research of Catalonia (ICIQ-Cerca), the Barcelona Institute of Science and Technology (BIST), Tarragona, Spain. Email: akleij@iciq.es

[‡] Departament de Química Física i Inorgànica, Universitat Rovira i Virgili, Tarragona, Spain

[#] Catalan Institute of Research and Advanced Studies (ICREA), Barcelona, Spain

Contents

1. General comments 3
2. Experimental procedures 4
   Experimental procedures for the synthesis of bicyclic carbamates **1** 4
   General experimental procedure for the synthesis of 1-vinyl-2,4-diazabicyclo-[3.1.1]heptan-3-ones **3** 6
   Experimental procedure for the scale-up synthesis of **3p** 7
   Experimental procedure for the scale-up synthesis of **3w** 8
   Experimental procedure for the scale-up synthesis of **5f** 9
   Experimental procedures for the post-modification reactions 10
3. Full process screening data 24
4. Characterization data for all new compounds 26
   Bicyclic carbamate substrates 26
   2,4-Diazabicyclo[3.1.1]heptan-3-one products 31
   Post-modification products 42
   Drug-mimic syntheses 45
5. Copies of NMR spectra 48
6. X-ray analysis and comments 111
7. References 116

1. General comments

All Pd-catalyzed reactions were carried out under a nitrogen atmosphere in oven-dried glassware. Heating of the reaction mixtures was achieved through an oil bath and facilitated by a magnetic stirring plate using an IKA RCT basic magnetic stirring hotplate. All reagents were used as received from commercial suppliers (Aldrich, Acros, or TCI) unless otherwise stated. Flash column chromatography was carried out on Sigma-Aldrich silica gel 60 (70-230 mesh). NMR-spectra were recorded on Bruker AV-300, AV-400, or AV-500 spectrometers. The residual solvent signals were used as references for ^{1}H and ^{13}C NMR spectra ($CDCl_3$: δH = 7.26 ppm, δC = 77.16 ppm, $(CD_3)_2SO$: δ^H = 2.5 ppm, δ^C = 39.52 ppm). ^{19}F NMR spectra were externally referenced. FT-IR measurements were carried out on a Bruker Optics FTIR-ATR TR0 spectrometer. High Resolution Mass Spectrometry (HRMS) were recorded by the Research Support Area (RSA) at ICIQ on a MicroTOF II (Bruker Daltonics) using electrospray ionization(ESI) source or MaXis impact (Bruker Daltonics) using an atmospheric pressure chemical ionization (APCI) source. Solvents were dried using an Innovative Technology PURE SOLV solvent purification system.

2. Experimental procedures

Experimental procedures for the synthesis of bicyclic carbamates **1**

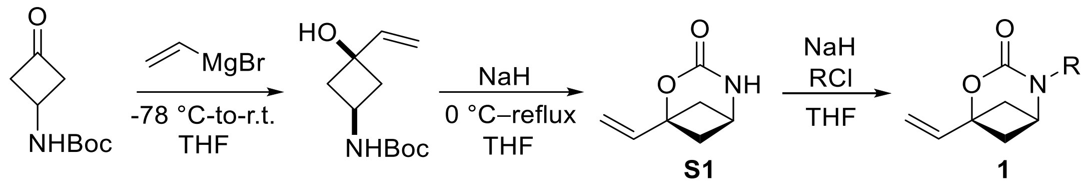

Under a N_2 atmosphere, a vinylmagnesium bromide solution (22 mL, 1 M in THF, 1.1 equiv) was slowly added to a solution of *tert*-butyl 3-oxocyclobutylcarbamate (1.85 g, 10 mmol, 1.0 equiv) in 20 mL anhydrous THF at -78 °C. After the addition, the reaction mixture was allowed to reach room temperature and stirred for another 1 h. It was then quenched with a saturated solution of NH_4Cl. The aqueous phase was extracted with ethyl acetate (EtOAc, 3 × 20 mL), and then the organic phases were combined, dried over Na_2SO_4, filtered, and concentrated under reduced pressure. The residue was purified by flash silica column chromatography employing 33% EtOAc in hexane as eluent to afford the desired product (typically with a 50-60 % yield).

The freshly prepared product from the first step (typically 1.07 g, 5.0 mmol) was dissolved in anhydrous THF (0.33 M). At 0 °C, sodium hydride (2.0 equiv) was added portion-wise. After that, the mixture was refluxed at 70 °C until the starting material had been fully consumed as detected by TLC. Then the reaction mixture was allowed to cool down to room temperature and subsequently quenched by the addition of water. The aqueous phase was extracted with EtOAc (3 × 20 mL), and the organic phases were dried over anhydrous Na_2SO_4, filtered, and concentrated under reduced pressure. The residue was purified by flash silica column chromatography employing 100% EtOAc in hexane as eluent to afford 1-vinyl-2-oxa-4-azabicyclo[3.1.1]heptan-3-one **S1** as a light brown amorphous solid (typically 420 mg, which is ~60% yield).

1-Vinyl-2-oxa-4-azabicyclo[3.1.1]heptan-3-one **S1** was dissolved in anhydrous THF (0.33 M). At 0 °C, sodium hydride (2.0 equiv) was added portion-wise. After stirring for 15 minutes at the same temperature, the respective RCl reagent (1.2 equiv) was added in one portion. The mixture was stirred until the starting material had been fully consumed as detected by TLC (note that for R = 4-methoxy-benzylchloride, the reaction mixture

was stirred at 70 °C). Then the reaction was quenched by the addition of water. The aqueous phase was extracted with EtOAc (3 × 20 mL), and the organic phases were combined, washed with saturated aqueous NaCl, and then dried over anhydrous Na_2SO_4. After filtration and concentration, the crude product was purified by flash silica column chromatography employing EtOAc in hexane as eluent to afford the *N*-protected 1-vinyl-2-oxa-4-azabicyclo[3.1.1]heptan-3-one as a white amorphous solid.

General experimental procedure for the synthesis of 1-vinyl-2,4-diazabicyclo-[3.1.1]heptan-3-ones **3**

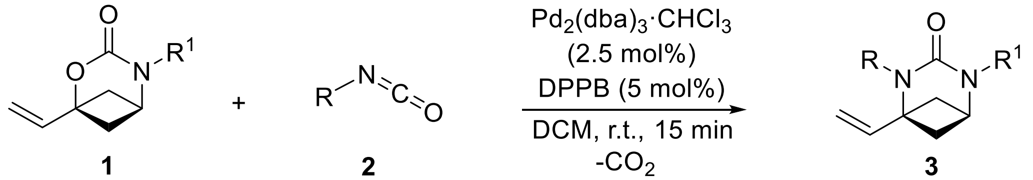

Under a N_2 atmosphere, to a vial charged with $Pd_2(dba)_3 \cdot CHCl_3$ (2.6 mg, 0.0025 mmol, 2.5 mol%), 1,4-bis(diphenylphosphino)butane (DPPB, 2.1 mg, 0.0050 mmol, 5 mol%), and 1-vinyl-2-oxa-4-azabicyclo[3.1.1]heptan-3-one derivatives **1** (0.10 mmol, 1.0 equiv) was added a solution of the corresponding isocyanate **2** (0.12 mmol **2** in 1 mL of the solvent). The resulting mixture was stirred at room temperature until the starting material had been fully consumed as detected by TLC, which was typically in ≤15 min. Then, the mixture was purified by flash silica column chromatography employing EtOAc in hexane as eluent (NOTE: exact ratio indicated for each compound below) to afford the respective 1-vinyl-2,4-diazabicyclo[3.1.1]heptan-3-one **3**. For products **5b** and **5g**, the reaction time was 24 h.

Experimental procedure for the scale-up synthesis of **3p**

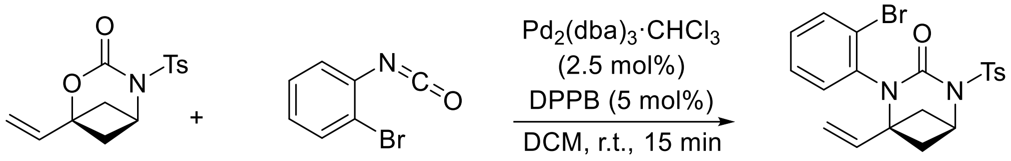

1a **2p** **3p**

Under a N_2 atmosphere, to a Schlenk flask charged with Pd$_2$(dba)$_3$·CHCl$_3$ (25.5 mg, 0.025 mmol, 2.5 mol%), 1,4-bis(diphenylphosphino)butane (DPPB, 21.3 mg, 0.050 mmol, 5 mol%), and **1a** (293.3 mg, 1.0 mmol, 1.0 equiv) was added a solution of **2p** (1.2 mmol **2p** in 10 mL of DCM). The resulting mixture was stirred at room temperature for 15 min. Then, the mixture was concentrated under reduced pressure and purified by flash silica column chromatography employing 25% EtOAc in hexane as eluent to afford **3p** (321.5 mg, 72% yield).

Experimental procedure for the scale-up synthesis of **3w**

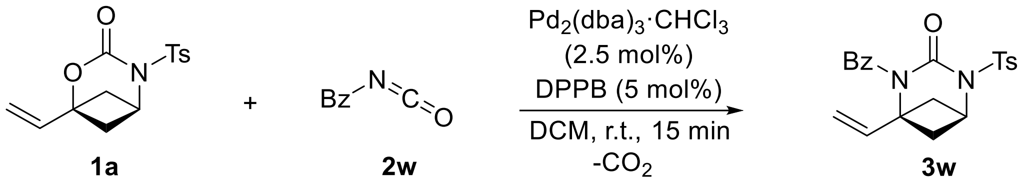

1a **2w** **3w**

Under a N_2 atmosphere, to a Schlenk flask charged with $Pd_2(dba)_3 \cdot CHCl_3$ (25.5 mg, 0.025 mmol, 2.5 mol%), 1,4-Bis(diphenylphosphino)butane (DPPB, 21.3 mg, 0.050 mmol, 5 mol%), and **1g** (293.3 mg, 1.0 mmol, 1.0 equiv) was added the solution of **2w** (1.2 mmol **2w** in 10 mL of DCM). The resulting mixture was stirred at room temperature for 15 min. Then, the mixture was concentrated under reduced pressure and purified by flash silica column chromatography employing 25% EtOAc in hexane as eluent to afford **3w** (335.8 mg, 85% yield).

Experimental procedure for the scale-up synthesis of **5f**

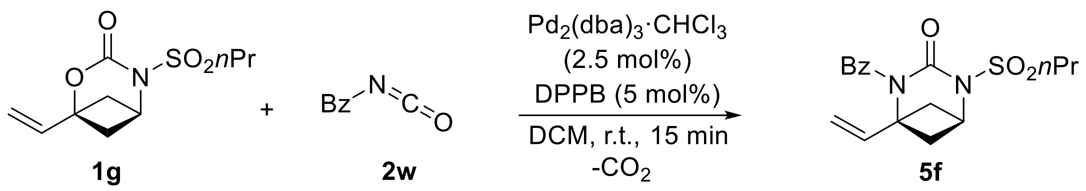

Under a N_2 atmosphere, to a Schlenk flask charged with $Pd_2(dba)_3$·$CHCl_3$ (25.5 mg, 0.025 mmol, 2.5 mol%), 1,4-bis(diphenylphosphino)butane (DPPB, 21.3 mg, 0.050 mmol, 5 mol%), and **1g** (245.3 mg, 1.0 mmol, 1.0 equiv) was added a solution of **2w** (1.2 mmol **2w** in 10 mL of DCM). The resulting mixture was stirred at room temperature for 15 min. Then, the mixture was concentrated under reduced pressure and purified by flash silica column chromatography employing 25% EtOAc in hexane as eluent to afford **3w** (278.6 mg, 80% yield).

Experimental procedures for the post-modification reactions

*Synthesis of boronated product **6***

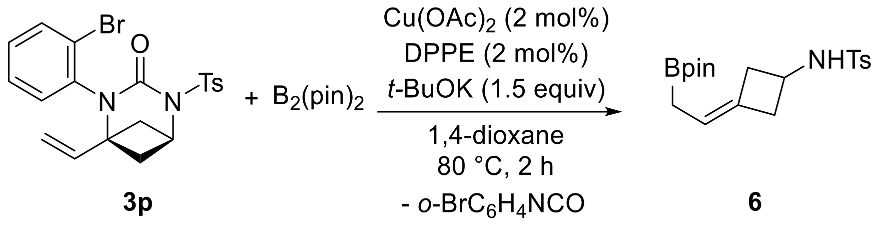

Under a N_2 atmosphere, to a Schlenk tube charged with **3p** (0.10 mmol, 44.7 mg), $Cu(OAc)_2$ (0.4 mg, 0.002 mmol, 2 mol%), 1,2-bis(diphenylphosphino)ethane (DPPE, 0.8 mg, 0.002 mmol, 2 mol%), bis(pinacolato)diboron ($Bpin_2$, 38.1 mg, 0.15 mmol, 1.5 equiv), *t*-BuOK (16.8 mg, 0.15 mmol, 1.5 equiv) was added 1 mL of dry 1,4-dioxane. The resulting mixture was stirred at 80 °C for 2 h. After that time, the solvent was removed under reduced pressure, and the residue was purified by flash silica column chromatography employing 17% EtOAc in hexane as eluent to afford **6** (21.8 mg, 58% yield) as a colorless oil.

*N-Deprotection of **3a** to afford of **7***

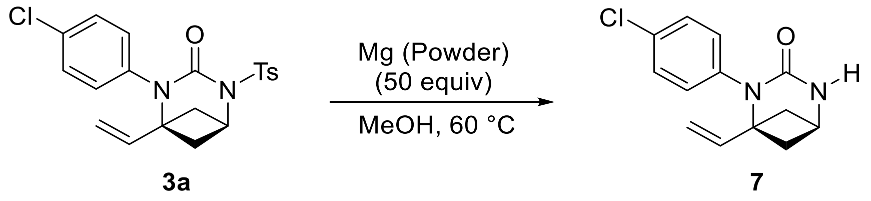

Under an Ar atmosphere, to a Schlenk tube charged with **3a** (40.3 mg, 0.10 mmol) and Mg powder (120.0 mg, 5.0 mmol, 200-300 mesh) was added anhydrous MeOH (3 mL). The resulting mixture was stirred at room temperature until it started to release a lot of heat. After that, the reaction was stirred at 60 °C until the starting material had been fully consumed as detected by TLC. Then saturated NH_4Cl (2 mL) was added to the reaction mixture to quench any excess of magnesium powder that remained. The aqueous phase was extracted with EtOAc (3 × 20 mL), and the organic phases were dried over Na_2SO_4. After filtration and concentration, the residue was purified by flash silica column chromatography first employing a 2:1 mixture of EtOAc and hexane, following by 100% EtOAc as eluent to afford **7** (20.8 mg, 84% yield) as a white amorphous solid.

*Cascade N-deprotection/benzylation of **3m** to give **8***

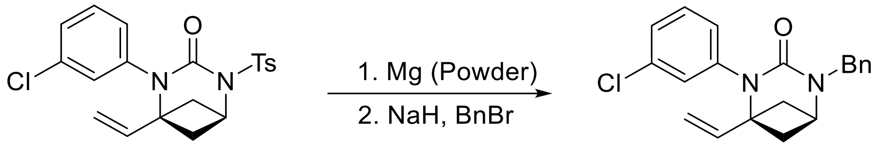

Under an Ar atmosphere, to a Schlenk tube charged with **3m** (120.9 mg, 0.30 mmol) and Mg powder (320.0 mg, 15.0 mmol, 200-300 mesh) was added anhydrous MeOH (9 mL). The resulting mixture was stirred at room temperature until it started to release a lot of heat. After that, the reaction mixture was allowed to stir at 60 °C until the starting material had been fully consumed as detected by TLC. Subsequently, saturated aqueous NH_4Cl (6 mL) was added to the reaction mixture to quench the excess of magnesium powder that remained. The aqueous phase was extracted with ethyl acetate EtOAc (3 × 20 mL), and the organic phases were dried over Na_2SO_4. After filtration and concentration, the residue was dissolved into 1.5 mL of anhydrous THF. Under 0 °C, to this solution was added NaH (60 wt% in mineral oil, 14.4 mg, 0.36 mmol, 1.2 equiv), and after the mixture had been stirred at the same temperature for 5 minutes, BnBr (61.6 mg, 0.36 mmol, 1.2 equiv) was added. Then, the reaction was heated to 70 °C and stirred for another 16 hours. After cooling down, the reaction was quenched by the addition of water. The aqueous phase was extracted with EtOAc (3 × 20 mL), and the organic phases were washed with saturated aqueous NaCl and dried over anhydrous Na_2SO_4. After filtration and concentration, the residue was purified by flash silica column chromatography employing 17-25% EtOAc in hexane as eluent to afford **8** (81.4 mg, 80% yield in two steps) as a white amorphous solid.

*Synthesis of **9a** and **9b** from **3a*** [1]

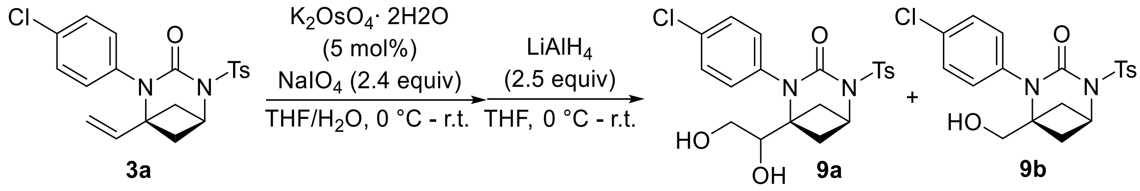

3a **9a** **9b**

To a solution of **1a** (201.4 mg, 0.50 mmol, 1.0 equiv) in THF (5.0 mL) and H_2O (2.5 mL) was added $NaIO_4$ (256.5 mg, 4.8 mmol, 2.4 equiv). The reaction mixture was cooled with iced water, then $K_2OsO_4 \cdot 2H_2O$ (9.0 mg, 0.025 mmol, 5 mol%) was added. After the addition, the reaction mixture was warmed to room temperature and stirred for 16 h. After reaction completion as monitored by TLC, the mixture was quenched with aqueous $Na_2S_2O_3$ (30 mL) and stirred for 30 mins. The biphasic reaction mixture was then extracted with EtOAc (3 × 20 mL), and the combined organic layers were washed with brine, dried over anhydrous Na_2SO_4, filtered, and concentrated. The filtrate was evaporated under reduced pressure, and the residue was dissolved in anhydrous THF. At 0 °C, to this solution was added $LiAlH_4$ (1.25 mL, 1 M in THF, 2.5 equiv). The resulting mixture was stirred at room temperature until the starting material had been fully consumed as detected by TLC. Then water (10 mL) and saturated aqueous potassium sodium tartrate (10 mL) were added successively to quench the reaction. The aqueous phase was extracted with EtOAc (3 × 20 mL), and the organic phases were dried over Na_2SO_4. After filtration and concentration, the residue was purified by flash silica column chromatography employing 1:1 to 2:1 EtOAc/hexane as eluent to afford **9a** (57.0 mg, 26% yield) as a white amorphous solid, and **9b** in 44% yield.

*Synthesis of **9b** from **3a***

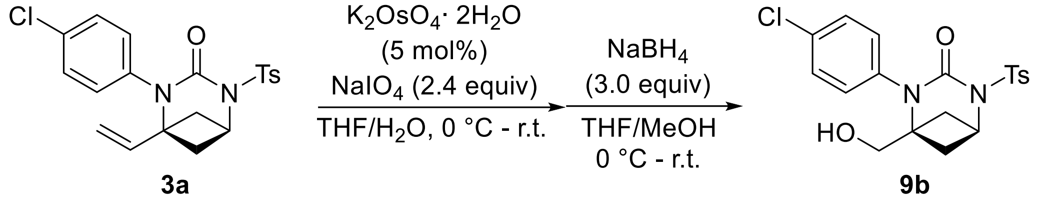

To a solution of **3a** (201.4 mg, 0.50 mmol, 1.0 equiv) in THF (5.0 mL) and H_2O (2.5 mL) was added $NaIO_4$ (256.5 mg, 4.8 mmol, 2.4 equiv). The reaction mixture was cooled with iced water, then $K_2OsO_4{\cdot}2H_2O$ (9.0 mg, 0.025 mmol, 5 mol%) was added. The reaction mixture was then warmed to room temperature and stirred for 16 h. After reaction completion as monitored by TLC, the mixture was then quenched with aqueous $Na_2S_2O_3$ (30 mL) and stirred for 30 mins. The biphasic reaction mixture was then extracted with EtOAc (3 × 20 mL), and the combined organic layers were washed with brine, dried over anhydrous Na_2SO_4, filtered, and concentrated. The filtrate was evaporated under reduced pressure, and the residue was dissolved into THF/MeOH (4:1 v/v, 5 mL). At 0 °C, to this solution was added $NaBH_4$ (56.7 mg, 1.5 mmol, 3.0 equiv). The resulting mixture was stirred at room temperature until the starting material had been fully consumed as detected by TLC. Then water (10 mL) was added to quench the reaction. The aqueous phase was extracted with EtOAc (3 × 20 mL), and the organic phases were dried over Na_2SO_4. After filtration and concentration, the residue was purified by flash silica column chromatography employing 50-100% EtOAc in hexane as eluent to afford **9b** (143.8 mg, 71% yield) as a white amorphous solid.

*Reduction of **9b** to give **10***

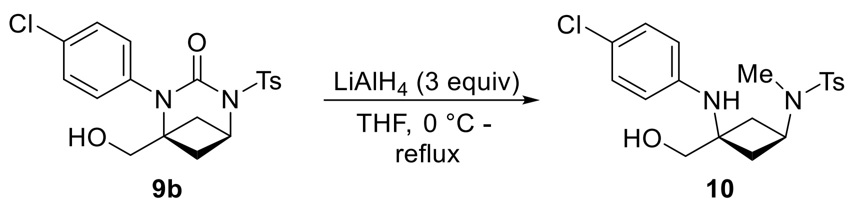

Under 0 °C, to a solution of **9b** (122.1 mg, 0.30 mmol, 1.0 equiv) in 3 mL of THF was added $LiAlH_4$ (0.9 mL, 1 M in THF, 3.0 equiv). The reaction mixture was then stirred at 70 °C for 4 h. After reaction completion as monitored by TLC, the mixture was cooled to room temperature and then quenched with water (10 mL) and saturated aqueous potassium sodium tartrate (10 mL). The aqueous phase was extracted with EtOAc (3 × 20 mL), and the organic phases were dried over Na_2SO_4. After filtration and concentration, the residue was purified by flash silica column chromatography employing 33-50% EtOAc in hexane as eluent to afford **10** (72.3 mg, 61% yield) as a white amorphous solid.

*Nucleophilic addition-ring opening of **3w** to prepare **11***

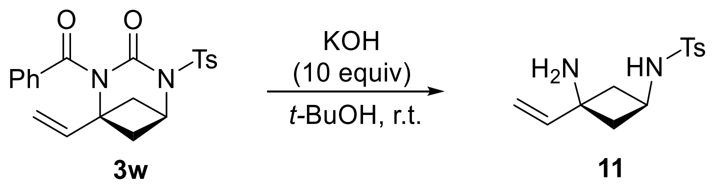

Compound **3w** (39.6 mg, 0.10 mmol) was dissolved in 2 mL of *t*-BuOH. To this solution was added KOH (56.1 mg, 1.0 mmol, 10 equiv). The resulting mixture was stirred at room temperature for 2 h and then concentrated under reduced pressure. To the residue was added 5 mL of water, and the aqueous phase was extracted with EtOAc (3 × 20 mL). The organic phases were washed with saturated aqueous NaCl and then dried over anhydrous Na_2SO_4. After filtration and concentration, the residue was purified by flash silica column chromatography employing 5-10% MeOH in DCM as eluent to afford **11** (21.9 mg, 82% yield) as a white amorphous solid.

*Synthesis of **12** from **11*** [2]

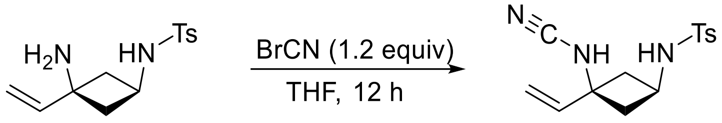

11 **12**

Primary amine **11** was dissolved in anhydrous THF (2 mL), followed by the addition of BrCN (21.1 mg, 0.20 mmol) and Et_3N (15.2 mg, 0.15 mmol). The reaction mixture was stirred at room temperature for 8 h, and 5 mL saturated aqueous NH_4Cl was added. The aqueous phase was extracted with DCM. The combined organic phases were dried over anhydrous Na_2SO_4, filtered, and concentrated. The residue was purified by flash silica column chromatography employing 50% EtOAc in hexane as eluent to afford **12** (18.8 mg, 68% yield) as a light-yellow oil.

Figure S1

Figure S1: Top

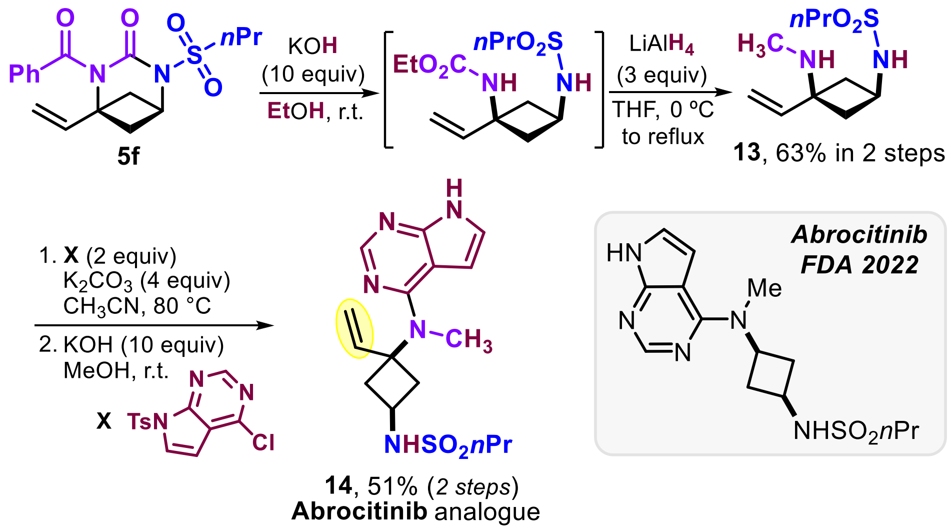

Figure S1: Below

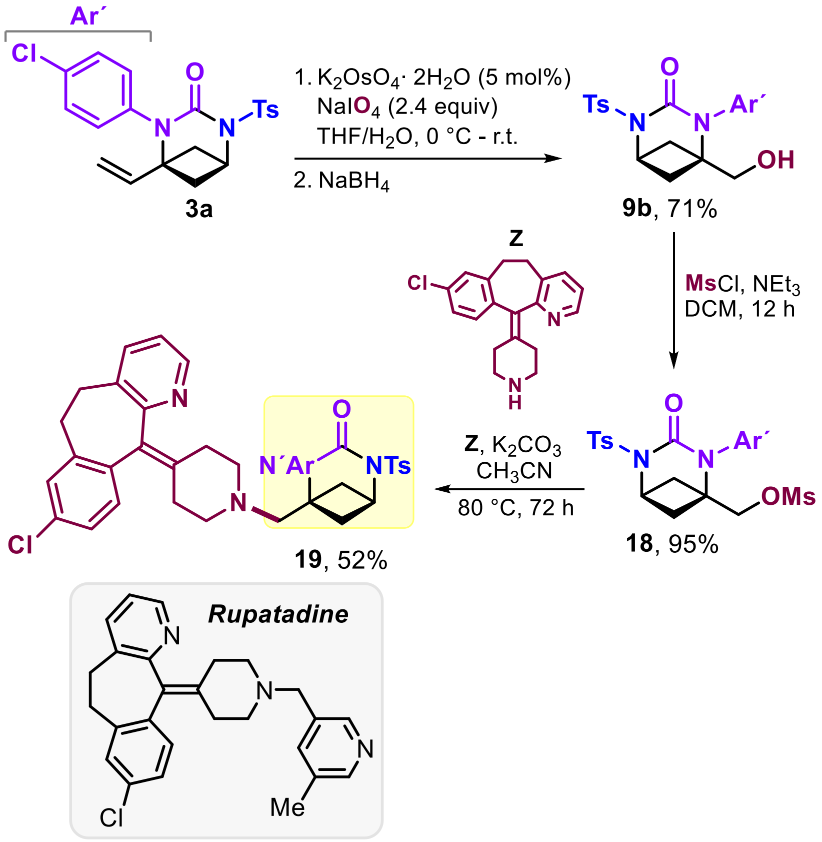

*Synthesis of Abrocitinib analogue **14*** [3]

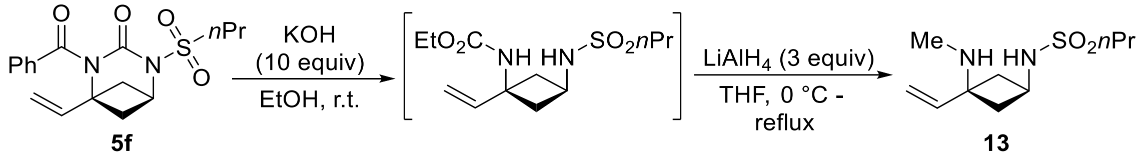

Compound **5f** (139.4 mg, 0.40 mmol) was dissolved in 8 mL of EtOH. To this solution was added KOH (224.4 mg, 4.0 mmol, 10 equiv). The resulting mixture was stirred at room temperature for 2 h and then concentrated under reduced pressure. To the residue was added water (20 mL), and the aqueous phase was extracted with EtOAc (3 × 20 mL). The combined organic phase was washed with saturated aqueous NaCl and then dried over anhydrous Na_2SO_4. After filtration and concentration, the residue was dissolved in 4 mL of THF and cooled to 0 °C. To this solution was added $LiAlH_4$ (1.2 mL, 1 M in THF, 3.0 equiv). The reaction mixture was then stirred at 70 °C for 4 h. After reaction completion as monitored by TLC, the mixture was cooled to room temperature and then quenched with water (10 mL) and saturated aqueous potassium sodium tartrate (10 mL). The aqueous phase was extracted with EtOAc (3 × 20 mL), and the combined organic phases were dried over Na_2SO_4. After filtration and concentration, the residue was purified by flash silica column chromatography employing 10% MeOH in DCM as eluent to afford **13** (58.3 mg, 63% yield) as a colorless oil.

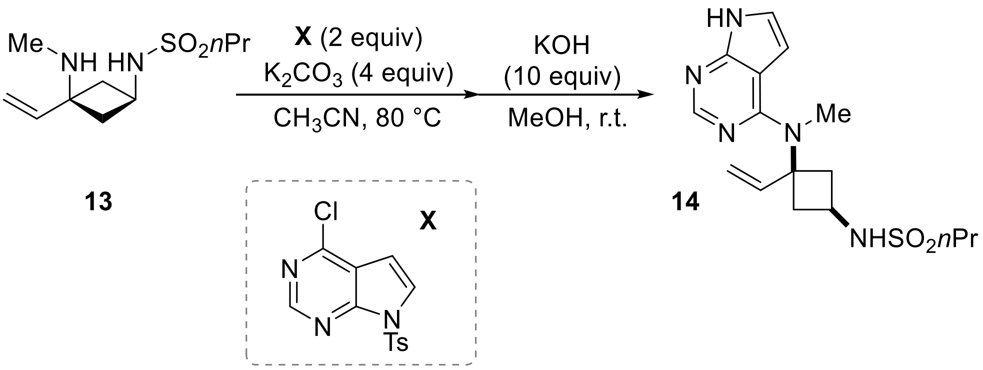

Compound **13** (58.3 mg, 0.25 mmol) and **X** (153.9 mg, 0.5 mmol, 2.0 equiv) were dissolved in 1 mL of anhydrous CH_3CN. To this solution was added K_2CO_3 (138.2 mg,

1.0 mmol, 4.0 equiv). After being stirred at 80 °C for 72 h, the reaction mixture was quenched by the addition of water. The aqueous phase was extracted with EtOAc (3 × 20 mL), and the organic phases were washed with saturated aqueous NaCl and then dried over anhydrous Na_2SO_4. After filtration and concentration, the residue was purified through a short pad of silica to remove most impurities. The obtained crude product was dissolved in 5 mL of MeOH, to this solution was added KOH (140.2 mg, 2.5 mmol, 10 equiv). The resulting mixture was stirred at room temperature for 5 h and then concentrated under reduced pressure. To the residue was added water 10 mL, and the aqueous phase was extracted with EtOAc (3 × 20 mL). The combined organic phases were washed with saturated aqueous NaCl and then dried over anhydrous Na_2SO_4. After filtration and concentration, pure **14** was obtained by recrystallization from diethyl ether (44.4 mg, 51% yield in two steps) as a white amorphous solid.

*Synthesis of 3D drug analogue **17*** [4]

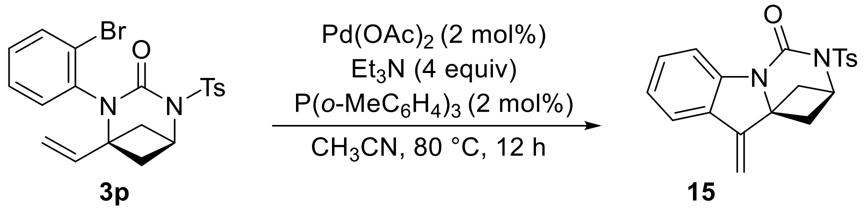

Under a N_2 atmosphere, to a Schlenk tube charged with **3p** (44.7 mg, 0.10 mmol), $Pd(OAc)_2$ (0.5 mg, 0.002 mmol, 2 mol%), and tri(*o*-tolyl)phosphine (0.6 mg, 0.002 mmol, 2 mol%) was successively added 1 mL of CH_3CN and Et_3N (40.5 mg, 0.40 mmol, 4.0 equiv). The reaction mixture was stirred at 80 °C for 12 h. Then, the solvent was removed under reduced pressure, and the residue was purified by flash silica column chromatography employing 17% EtOAc in hexane as eluent to afford **15** (25.7 mg, 70% yield) as a white amorphous solid.

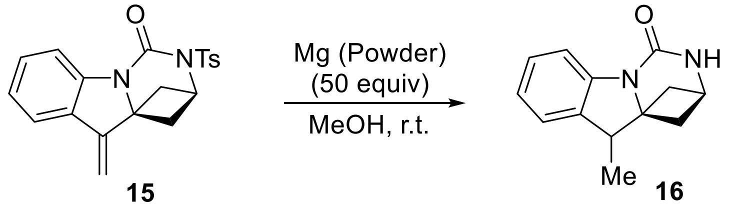

Under an Ar atmosphere, to a Schlenk tube charged with compound **15** (73.3 mg, 0.20 mmol) and Mg powder (240.0 mg, 10.0 mmol, 200-300 mesh) was added anhydrous MeOH (6 mL). The resulting mixture was stirred at room temperature (**Caution**: it releases a lot of heat) until the starting material had been fully consumed as detected by TLC. Then saturated NH_4Cl (4 mL) was added to the reaction mixture to quench any remaining excess of magnesium powder. The aqueous phase was extracted with e EtOAc (3 × 20 mL), and the combined organic phases were dried over Na_2SO_4. After filtration and concentration, the residue was purified by flash silica column chromatography employing 2:1 v/v EtOAc/hexane as eluent to afford **16** (39.8 mg, 93% yield) as a white amorphous solid.

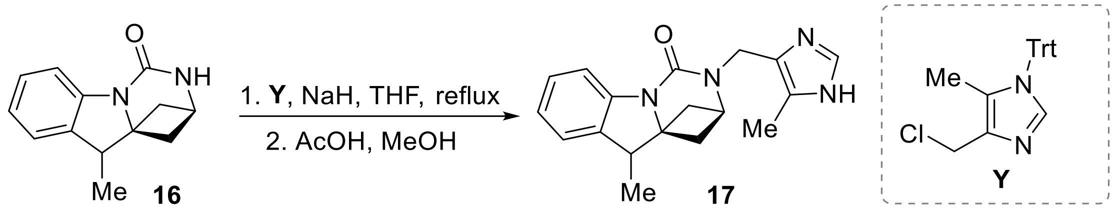

Compound **16** (32.1 mg, 0.15 mmol) was dissolved in 1 mL of anhydrous THF. Under 0 °C, to this solution was added NaH (60 wt% in mineral oil, 12.0 mg, 0.30 mmol, 2.0 equiv). After being stirred at the same temperature for 5 minutes, to the mixture was added **Y** (67.1 mg, 0.18 mmol, 1.2 equiv). The reaction mixture was then heated to 70 °C and stirred for another 16 hours. After cooling down, the reaction was quenched by the addition of water. The aqueous phase was extracted with EtOAc (3 × 20 mL), and the combined organic phases were washed with saturated aqueous NaCl and then dried over anhydrous Na_2SO_4. After filtration and concentration, the residue was dissolved in AcOH/H_2O (4:1, 2 mL), and the mixture was heated to 65 °C for 4 h. After evaporation of the solvent, the residue was neutralized with saturated aqueous $NaHCO_3$ and extracted with DCM (3 × 20 mL). The combined organic phases were dried over anhydrous Na_2SO_4, filtered, and concentrated. The residue was purified by flash silica column chromatography employing 5-10% MeOH in DCM as eluent to afford **17** (22.1 mg, 48% yield in two steps) as a white amorphous solid.

*Synthesis of 3D-analogue of Rupatadine **19***

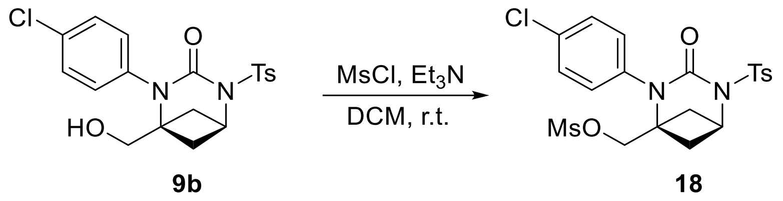

9b (122.1 mg, 0.30 mmol) and MsCl (37.8 mg, 0.33 mmol, 1.1 equiv) were dissolved in 3 mL of anhydrous DCM. To this solution was added Et_3N (60.7 mg, 0.6 mmol, 2.0 equiv), and the mixture was further stirred at room temperature for 2 h. Then the reaction was quenched by the addition of water. The aqueous phase was extracted with DCM (3 × 20 mL), and the combined organic phases were washed with saturated aqueous NaCl and then dried over anhydrous Na_2SO_4. After filtration and concentration, the residue was purified by flash silica column chromatography employing 50% EtOAc in hexane as eluent to afford **18** (137.6 mg, 95% yield) as a white amorphous solid.

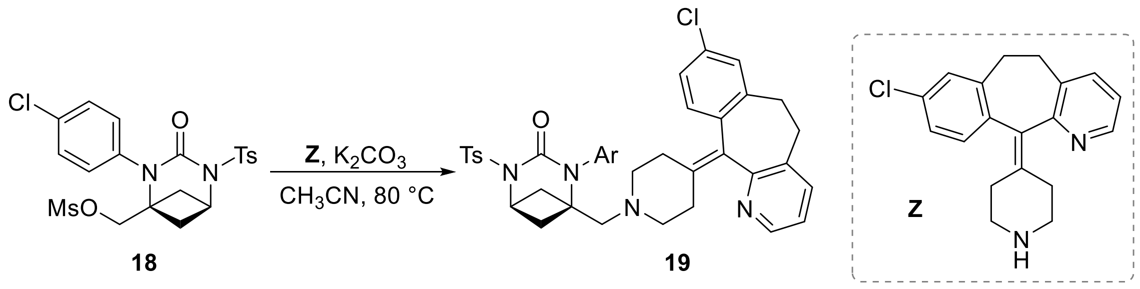

18 (48.5 mg, 0.1 mmol) and **Z** (37.3 mg, 0.12 mmol, 1.2 equiv) were dissolved in 0.5 mL of CH_3CN. To this solution was added K_2CO_3 (27.6 mg, 0.2 mmol, 2.0 equiv), and the resulting mixture was stirred at 80 °C for 72 h, after which it was concentrated under reduced pressure. To the residue was added 5 mL of water, and the aqueous phase was extracted with EtOAc (3 × 20 mL). The combined organic phases were washed with saturated aqueous NaCl and then dried over anhydrous Na_2SO_4. After filtration and concentration, the residue was purified by flash silica column chromatography employing 50% EtOAc in hexane as eluent to afford **19** (36.5 mg, 52% yield) as a white amorphous solid.

3. Full process screening data

Table S1 (Part 1). Screening of process conditions for the Pd-catalyzed transformation of carbamate **1a** and **2a** into 2,4-diazabicyclo[3.1.1]heptan-3-one 3a[a]

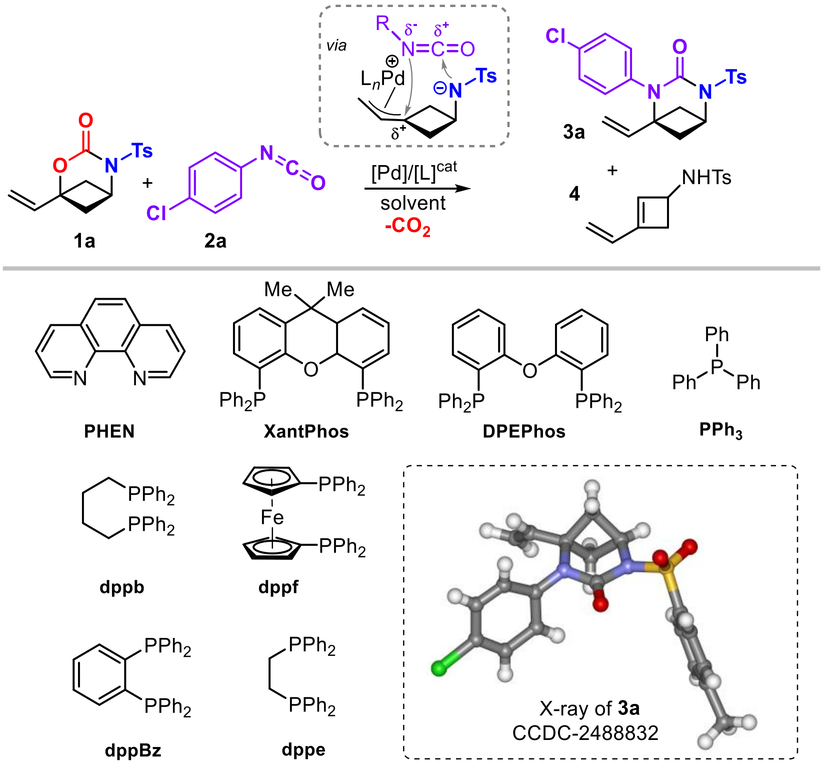

Table S1 (Part 2). Screening of process conditions for the Pd-catalyzed transformation of carbamate **1a** and **2a** into 2,4-diazabicyclo[3.1.1]heptan-3-one 3a[a]

entry	**L**	Sol.	t (min)	Conv. **1a** (%)[b]	**3a**:**4** (%)[b]
1	PHEN	DCM	15	>99	66:17
2	XantPhos	DCM	15	>99	10:10
3	DPEPhos	DCM	15	>99	22:17
4	PPh_3	DCM	15	>99	51:6
**5**	**dppb**	**DCM**	**15**	**>99**	**90**[c]
6	dppf	DCM	15	>99	66:6
7	dppBz	DCM	15	>99	47:10
8	dppe	DCM	15	>99	42:6
9	–	DCM	1440	<5	–
10[d]	dppb	DCM	15	>99	0:26
11[e]	dppb	DCM	1440	<5	–
12	dppb	TOL	15	>99	70:<1
13	dppb	ACN	15	>99	78:<1
14	dppb	DCE	15	>99	84:<1
15	dppb	THF	15	>99	89:<1
16[f]	dppb	DCM	15	>99	88:<1
17[g]	dppb	DCM	60	>99	71:<1
18[h]	–	DCM	1440	<5	–

[a]Unless otherwise stated, all reactions were carried out with **1a** (0.10 mmol), **2a** (0.12 mmol), $Pd_2(dba)_3 \cdot CHCl_3$ (2.5 mol%), and **L** (5 mol%) in 1.0 mL of the indicated solvent under an N_2 atmosphere. [b]Conversion and NMR yields measured by ^1H NMR ($CDCl_3$) using 1,3,5-trimethoxybenzene as internal standard. [c]Yield of the isolated product **3a** was 88% with <1% of **4** formed. [d]Without adding **2a**, a mixture of unidentifiable components was obtained, except for **4**. [e]Without the addition of [Pd]. [f]Using **1a** (0.12 mmol) and **2a** (0.10 mmol). [g]Using $Pd_2(dba)_3 \cdot CHCl_3$ (1.0 mol%) and dppb (2.0 mol%). [h]$Pd(PPh_3)_4$ was used. Sol = solvent.

4. Characterization data for all new compounds

Bicyclic carbamate substrates

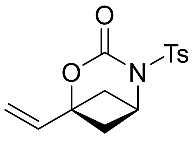

1a

The product was obtained as a white amorphous solid after purification by silica gel column chromatography, eluent: 17% EtOAc in hexane, 472.9 mg, 81% yield from 2 mmol of **S1**. **^{1}H NMR** (300 MHz, $CDCl_3$) δ 7.93 – 7.86 (m, 2H), 7.38 – 7.29 (m, 2H), 5.91 (dd, *J* = 17.4, 10.9 Hz, 1H), 5.36 (dd, *J* = 17.3, 0.9 Hz, 1H), 5.29 (dd, *J* = 10.9, 0.9 Hz, 1H), 5.13 (t, *J* = 4.7 Hz, 1H), 2.57 – 2.46 (m, 2H), 2.43 (s, 3H), 1.95 – 1.84 (m, 2H); **^{13}C NMR** (101 MHz, $CDCl_3$) δ 147.4, 145.4, 135.7, 134.1, 129.8, 128.5, 117.9, 88.3, 52.6, 39.7, 21.8; **HRMS** (ESI/TOF) *m/z* Calcd for $C_{14}H_{16}NO_4S$ $[M + H]^+$ 294.0795; Found 294.0802.

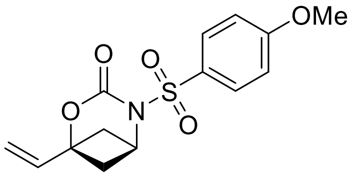

1b

The product was obtained as a colorless oil after purification by silica gel column chromatography, eluent: 33% EtOAc in hexane, 413.9 mg, 67% yield from 2 mmol of **S1**. **^{1}H NMR** (400 MHz, $CDCl_3$) δ 7.98 – 7.92 (m, 2H), 7.01 – 6.95 (m, 2H), 5.91 (dd, *J* = 17.3, 10.9 Hz, 1H), 5.39 – 5.26 (m, 2H), 5.12 (t, *J* = 4.7 Hz, 1H), 3.87 (s, 3H), 2.54 – 2.45 (m, 2H), 1.93 – 1.84 (m, 2H); **^{13}C NMR** (101 MHz, $CDCl_3$) δ 164.2, 147.5, 134.1, 130.9, 130.0, 117.9, 114.3, 88.3, 55.9, 52.5, 39.7; **HRMS** (ESI/TOF) *m/z* Calcd for $C_{14}H_{15}NNaO_5S$ $[M + Na]^+$ 332.0563; Found 332.0570. ^{13}C NMR (101 MHz, $CDCl_3$) δ 164.19, 147.51, 134.11, 130.85, 129.98, 117.87, 114.32, 88.30, 55.86, 52.49, 39.66.

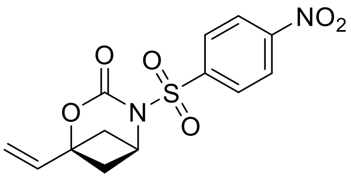

1c

The product was obtained as a white amorphous solid after purification by silica gel column chromatography, eluent: 33% EtOAc in hexane, 421.6 mg, 65% yield from 2 mmol of **S1**. **^{1}H NMR** (400 MHz, $CDCl_3$) δ 8.42 – 8.34 (m, 2H), 8.26 – 8.19 (m, 2H), 5.92 (dd, *J* = 17.4, 10.9 Hz, 1H), 5.43 – 5.28 (m, 2H), 5.16 (t, *J* = 4.7 Hz, 1H), 2.64 – 2.52 (m, 2H), 2.00 – 1.91 (m, 2H); **^{13}C NMR** (101 MHz, $CDCl_3$) δ 150.9, 147.1, 143.9, 133.6, 130.1, 124.3, 118.3, 88.9, 52.9, 39.8; **HRMS** (ESI/TOF) *m/z* Calcd for $C_{13}H_{12}N_2NaO_6S$ $[M + Na]^+$ 347.0308; Found 347.0308.

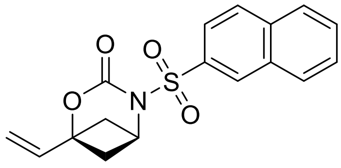

1d

The product was obtained as a white amorphous solid after purification by silica gel column chromatography, eluent: 25% EtOAc in hexane, 462.4 mg, 70% yield from 2 mmol of **S1**. **^{1}H NMR** (500 MHz, $CDCl_3$) δ 8.67 (d, *J* = 1.9 Hz, 1H), 8.04 – 8.00 (m, 1H), 7.97 (d, *J* = 8.7 Hz, 1H), 7.94 – 7.87 (m, 2H), 7.68 (m, 1H), 7.63 (m, 1H), 5.90 (dd, *J* = 17.3, 10.9 Hz, 1H), 5.35 (dd, *J* = 17.3, 0.9 Hz, 1H), 5.28 (dd, *J* = 10.9, 0.9 Hz, 1H), 5.22 (t, *J* = 4.7 Hz, 1H), 2.57 – 2.49 (m, 2H), 1.96 – 1.90 (m, 2H); **^{13}C NMR** (101 MHz, $CDCl_3$) δ 147.4, 135.6, 135.3, 134.0, 132.0, 131.1, 129.9, 129.7, 129.5, 128.0, 127.9, 122.4, 118.0, 88.4, 52.7, 39.8; **HRMS** (ESI/TOF) *m/z* Calcd for $C_{17}H_{15}NNaO_4S$ $[M + Na]^+$ 352.0614; Found 352.0617.

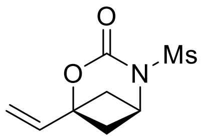

1e

The product was obtained as a white amorphous solid after purification by silica gel column chromatography, eluent: 25% EtOAc in hexane, 383.2 mg, 88% yield from 2 mmol of **S1**. **^{1}H NMR** (500 MHz, $CDCl_3$) δ 5.98 (dd, *J* = 17.4, 10.9 Hz, 1H), 5.43 (dd, *J* = 17.3, 0.8 Hz, 1H), 5.35 (dd, *J* = 11.0, 0.8 Hz, 1H), 4.97 (t, *J* = 4.7 Hz, 1H), 3.41 (s, 3H), 2.59 – 2.51 (m, 2H), 2.05 – 1.97 (m, 2H); **^{13}C NMR** (126 MHz, $CDCl_3$) δ 148.6, 133.9, 118.2, 88.8, 51.6, 42.1, 39.8; **HRMS** (ESI/TOF) *m/z* Calcd for $C_8H_{11}NNaO_4S$ $[M + Na]^+$ 240.0301; Found 240.0297.

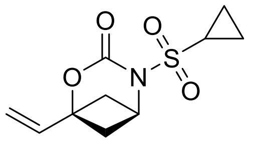

1f

The product was obtained as a white amorphous solid after purification by silica gel column chromatography, eluent: 17% EtOAc in hexane, 405.3 mg, 83% yield from 2 mmol of **S1**. **^{1}H NMR** (400 MHz, $CDCl_3$) δ 5.98 (dd, *J* = 17.4, 10.9 Hz, 1H), 5.43 (d, *J* = 17.3 Hz, 1H), 5.34 (d, *J* = 10.9 Hz, 1H), 4.86 (t, *J* = 4.7 Hz, 1H), 3.26 (m, 1H), 2.57 – 2.46 (m, 2H), 2.05 – 1.95 (m, 2H), 1.39 – 1.31 (m, 2H), 1.17 – 1.09 (m, 2H); **^{13}C NMR** (101 MHz, $CDCl_3$) δ 148.5, 134.1, 118.0, 88.5, 52.2, 39.7, 31.7, 6.4; **HRMS** (ESI/TOF) *m/z* Calcd for $C_{10}H_{13}NNaO_4S$ $[M + Na]^+$ 266.0457; Found 266.0454.

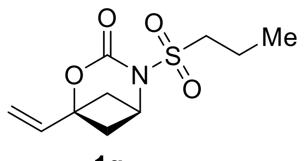

1g

The product was obtained as a white amorphous solid after purification by silica gel column chromatography, eluent: 33% EtOAc in hexane, 373.8 mg, 76% yield from 2 mmol of **S1**. **^{1}H NMR** (400 MHz, $CDCl_3$) δ 5.98 (dd, *J* = 17.4, 10.9 Hz, 1H), 5.47 – 5.32 (m, 2H), 4.92 (t, *J* = 4.7 Hz, 1H), 3.62 – 3.54 (m, 2H), 2.59 – 2.48 (m, 2H), 2.05 – 1.96 (m, 2H), 1.88 – 1.77 (m, 2H), 1.08 (t, *J* = 7.5 Hz, 3H); **^{13}C NMR** (101 MHz, $CDCl_3$) δ 148.5, 134.0, 118.1, 88.7, 55.4, 51.6, 39.8, 17.2, 12.8; **HRMS** (ESI/TOF) *m/z* Calcd for $C_{10}H_{15}NNaO_4S$ $[M + Na]^+$ 268.0614; Found 268.0613.

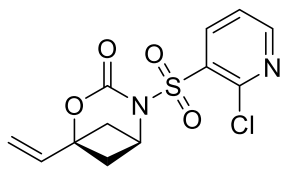

1h

The product was obtained as a white amorphous solid after purification by silica gel column chromatography, eluent: 25% EtOAc in hexane, 372.0 mg, 59% yield from 2 mmol of **S1**. **^{1}H NMR** (400 MHz, $CDCl_3$) δ 8.65 – 8.59 (m, 2H), 7.52 – 7.48 (m, 1H), 5.96 (dd, *J* = 17.4, 10.9 Hz, 1H), 5.37 (dd, *J* = 27.7, 14.1 Hz, 2H), 5.22 (t, *J* = 4.8 Hz, 1H), 2.69 – 2.55 (m, 2H), 2.20 – 2.15 (m, 2H); **^{13}C NMR** (101 MHz, $CDCl_3$) δ 153.7, 147.9, 147.1, 143.3, 133.8, 133.7, 122.9, 118.3, 88.8, 53.2, 39.8; **HRMS** (ESI/TOF) *m/z* Calcd for $C_{12}H_{11}ClN_2NaO_4S$ $[M + Na]^+$ 337.0020; Found 337.0028.

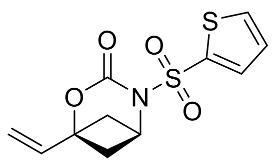

1i

The product was obtained as a colorless oil after purification by silica gel column chromatography, eluent: 25% EtOAc in hexane, 401.1 mg, 70% yield from 2 mmol of **S1**. **^{1}H NMR** (400 MHz, $CDCl_3$) δ 7.92 (dd, *J* = 3.8, 1.4 Hz, 1H), 7.69 (dd, *J* = 5.0, 1.4 Hz, 1H), 7.13 (dd, *J* = 5.0, 3.8 Hz, 1H), 5.92 (dd, *J* = 17.4, 10.9 Hz, 1H), 5.42 – 5.27 (m, 2H), 5.11 (t, *J* = 4.7 Hz, 1H), 2.57 – 2.47 (m, 2H), 1.97 – 1.89 (m, 2H); **^{13}C NMR** (101 MHz, $CDCl_3$) δ 147.2, 138.2, 135.9, 134.2, 134.0, 127.7, 118.1, 88.5, 53.0, 39.7; **HRMS** (ESI/TOF) *m/z* Calcd for $C_{11}H_{11}NNaO_4S_2$ $[M + Na]^+$ 308.0022; Found 308.0030.

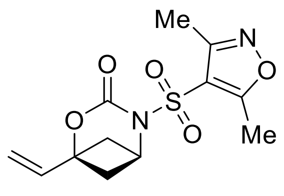

1j

The product was obtained as a light-yellow oil after purification by silica gel column chromatography, eluent: 25% EtOAc in hexane, 331.7 mg, 56% yield from 2 mmol of **S1**. **^{1}H NMR** (500 MHz, $CDCl_3$) δ 5.95 (dd, *J* = 17.3, 10.9 Hz, 1H), 5.43 – 5.32 (m, 2H), 5.13 (t, *J* = 4.7 Hz, 1H), 2.74 (s, 3H), 2.62 – 2.54 (m, 2H), 2.36 (s, 3H), 2.03 – 1.96 (m, 2H); **^{13}C NMR** (101 MHz, $CDCl_3$) δ 177.3, 157.4, 147.5, 133.7, 118.3, 114.7, 88.6, 52.4, 39.9, 13.3, 10.9; **HRMS** (ESI/TOF) *m/z* Calcd for $C_{12}H_{14}N_2NaO_5S$ $[M + Na]^+$ 321.0516; Found 321.0503.

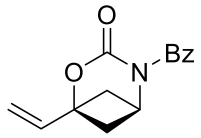

1k

The product was obtained as a white amorphous solid after purification by silica gel column chromatography, eluent: 25% EtOAc in hexane, 413.5 mg, 85% yield from 2 mmol of **S1**. **^{1}H NMR** (400 MHz, $CDCl_3$) δ 7.67 – 7.61 (m, 2H), 7.56 – 7.49 (m, 1H), 7.46 – 7.39 (m, 2H), 6.03 (dd, *J* = 17.4, 10.9 Hz, 1H), 5.45 (dd, *J* = 17.4, 0.9 Hz, 1H), 5.35 (dd, *J* = 10.9, 0.9 Hz, 1H), 5.16 (t, *J* = 4.7 Hz, 1H), 2.63 – 2.52 (m, 2H), 2.12 – 2.03 (m, 2H); **^{13}C NMR** (101 MHz, $CDCl_3$) δ 171.7, 149.0, 135.0, 134.5, 132.2, 128.8, 128.1, 117.8, 87.8, 50.1, 39.4; **HRMS** (ESI/TOF) *m/z* Calcd for $C_{14}H_{13}NNaO_3$ $[M + Na]^+$ 266.0788; Found 266.0792.

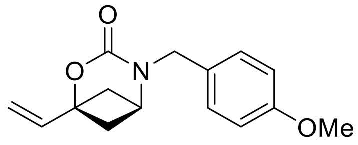

1l

The product was obtained as a white amorphous solid after purification by silica gel column chromatography, eluent: 25% EtOAc in hexane, 391.6 mg, 76% yield from 2 mmol of **S1**. **^{1}H NMR** (500 MHz, $CDCl_3$) δ 7.25 – 7.21 (m, 2H), 6.87 – 6.84 (m, 2H), 5.96 (dd, *J* = 17.4, 10.9 Hz, 1H), 5.38 (dd, *J* = 17.3, 1.1 Hz, 1H), 5.25 (dd, *J* = 10.9, 1.1 Hz, 1H), 4.54 (s, 2H), 3.79 (s, 3H), 3.72 (t, *J* = 4.4 Hz, 1H), 2.27 – 2.20 (m, 2H), 1.79 – 1.73 (m, 2H); **^{13}C NMR** (126 MHz, $CDCl_3$) δ 159.3, 153.2, 135.7, 129.6, 128.9, 116.7, 114.2, 86.5, 55.4, 51.1, 51.0, 39.2; **HRMS** (ESI/TOF) *m/z* Calcd for $C_{15}H_{18}NO_3$ $[M + H]^+$ 260.1281; Found 260.1285.

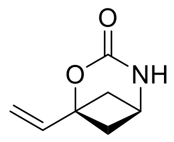

S1

The product was obtained as a light-brown amorphous solid after purification by silica gel column chromatography, eluent: 100% EtOAc in hexane, 482.9 mg, 35% yield from 10 mmol of tert-butyl (3-oxocyclobutyl)carbamate in 2 steps. **^{1}H NMR** (500 MHz, $CDCl_3$) δ 7.13 (s, 1H), 5.98 (dd, *J* = 17.4, 10.9 Hz, 1H), 5.40 (dd, *J* = 17.4, 1.1 Hz, 1H), 5.26 (dd, *J* = 10.9, 1.1 Hz, 1H), 3.88 (m, 1H), 2.36 – 2.28 (m, 2H), 1.92 – 1.84 (m, 2H); **^{13}C NMR** (126 MHz, $CDCl_3$) δ 154.0, 135.7, 116.6, 87.0, 46.9, 39.4; **HRMS** (ESI/TOF) *m/z* Calcd for $C_7H_9NNaO_2$ [M + Na]$^+$ 162.0525; Found 162.0526.

2,4-Diazabicyclo[3.1.1]heptan-3-one products

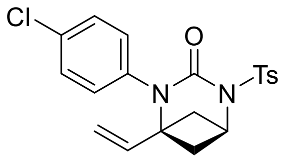

3a

The product was obtained as a white amorphous solid after purification by silica gel column chromatography, eluent: 17% EtOAc in hexane, 35.6 mg, 88% yield. **^{1}H NMR** (400 MHz, $CDCl_3$) δ 7.95 – 7.82 (m, 2H), 7.29 – 7.27 (m, , 2H), 7.26 – 7.21 (m, 2H), 6.96 – 6.90 (m, 2H), 5.47 (dd, *J* = 17.2, 10.6 Hz, 1H), 5.25 (t, *J* = 4.9 Hz, 1H), 5.12 (dd, *J* = 17.2, 1.0 Hz, 1H), 5.07 (dd, *J* = 10.5, 1.0 Hz, 1H), 2.60 – 2.53 (m, 2H), 2.41 (s, 3H), 2.10 – 2.02 (m, 2H); **^{13}C NMR** (126 MHz, $CDCl_3$) δ 150.1, 144.5, 137.0, 136.8, 135.5, 133.7, 131.2, 129.6, 129.1, 128.4, 117.4, 68.3, 52.4, 39.7, 21.8; **HRMS** (ESI/TOF) *m/z* Calcd for $C_{20}H_{19}ClN_2NaO_3S$ $[M + Na]^+$ 425.0697; Found 425.0690.

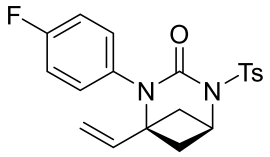

3b

The product was obtained as a white amorphous solid after purification by silica gel column chromatography, eluent: 17% EtOAc in hexane, 23.4 mg, 61% yield. **^{1}H NMR** (400 MHz, $CDCl_3$) δ 7.92 – 7.86 (m, 2H), 7.31 – 7.26 (m, 2H), 6.98 – 6.92 (m, 4H), 5.46 (dd, *J* = 17.2, 10.5 Hz, 1H), 5.26 (t, *J* = 4.9 Hz, 1H), 5.11 (dd, *J* = 17.2, 1.0 Hz, 1H), 5.05 (dd, *J* = 10.6, 1.0 Hz, 1H), 2.60 – 2.52 (m, 2H), 2.41 (s, 3H), 2.10 – 2.02 (m, 2H); **^{13}C NMR** (101 MHz, $CDCl_3$) δ 161.9 (d, *J* = 247.6 Hz), 150.3, 144.5, 137.0, 135.6, 134.2 (d, *J* = 3.3 Hz), 131.5 (d, *J* = 8.7 Hz), 129.6, 128.4, 117.3, 115.9 (d, *J* = 22.7 Hz), 68.3, 52.4, 39.7, 21.8; **^{19}F NMR** (376 MHz, $CDCl_3$) δ -113.72; **HRMS** (ESI/TOF) *m/z* Calcd for $C_{20}H_{20}FN_2O_3S$ $[M + H]^+$ 387.1173; Found 387.1167.

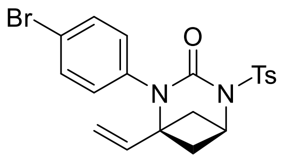

3c

The product was obtained as a white amorphous solid after purification by silica gel column chromatography, eluent: 14-17% EtOAc in hexane, 32.7 mg, 74% yield. **^{1}H NMR** (400 MHz, $CDCl_3$) δ 7.93 – 7.84 (m, 2H), 7.44 – 7.36 (m, 2H), 7.30 – 7.26 (m, 2H), 6.90 – 6.83 (m, 2H), 5.47 (dd, *J* = 17.2, 10.5 Hz, 1H), 5.25 (t, *J* = 4.9 Hz, 1H), 5.12 (dd, *J* = 17.2, 0.9 Hz, 1H), 5.07 (dd, *J* = 10.5, 0.9 Hz, 1H), 2.60 – 2.53 (m, 2H), 2.41 (s, 3H), 2.10 – 2.02 (m, 2H); **^{13}C NMR** (126 MHz, $CDCl_3$) δ 150.0, 144.5, 137.3, 137.0, 135.5, 132.1, 131.5, 129.6, 128.4, 121.8, 117.4, 68.3, 52.4, 39.7, 21.8; **HRMS** (ESI/TOF) *m/z* Calcd for $C_{20}H_{20}BrN_2O_3S$ $[M + H]^+$ 447.0373; Found 447.0368.

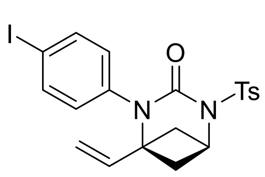

3d

The product was obtained as a white amorphous solid after purification by silica gel column chromatography, eluent: 14-17% EtOAc in hexane, 41.1 mg, 83% yield. **^{1}H NMR** (500 MHz, $CDCl_3$) δ 7.91 – 7.85 (m, 2H), 7.62 – 7.57 (m, 2H), 7.29 – 7.26 (m, 2H), 6.76 – 6.71 (m, 2H), 5.47 (dd, J = 17.2, 10.6 Hz, 1H), 5.25 (t, J = 4.9 Hz, 1H), 5.12 (dd, J = 17.2, 1.0 Hz, 1H), 5.07 (dd, J = 10.6, 1.0 Hz, 1H), 2.59 – 2.53 (m, 2H), 2.40 (s, 3H), 2.08 – 2.02 (m, 2H); **^{13}C NMR** (126 MHz, $CDCl_3$) δ 150.0, 144.5, 138.1, 138.0, 137.0, 135.5, 131.8, 129.5, 128.4, 117.4, 93.4, 68.3, 52.3, 39.7, 21.8; **HRMS** (ESI/TOF) *m/z* Calcd for $C_{20}H_{20}IN_2O_3S$ $[M + H]^+$ 495.0234; Found 495.0232.

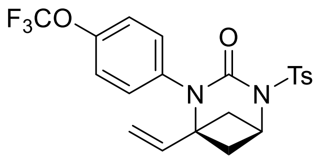

3e

The product was obtained as a white amorphous solid after purification by silica gel column chromatography, eluent: 17% EtOAc in hexane, 35.8 mg, 79% yield. **^{1}H NMR** (500 MHz, $CDCl_3$) δ 7.96 – 7.80 (m, 2H), 7.31 – 7.27 (m, 2H), 7.14 – 7.09 (m, 2H), 7.05 – 7.00 (m, 2H), 5.46 (dd, J = 17.2, 10.5 Hz, 1H), 5.27 (t, J = 4.9 Hz, 1H), 5.13 (dd, J = 17.2, 0.9 Hz, 1H), 5.08 (dd, J = 10.6, 0.9 Hz, 1H), 2.60 – 2.55 (m, 2H), 2.41 (s, 3H), 2.11 – 2.04 (m, 2H); **^{13}C NMR** (126 MHz, $CDCl_3$) δ 150.13, 148.39, 148.37, 148.36, 148.34, 144.54, 137.00, 136.68, 135.48, 131.33, 129.58, 128.38, 121.27, 120.43 (q, J = 257.04 Hz), 117.49, 68.40, 52.35, 39.70, 21.77; **^{19}F NMR** (376 MHz, $CDCl_3$) δ -58.00; **HRMS** (ESI/TOF) *m/z* Calcd for $C_{21}H_{20}F_3N_2O_4S$ $[M + H]^+$ 453.1090; Found 453.1092.

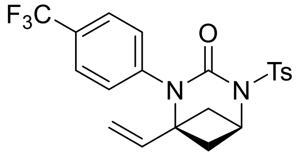

3f

The product was obtained as a white amorphous solid after purification by silica gel column chromatography, eluent: 12.5-17% EtOAc in hexane, 40.2 mg, 92% yield. **^{1}H NMR** (400 MHz, $CDCl_3$) δ 7.94 – 7.86 (m, 2H), 7.55 – 7.53 (m, 2H), 7.30 – 7.28 (m, 2H), 7.14 – 7.12 (m, 2H), 5.45 (dd, J = 17.2, 10.5 Hz, 1H), 5.28 (t, J = 4.9 Hz, 1H), 5.16 (dd, J = 17.2, 0.9 Hz, 1H), 5.09 (dd, J = 10.5, 0.9 Hz, 1H), 2.64 – 2.56 (m, 2H), 2.41 (s, 3H), 2.14 – 2.06 (m, 2H); **^{13}C NMR** (101 MHz, $CDCl_3$) δ 150.0, 144.6, 141.4, 136.9, 135.4, 130.3, 129.6, 128.4, 126.0 (q, J = 3.7 Hz), 123.8 (q, J = 272.3 Hz) 117.7, 68.5, 52.4, 39.8, 21.7; **^{19}F NMR** (376 MHz, $CDCl_3$) δ -62.72; **HRMS** (ESI/TOF) *m/z* Calcd for $C_{21}H_{20}F_3N_2O_3S$ $[M + H]^+$ 437.1141; Found 437.1138.

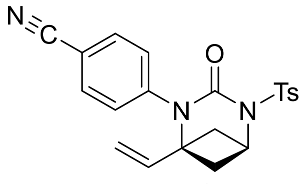

3g

The product was obtained as a white amorphous solid after purification by silica gel column chromatography, eluent: 25-33% EtOAc in hexane, 37.7 mg, 96% yield. **^{1}H NMR** (400 MHz, $CDCl_3$) δ 7.93 – 7.83 (m, 2H), 7.61 – 7.51 (m, 2H), 7.32 – 7.26 (m, 2H), 7.15 – 7.08 (m, 2H), 5.44 (dd, *J* = 17.2, 10.5 Hz, 1H), 5.27 (t, *J* = 4.9 Hz, 1H), 5.17 (dd, *J* = 17.2, 0.9 Hz, 1H), 5.11 (dd, *J* = 10.5, 0.9 Hz, 1H), 2.67 – 2.59 (m, 2H), 2.41 (s, 3H), 2.12 – 2.04 (m, 2H); **^{13}C NMR** (101 MHz, $CDCl_3$) δ 149.7, 144.7, 142.4, 136.8, 135.2, 132.7, 130.8, 129.6, 128.3, 118.3, 118.0, 111.5, 68.6, 52.4, 39.7, 21.7; **HRMS** (ESI/TOF) *m/z* Calcd for $C_{21}H_{20}N_3O_3S$ $[M + H]^+$ 394.1220; Found 394.1207.

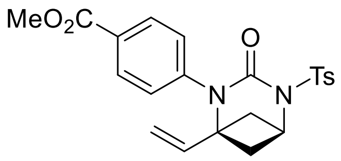

3h

The product was obtained as a white amorphous solid after purification by silica gel column chromatography, eluent: 33% EtOAc in hexane, 41.2 mg, 97% yield. **^{1}H NMR** (400 MHz, $CDCl_3$) δ 7.98 – 7.91 (m, 2H), 7.91 – 7.83 (m, 2H), 7.31 – 7.26 (m, 2H), 7.11 – 7.03 (m, 2H), 5.45 (dd, *J* = 17.2, 10.5 Hz, 1H), 5.26 (t, *J* = 4.9 Hz, 1H), 5.13 (dd, *J* = 17.2, 0.9 Hz, 1H), 5.05 (dd, *J* = 10.6, 0.9 Hz, 1H), 3.87 (s, 3H), 2.62 – 2.54 (m, 2H), 2.40 (s, 3H), 2.14 – 2.04 (m, 2H); **^{13}C NMR** (126 MHz, $CDCl_3$) δ 166.4, 149.8, 144.5, 142.4, 136.9, 135.5, 130.2, 129.9, 129.6, 129.4, 128.4, 117.5, 68.5, 52.4, 52.3, 39.7, 21.7; **HRMS** (ESI/TOF) *m/z* Calcd for $C_{22}H_{23}N_2O_5S$ $[M + H]^+$ 427.1322; Found 427.1328.

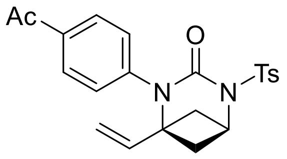

3i

The product was obtained as a colorless oil after purification by silica gel column chromatography, eluent: 50% EtOAc in hexane, 35.6 mg, 87% yield. **^{1}H NMR** (500 MHz, $CDCl_3$) δ 7.91 – 7.83 (m, 4H), 7.30 – 7.26 (m, 2H), 7.11 – 7.08 (m, 2H), 5.46 (dd, *J* = 17.2, 10.6 Hz, 1H), 5.27 (t, *J* = 4.9 Hz, 1H), 5.15 (dd, *J* = 17.2, 0.9 Hz, 1H), 5.07 (dd, *J* = 10.5, 0.9 Hz, 1H), 2.62 – 2.54 (m, 2H), 2.54 (s, 3H), 2.40 (s, 3H), 2.13 – 2.07 (m, 2H); **^{13}C NMR** (101 MHz, $CDCl_3$) δ 197.3, 149.9, 144.6, 142.6, 136.9, 136.2, 135.5, 130.0, 129.6, 128.9, 128.4, 117.6, 68.5, 52.4, 39.8, 26.7, 21.8; **HRMS** (ESI/TOF) *m/z* Calcd for $C_{22}H_{23}N_2O_4S$ $[M + H]^+$ 411.1373; Found 411.1375.

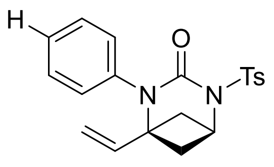

3j

The product was obtained as a colorless oil after purification by silica gel column chromatography, eluent: 17-25% EtOAc in hexane, 16.8 mg, 46% yield. **^{1}H NMR** (400 MHz, $CDCl_3$) δ 7.93 – 7.88 (m, 2H), 7.33 – 7.27 (m, 3H), 7.26 – 7.20 (m, 2H), 7.02 – 6.96 (m, 2H), 5.48 (dd, *J* = 17.2, 10.6 Hz, 1H), 5.26 (t, *J* = 4.9 Hz, 1H), 5.10 (dd, *J* = 17.2, 1.0 Hz, 1H), 5.02 (dd, *J* = 10.5, 1.0 Hz, 1H), 2.60 – 2.52 (m, 2H), 2.41 (s, 3H), 2.14 – 2.05 (m, 2H); **^{13}C NMR** (101 MHz, $CDCl_3$) δ 150.2, 144.4, 138.3, 137.2, 135.8, 129.9, 129.5, 129.0, 128.4, 127.9, 116.9, 68.3, 52.4, 39.8, 21.8; **HRMS** (ESI/TOF) *m/z* Calcd for $C_{20}H_{21}N_2O_3S$ $[M + H]^+$ 369.1267; Found 369.1265.

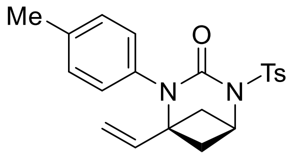

3k

The product was obtained as a colorless oil after purification by silica gel column chromatography, eluent: 17-25% EtOAc in hexane, 15.0 mg, 39% yield. **^{1}H NMR** (400 MHz, $CDCl_3$) δ 7.93 – 7.86 (m, 2H), 7.29 – 7.25 (m, 2H), 7.11 – 7.04 (m, 2H), 6.89 – 6.83 (m, 2H), 5.50 (dd, *J* = 17.2, 10.6 Hz, 1H), 5.25 (t, *J* = 4.9 Hz, 1H), 5.09 (dd, *J* = 17.2, 1.0 Hz, 1H), 5.02 (dd, *J* = 10.6, 1.0 Hz, 1H), 2.58 – 2.51 (m, 2H), 2.41 (s, 3H), 2.28 (s, 3H), 2.11 – 2.03 (m, 2H); **^{13}C NMR** (101 MHz, $CDCl_3$) δ 150.2, 144.3, 137.8, 137.2, 135.9, 135.7, 129.6, 129.54, 129.48, 128.5, 116.7, 68.2, 52.3, 39.8, 21.8, 21.2; **HRMS** (ESI/TOF) *m/z* Calcd for $C_{21}H_{23}N_2O_3S$ $[M + H]^+$ 383.1424; Found 383.1417.

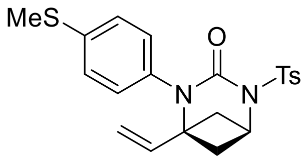

3l

The product was obtained as a light-yellow amorphous solid after purification by silica gel column chromatography, eluent: 25-33% EtOAc in hexane, 23.0 mg, 55% yield. **^{1}H NMR** (400 MHz, $CDCl_3$) δ 7.92 – 7.85 (m, 2H), 7.28 – 7.26 (m, 2H), 7.15 – 7.10 (m, 2H), 6.95 – 6.83 (m, 2H), 5.49 (dd, *J* = 17.2, 10.6 Hz, 1H), 5.25 (t, *J* = 4.9 Hz, 1H), 5.10 (dd, *J* = 17.2, 1.0 Hz, 1H), 5.04 (dd, *J* = 10.6, 1.0 Hz, 1H), 2.59 – 2.52 (m, 2H), 2.42 (s, 3H), 2.40 (s, 3H), 2.10 – 2.02 (m, 2H); **^{13}C NMR** (101 MHz, $CDCl_3$) δ 150.3, 144.4, 138.5, 137.1, 135.7, 135.2, 130.1, 129.5, 128.4, 126.8, 117.0, 68.3, 52.4, 39.7, 21.8, 15.8; **HRMS** (ESI/TOF) *m/z* Calcd for $C_{21}H_{22}N_2NaO_3S_2$ $[M + Na]^+$ 437.0964; Found 437.0958.

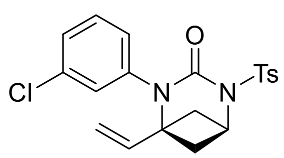

3m

The product was obtained as a colorless oil after purification by silica gel column chromatography, eluent: 17-25% EtOAc in hexane, 36.7 mg, 93% yield. **^{1}H NMR** (300 MHz, $CDCl_3$) δ 8.02 – 7.78 (m, 2H), 7.33 – 7.26 (m, 2H), 7.24 – 7.17 (m, 2H), 7.06 – 6.96 (m, 1H), 6.94 – 6.87 (m, 2H), 5.48 (dd, *J* = 17.2, 10.5 Hz, 1H), 5.26 (t, *J* = 4.9 Hz, 1H), 5.18 – 5.05 (m, 2H), 2.62 – 2.52 (m, 2H), 2.41 (s, 3H), 2.12 – 2.00 (m, 2H); **^{13}C NMR** (101 MHz, $CDCl_3$) δ 150.0, 144.5, 139.3, 137.0, 135.4, 134.3, 130.2, 129.8, 129.6, 128.4, 128.23, 128.21, 117.5, 68.4, 52.4, 39.7, 21.8; **HRMS** (ESI/TOF) *m/z* Calcd for $C_{20}H_{20}ClN_2O_3S$ $[M + H]^+$ 403.0878; Found 403.0882.

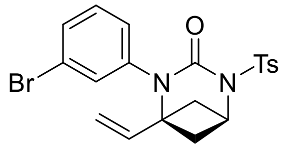

3n

The product was obtained as a white amorphous solid after purification by silica gel column chromatography, eluent: 17-25% EtOAc in hexane, 42.2 mg, 94% yield. **^{1}H NMR** (500 MHz, $CDCl_3$) δ 7.91 – 7.86 (m, 2H), 7.37 (m, 1H), 7.30 – 7.27 (m, 2H), 7.17 – 7.11 (m, 2H), 6.95 (m, 1H), 5.47 (dd, *J* = 17.2, 10.6 Hz, 1H), 5.25 (t, *J* = 4.9 Hz, 1H), 5.13 (dd, *J* = 17.2, 0.9 Hz, 1H), 5.08 (dd, *J* = 10.6, 0.9 Hz, 1H), 2.60 – 2.54 (m, 2H), 2.41 (s, 3H), 2.10 – 2.04 (m, 2H); **^{13}C NMR** (126 MHz, $CDCl_3$) δ 149.89, 144.45, 139.35, 136.85, 135.30, 132.91, 131.00, 129.97, 129.49, 128.62, 128.27, 122.06, 117.45, 68.35, 52.26, 39.63, 21.68; **HRMS** (ESI/TOF) *m/z* Calcd for $C_{20}H_{20}BrN_2O_3S$ $[M + H]^+$ 447.0373; Found 447.0370.

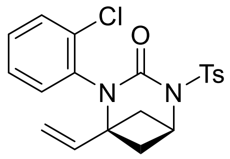

3o

The product was obtained as a white amorphous solid after purification by silica gel column chromatography, eluent: 17-25% EtOAc in hexane, 30.3 mg, 75% yield. **^{1}H NMR** (500 MHz, $CDCl_3$) δ 7.93 – 7.86 (m, 2H), 7.36 – 7.33 (m, 1H), 7.30 – 7.26 (m, 2H), 7.23 – 7.16 (m, 2H), 7.09 – 7.04 (m, 1H), 5.47 (dd, *J* = 17.1, 10.5 Hz, 1H), 5.26 (t, *J* = 4.9 Hz, 1H), 5.14 (dd, *J* = 17.1, 1.1 Hz, 1H), 5.01 (dd, *J* = 10.5, 1.1 Hz, 1H), 2.60 (m, 1H), 2.49 (m, 1H), 2.41 (s, 3H), 2.31 (dd, *J* = 10.5, 9.5 Hz, 1H), 2.11 (dd, *J* = 10.4, 9.6 Hz, 1H); **^{13}C NMR** (126 MHz, $CDCl_3$) δ 149.2, 144.4, 138.0, 136.1, 134.6, 134.3, 132.4, 130.0, 129.7, 129.5, 128.4, 127.5, 118.0, 68.3, 52.8, 39.9, 39.2, 21.8; **HRMS** (ESI/TOF) *m/z* Calcd for $C_{20}H_{20}ClN_2O_3S$ $[M + H]^+$ 403.0878; Found 403.0892.

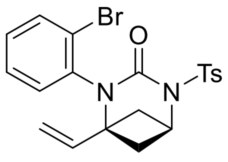

3p

The product was obtained as a white amorphous solid after purification by silica gel column chromatography, eluent: 25-33% EtOAc in hexane, 29.4 mg, 66% yield (321.5 mg, 72% yield on 1.0 mmol scale). **^{1}H NMR** (400 MHz, $CDCl_3$) δ 7.94 – 7.87 (m, 2H), 7.52 (dd, *J* = 8.0, 1.5 Hz, 1H), 7.30 – 7.26 (m, 2H), 7.25 – 7.21 (m, 1H), 7.16 – 7.10 (m, 1H), 7.05 (dd, *J* = 7.8, 1.7 Hz, 1H), 5.47 (dd, *J* = 17.1, 10.5 Hz, 1H), 5.26 (t, *J* = 4.8 Hz, 1H), 5.14 (dd, *J* = 17.1, 1.1 Hz, 1H), 5.01 (dd, *J* = 10.5, 1.0 Hz, 1H), 2.66 – 2.59 (m, 1H), 2.48 (dd, *J* = 10.5, 4.7 Hz, 1H), 2.43 – 2.38 (m, 4H), 2.13 – 2.07 (m, 1H); **^{13}C NMR** (126 MHz, $CDCl_3$) δ 149.0, 144.4, 137.8, 136.9, 134.4, 133.1, 132.3, 129.8, 129.4, 128.4, 128.1, 125.4, 118.0, 68.3, 52.7, 39.7, 39.4, 21.7; **HRMS** (ESI/TOF) *m/z* Calcd for $C_{20}H_{20}BrN_2O_3S$ $[M + H]^+$ 447.0373; Found 447.0373.

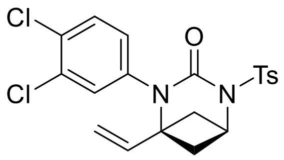

3q

The product was obtained as a white amorphous solid after purification by silica gel column chromatography, eluent: 12.5-17% EtOAc in hexane, 38.6 mg, 88% yield. ^{1}H NMR (500 MHz, $CDCl_3$) δ 7.92 – 7.86 (m, 2H), 7.34 (d, *J* = 8.5 Hz, 1H), 7.31 – 7.27 (m, 2H), 7.11 (d, *J* = 2.4 Hz, 1H), 6.87 (dd, *J* = 8.5, 2.4 Hz, 1H), 5.48 (dd, *J* = 17.2, 10.6 Hz, 1H), 5.26 (t, *J* = 4.9 Hz, 1H), 5.18 – 5.09 (m, 2H), 2.61 – 2.54 (m, 2H), 2.41 (s, 3H), 2.09 – 2.03 (m, 2H); **^{13}C NMR** (126 MHz, $CDCl_3$) δ 150.0, 144.7, 137.5, 136.8, 135.2, 132.7, 132.2, 131.9, 130.5, 129.6, 129.4, 128.4, 117.9, 68.5, 52.4, 39.7, 21.8; **HRMS** (ESI/TOF) *m/z* Calcd for $C_{20}H_{19}Cl_2N_2O_3S$ $[M + H]^+$ 437.0488; Found 437.0486.

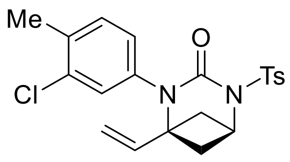

3r

The product was obtained as a colorless oil after purification by silica gel column chromatography, eluent: 17-25% EtOAc in hexane, 30.3 mg, 73% yield. **^{1}H NMR** (400 MHz, $CDCl_3$) δ 7.93 – 7.86 (m, 2H), 7.31 – 7.26 (m, 2H), 7.12 (dd, *J* = 8.1, 0.8 Hz, 1H), 6.99 (d, *J* = 2.1 Hz, 1H), 6.81 (dd, *J* = 8.0, 2.1 Hz, 1H), 5.49 (dd, *J* = 17.2, 10.6 Hz, 1H), 5.25 (t, *J* = 4.9 Hz, 1H), 5.15 – 5.05 (m, 2H), 2.60 – 2.52 (m, 2H), 2.41 (s, 3H), 2.29 (s, 3H), 2.10 – 2.02 (m, 2H); **^{13}C NMR** (101 MHz, $CDCl_3$) δ 150.1, 144.5, 137.0, 136.8, 136.1, 135.5, 134.3, 131.0, 130.3, 129.6, 128.4, 128.1, 117.4, 68.3, 52.4, 39.7, 21.8, 19.8; **HRMS** (ESI/TOF) *m/z* Calcd for $C_{21}H_{22}ClN_2O_3S$ $[M + H]^+$ 417.1034; Found 417.1039.

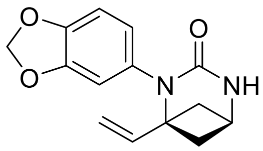

3s

The product was obtained as a white amorphous solid after purification by silica gel column chromatography, eluent: 200% EtOAc in hexane, then EtOAc, 9.3 mg, 36% yield in two steps. **^{1}H NMR** (500 MHz, $CDCl_3$) δ 6.75 – 6.73 (m, 1H), 6.62 – 6.59 (m, 2H), 5.97 (s, 1H), 5.96 (s, 2H), 5.65 (dd, J = 17.2, 10.6 Hz, 1H), 5.10 (dd, J = 17.2, 1.2 Hz, 1H), 5.03 (dd, J = 10.6, 1.2 Hz, 1H), 3.93 (q, J = 4.7 Hz, 1H), 2.37 – 2.31 (m, 2H), 2.02 – 1.95 (m, 2H); **^{13}C NMR** (101 MHz, $CDCl_3$) δ 155.7, 147.8, 146.9, 137.1, 133.7, 123.6, 115.9, 111.1, 108.0, 101.6, 68.1, 47.3, 39.2; **HRMS** (ESI/TOF) *m/z* Calcd for $C_{14}H_{15}N_2O_3$ [M + H]$^+$ 259.1077; Found 259.1071.

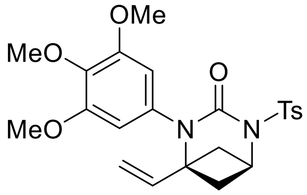

3t

The product was obtained as a colorless oil after purification by silica gel column chromatography, eluent: 50% EtOAc in hexane, 19.2 mg, 42%. **^{1}H NMR** (500 MHz, $CDCl_3$) δ 7.94 – 7.89 (m, 2H), 7.31 – 7.27 (m, 2H), 6.20 (s, 2H), 5.55 (dd, J = 17.2, 10.6 Hz, 1H), 5.26 (t, J = 4.9 Hz, 1H), 5.14 – 5.04 (m, 2H), 3.78 (s, 3H), 3.75 (s, 6H), 2.59 – 2.54 (m, 2H), 2.41 (s, 3H), 2.10 – 2.03 (m, 2H); **^{13}C NMR** (126 MHz, $CDCl_3$) δ 153.3, 150.1, 144.4, 137.8, 137.2, 135.6, 133.8, 129.5, 128.5, 116.8, 107.3, 68.4, 60.9, 56.2, 52.3, 39.8, 21.8; **HRMS** (ESI/TOF) *m/z* Calcd for $C_{23}H_{26}N_2NaO_6S$ [M + Na]$^+$ 481.1404; Found 481.1388.

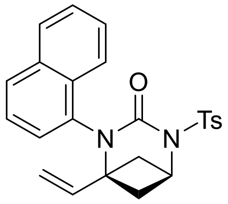

3u

The product was obtained as a colorless oil after purification by silica gel column chromatography, eluent: 17-25% EtOAc in hexane, 25.7 mg, 61%. **^{1}H NMR** (400 MHz, $CDCl_3$) δ 7.95 – 7.90 (m, 2H), 7.83 – 7.75 (m, 2H), 7.56 – 7.53 (m, 1H), 7.46 – 7.34 (m, 3H), 7.31 – 7.27 (m, 2H), 7.13 (dd, J = 7.3, 1.2 Hz, 1H), 5.39 – 5.28 (m, 2H), 5.04 (dd, J = 17.1, 1.0 Hz, 1H), 4.81 (dd, J = 10.6, 1.0 Hz, 1H), 2.74 – 2.68 (m, 1H), 2.60 – 2.56 (m, 1H), 2.42 (s, 3H), 2.38 (dd, J = 10.6, 9.6 Hz, 1H), 2.19 (dd, J = 10.4, 9.6 Hz, 1H); **^{13}C NMR** (101 MHz, $CDCl_3$) δ 150.0, 144.4, 137.1, 135.4, 134.5, 134.4, 132.1, 129.5, 128.9, 128.5, 128.4, 127.7, 127.0, 126.3, 125.4, 123.3, 117.3, 68.7, 52.6, 40.6, 39.1, 21.8; **HRMS** (ESI/TOF) *m/z* Calcd for $C_{24}H_{23}N_2O_3S$ [M + H]$^+$ 419.1424; Found 419.1417.

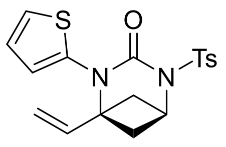

3v

The product was obtained as a light-yellow oil after purification by silica gel column chromatography, eluent: 17-20% EtOAc in hexane, 35.2 mg, 94%. **^{1}H NMR** (300 MHz, $CDCl_3$) δ 7.96 – 7.86 (m, 2H), 7.32 – 7.27 (m, 2H), 7.13 (dd, *J* = 5.6, 1.4 Hz, 1H), 6.83 (dd, *J* = 5.6, 3.7 Hz, 1H), 6.67 (dd, *J* = 3.7, 1.4 Hz, 1H), 5.67 (dd, *J* = 17.2, 10.5 Hz, 1H), 5.23 (t, *J* = 4.9 Hz, 1H), 5.16 – 5.06 (m, 2H), 2.59 – 2.49 (m, 2H), 2.41 (s, 3H), 2.10 – 1.99 (m, 2H); **^{13}C NMR** (101 MHz, $CDCl_3$) δ 150.3, 144.6, 139.7, 136.8, 134.7, 129.6, 128.5, 127.6, 125.4, 125.2, 117.5, 69.2, 52.2, 39.3, 21.8; **HRMS** (ESI/TOF) *m/z* Calcd for $C_{18}H_{19}N_2O_3S_2$ $[M + H]^+$ 375.0832; Found 375.0820.

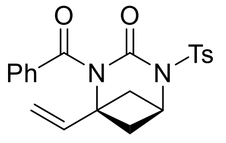

3w

The product was obtained as a white amorphous solid after purification by silica gel column chromatography, eluent: 12.5-17% EtOAc in hexane, 33.0 mg, 83% yield (335.8 mg, 85% on 1.0 mmol scale). **^{1}H NMR** (300 MHz, $CDCl_3$) δ 7.85 – 7.79 (m, 2H), 7.79 – 7.73 (m, 2H), 7.57 – 7.50 (m, 1H), 7.42 – 7.35 (m, 2H), 7.30 – 7.24 (m, 2H), 6.01 (dd, *J* = 17.3, 10.6 Hz, 1H), 5.24 – 5.14 (m, 3H), 2.64 – 2.54 (m, 2H), 2.42 (s, 3H), 2.18 – 2.08 (m, 2H); **^{13}C NMR** (101 MHz, $CDCl_3$) δ 172.6, 149.1, 144.9, 136.3, 135.6, 135.1, 133.7, 130.2, 129.7, 128.6, 128.3, 117.1, 67.4, 52.6, 39.9, 21.8; **HRMS** (ESI/TOF) *m/z* Calcd for $C_{21}H_{21}N_2O_4S$ $[M + H]^+$ 397.1217; Found 397.1218.

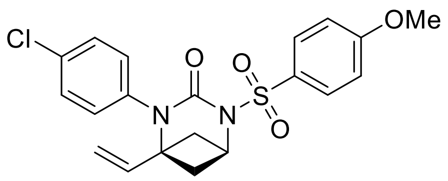

5a

The product was obtained as a colorless oil after purification by silica gel column chromatography, eluent: 20-25% EtOAc in hexane, 26.3 mg, 63% yield. **^{1}H NMR** (400 MHz, $CDCl_3$) δ 7.99 – 7.91 (m, 2H), 7.28 – 7.23 (m, 2H), 6.98 – 6.90 (m, 4H), 5.47 (dd, *J* = 17.2, 10.5 Hz, 1H), 5.25 (t, *J* = 4.9 Hz, 1H), 5.12 (dd, *J* = 17.2, 1.0 Hz, 1H), 5.07 (dd, *J* = 10.5, 0.9 Hz, 1H), 3.85 (s, 3H), 2.60 – 2.52 (m, 2H), 2.10 – 2.01 (m, 2H); **^{13}C NMR** (101 MHz, $CDCl_3$) δ 163.6, 150.2, 136.8, 135.5, 133.7, 131.4, 131.2, 130.7, 129.2, 117.4, 114.1, 68.3, 55.8, 52.3, 39.7; **HRMS** (ESI/TOF) *m/z* Calcd for $C_{20}H_{19}ClN_2NaO_4S$ $[M + Na]^+$ 441.0646; Found 441.0647.

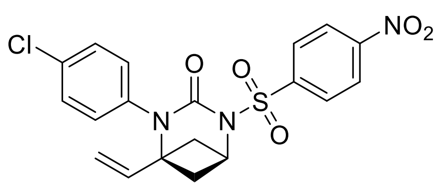

5b

The product was obtained as a white amorphous solid after purification by silica gel column chromatography, eluent: 20% EtOAc in hexane, 17.5 mg, 40% yield. **^{1}H NMR** (400 MHz, $CDCl_3$) δ 8.35 – 8.29 (m, 2H), 8.23 – 8.18 (m, 2H), 7.29 – 7.25 (m, 2H), 6.96 – 6.91 (m, 2H), 5.48 (dd, *J* = 17.2, 10.5 Hz, 1H), 5.27 (t, *J* = 4.8 Hz, 1H), 5.19 – 5.08 (m, 2H), 2.67 – 2.60 (m, 2H), 2.14 – 2.08 (m, 2H); **^{13}C NMR** (101 MHz, $CDCl_3$) δ 150.6, 149.8, 145.4, 136.2, 135.1, 134.2, 131.0, 130.0, 129.4, 124.2, 117.9, 68.5, 52.7, 39.9; **HRMS** (ESI/TOF) *m/z* Calcd for $C_{19}H_{16}N_3ClNaO_5S$ $[M + Na]^+$ 456.0391; Found 456.0373.

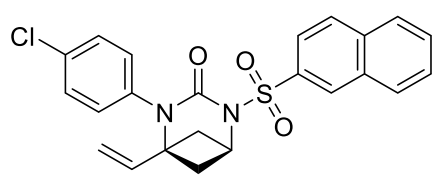

5c

The product was obtained as a white amorphous solid after purification by silica gel column chromatography, eluent: 17-25% EtOAc in hexane, 24.0 mg, 55% yield. **^{1}H NMR** (500 MHz, $CDCl_3$) δ 8.62 – 8.61 (m, 1H), 7.99 – 7.88 (m, 4H), 7.65 – 7.57 (m, 2H), 7.24 – 7.18 (m, 2H), 6.94 – 6.87 (m, 2H), 5.46 (dd, *J* = 17.2, 10.5 Hz, 1H), 5.34 (t, *J* = 4.9 Hz, 1H), 5.12 (dd, *J* = 17.2, 1.0 Hz, 1H), 5.07 (dd, *J* = 10.5, 1.0 Hz, 1H), 2.63 – 2.57 (m, 2H), 2.14 – 2.07 (m, 2H); **^{13}C NMR** (101 MHz, $CDCl_3$) δ 150.1, 136.8, 136.7, 135.5, 135.4, 133.8, 132.1, 131.2, 130.5, 129.8, 129.3, 129.2, 128.0, 127.5, 122.9, 117.5, 68.4, 52.6, 39.8; **HRMS** (ESI/TOF) *m/z* Calcd for $C_{23}H_{19}ClN_2NaO_3S$ $[M + Na]^+$ 461.0697; Found 461.0699.

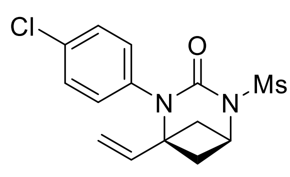

5d

The product was obtained as a white amorphous solid after purification by silica gel column chromatography, eluent: 25-33% EtOAc in hexane, 30.1 mg, 92% yield. **^{1}H NMR** (500 MHz, $CDCl_3$) δ 7.35 – 7.30 (m, 2H), 7.08 – 7.04 (m, 2H), 5.52 (dd, *J* = 17.2, 10.6 Hz, 1H), 5.19 – 5.09 (m, 2H), 5.05 (t, *J* = 4.9 Hz, 1H), 3.42 (s, 3H), 2.60 – 2.54 (m, 2H), 2.16 – 2.07 (m, 2H); **^{13}C NMR** (126 MHz, $CDCl_3$) δ 151.0, 136.6, 135.3, 133.9, 131.1, 129.3, 117.6, 68.4, 51.3, 43.3, 39.7; **HRMS** (ESI/TOF) *m/z* Calcd for $C_{14}H_{15}ClN_2O_3S$ $[M + Na]^+$ 349.0384; Found 349.0380.

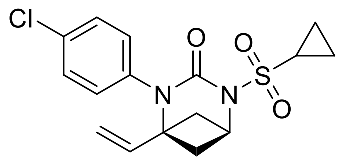

5e

The product was obtained as a white amorphous solid after purification by silica gel column chromatography, eluent: 12.5-17% EtOAc in hexane, 28.7 mg, 81% yield. **^{1}H NMR** (400 MHz, $CDCl_3$) δ 7.37 – 7.29 (m, 2H), 7.11 – 7.04 (m, 2H), 5.53 (dd, J = 17.2, 10.5 Hz, 1H), 5.20 – 5.08 (m, 2H), 4.96 (t, J = 4.8 Hz, 1H), 3.31 (tt, J = 8.1, 4.8 Hz, 1H), 2.58 – 2.51 (m, 2H), 2.17 – 2.08 (m, 2H), 1.35 – 1.31 (m, 2H), 1.11 – 1.04 (m, 2H); **^{13}C NMR** (101 MHz, $CDCl_3$) δ 151.0, 136.8, 135.5, 133.8, 131.2, 129.2, 117.5, 68.3, 51.9, 39.7, 32.6, 6.5; **HRMS** (ESI/TOF) *m/z* Calcd for $C_{16}H_{17}ClN_2NaO_3S$ $[M + Na]^+$ 375.0541; Found 375.0532.

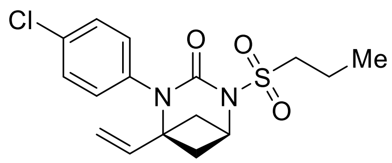

5f

The product was obtained as a white amorphous solid after purification by silica gel column chromatography, eluent: 25% EtOAc in hexane, 26.0 mg, 75% yield (278.6 mg, 80% on 1.0 mmol scale). **^{1}H NMR** (300 MHz, $CDCl_3$) δ 7.93 – 7.82 (m, 2H), 7.63 – 7.52 (m, 1H), 7.51 – 7.43 (m, 2H), 6.08 (dd, J = 17.3, 10.6 Hz, 1H), 5.29 – 5.21 (m, 2H), 4.96 (t, J = 4.8 Hz, 1H), 3.55 – 3.46 (m, 2H), 2.66 – 2.55 (m, 2H), 2.29 – 2.17 (m, 2H), 1.88 – 1.73 (m, 2H), 1.03 (t, J = 7.4 Hz, 3H); **^{13}C NMR** (101 MHz, $CDCl_3$) δ 172.7, 150.2, 135.7, 135.3, 133.9, 130.2, 128.8, 117.3, 67.5, 56.2, 51.5, 40.0, 17.2, 12.8; **HRMS** (ESI/TOF) *m/z* Calcd for $C_{17}H_{21}N_2O_4S$ $[M + H]^+$ 349.1217; Found 349.1208.

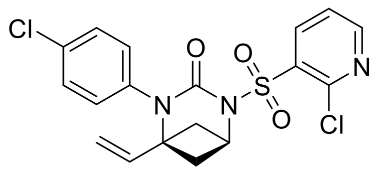

5g

The product was obtained as a white amorphous solid after purification by silica gel column chromatography, eluent: 17-25% EtOAc in hexane, 29.8 mg, 70% yield. **^{1}H NMR** (400 MHz, $CDCl_3$) δ 8.61 – 8.50 (m, 2H), 7.40 (dd, J = 7.9, 4.8 Hz, 1H), 7.28 – 7.24 (m, 2H), 7.00 – 6.89 (m, 2H), 5.51 (dd, J = 17.2, 10.5 Hz, 1H), 5.31 (t, J = 4.8 Hz, 1H), 5.18 (dd, J = 17.2, 0.9 Hz, 1H), 5.12 (dd, J = 10.5, 0.8 Hz, 1H), 2.70 – 2.63 (m, 2H), 2.34 – 2.24 (m, 2H); **^{13}C NMR** (101 MHz, $CDCl_3$) δ 153.1, 149.7, 147.8, 142.8, 136.2, 135.2, 135.0, 134.1, 131.0, 129.3, 123.0, 117.8, 68.3, 52.9, 39.8; **HRMS** (ESI/TOF) *m/z* Calcd for $C_{18}H_{15}Cl_2N_3NaO_3S$ $[M + Na]^+$ 446.0103; Found 446.0102.

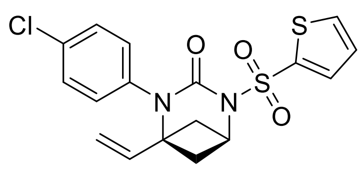

5h

The product was obtained as a colorless oil after purification by silica gel column chromatography, eluent: 20-25%% EtOAc in hexane, 27.6 mg, 70% yield. **^{1}H NMR** (400 MHz, $CDCl_3$) δ 7.86 (dd, *J* = 3.8, 1.4 Hz, 1H), 7.63 (dd, *J* = 5.0, 1.4 Hz, 1H), 7.29 – 7.25 (m, 2H), 7.06 (dd, *J* = 5.0, 3.8 Hz, 1H), 7.00 – 6.91 (m, 2H), 5.48 (dd, *J* = 17.2, 10.6 Hz, 1H), 5.22 (t, *J* = 4.9 Hz, 1H), 5.16 – 5.05 (m, 2H), 2.60 – 2.53 (m, 2H), 2.13 – 2.04 (m, 2H); **^{13}C NMR** (101 MHz, $CDCl_3$) δ 149.9, 139.8, 136.7, 135.4, 135.3, 133.9, 133.4, 131.2, 129.2, 127.3, 117.6, 68.3, 52.9, 39.6; **HRMS** (ESI/TOF) *m/z* Calcd for $C_{17}H_{15}ClN_2NaO_3S_2$ $[M + Na]^+$ 417.0105; Found 417.0102.

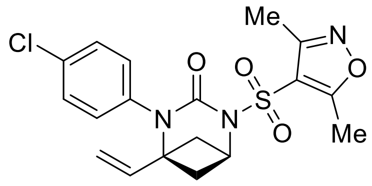

5i

The product was obtained as a white amorphous solid after purification by silica gel column chromatography, eluent: 17-25% EtOAc in hexane, 24.5 mg, 60% yield. **^{1}H NMR** (500 MHz, $CDCl_3$) δ 7.33 – 7.28 (m, 2H), 7.02 – 6.98 (m, 2H), 5.49 (dd, *J* = 17.2, 10.6 Hz, 1H), 5.24 (t, *J* = 4.9 Hz, 1H), 5.15 (dd, *J* = 17.2, 0.9 Hz, 1H), 5.11 (dd, *J* = 10.6, 0.9 Hz, 1H), 2.67 (s, 3H), 2.64 – 2.59 (m, 2H), 2.38 (s, 3H), 2.15 – 2.09 (m, 2H); **^{13}C NMR** (126 MHz, $CDCl_3$) δ 176.3, 157.5, 150.0, 136.5, 135.2, 134.2, 131.2, 129.4, 117.8, 116.0, 68.4, 52.3, 39.8, 13.4, 11.0; **HRMS** (ESI/TOF) *m/z* Calcd for $C_{18}H_{18}ClN_3NaO_4S$ $[M + Na]^+$ 430.0599; Found 430.0595.

Post-modification products

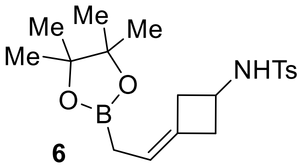

6

The product was obtained as a colorless oil after purification by silica gel column chromatography, eluent: 17% EtOAc in hexane, 21.8 mg, 58% yield. **^{1}H NMR** (400 MHz, $CDCl_3$) δ 7.81 – 7.67 (m, 2H), 7.34 – 7.27 (m, 2H), 5.19 (tt, *J* = 7.7, 2.4 Hz, 1H), 4.70 (d, *J* = 8.7 Hz, 1H), 3.87 – 3.76 (m, 1H), 2.87 – 2.75 (m, 2H), 2.43 (s, 3H), 2.42 – 2.28 (m, 2H), 1.42 (d, *J* = 7.7 Hz, 2H), 1.22 (s, 12H); **^{13}C NMR** (101 MHz, $CDCl_3$) δ 143.6, 137.7, 130.3, 129.9, 127.3, 117.8, 83.4, 44.7, 40.2, 38.5, 24.9, 21.7; **HRMS** (ESI/TOF) *m/z* Calcd for $C_{19}H_{28}BNNaO_4S$ $[M + Na]^+$ 400.1724; Found 400.1738.

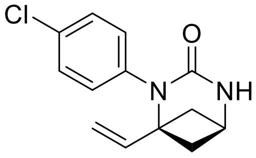

7

The product was obtained as a white amorphous solid after purification by silica gel column chromatography, eluent: first 2:1 v/v EtOAc/hexane, followed by pure EtOAc, 10.4 mg, 84% yield. **^{1}H NMR** (400 MHz, $CDCl_3$) δ 7.32 – 7.26 (m, 2H), 7.09 – 7.02 (m, 2H), 6.42 (d, *J* = 4.7 Hz, 1H), 5.58 (dd, *J* = 17.2, 10.6 Hz, 1H), 5.12 (dd, *J* = 17.2, 1.1 Hz, 1H), 5.04 (dd, *J* = 10.6, 1.1 Hz, 1H), 3.94 (q, *J* = 4.7 Hz, 1H), 2.39 – 2.32 (m, 2H), 2.04 – 1.96 (m, 2H); **^{13}C NMR** (101 MHz, $CDCl_3$) δ 155.5, 138.4, 137.1, 133.0, 131.5, 129.0, 116.3, 68.0, 47.2, 39.2; **HRMS** (ESI/TOF) *m/z* Calcd for $C_{13}H_{13}ClN_2NaO$ $[M + Na]^+$ 271.0609; Found 271.0614.

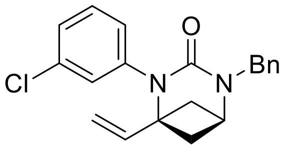

8

The product was obtained as a white amorphous solid after purification by silica gel column chromatography, eluent: 17-25% EtOAc in hexane, 81.4 mg, 80% yield. **^{1}H NMR** (500 MHz, $CDCl_3$) δ 7.37 – 7.32 (m, 4H), 7.31 – 7.21 (m, 3H), 7.17 (m, 1H), 7.07 – 7.05 (m, 1H), 5.60 (dd, *J* = 17.2, 10.6 Hz, 1H), 5.13 – 5.02 (m, 2H), 4.68 (s, 2H), 3.83 (t, *J* = 4.6 Hz, 1H), 2.34 – 2.27 (m, 2H), 1.97 – 1.90 (m, 2H); **^{13}C NMR** (101 MHz, $CDCl_3$) δ 154.8, 141.6, 138.0, 137.1, 133.9, 130.4, 129.4, 128.7, 128.5, 128.2, 127.5, 127.2, 116.2, 68.0, 51.6, 50.9, 38.9; **HRMS** (ESI/TOF) *m/z* Calcd for $C_{20}H_{20}ClN_2O$ $[M + H]^+$ 339.1259; Found 339.1263.

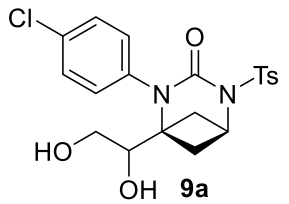

9a

The product was obtained as a white amorphous solid after purification by silica gel column chromatography, eluent: 1:1 to 2:1 v/v EtOAc/hexane, 57.0 mg, 26% yield. **^{1}H NMR** (400 MHz, DMSO) δ 7.92 – 7.84 (m, 2H), 7.56 – 7.47 (m, 4H), 7.31 – 7.21 (m, 2H), 5.14 (t, *J* = 4.8 Hz, 1H), 4.86 (d, *J* = 4.9 Hz, 1H), 4.62 (dd, *J* = 5.9, 4.8 Hz, 1H), 3.41 – 3.35 (m, 1H), 3.29 – 3.19 (m, 2H), 2.83 (dd, *J* = 9.9, 4.9 Hz, 1H), 2.74 (dd, *J* = 10.0, 4.9 Hz, 1H), 2.50 (s, 3H), 1.84 (dd, *J* = 9.4, 7.5 Hz, 2H); **^{13}C NMR** (126 MHz, DMSO) δ 149.9, 144.1, 137.1, 136.8, 132.2, 132.0, 129.5, 128.5, 127.6, 69.7, 68.3, 61.9, 52.6, 35.8, 35.6, 21.1; **HRMS** (ESI/TOF) *m/z* Calcd for $C_{20}H_{22}ClN_2O_5S$ $[M + H]^+$ 437.0932; Found 437.0927.

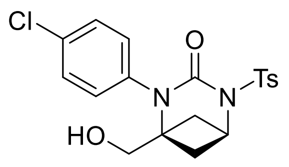

9b

The product was obtained as a white amorphous solid after purification by silica gel column chromatography, eluent: 1:2 to 1:1 v/v EtOAc/hexane, 143.8 mg, 71% yield. **^{1}H NMR** (400 MHz, DMSO) δ 7.82 – 7.73 (m, 2H), 7.45 – 7.36 (m, 4H), 7.19 – 7.13 (m, 2H), 5.09 (t, *J* = 4.7 Hz, 1H), 4.89 (t, *J* = 5.1 Hz, 1H), 3.04 (d, *J* = 5.1 Hz, 2H), 2.60 – 2.53 (m, 2H), 2.39 (s, 3H), 1.77 – 1.71 (m, 2H); **^{13}C NMR** (101 MHz, DMSO) δ 149.6, 144.1, 136.9, 136.5, 132.4, 131.9, 129.5, 128.6, 127.6, 67.4, 60.8, 53.1, 36.0, 21.1; **HRMS** (ESI/TOF) *m/z* Calcd for $C_{19}H_{20}ClN_2O_4S$ $[M + H]^+$ 407.0827; Found 407.0813.

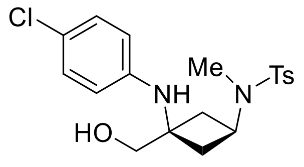

10

The product was obtained as a white amorphous solid after purification by silica gel column chromatography, eluent: 33-50% EtOAc in hexane, 72.3 mg, 61% yield. **^{1}H NMR** (500 MHz, DMSO) δ 7.80 (s, 1H), 7.67 – 7.66 (m, 2H), 7.40 – 7.39 (m, 2H), 7.12 – 7.05 (m, 2H), 7.49 – 7.46 (m, 2H), 4.93 (t, *J* = 5.6 Hz, 1H), 3.45 – 3.44 (m, 3H), 2.64 (s, 3H), 2.44 – 2.40 (m, 5H), 1.91 – 1.87 (m, 2H); **^{13}C NMR** (126 MHz, DMSO) δ 146.0, 142.6, 138.7, 129.6, 128.1, 126.4, 119.2, 114.6, 62.3, 58.3, 40.3, 38.6, 34.2, 21.0; **HRMS** (ESI/TOF) *m/z* Calcd for $C_{19}H_{24}ClN_2O_3S$ $[M + H]^+$ 395.1191; Found 395.1190.

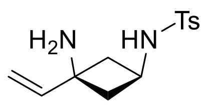

11

The product was obtained as a white amorphous solid after purification by silica gel column chromatography, eluent: 5%-10% MeOH in DCM, 21.9 mg, 82% yield. **^{1}H NMR** (300 MHz, $CDCl_3$) δ 7.77 – 7.68 (m, 2H), 7.32 – 7.24 (m, 2H), 5.90 (dd, *J* = 17.2, 10.6 Hz, 1H), 5.12 – 4.96 (m, 2H), 3.63 – 3.53 (m, 1H), 2.46 – 2.33 (m, 5H), 1.80 – 1.69 (m, 2H); **^{13}C NMR** (101 MHz, $CDCl_3$) δ 143.6, 143.4, 137.9, 129.8, 127.2, 111.8, 52.6, 44.1, 41.9, 21.7; **HRMS** (ESI/TOF) *m/z* Calcd for $C_{13}H_{19}N_2O_2S$ $[M + H]^+$ 267.1162; Found 267.1167.

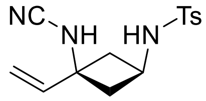

12

The product was obtained as a light-yellow oil after purification by silica gel column chromatography, eluent: 50% EtOAc in hexane, 18.8 mg, 68% yield. **^{1}H NMR** (300 MHz, $CDCl_3$) δ 7.77 – 7.69 (m, 2H), 7.34 – 7.27 (m, 2H), 5.84 (dd, *J* = 17.2, 10.5 Hz, 1H), 5.64 (d, *J* = 9.3 Hz, 1H), 5.33 – 5.23 (m, 2H), 4.31 (s, 1H), 3.62 – 3.49 (m, 1H), 2.52 – 2.37 (m, 5H), 2.22 – 2.14 (m, 2H); **^{13}C NMR** (126 MHz, $CDCl_3$) δ 144.0, 137.5, 130.0, 128.8, 127.1, 116.5, 113.9, 54.5, 41.8, 40.7, 21.7; **HRMS** (ESI/TOF) *m/z* Calcd for $C_{14}H_{17}N_3NaO_2S$ $[M + Na]^+$ 314.0934; Found 314.0938.

Drug-mimic syntheses

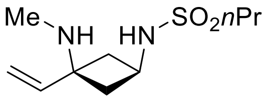

13

The product was obtained as a colorless oil after purification by silica gel column chromatography, eluent: 10% MeOH in DCM, 58.3 mg, 63% yield in two steps. **^{1}H NMR** (500 MHz, $CDCl_3$) δ 5.77 (dd, *J* = 17.4, 10.6 Hz, 1H), 5.23 – 5.11 (m, 2H), 3.74 – 3.68 (m, 1H), 2.97 – 2.91 (m, 2H), 2.62 – 2.55 (m, 2H), 2.20 (s, 3H), 1.91 – 1.78 (m, 4H), 1.05 (t, *J* = 7.5 Hz, 3H); **^{13}C NMR** (126 MHz, $CDCl_3$) δ 140.8, 114.6, 56.8, 55.3, 42.8, 41.4, 29.4, 17.5, 13.1; **HRMS** (ESI/TOF) *m/z* Calcd for $C_{10}H_{21}N_2O_2S$ $[M + H]^+$ 233.1318; Found 233.1324.

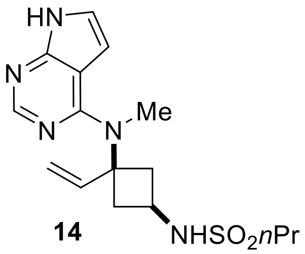

14

The product was obtained as a white amorphous solid after purification by silica gel column chromatography, recrystallized from Et_2O, 44.4 mg, 51% yield in two steps. **^{1}H NMR** (300 MHz, DMSO) δ 8.05 (s, 1H), δ 7.36 (d, *J* = 8.9 Hz, 1H), 7.14 (dd, *J* = 3.6, 2.2 Hz, 1H), 6.56 (dd, *J* = 3.6, 1.7 Hz, 1H), 6.22 (dd, *J* = 17.3, 10.5 Hz, 1H), 5.20 – 5.08 (m, 2H), 3.54 (m, 1H), 3.12 (s, 3H), 2.98 – 2.83 (m, 4H), 2.29 – 2.22 (m, 2H), 1.72 – 1.58 (m, 2H), 0.98 (t, *J* = 7.4 Hz, 3H); **^{13}C NMR** (126 MHz, DMSO) δ 156.0, 151.7, 150.2, 138.2, 120.8, 112.9, 102.9, 101.2, 58.5, 53.7, 41.8, 41.2, 33.7, 16.9, 12.7; **HRMS** (ESI/TOF) *m/z* Calcd for $C_{16}H_{24}N_5O_2S$ $[M + H]^+$ 350.1645; Found 350.1657.

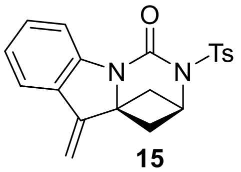

15

The product was obtained as a white amorphous solid after purification by silica gel column chromatography, eluent: 17% EtOAc in hexane, 25.7 mg, 70% yield. **^{1}H NMR** (400 MHz, $CDCl_3$) δ 7.97 – 7.88 (m, 3H), 7.41 – 7.39 (m, 1H), 7.34 – 7.29 (m, 2H), 7.28 – 7.23 (m, 1H), 7.04 – 7.00 (m, 1H), 5.57 (d, *J* = 0.9 Hz, 1H), 5.27 (t, *J* = 4.8 Hz, 1H), 5.14 (d, *J* = 0.9 Hz, 1H), 2.62 – 2.50 (m, 2H), 2.42 (s, 3H), 2.12 – 2.02 (m, 2H); **^{13}C NMR** (101 MHz, $CDCl_3$) δ 145.9, 144.7, 143.5, 141.8, 137.0, 130.7, 129.7, 128.2, 127.5, 123.9, 120.8, 115.6, 103.5, 72.0, 53.5, 41.9, 21.8; **HRMS** (ESI/TOF) *m/z* Calcd for $C_{20}H_{19}N_2O_3S$ $[M + H]^+$ 367.1111; Found 367.1106.

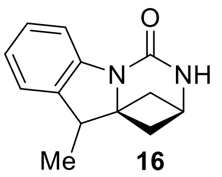

16

The product was obtained as a white amorphous solid after purification by silica gel column chromatography, eluent: 200% EtOAc in hexane, 39.8 mg, 93% yield. **^{1}H NMR** (400 MHz, $CDCl_3$) δ 7.91 – 7.82 (m, 1H), 7.20 – 7.16 (m, 1H), 7.12 – 7.09 (m, 1H), 6.95 – 6.91 (m, 1H), 6.40 – 6.31 (m, 1H), 3.97 (q, *J* = 4.7 Hz, 1H), 3.32 (q, *J* = 7.2 Hz, 1H), 2.31 (dd, *J* = 9.7, 4.6 Hz, 1H), 2.21 (dd, *J* = 9.6, 4.6 Hz, 1H), 1.91 (t, *J* = 9.7 Hz, 1H), 1.72 (t, *J* = 9.8 Hz, 1H), 1.27 (d, *J* = 7.2 Hz, 3H); **^{13}C NMR** (101 MHz, $CDCl_3$) δ 153.1, 141.4, 133.2, 128.1, 123.8, 122.2, 113.7, 73.4, 47.8, 40.1, 39.4, 35.4, 16.6; **HRMS** (ESI/TOF) m/z Calcd for $C_{13}H_{14}N_2NaO$ $[M + Na]^+$ 237.0998; Found 237.0998.

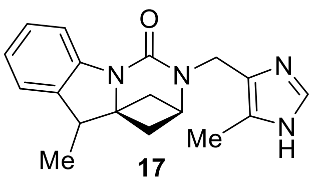

17

The product was obtained as a white amorphous solid after purification by silica gel column chromatography, eluent: 5-10% MeOH in DCM, 22.1 mg, 48% yield. **^{1}H NMR** (300 MHz, $CDCl_3$) δ 10.00 (s, 1H), 7.83 (d, *J* = 7.9 Hz, 1H), 7.63 (s, 1H), 7.11 (dd, *J* = 25.9, 7.5 Hz, 2H), 6.90 (t, *J* = 7.4 Hz, 1H), 4.54 (s, 2H), 4.02 (s, 1H), 3.23 (q, *J* = 6.7 Hz, 1H), 2.29 (s, 3H), 2.23 (dd, *J* = 9.7, 4.5 Hz, 1H), 2.18 – 2.09 (m, 1H), 1.71 (t, *J* = 9.7 Hz, 1H), 1.53 (t, *J* = 9.7 Hz, 1H), 1.20 (d, *J* = 7.1 Hz, 3H); **^{13}C NMR** (126 MHz, $CDCl_3$) δ 152.6, 141.4, 133.40, 133.38, 128.9, 128.0, 127.3, 123.9, 122.3, 113.4, 73.7, 53.5, 41.1, 40.0, 38.8, 34.9, 16.5, 10.7; **HRMS** (ESI/TOF) *m/z* Calcd for $C_{18}H_{21}N_4O$ $[M + H]^+$ 309.1710; Found 309.1716.

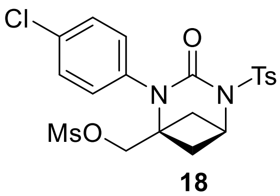

18

The product was obtained as a white amorphous solid after purification by silica gel column chromatography, eluent: 50% EtOAc in hexane, 137.6 mg, 95% yield. **^{1}H NMR** (400 MHz, $CDCl_3$) δ 7.90 – 7.84 (m, 2H), 7.38 – 7.32 (m, 2H), 7.31 – 7.27 (m, 2H), 7.08 – 7.03 (m, 2H), 5.31 (t, *J* = 4.9 Hz, 1H), 3.95 (s, 2H), 2.74 (s, 3H), 2.66 – 2.59 (m, 2H), 2.42 (s, 3H), 2.04 – 1.96 (m, 2H); **^{13}C NMR** (101 MHz, $CDCl_3$) δ 150.1, 144.8, 136.7, 135.4, 135.1, 131.5, 129.8, 129.7, 128.5, 68.0, 64.7, 52.9, 37.9, 37.4, 21.8; **HRMS** (ESI/TOF) *m/z* Calcd for $C_{20}H_{22}ClN_2O_6S_2$ $[M + H]^+$ 485.0602; Found 485.0606.

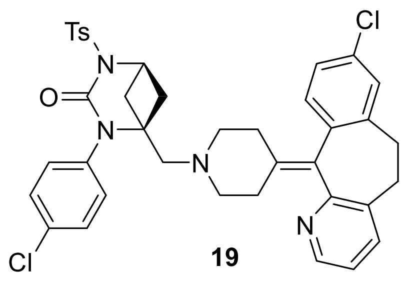

19

The product was obtained as a white amorphous solid after purification by silica gel column chromatography, eluent: 50% EtOAc in hexane, 36.5 mg, 52% yield. **^{1}H NMR** (500 MHz, $CDCl_3$) δ 8.37 (dd, *J* = 4.8, 1.7 Hz, 1H), 7.90 – 7.85 (m, 2H), 7.40 (dd, *J* = 7.7, 1.7 Hz, 1H), 7.31 – 7.26 (m, 2H), 7.25 (d, *J* = 5.0 Hz, 2H), 7.14 – 7.04 (m, 4H), 7.03 – 6.98 (m, 2H), 5.24 (t, *J* = 4.8 Hz, 1H), 3.36 – 3.25 (m, 2H), 2.83 – 2.71 (m, 2H), 2.58 – 2.53 (m, 2H), 2.40 (s, 3H), 2.37 – 2.28 (m, 3H), 2.24 – 2.11 (m, 5H), 2.02 – 1.95 (m, 2H), 1.88 – 1.81 (m, 2H); **^{13}C NMR** (126 MHz, $CDCl_3$) δ 157.4, 150.6, 146.7, 144.4, 139.6, 137.9, 137.5, 137.1, 136.6, 133.9, 133.5, 132.9, 131.7, 131.6, 131.3, 130.8, 129.5, 129.03, 128.97, 128.4, 126.2, 122.3, 66.7, 60.3, 56.12, 56.09, 53.6, 38.50, 38.47, 31.8, 31.6, 31.1, 30.9, 21.8; **HRMS** (ESI/TOF) *m/z* Calcd for $C_{38}H_{37}Cl_2N_4O_3S$ $[M + H]^+$ 699.1958; Found 699.1960.

5. Copies of NMR spectra

^{1}H NMR (**1a**, $CDCl_3$, 300 MHz)

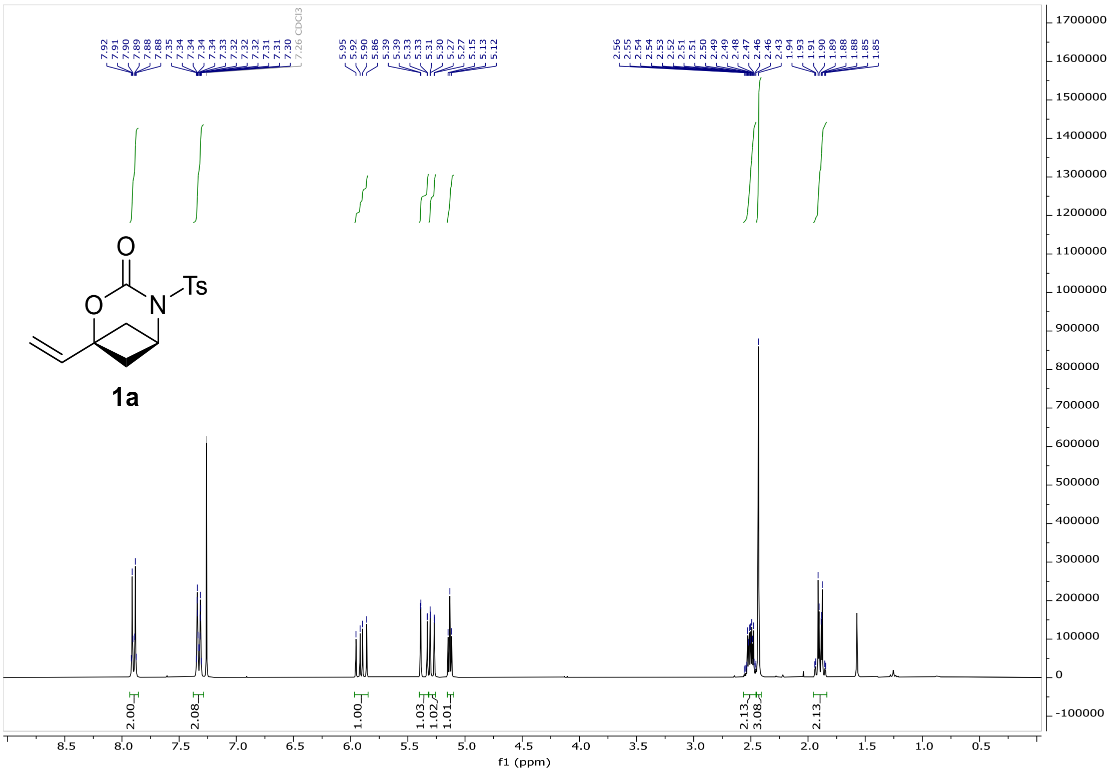

^{13}C NMR (**1a**, $CDCl_3$, 101 MHz)

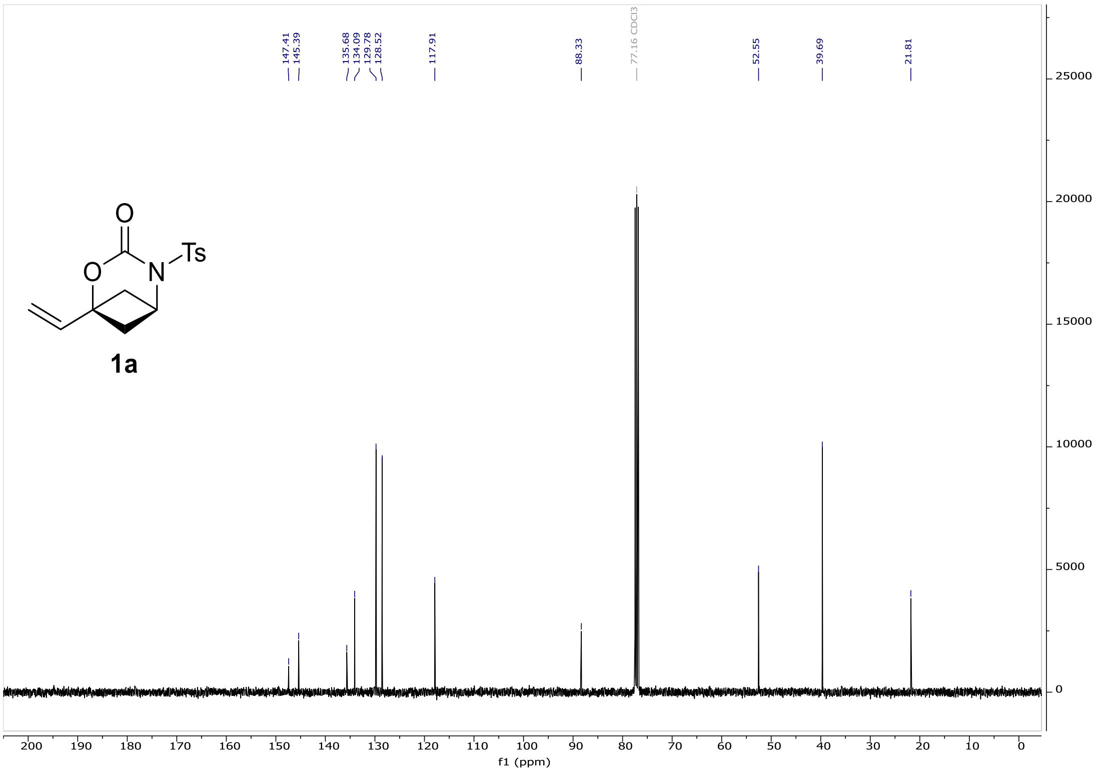

^{1}H NMR (**1b**, $CDCl_3$, 400 MHz)

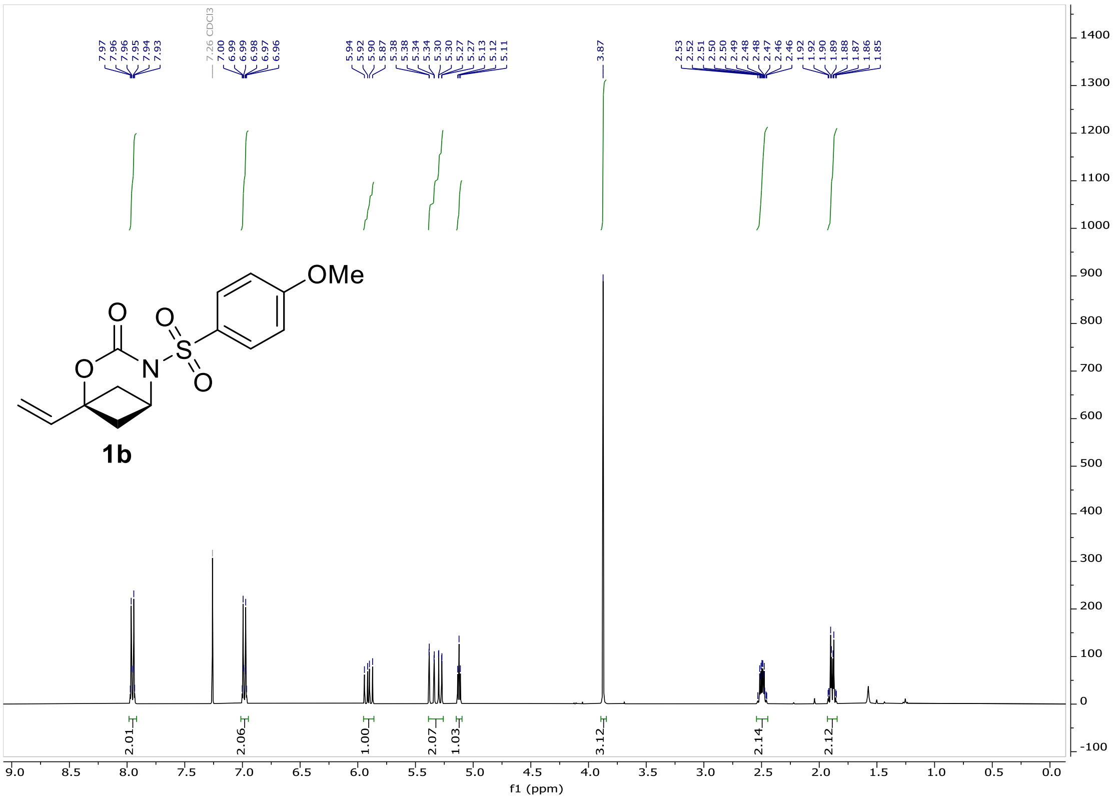

^{13}C NMR (**1b**, $CDCl_3$, 101 MHz)

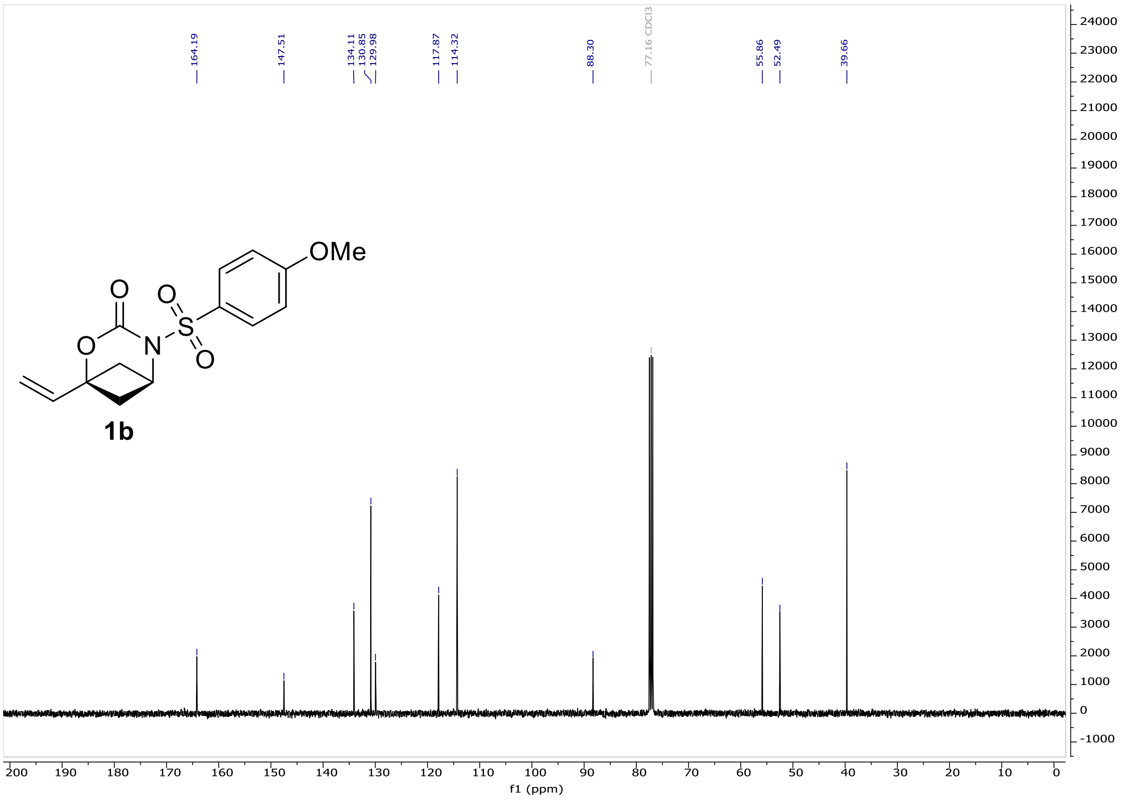

^{1}H NMR (**1c**, $CDCl_3$, 400 MHz)

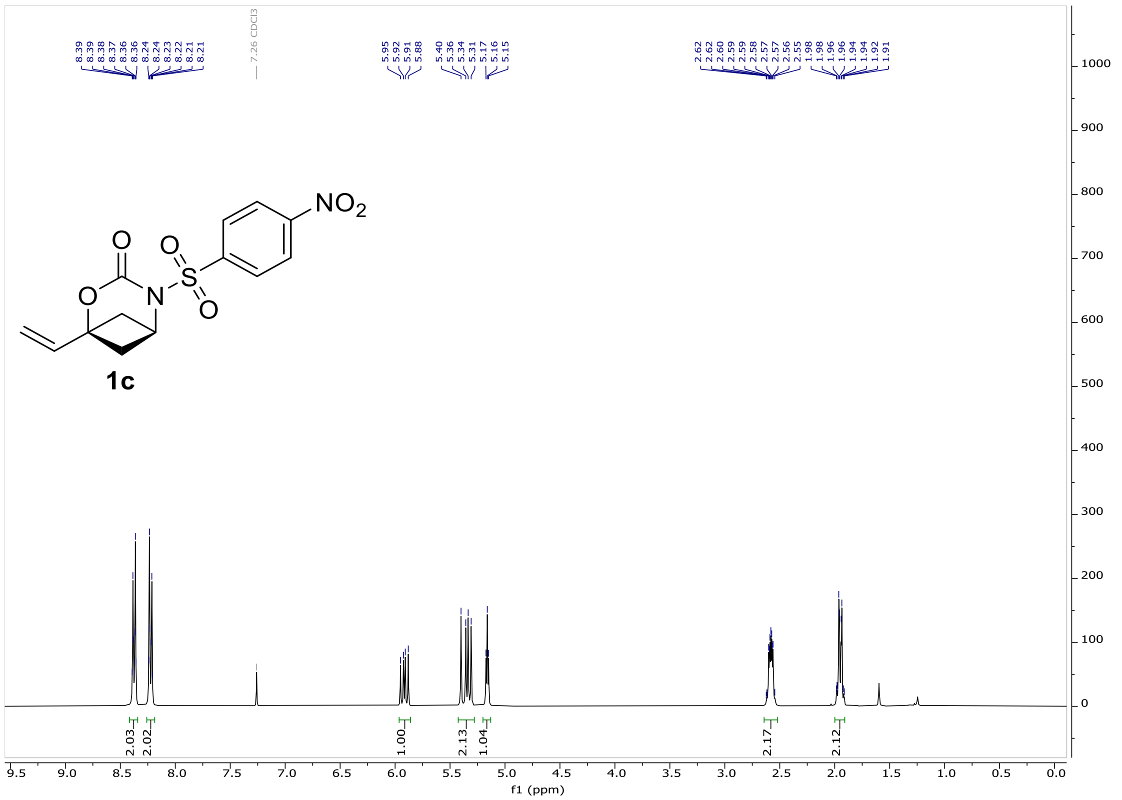

1c

^{13}C NMR (**1c**, $CDCl_3$, 101 MHz)

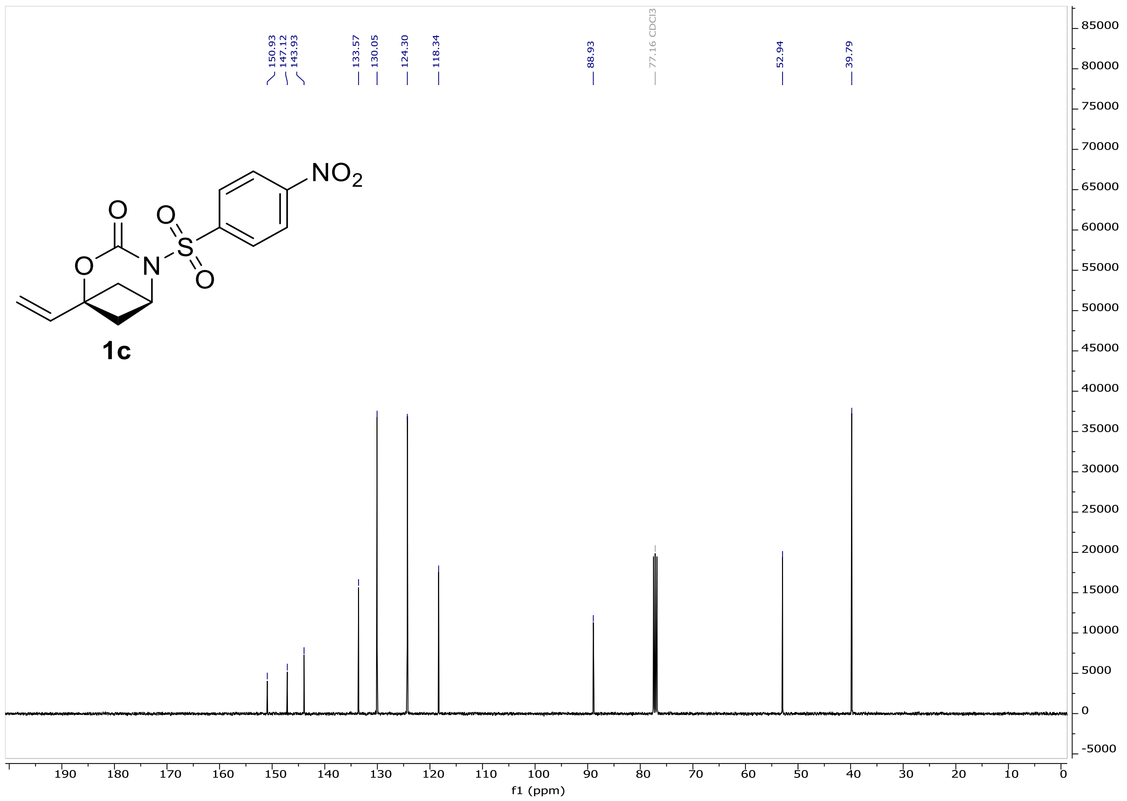

1c

^{1}H NMR (**1d**, $CDCl_3$, 500 MHz)

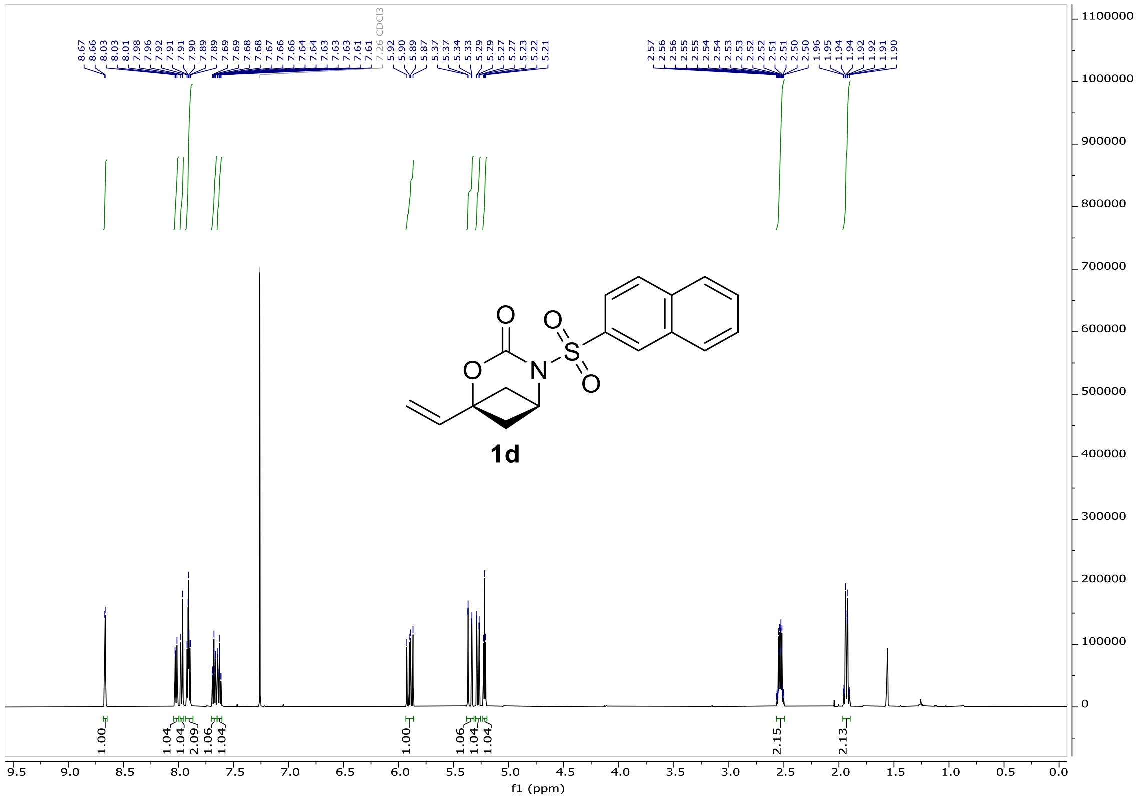

^{13}C NMR (**1d**, $CDCl_3$, 101 MHz)

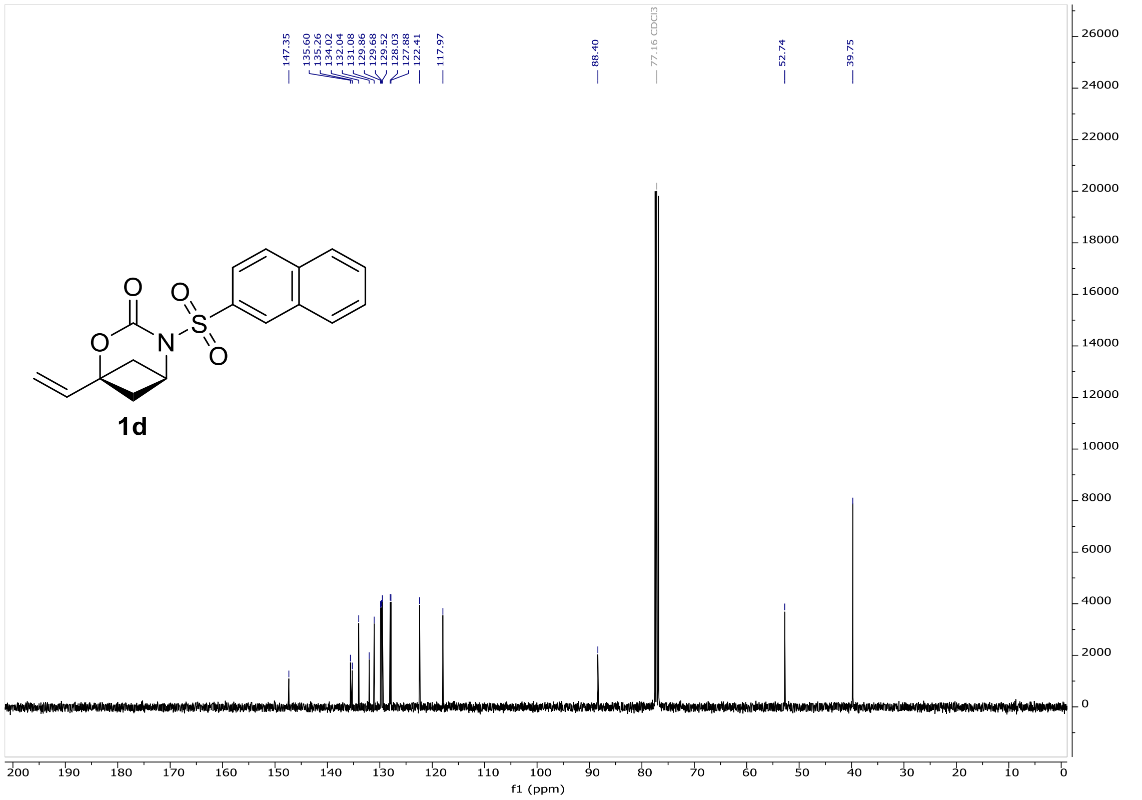

^{1}H NMR (**1e**, $CDCl_3$, 500 MHz)

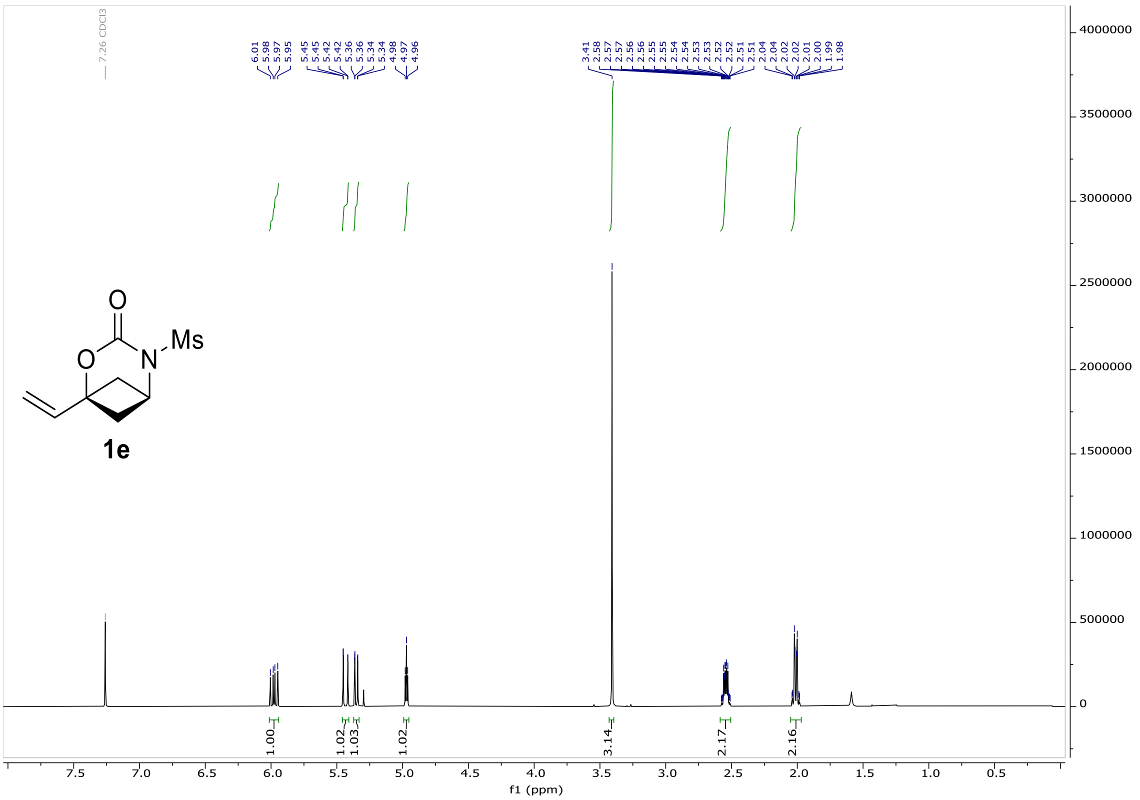

^{13}C NMR (**1e**, $CDCl_3$, 126 MHz)

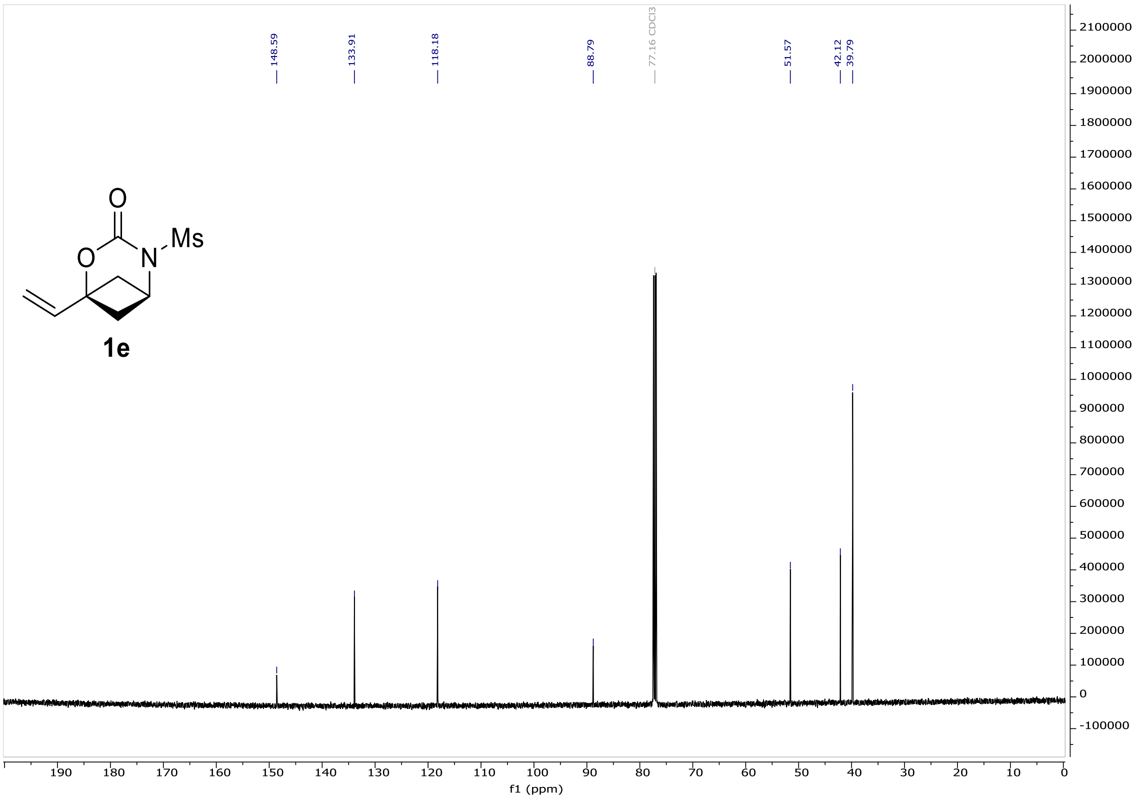

^{1}H NMR (**1f**, $CDCl_3$, 400 MHz)

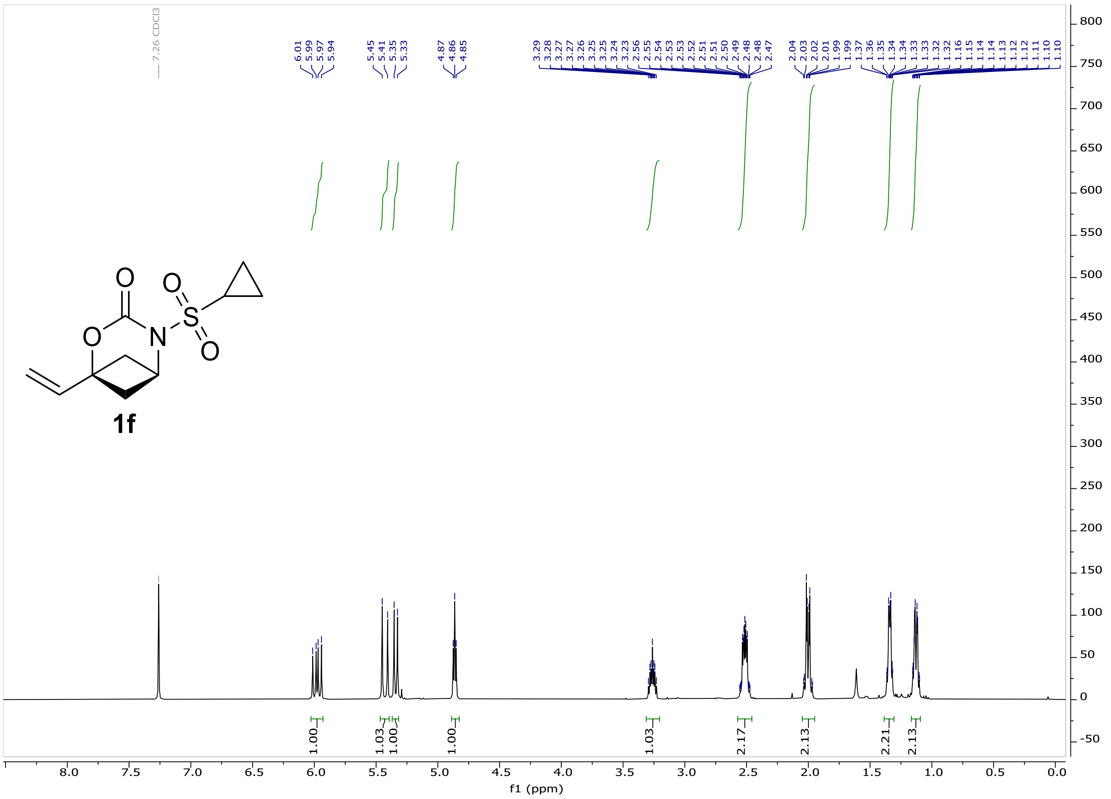

^{13}C NMR (**1f**, $CDCl_3$, 101 MHz)

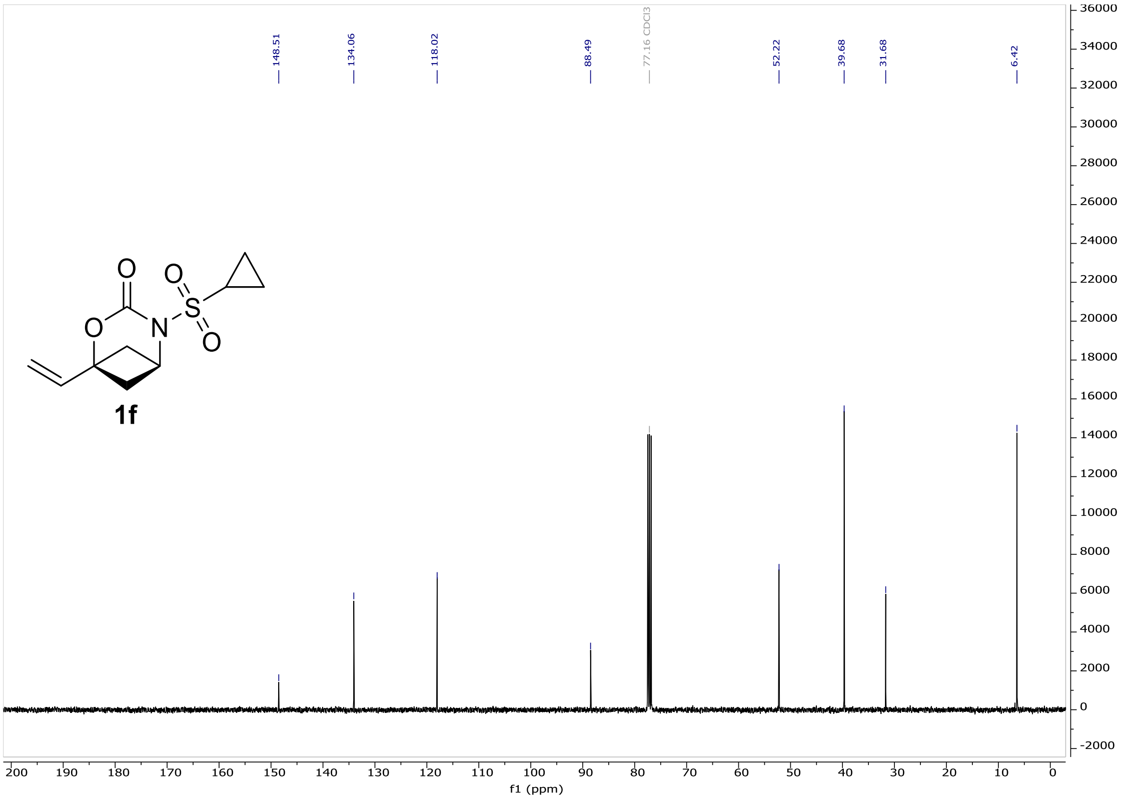

^{1}H NMR (**1g**, $CDCl_3$, 400 MHz)

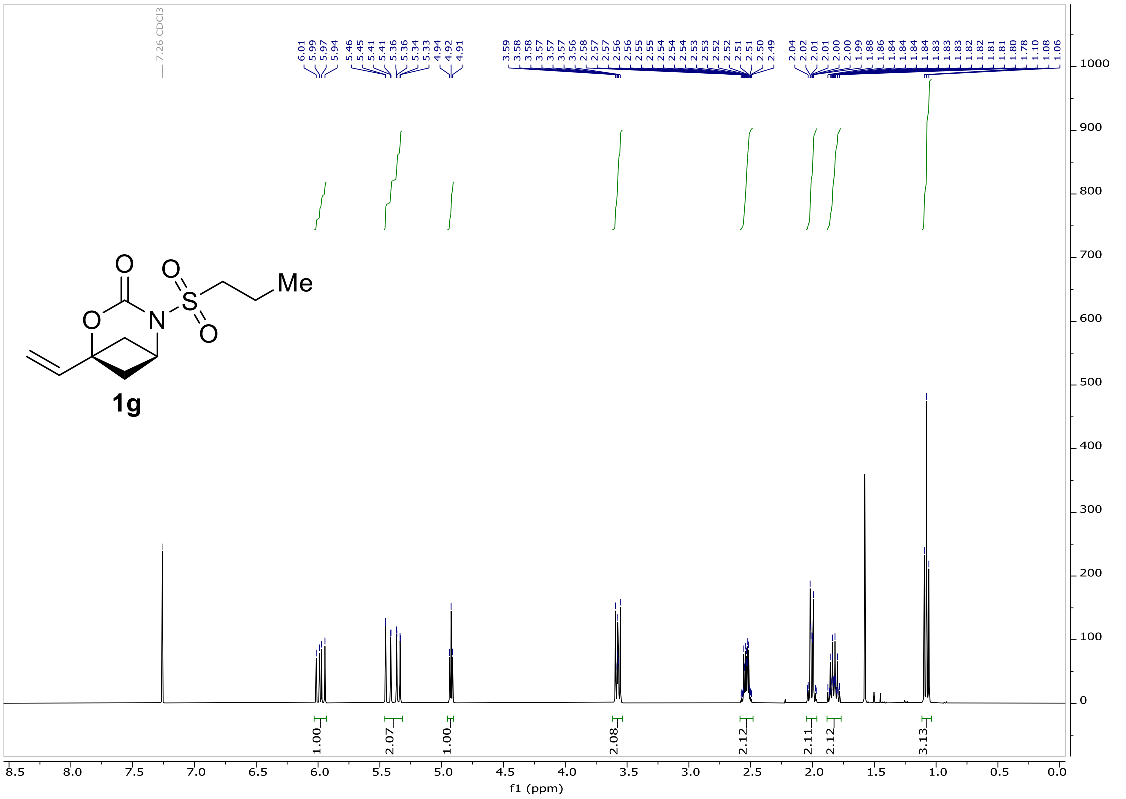

^{13}C NMR (**1g**, $CDCl_3$, 101 MHz)

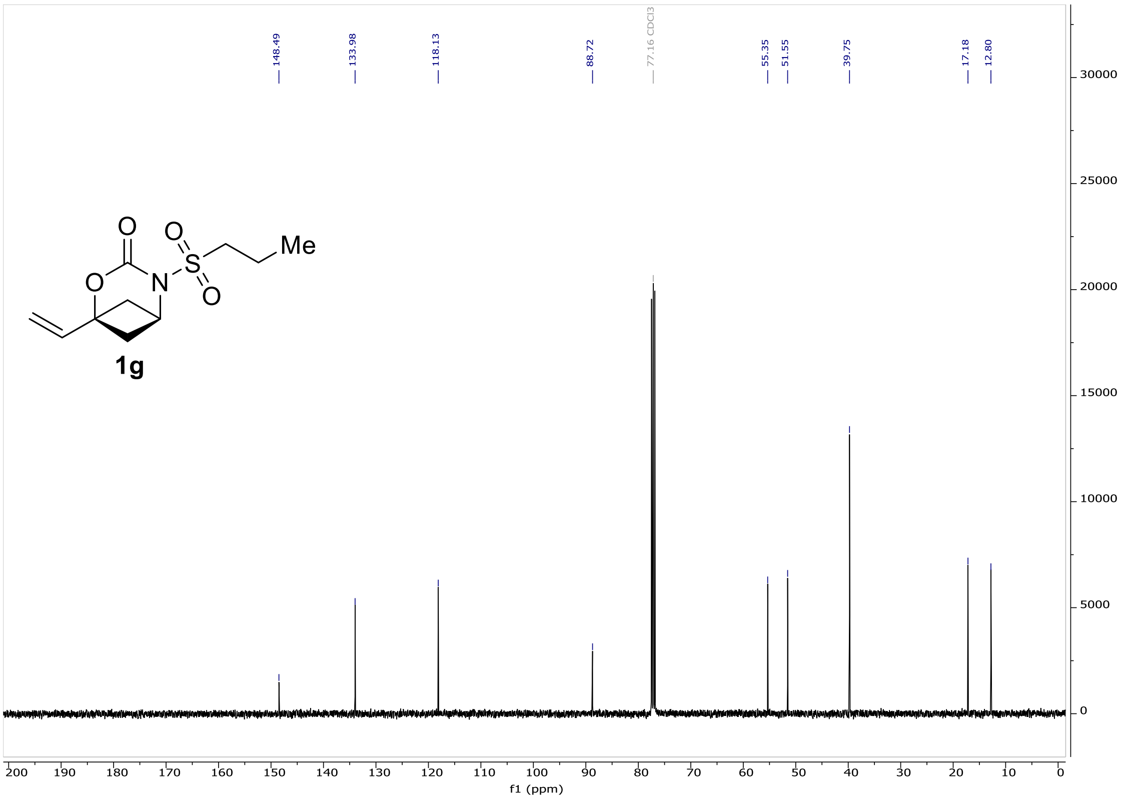

^{1}H NMR (**1h**, $CDCl_3$, 400 MHz)

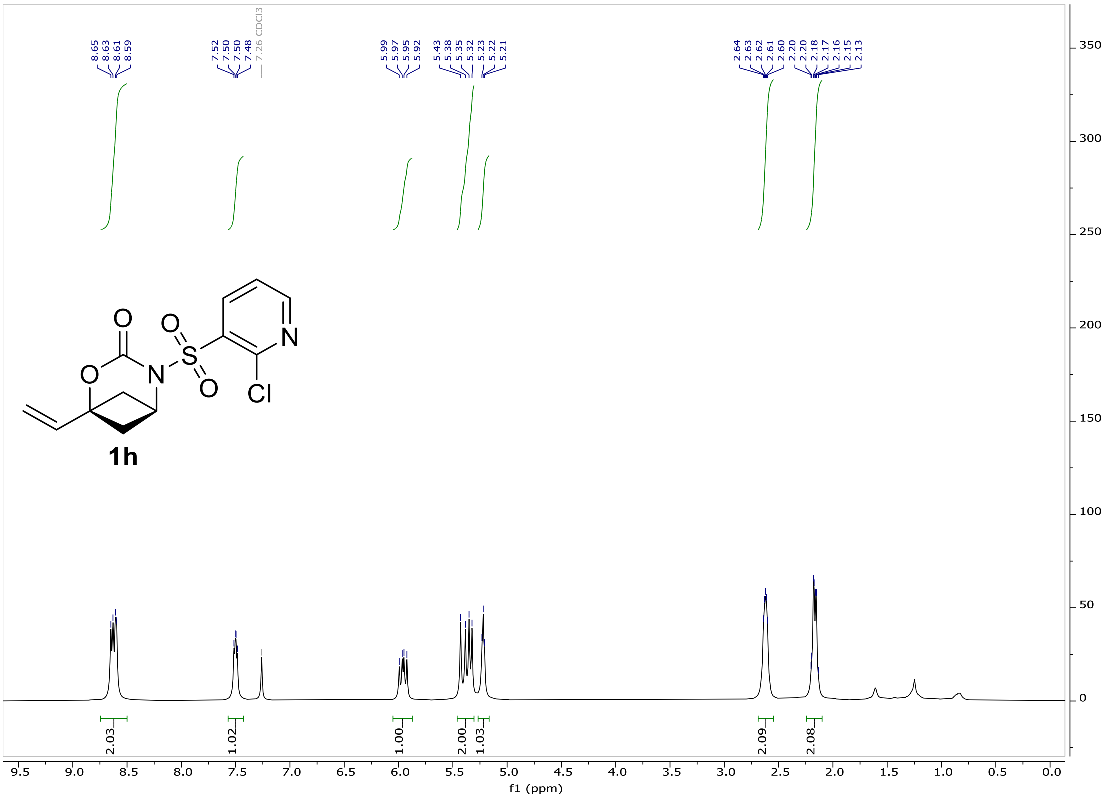

^{13}C NMR (**1h**, $CDCl_3$, 101 MHz)

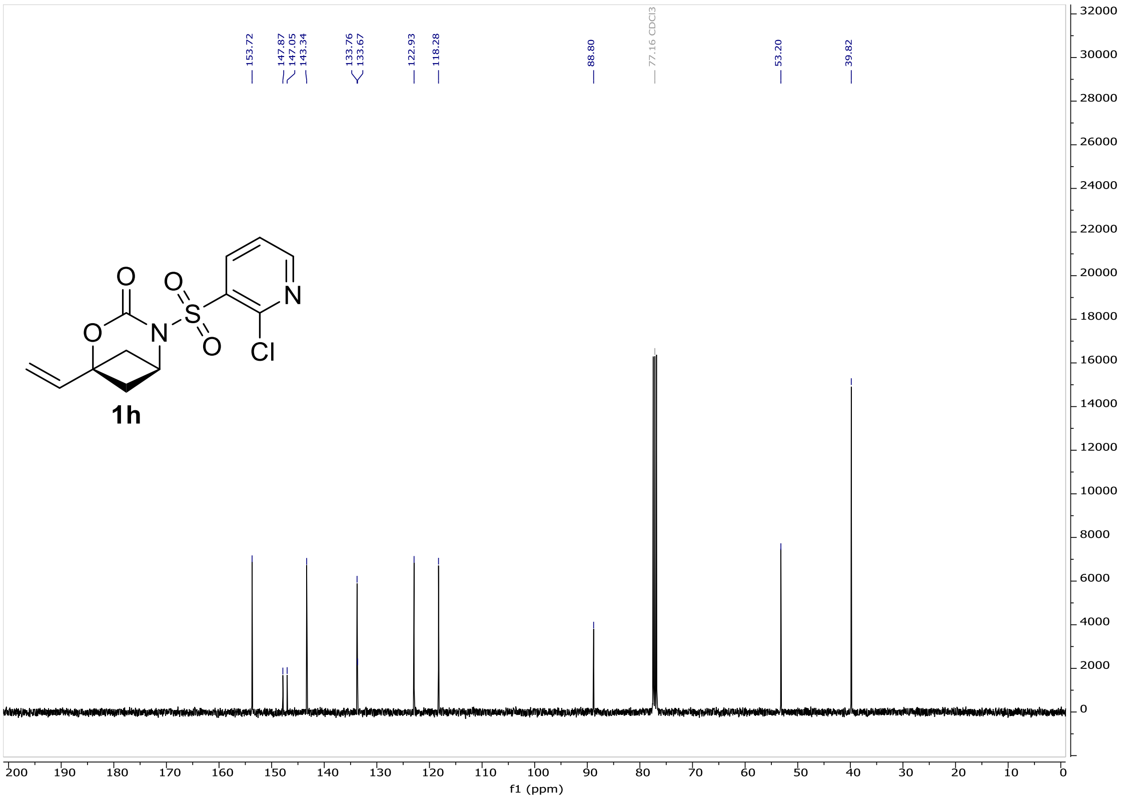

^{1}H NMR (**1i**, $CDCl_3$, 400 MHz)

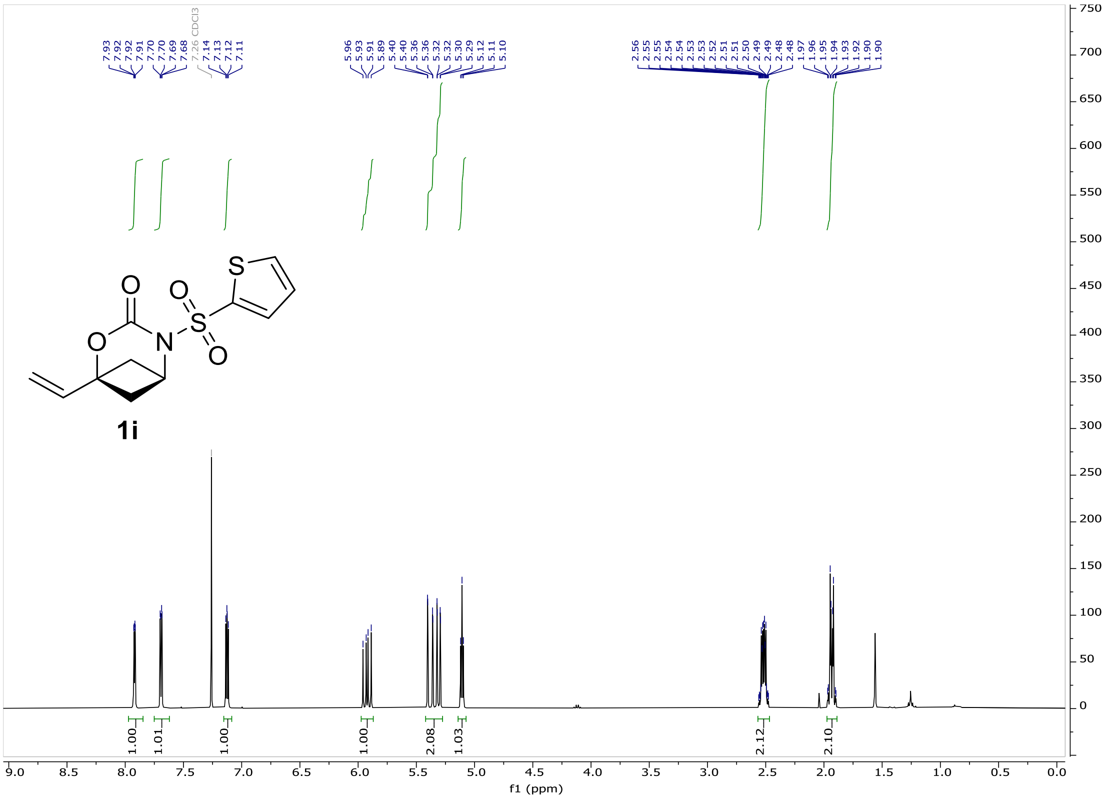

^{13}C NMR (**1i**, $CDCl_3$, 101 MHz)

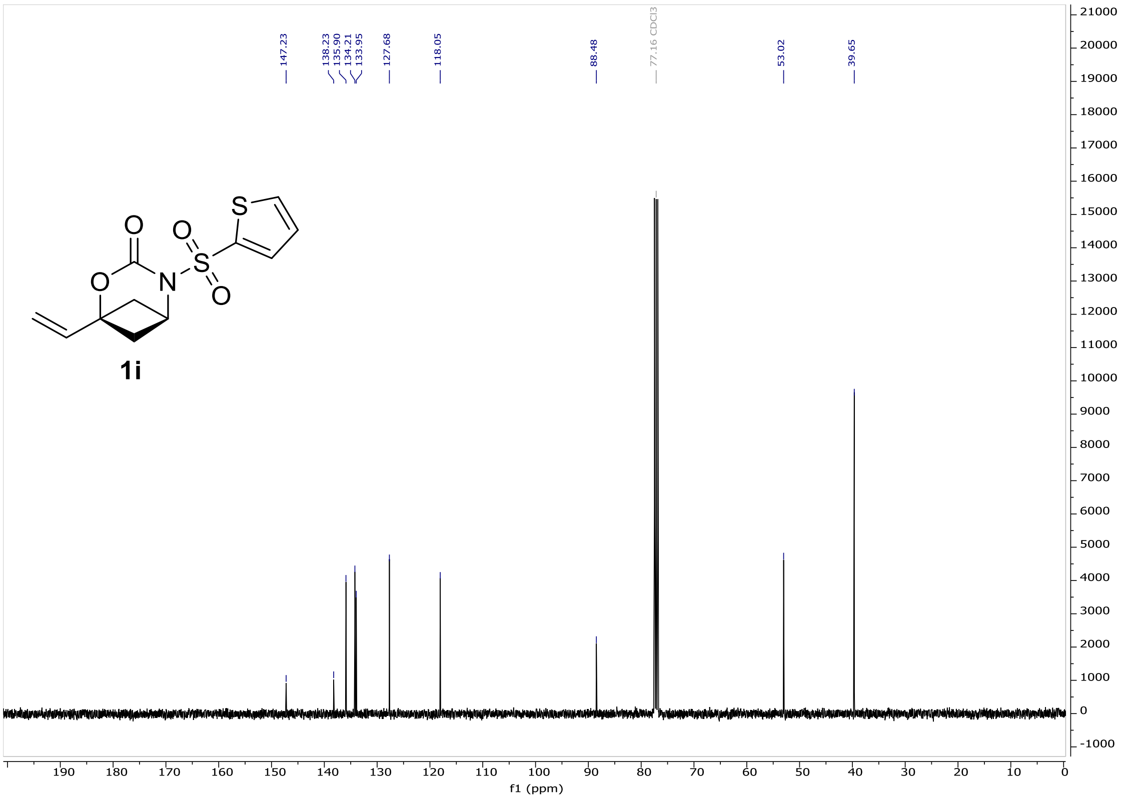

^{1}H NMR (**1j**, CDCl$_3$, 500 MHz)

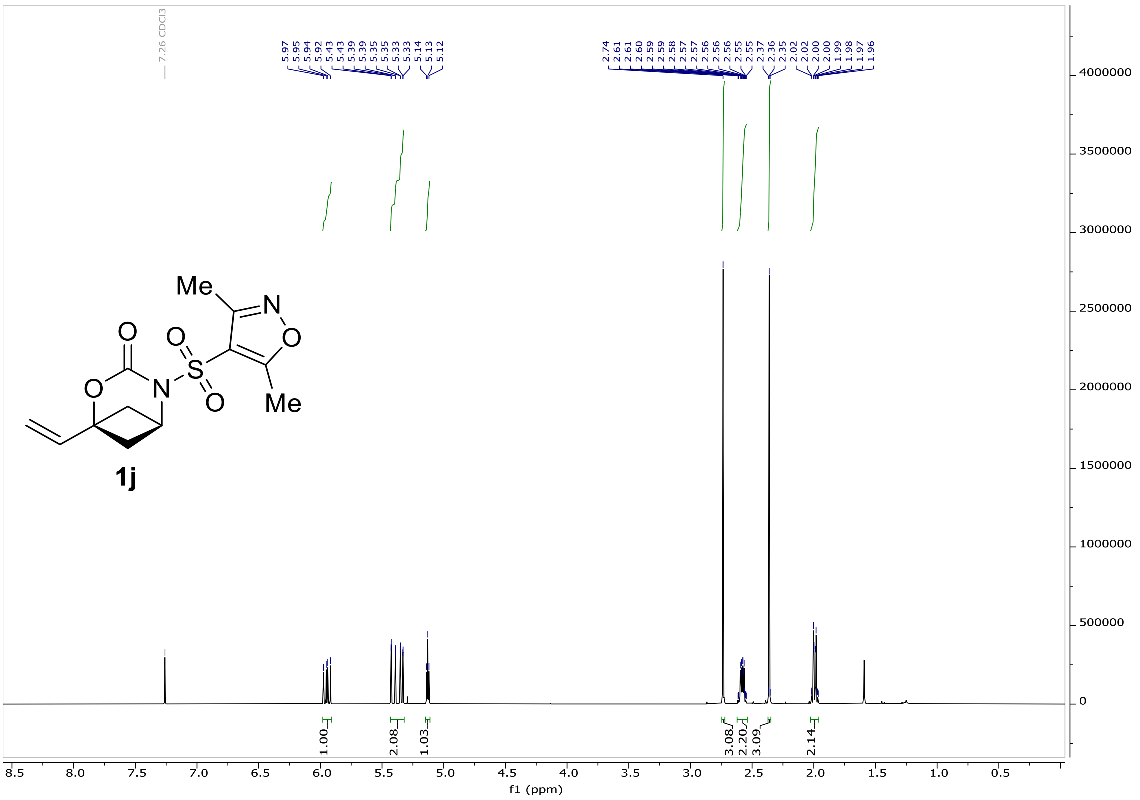

^{13}C NMR (**1j**, CDCl$_3$, 101 MHz)

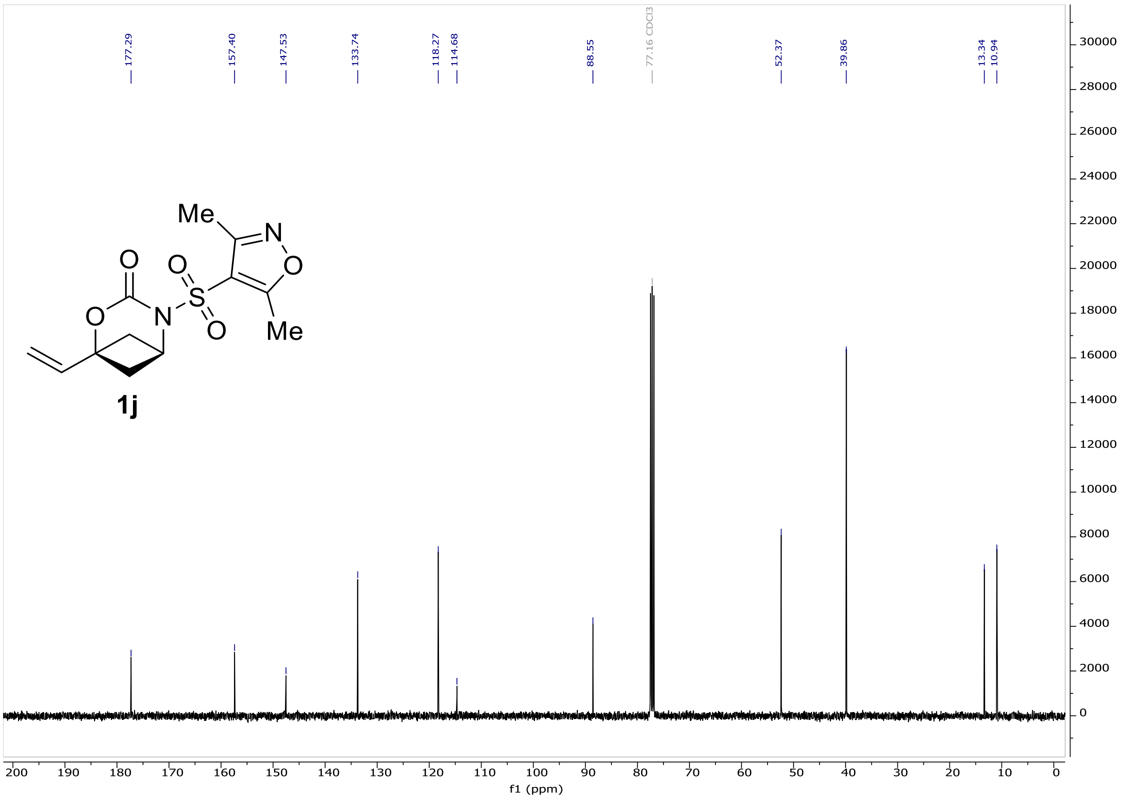

^{1}H NMR (**1k**, $CDCl_3$, 400 MHz)

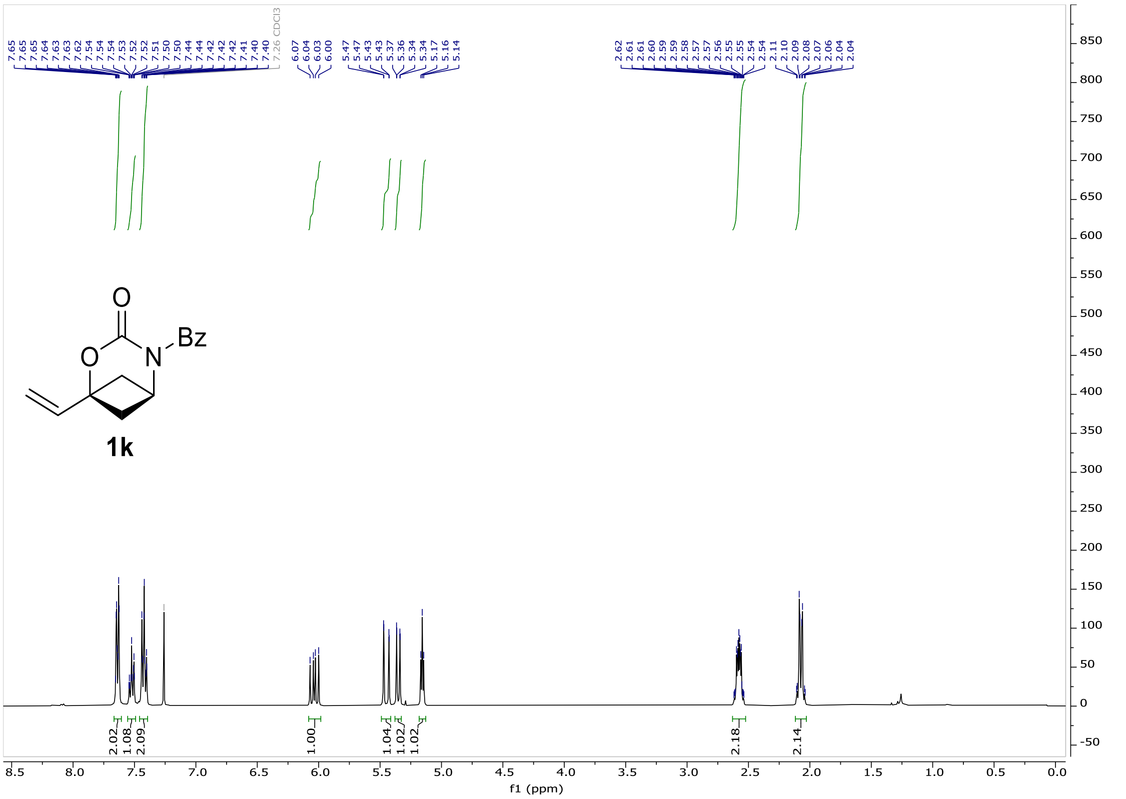

^{13}C NMR (**1k**, $CDCl_3$, 101 MHz)

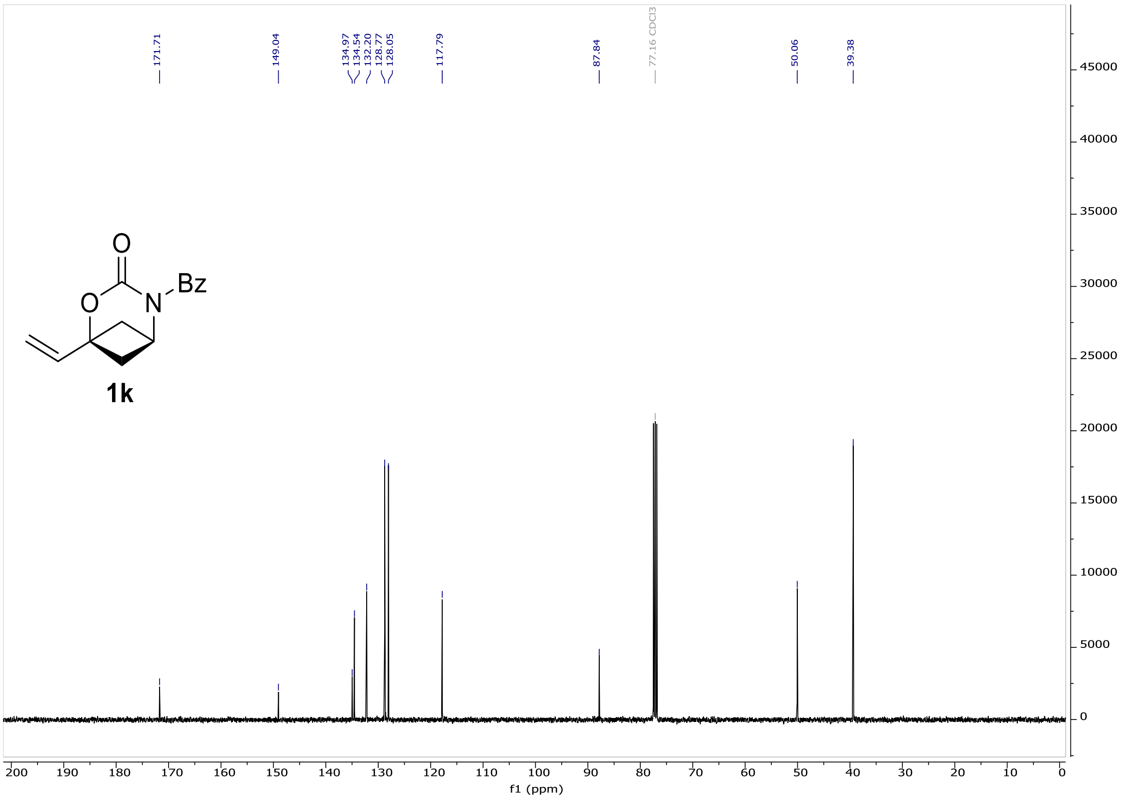

^{1}H NMR (**1l**, $CDCl_3$, 500 MHz)

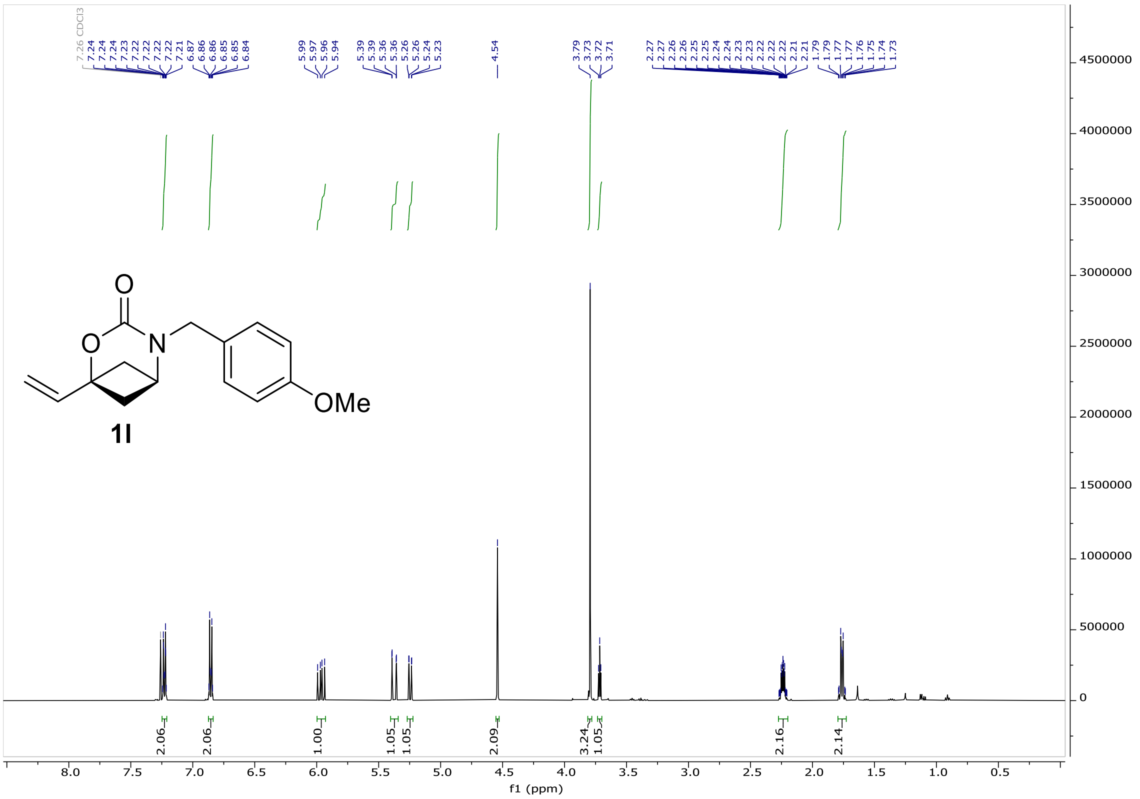

^{13}C NMR (**1l**, $CDCl_3$, 126 MHz)

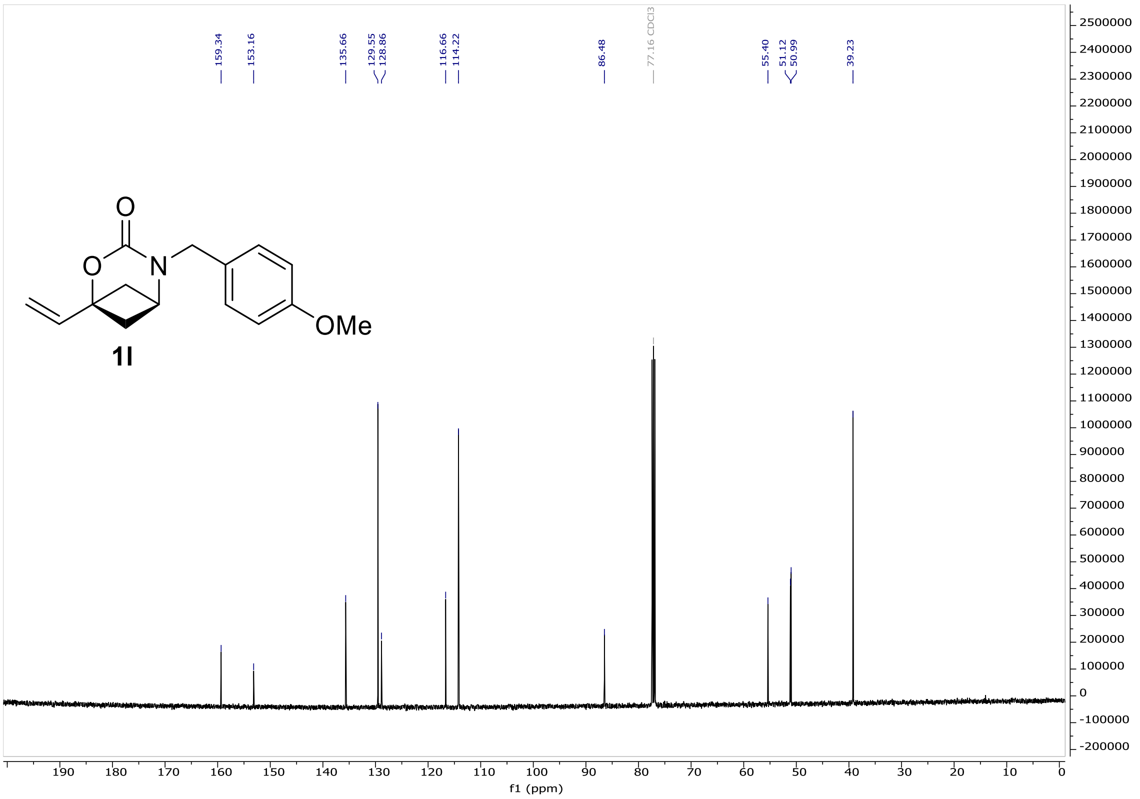

^{1}H NMR (**S1**, $CDCl_3$, 500 MHz)

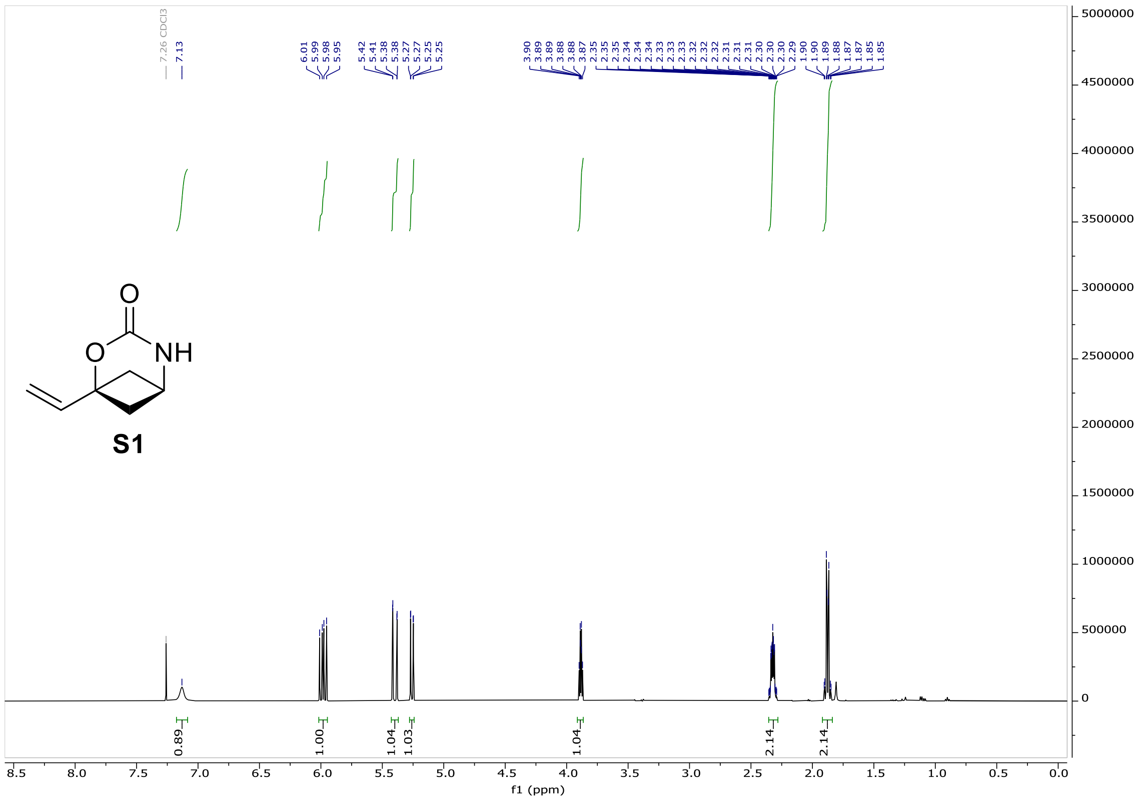

^{13}C NMR (**S1**, $CDCl_3$, 126 MHz)

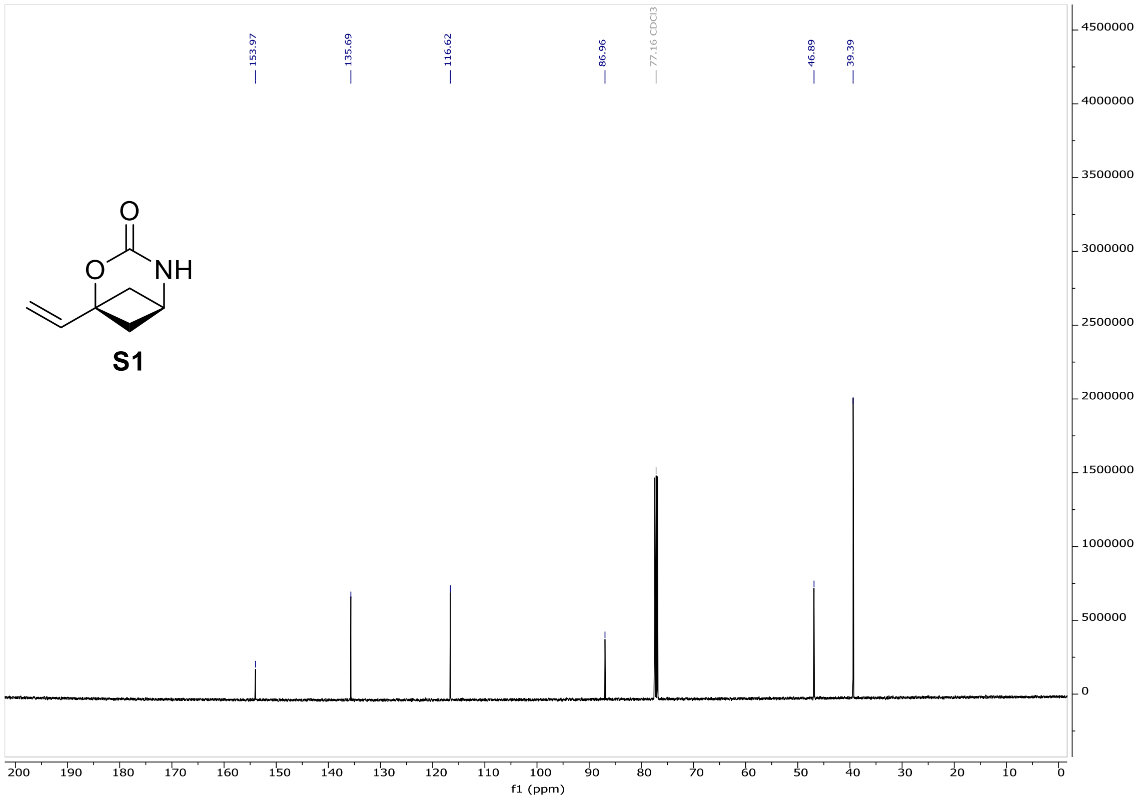

^{1}H NMR (**3a**, $CDCl_3$, 400 MHz)

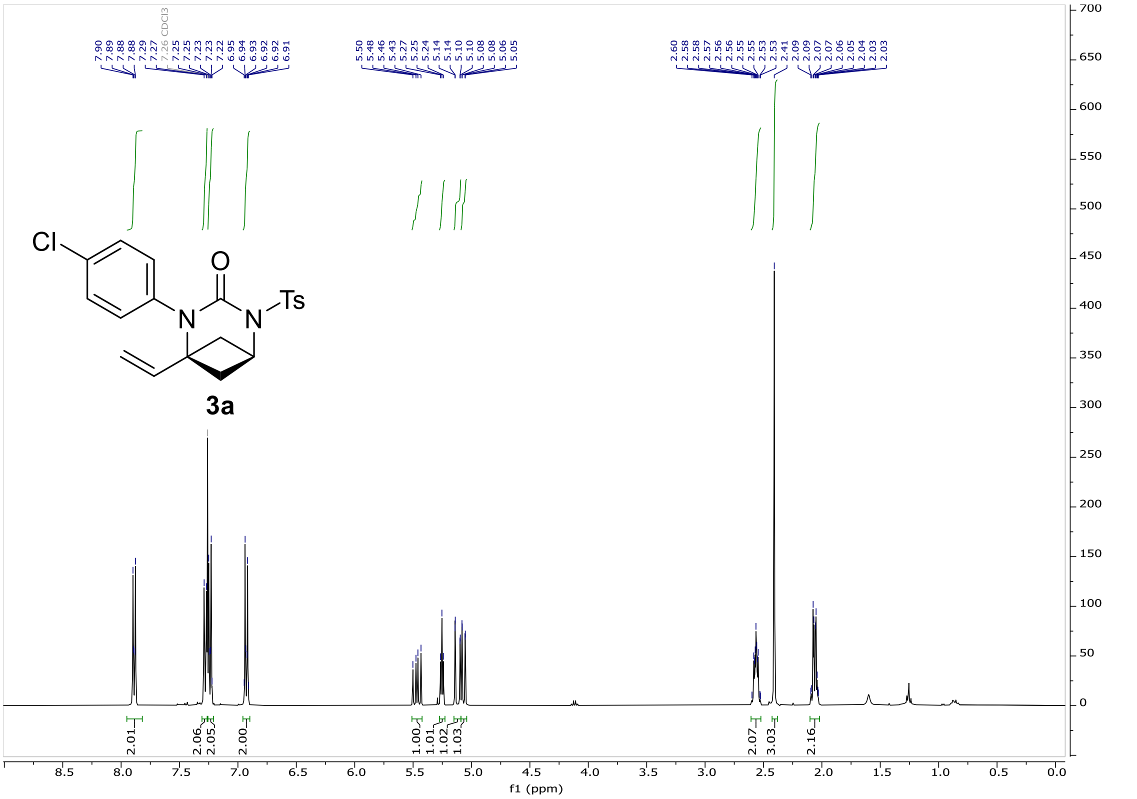

^{13}C NMR (**3a**, $CDCl_3$, 126 MHz)

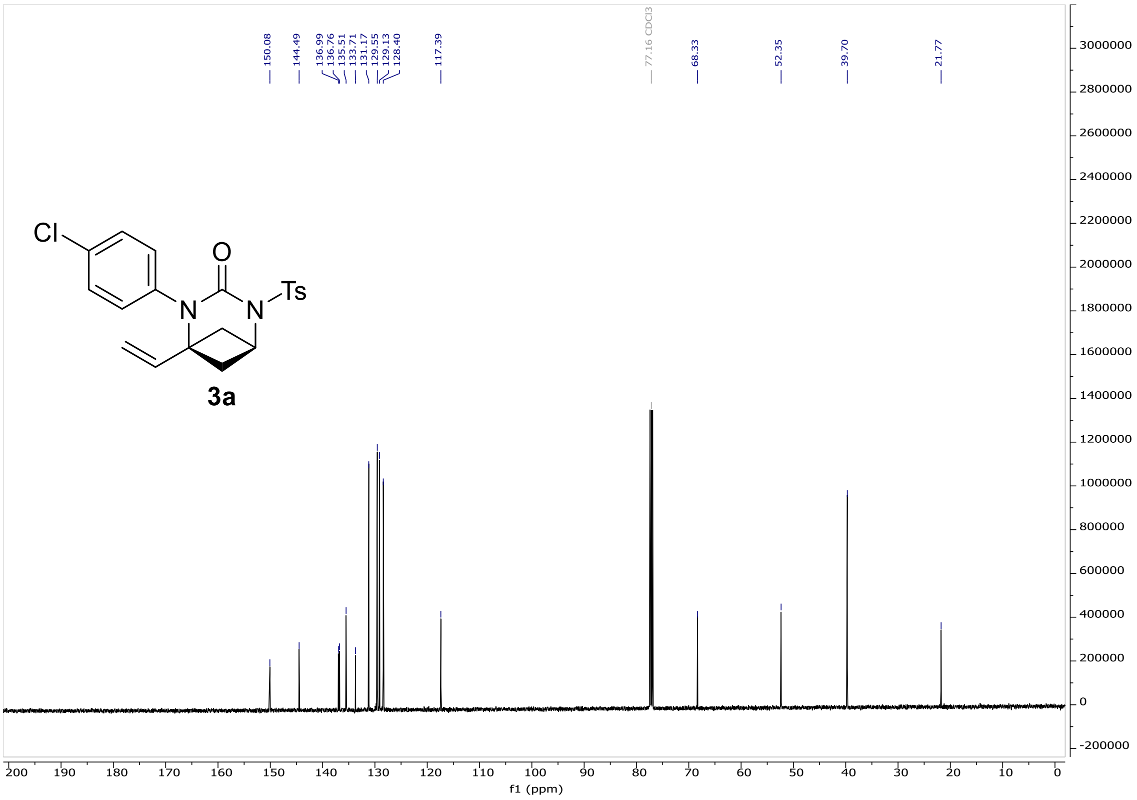

^{1}H NMR (**3b**, $CDCl_3$, 400 MHz)

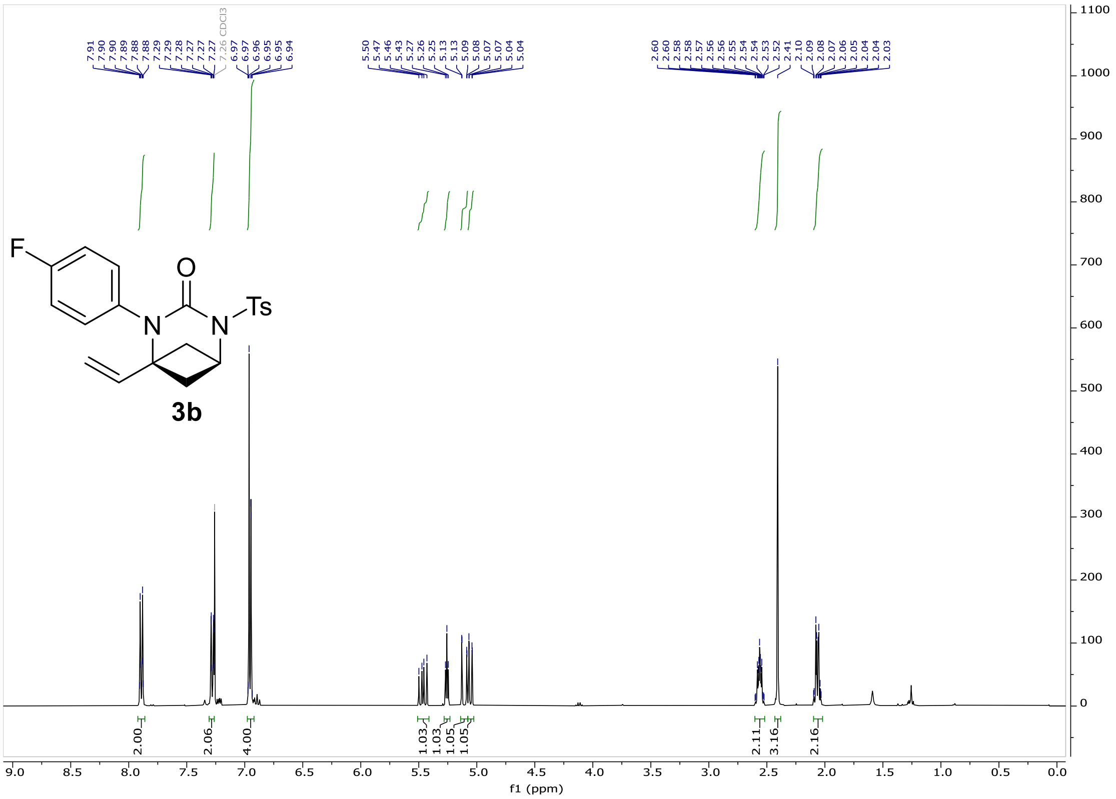

^{13}C NMR (**3b**, $CDCl_3$, 101 MHz)

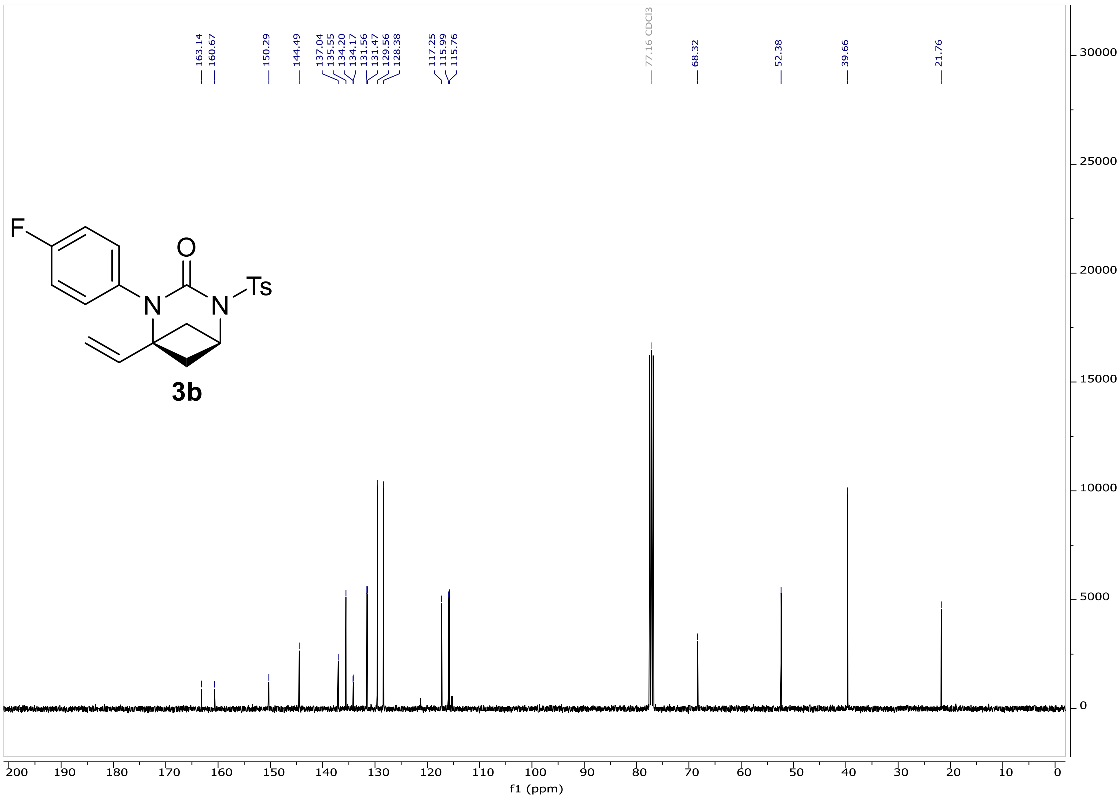

^{19}F NMR (**3b**, $CDCl_3$, 376 MHz)

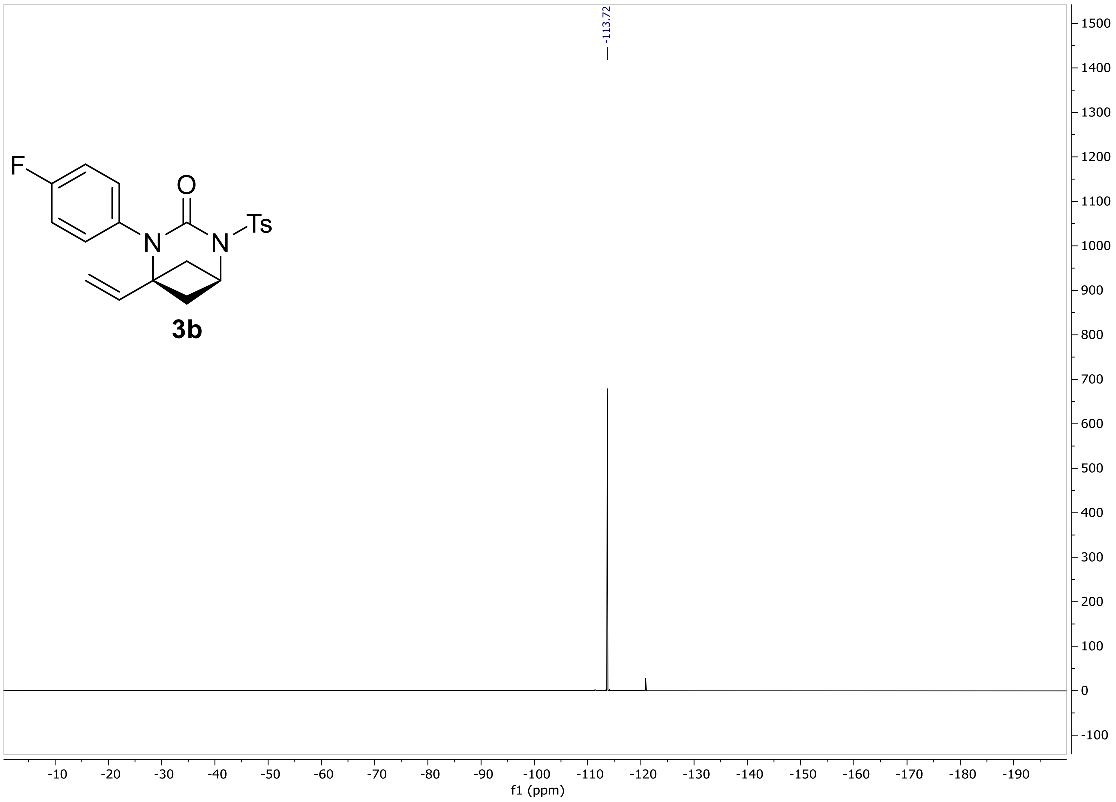

^{1}H NMR (**3c**, CDCl3, 400 MHz)

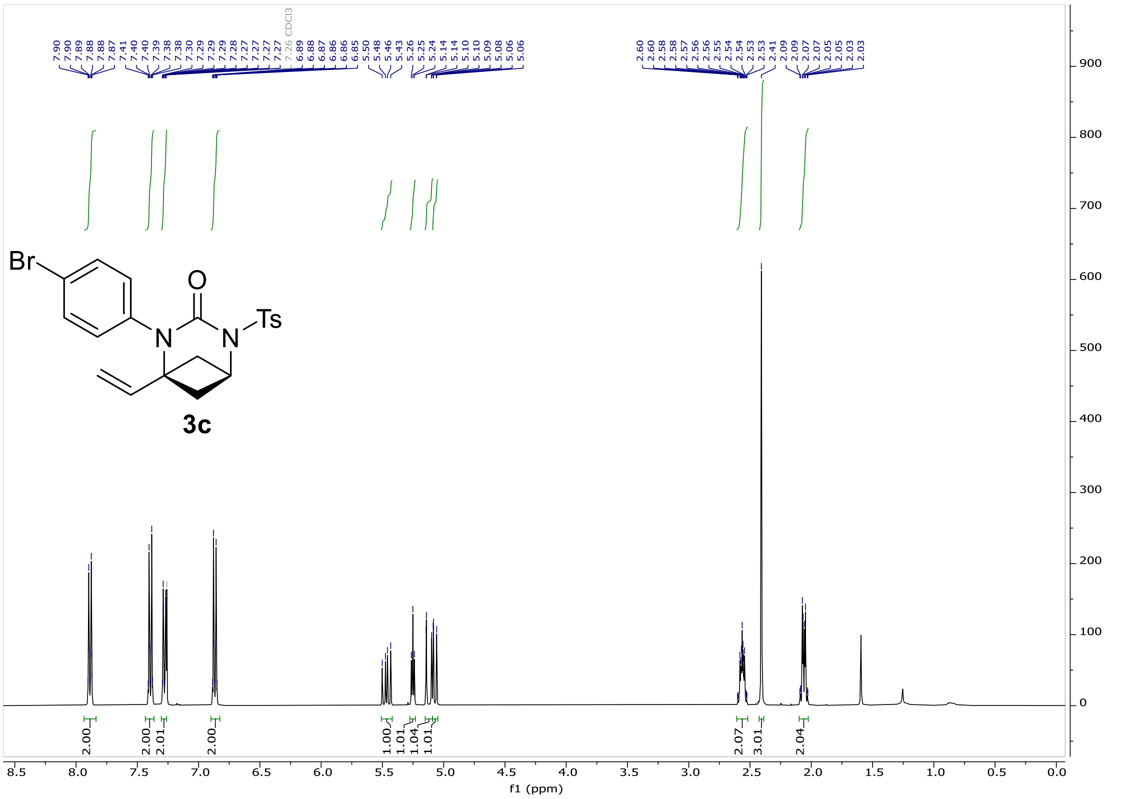

^{13}C NMR (**3c**, CDCl3, 126 MHz)

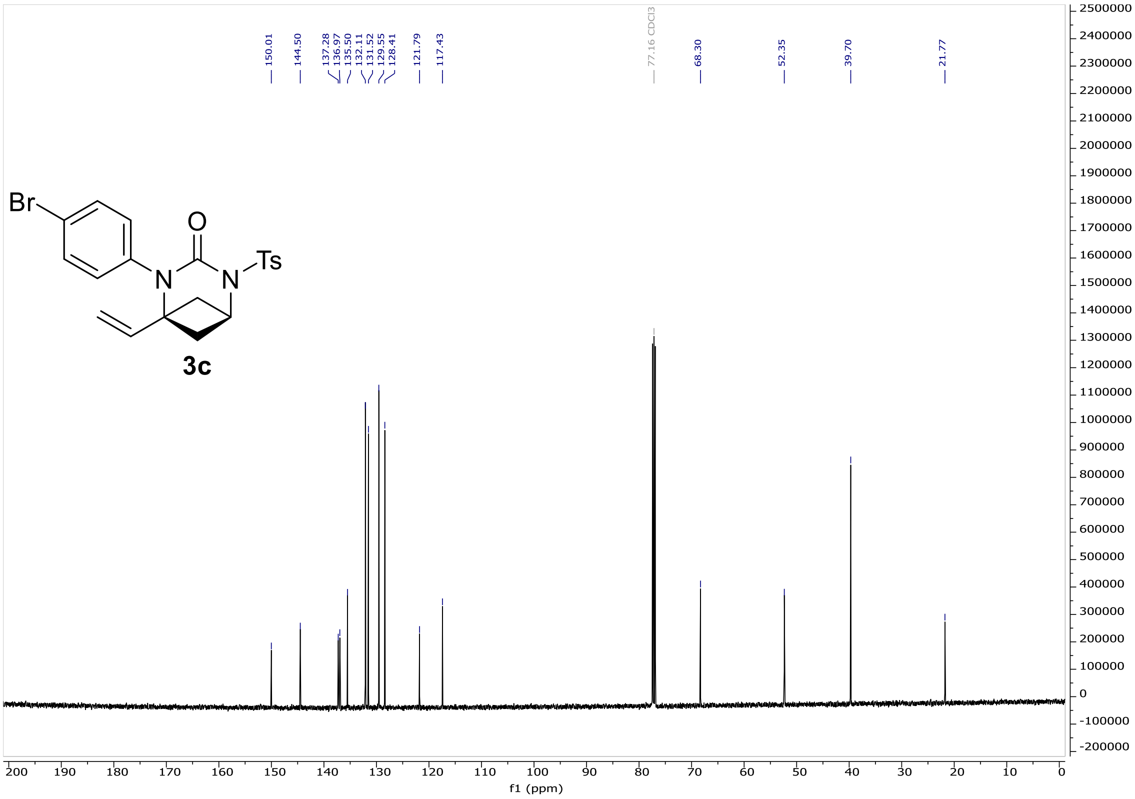

^{1}H NMR (**3d**, $CDCl_3$, 500 MHz)

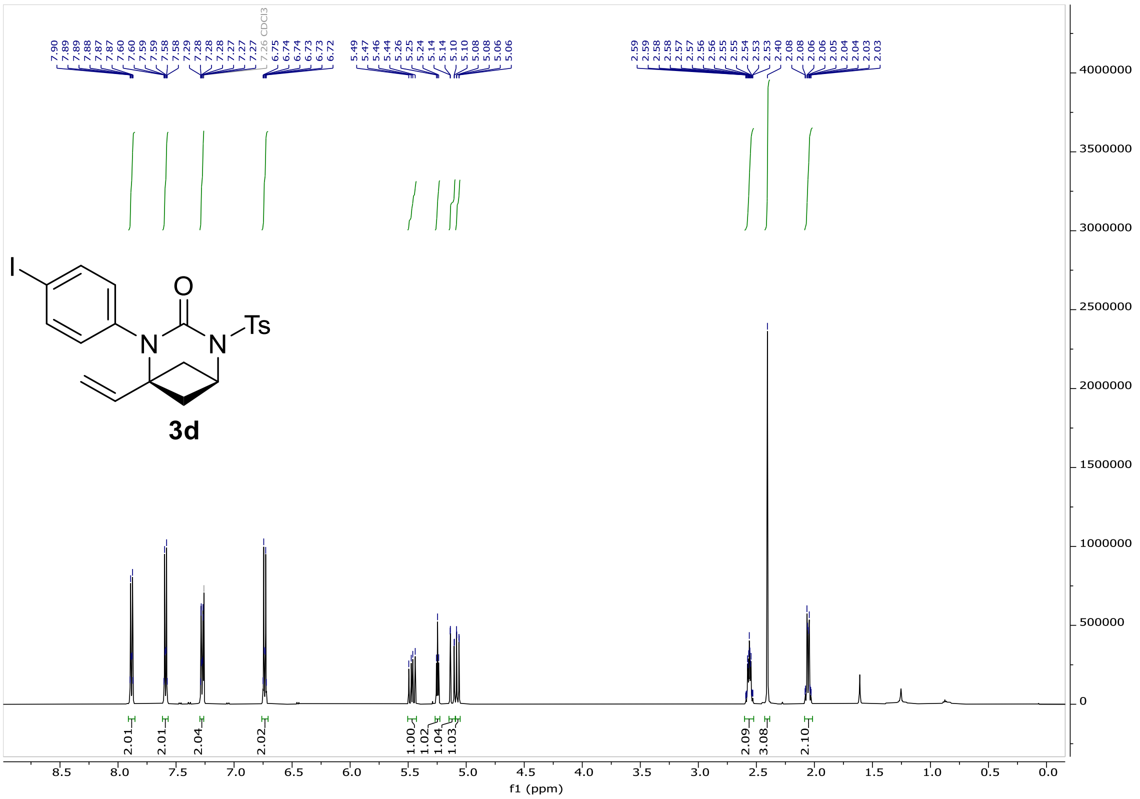

^{13}C NMR (**3d**, $CDCl_3$, 126 MHz)

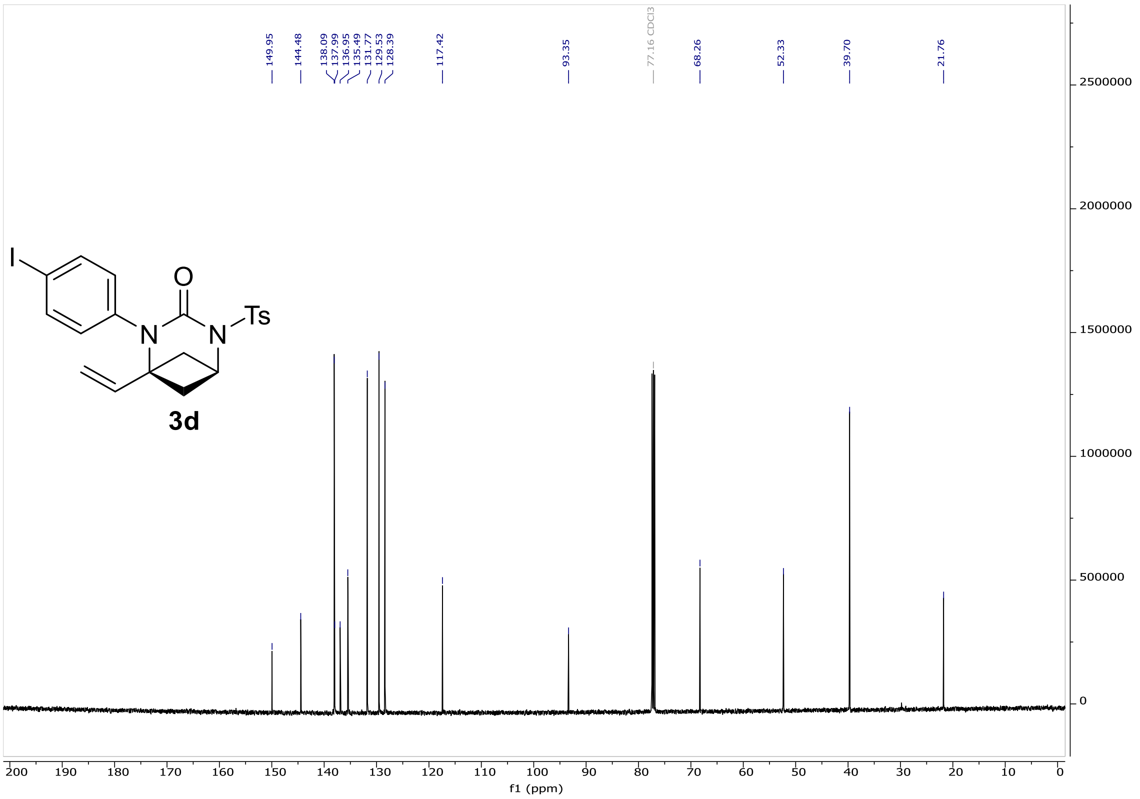

^{1}H NMR (**3e**, $CDCl_3$, 500 MHz)

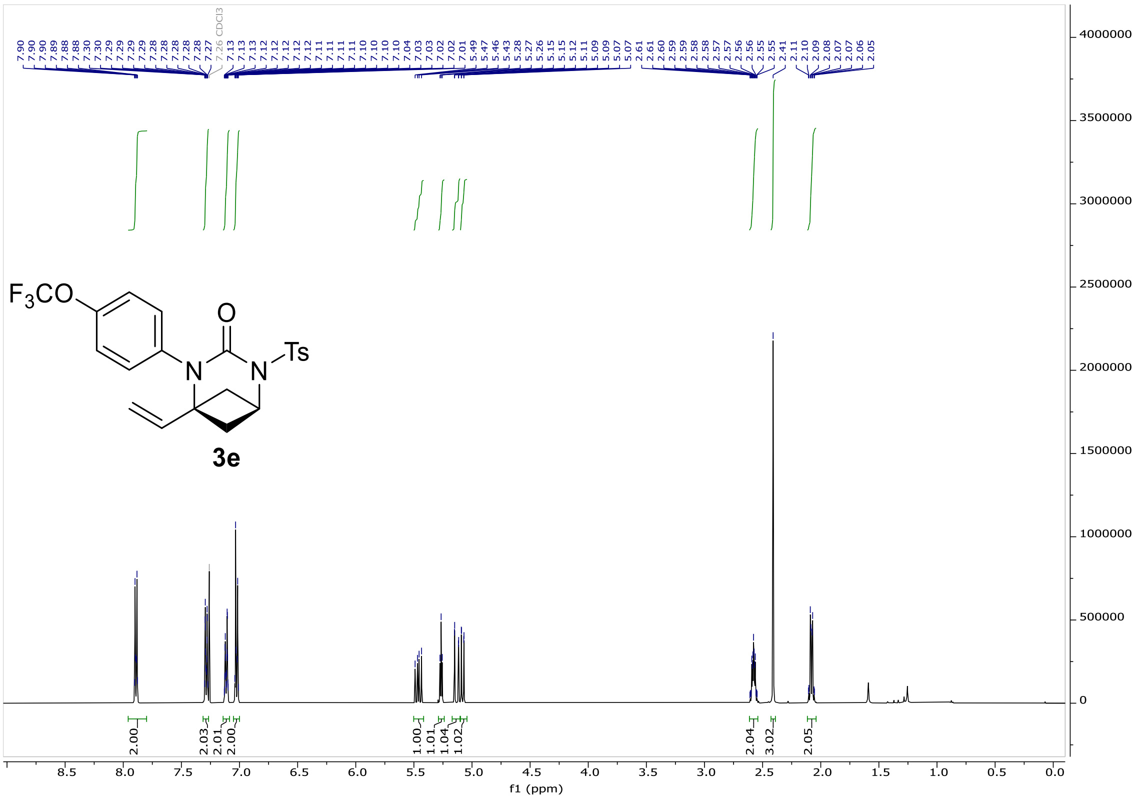

^{13}C NMR (**3e**, $CDCl_3$, 126 MHz)

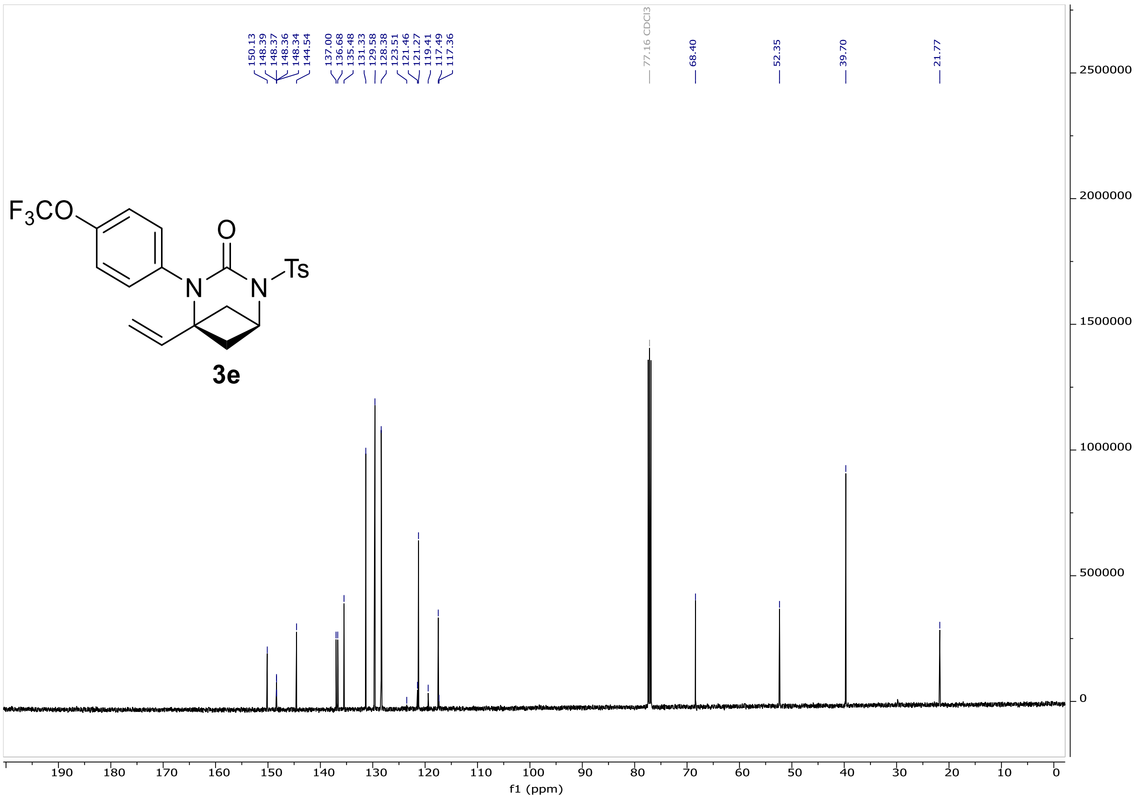

^{19}F NMR (**3e**, $CDCl_3$, 376 MHz)

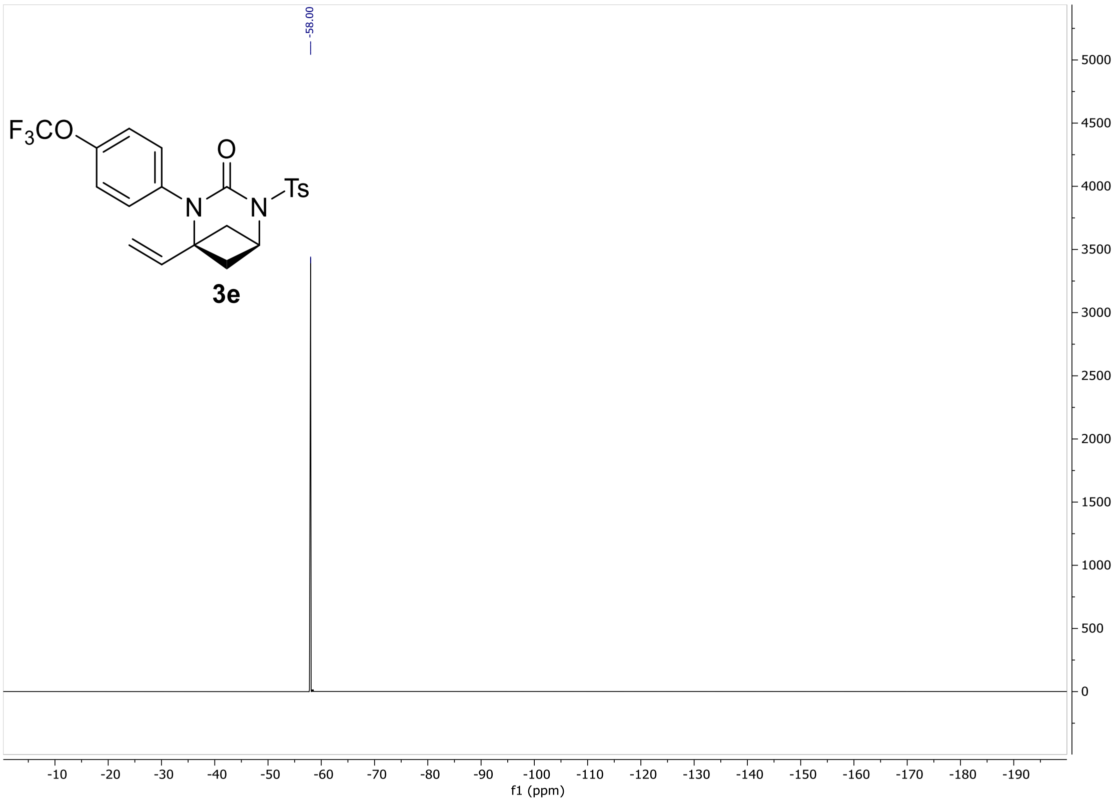

^{1}H NMR (**3f**, $CDCl_3$, 400 MHz)

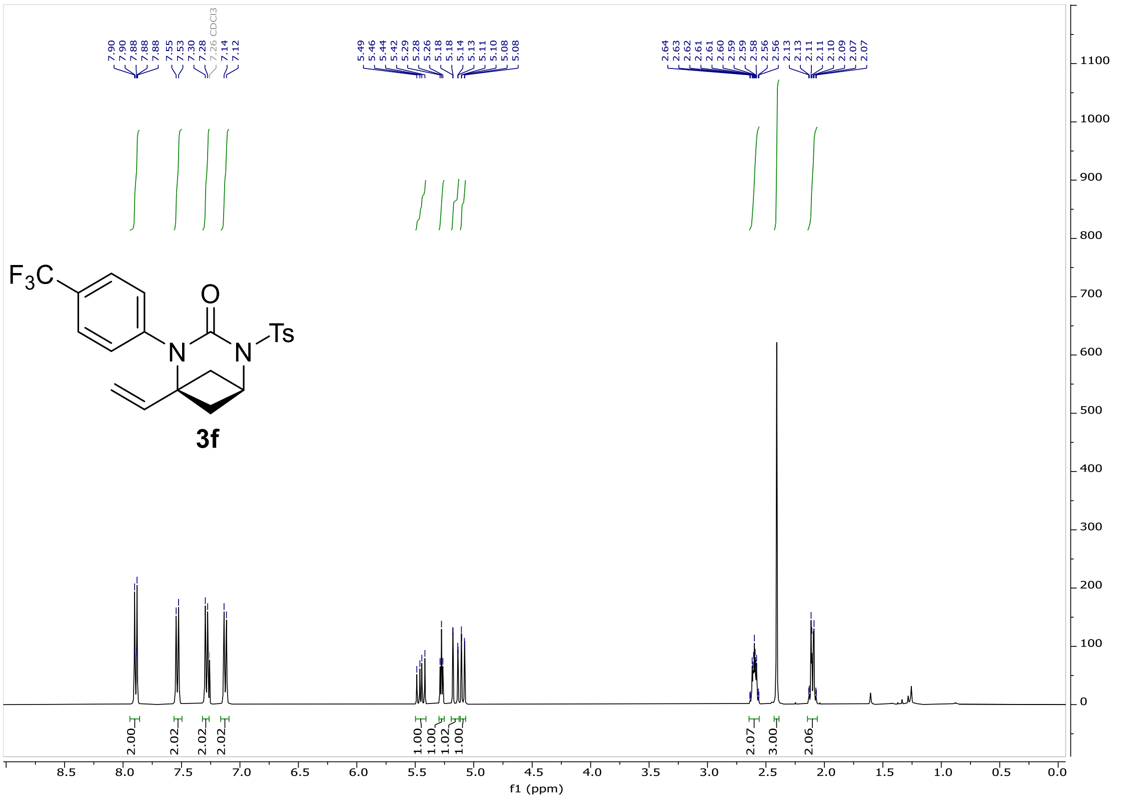

^{13}C NMR (**3f**, $CDCl_3$, 101 MHz)

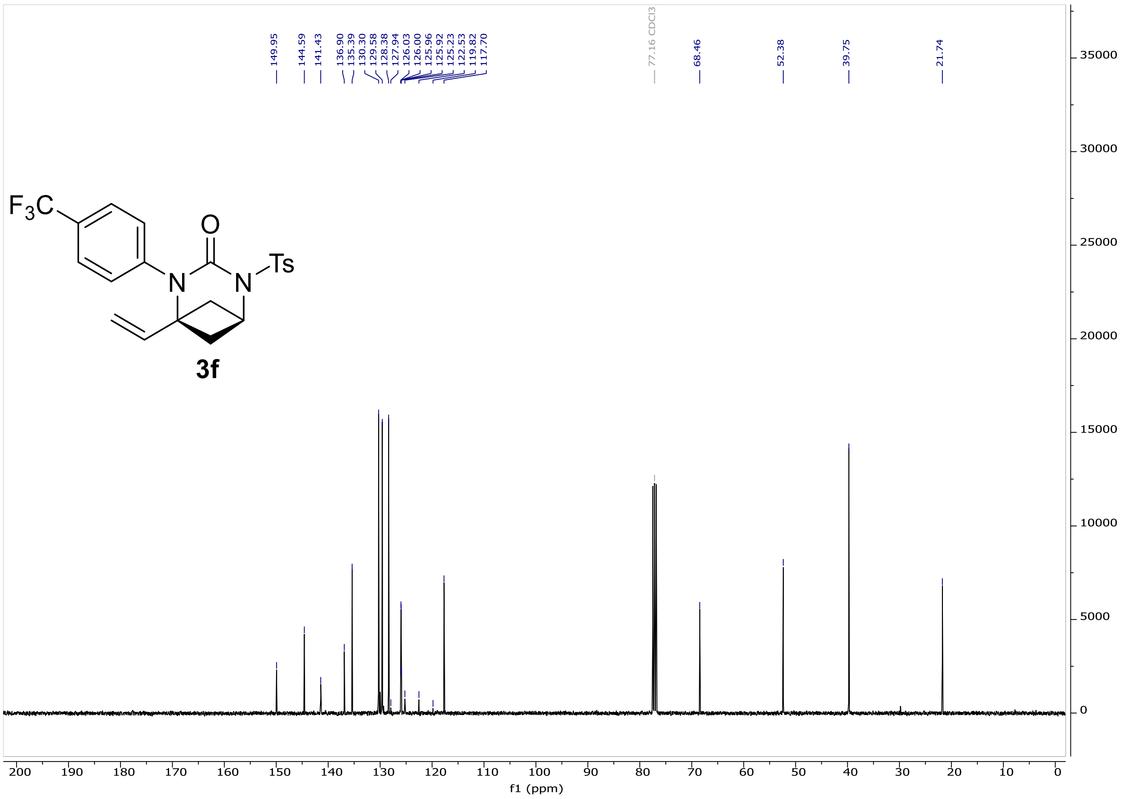

^{19}F NMR (**3f**, $CDCl_3$, 376 MHz)

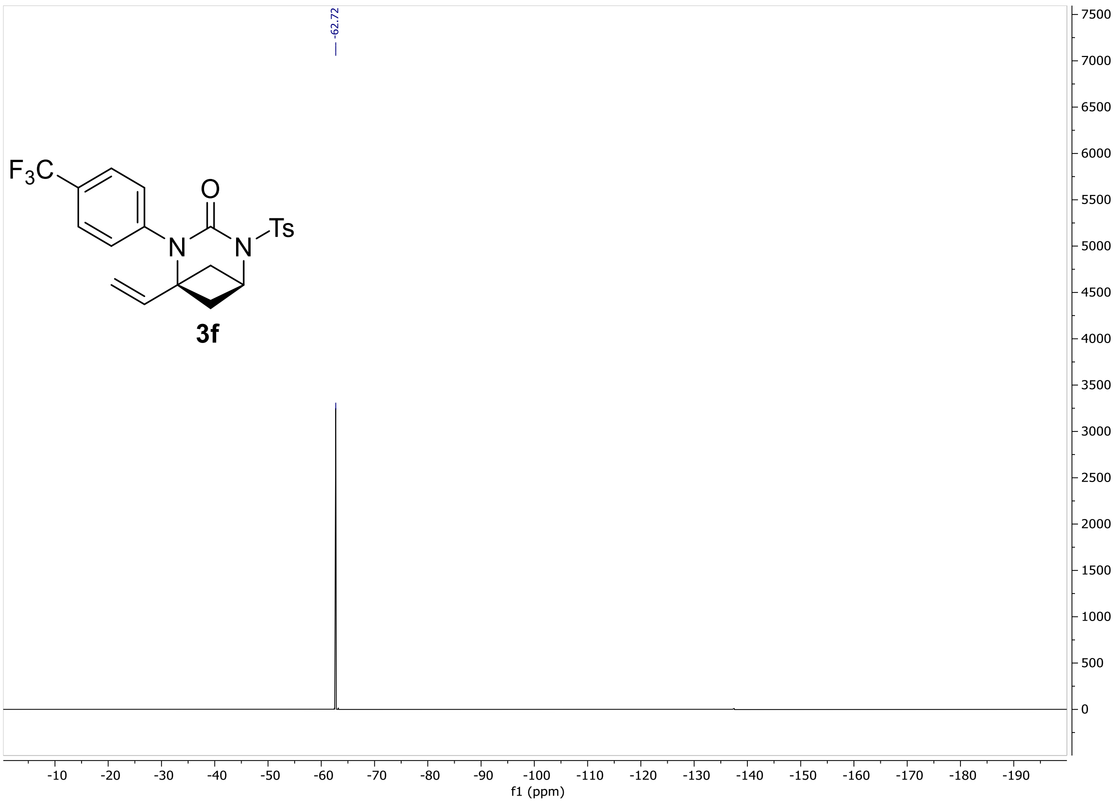

^{1}H NMR (**3g**, $CDCl_3$, 400 MHz)

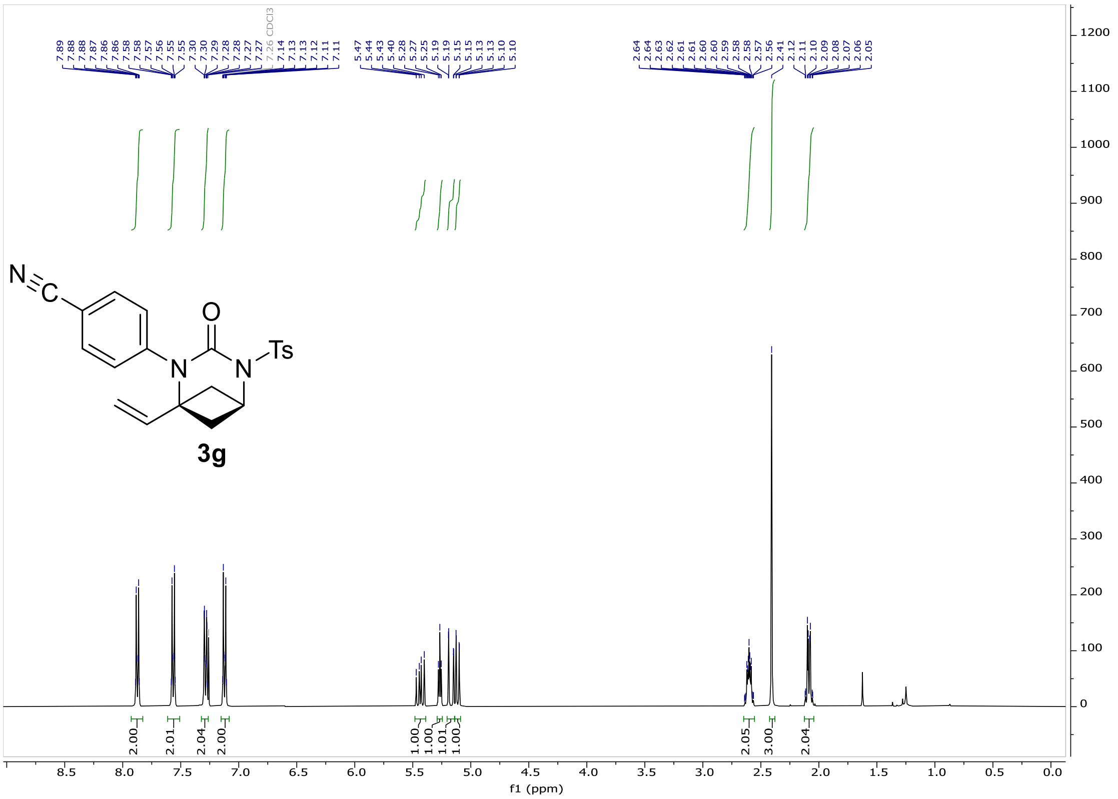

^{13}C NMR (**3g**, $CDCl_3$, 101 MHz)

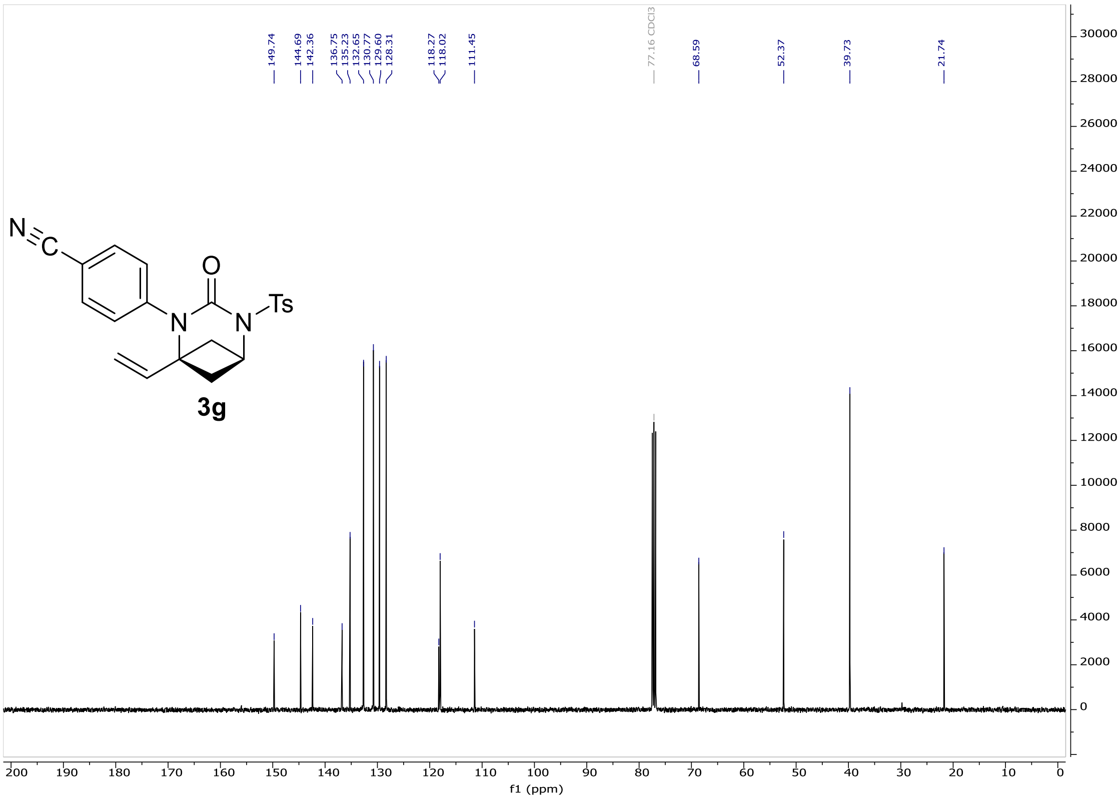

^{1}H NMR (**3h**, $CDCl_3$, 400 MHz)

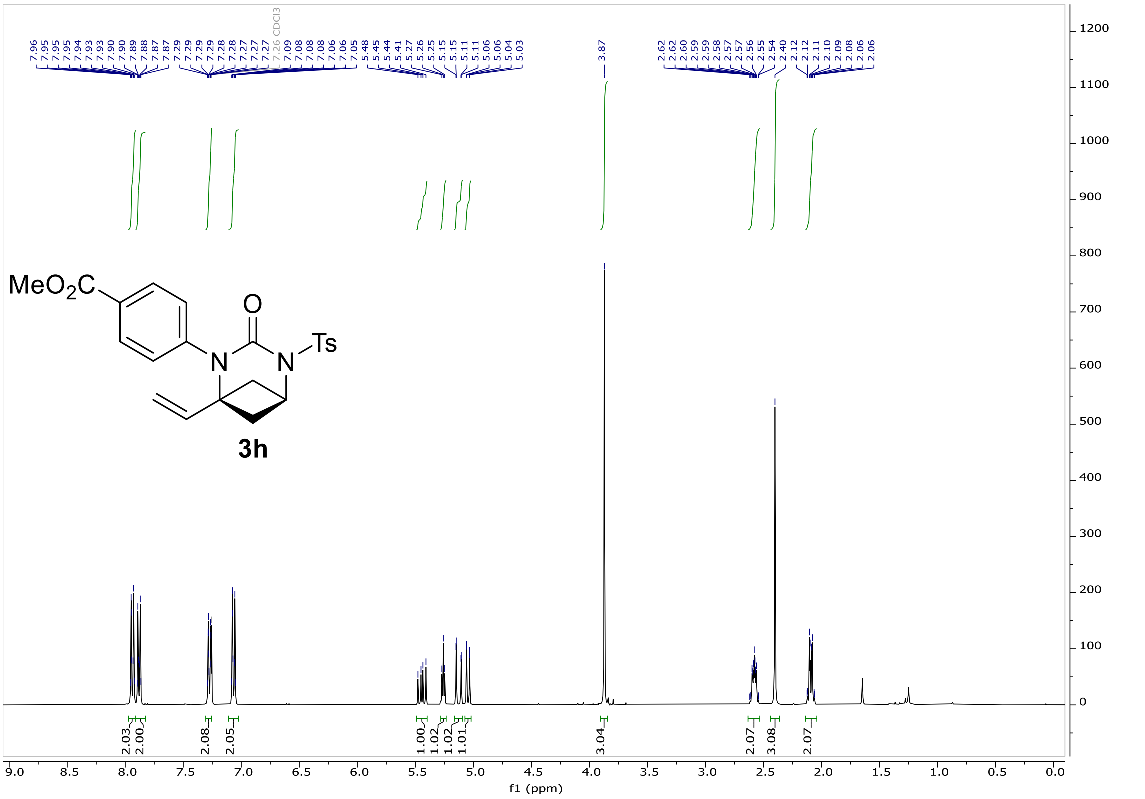

^{13}C NMR (**3h**, $CDCl_3$, 126 MHz)

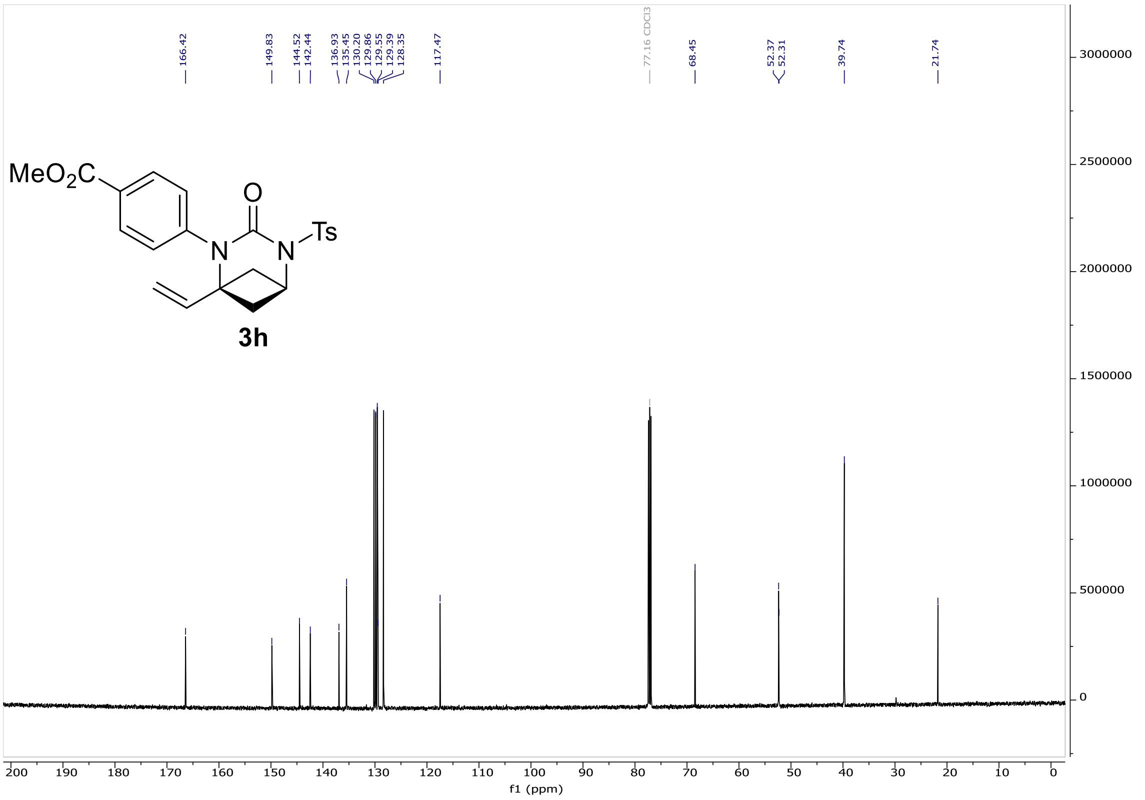

^{1}H NMR (**3i**, $CDCl_3$, 500 MHz)

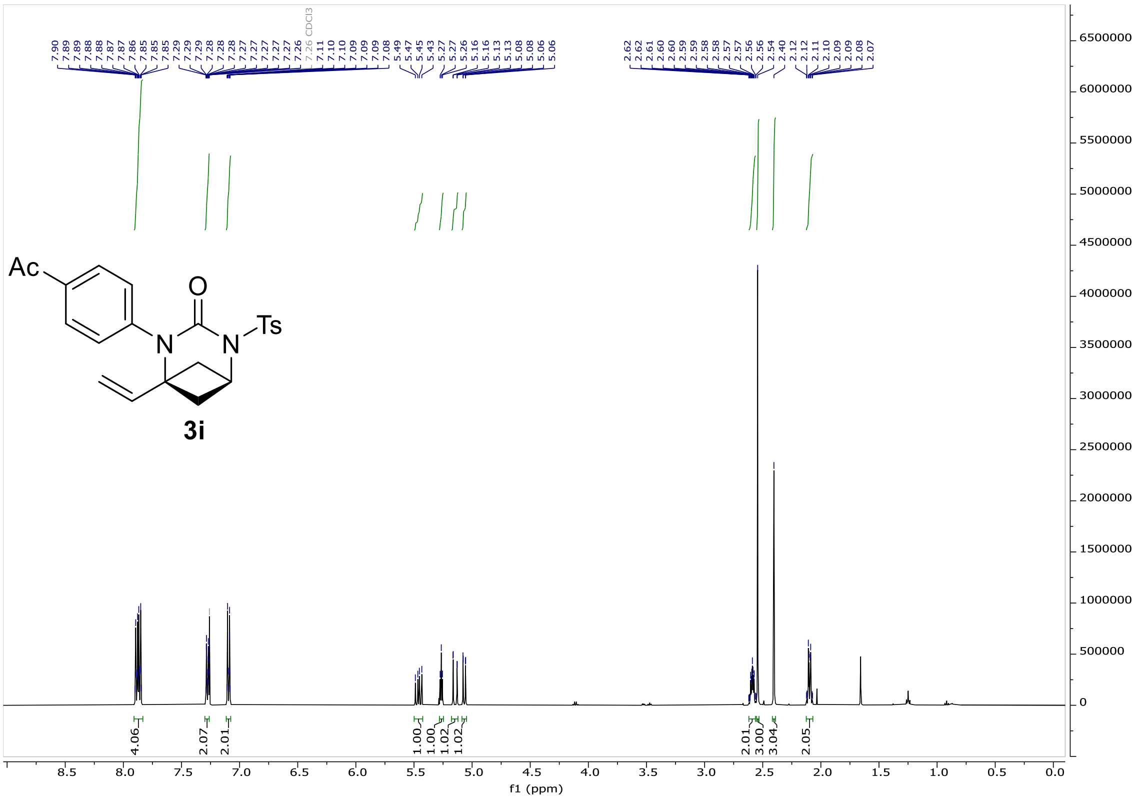

^{13}C NMR (**3i**, $CDCl_3$, 101 MHz)

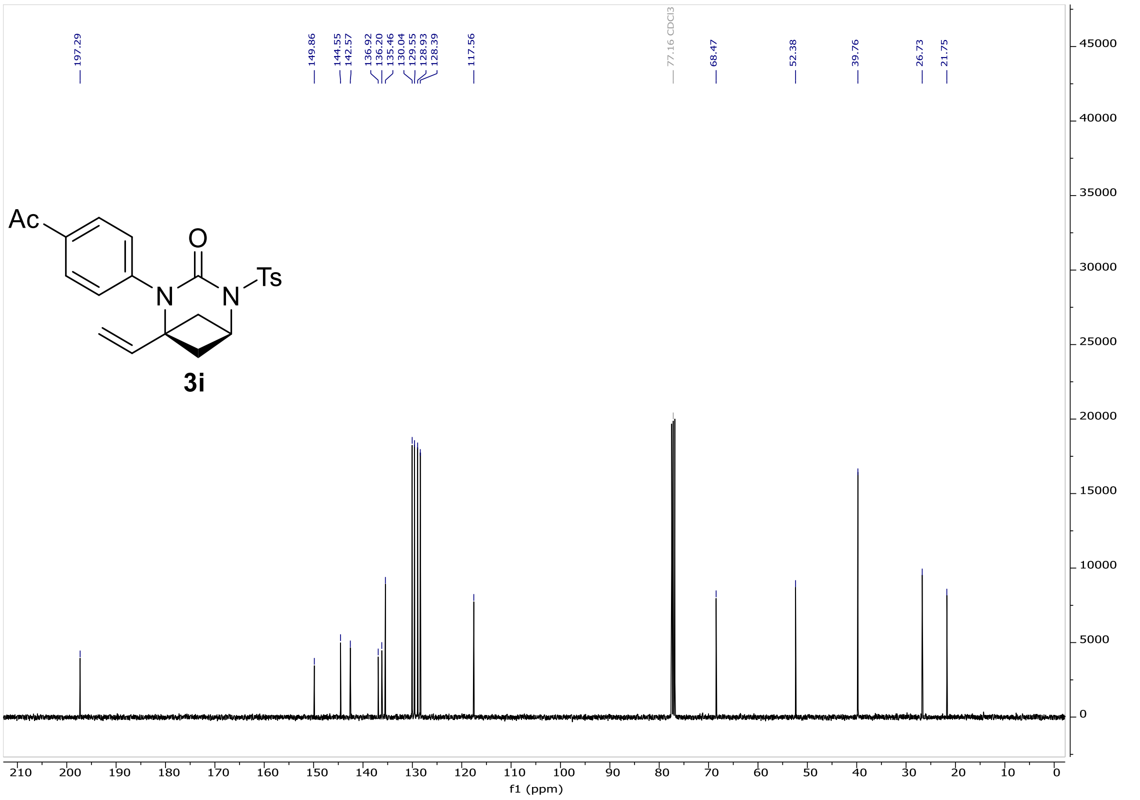

^{1}H NMR (**3j**, CDCl$_3$, 400 MHz)

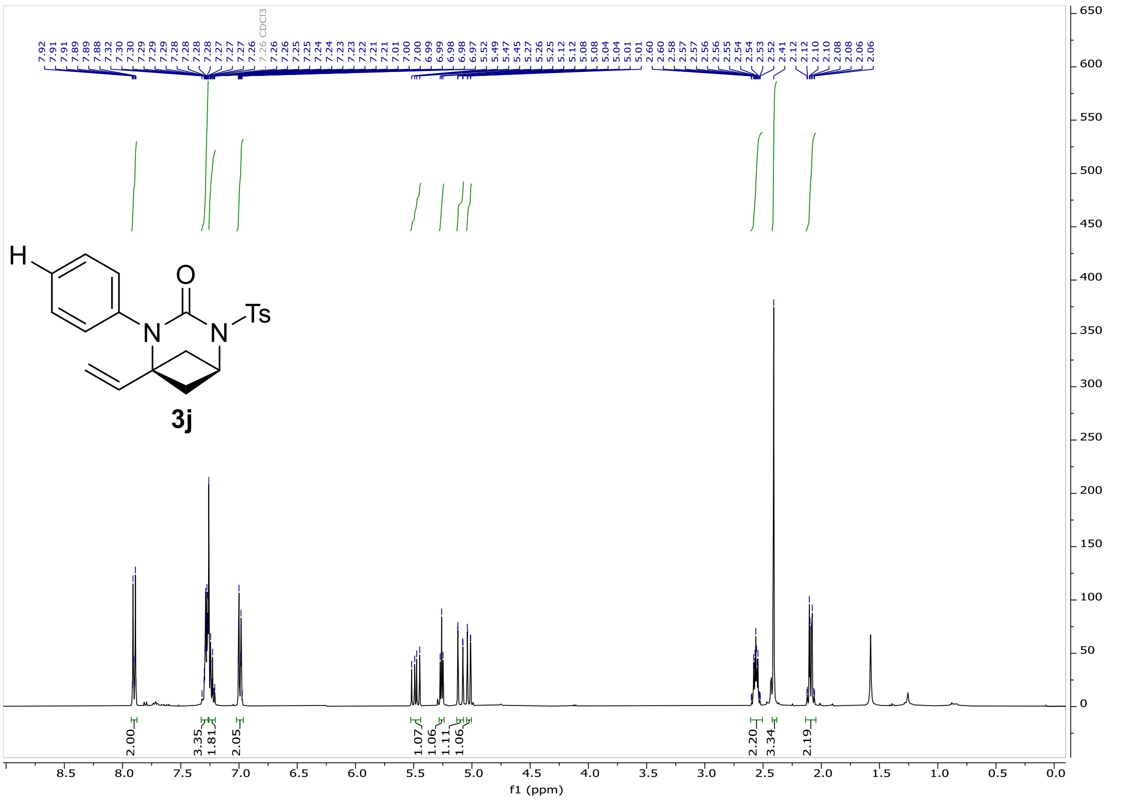

^{13}C NMR (**3j**, CDCl$_3$, 101 MHz)

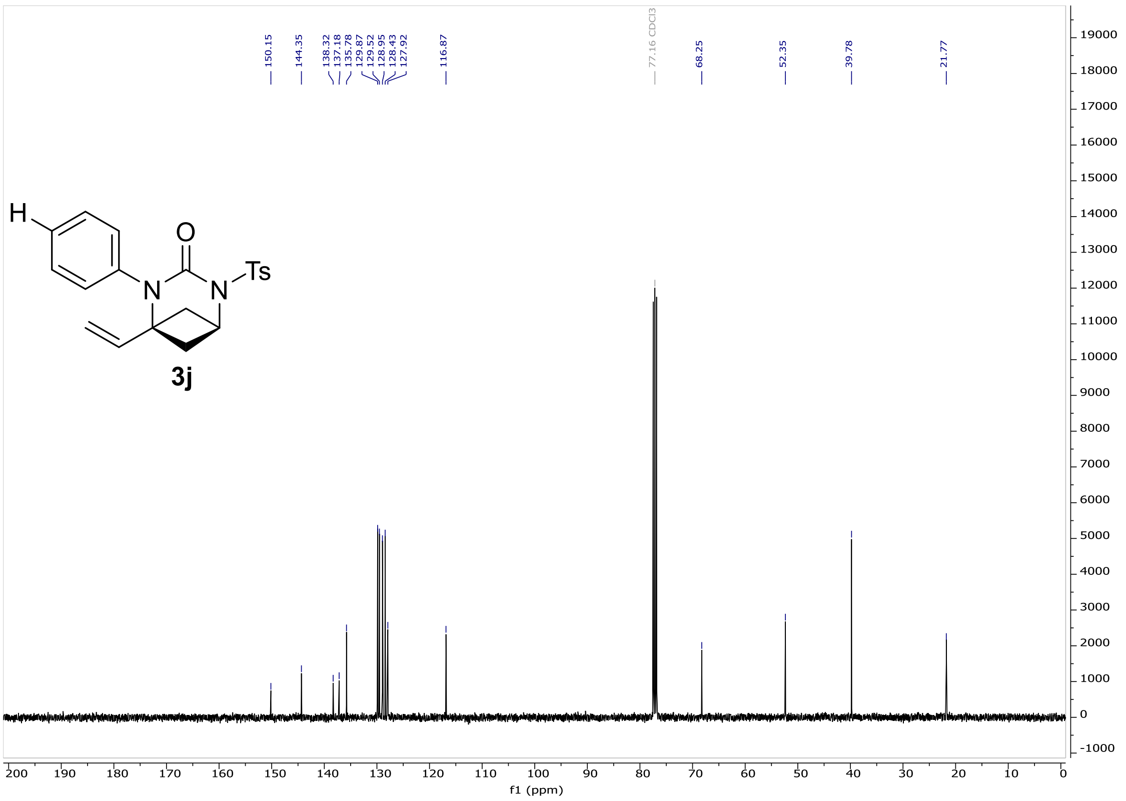

^{1}H NMR (**3k**, $CDCl_3$, 400 MHz)

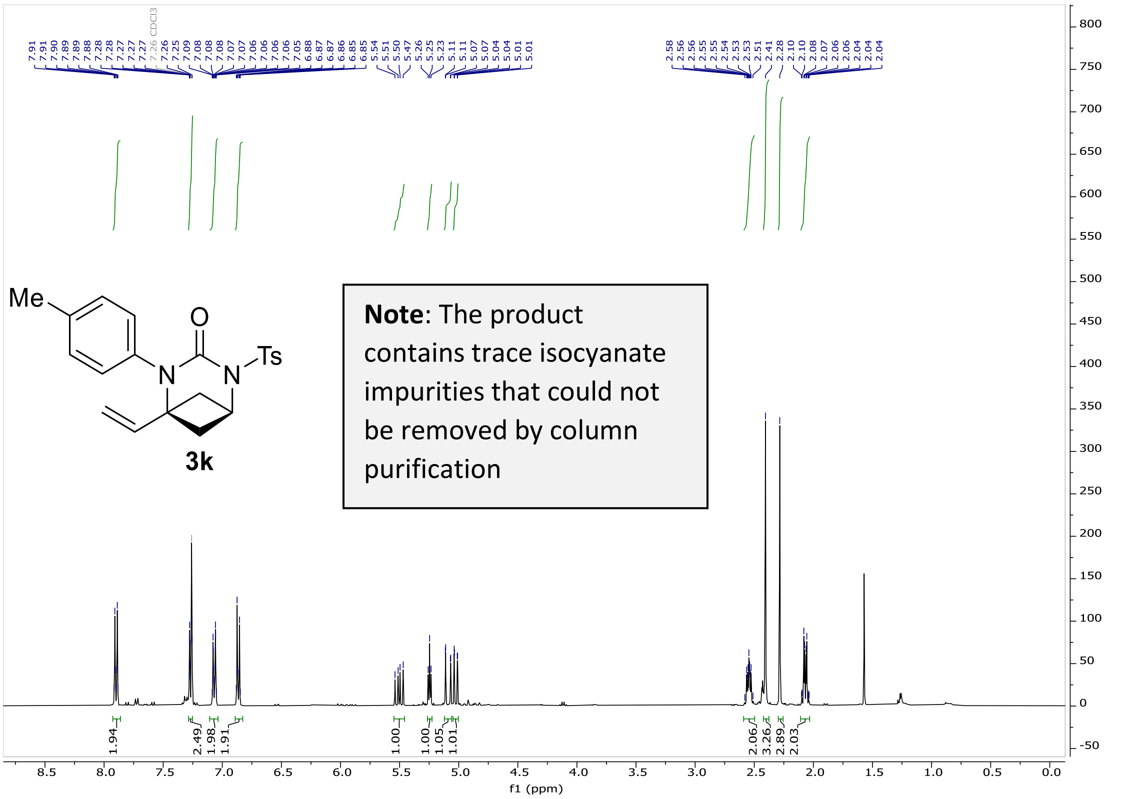

^{13}C NMR (**3k**, $CDCl_3$, 101 MHz)

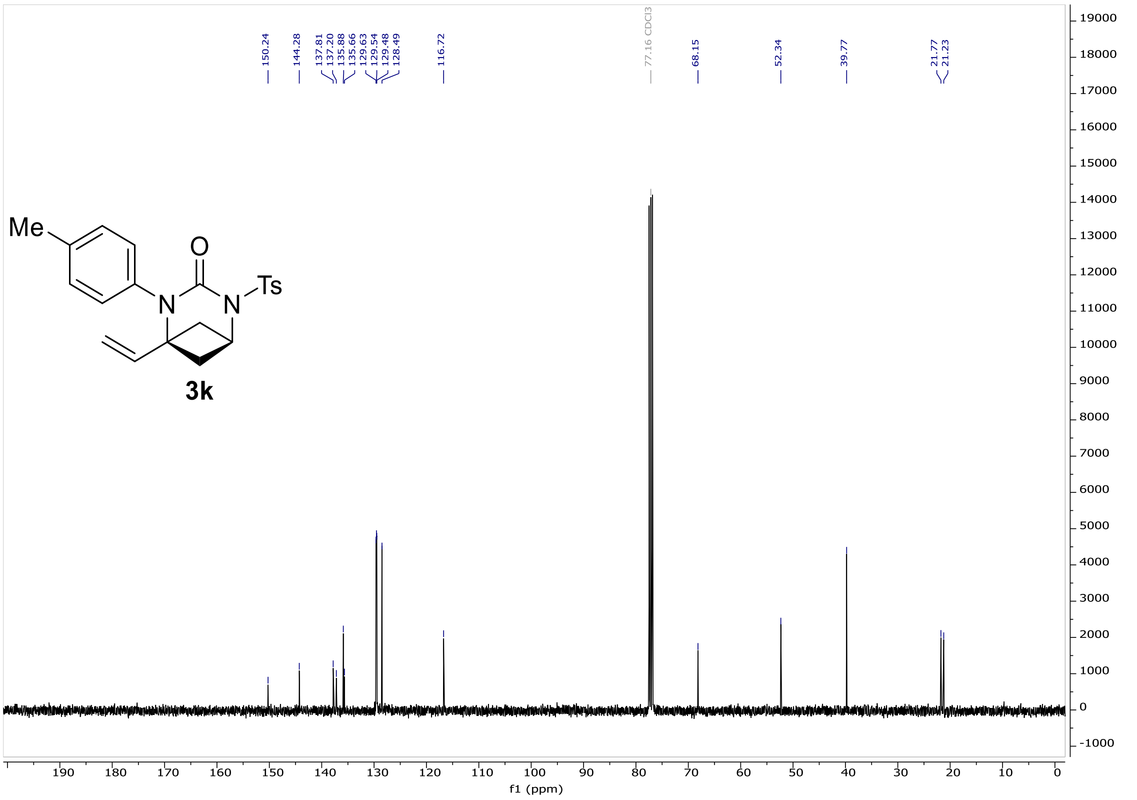

^{1}H NMR (**3l**, $CDCl_3$, 400 MHz)

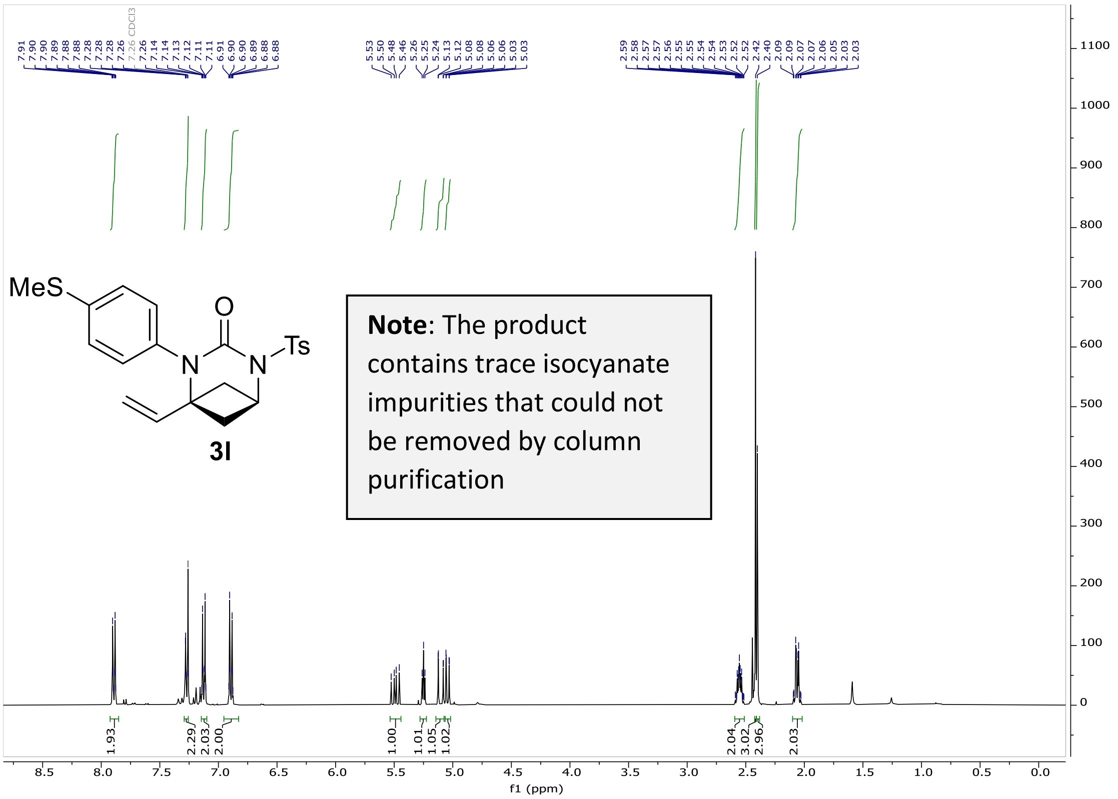

^{13}C NMR (**3l**, $CDCl_3$, 101 MHz)

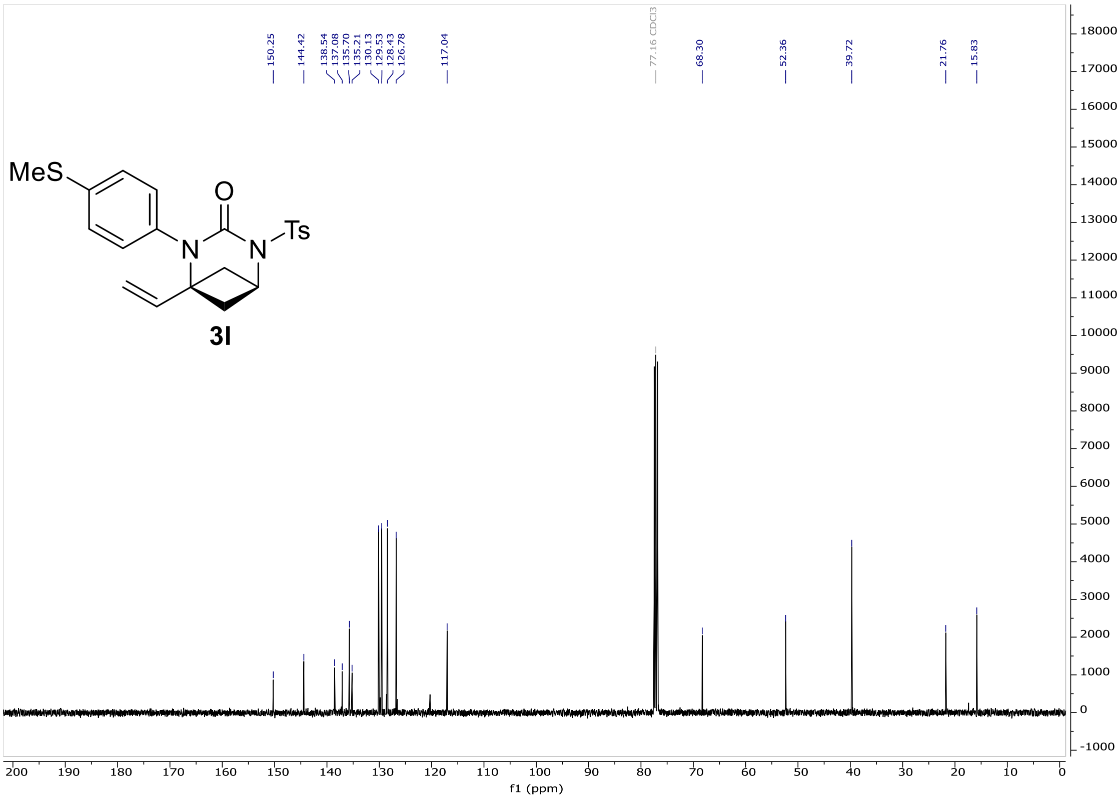

^{1}H NMR (**3m**, $CDCl_3$, 300 MHz)

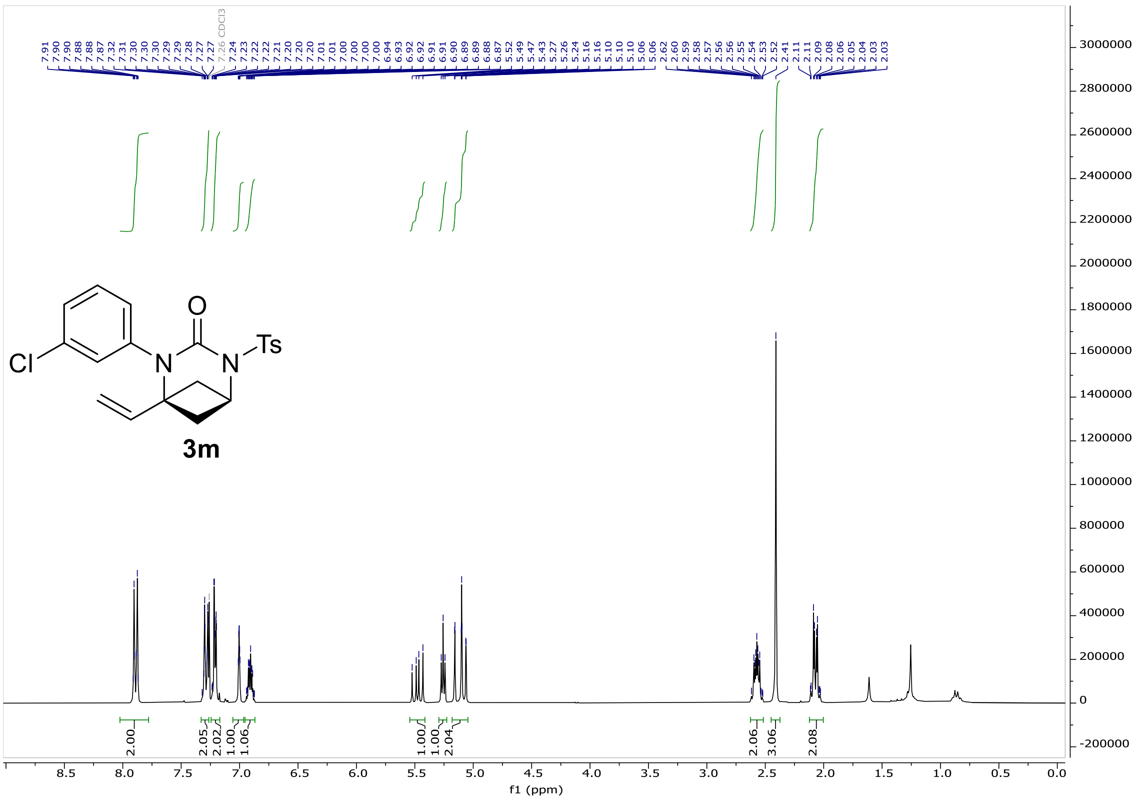

^{13}C NMR (**3m**, $CDCl_3$, 101 MHz)

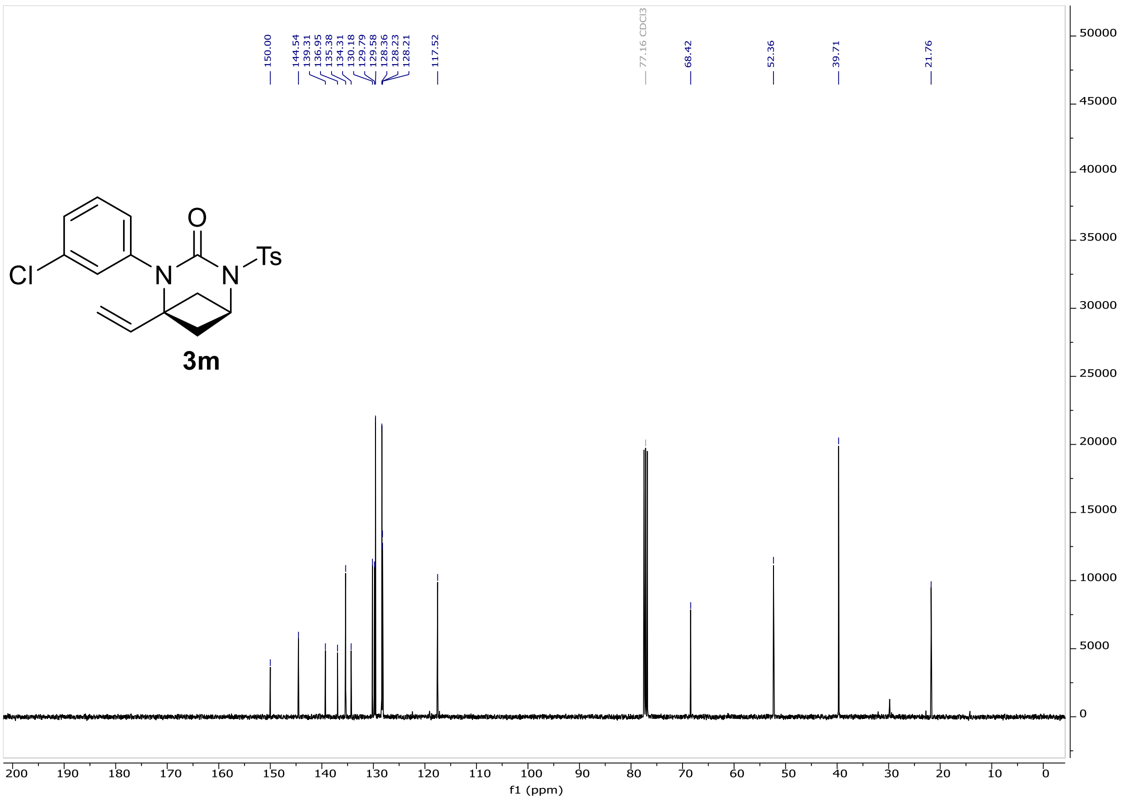

^{1}H NMR (**3n**, $CDCl_3$, 500 MHz)

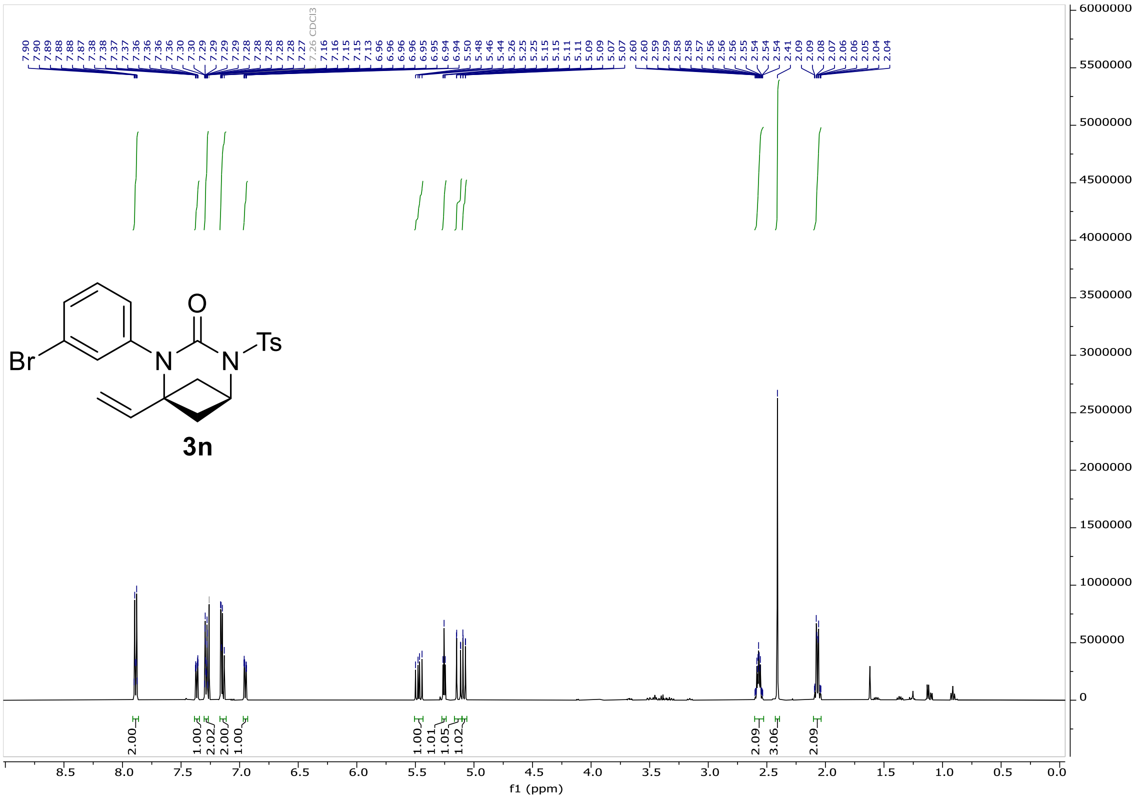

^{13}C NMR (**3n**, $CDCl_3$, 126 MHz)

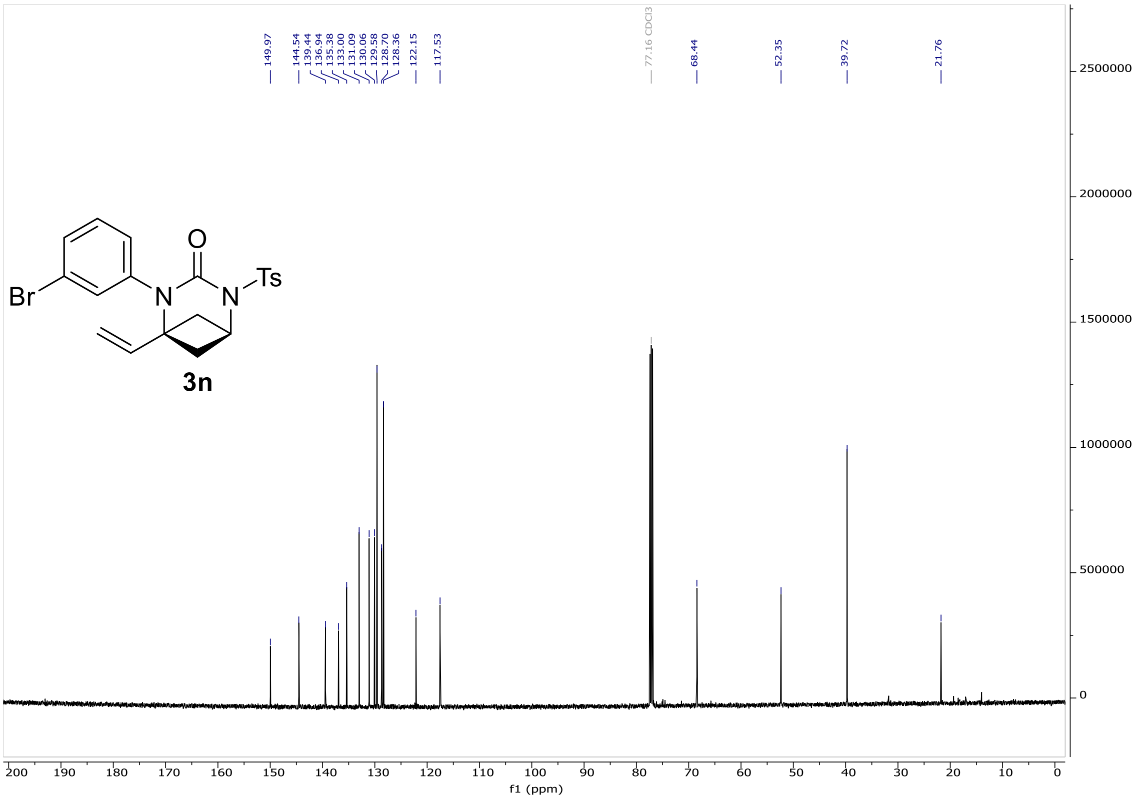

^{1}H NMR (**3o**, $CDCl_3$, 500 MHz)

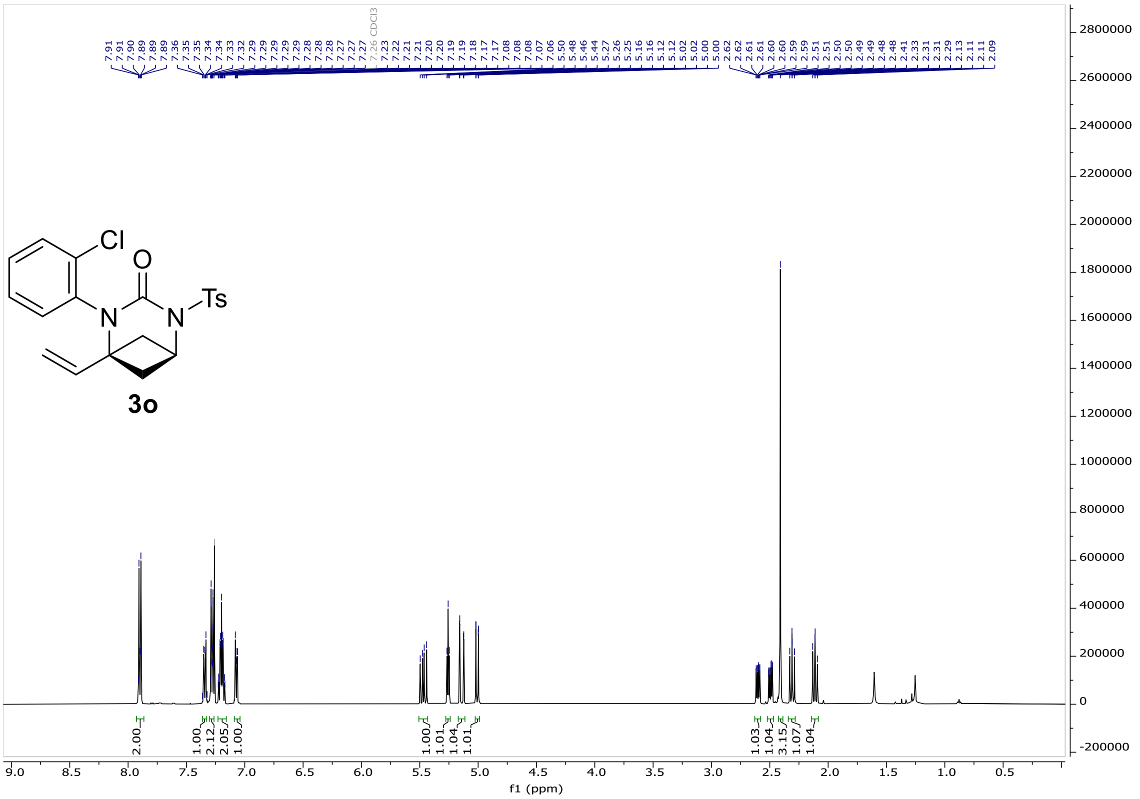

^{13}C NMR (**3o**, $CDCl_3$, 126 MHz)

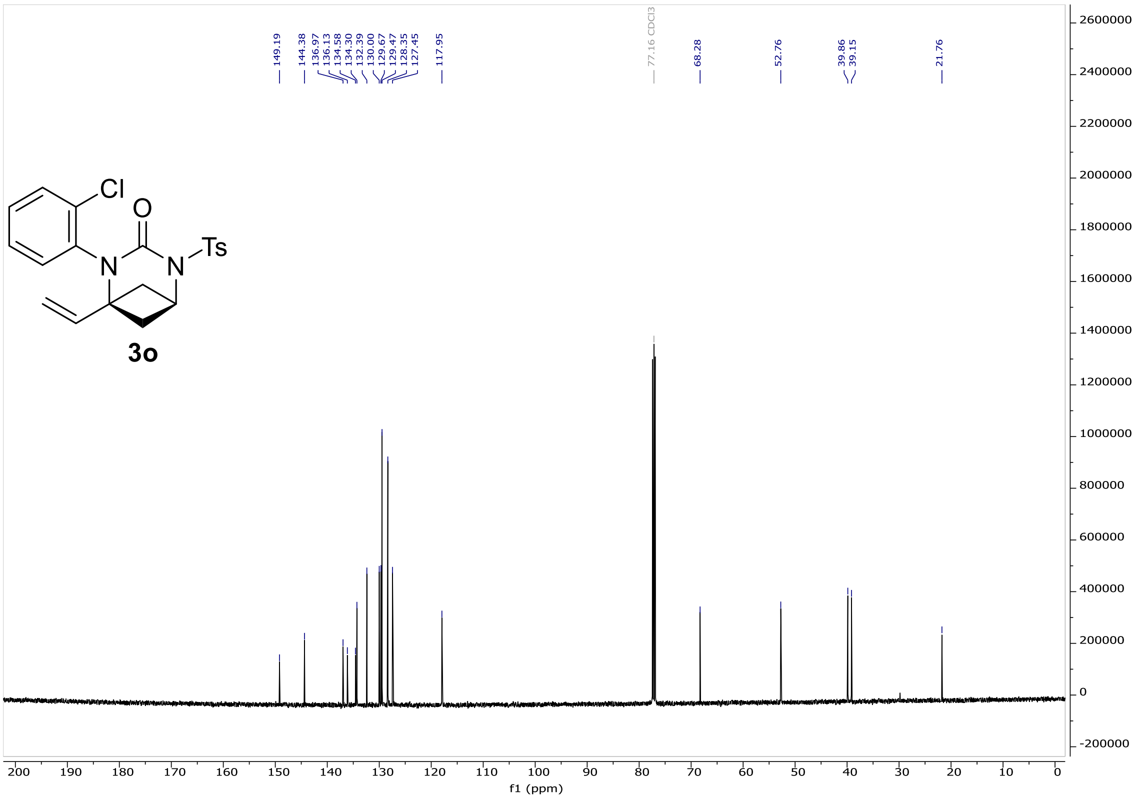

^{1}H NMR (**3p**, CDCl$_3$, 400 MHz)

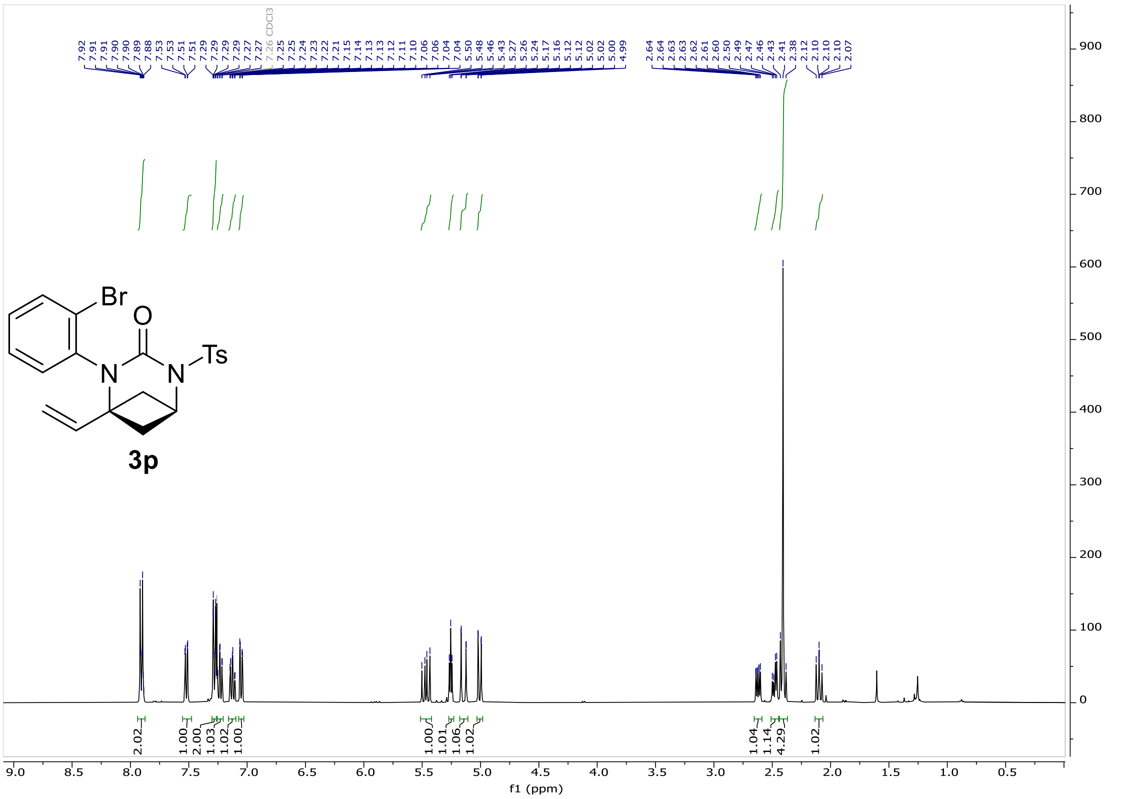

^{13}C NMR (**3p**, CDCl$_3$, 126 MHz)

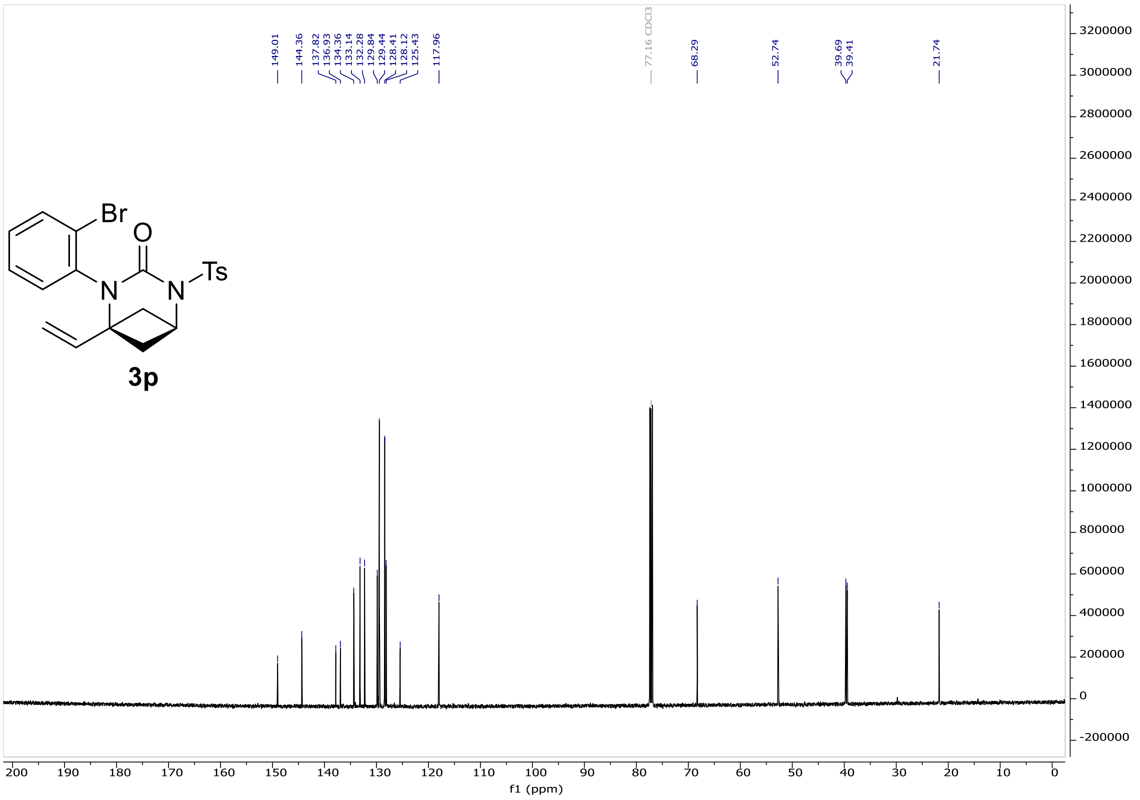

^{1}H NMR (**3q**, $CDCl_3$, 500 MHz)

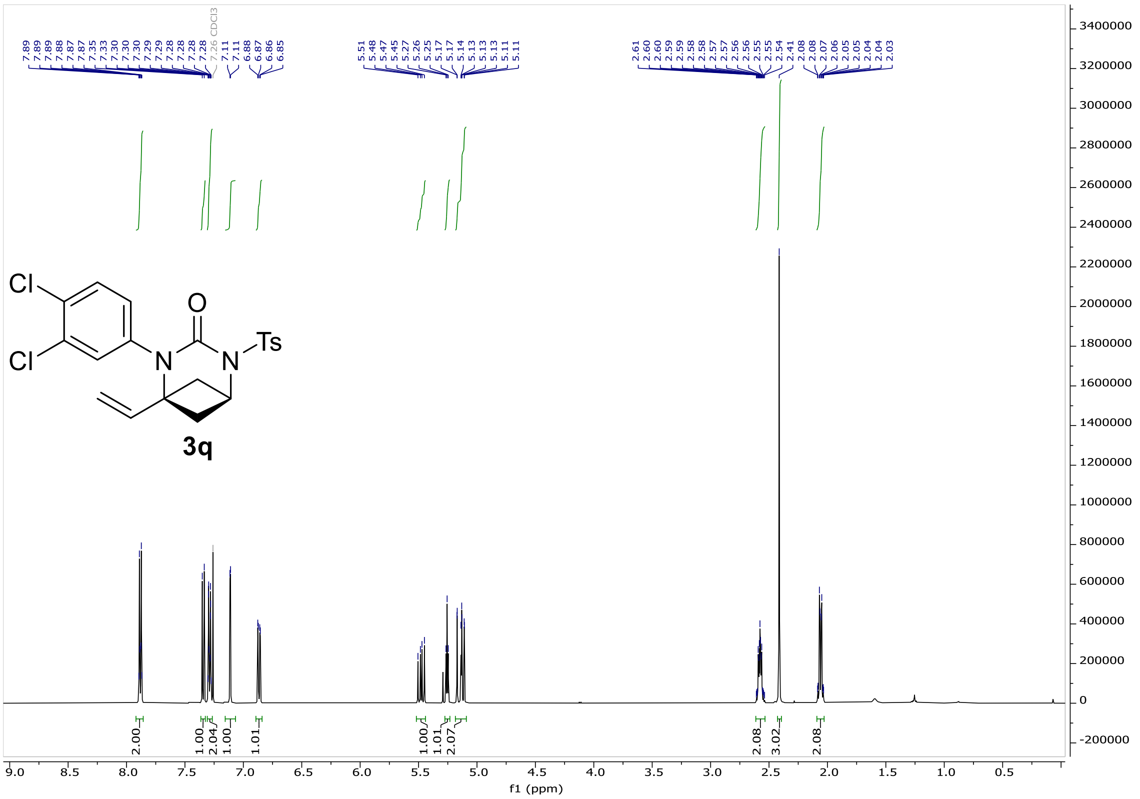

^{13}C NMR (**3q**, $CDCl_3$, 126 MHz)

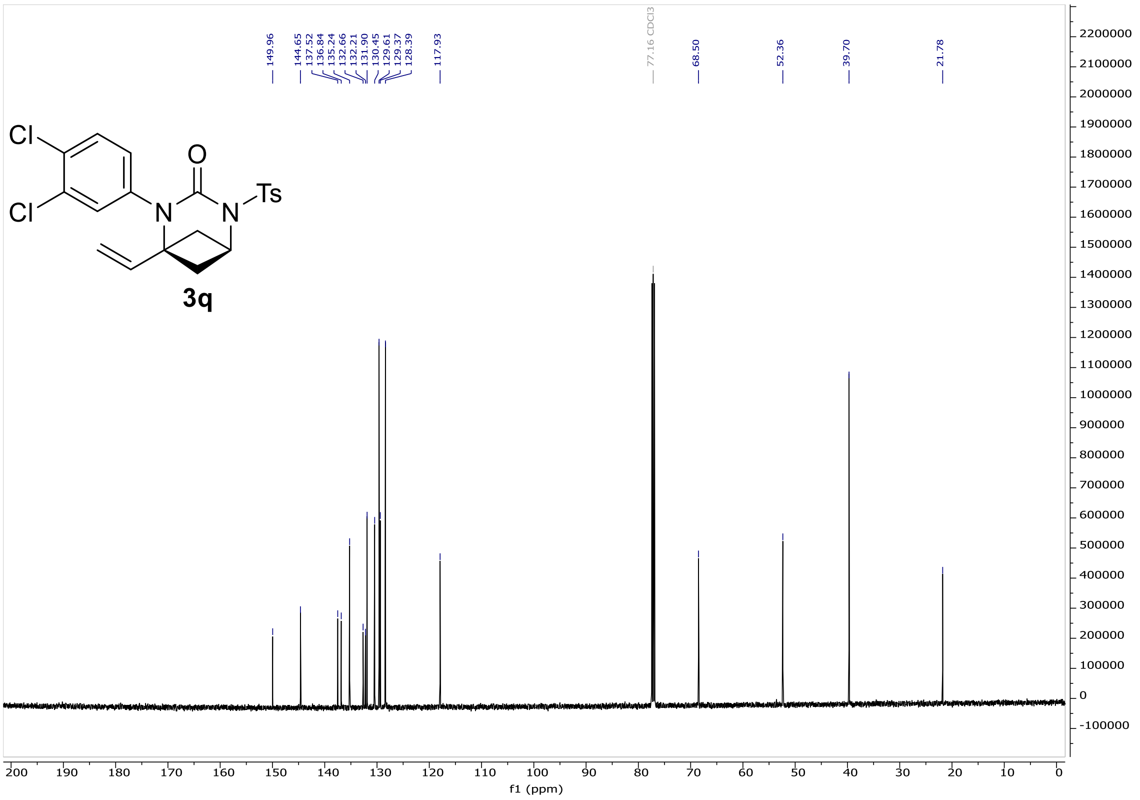

^{1}H NMR (**3r**, $CDCl_3$, 400 MHz)

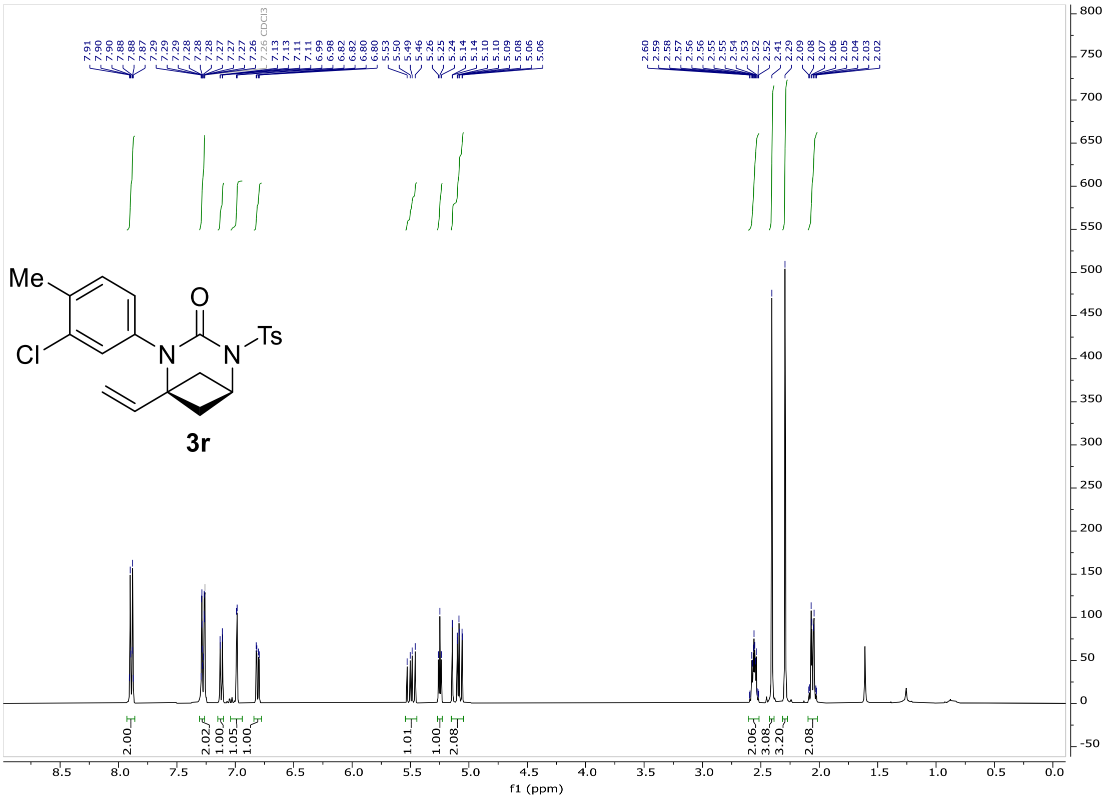

^{13}C NMR (**3r**, $CDCl_3$, 101 MHz)

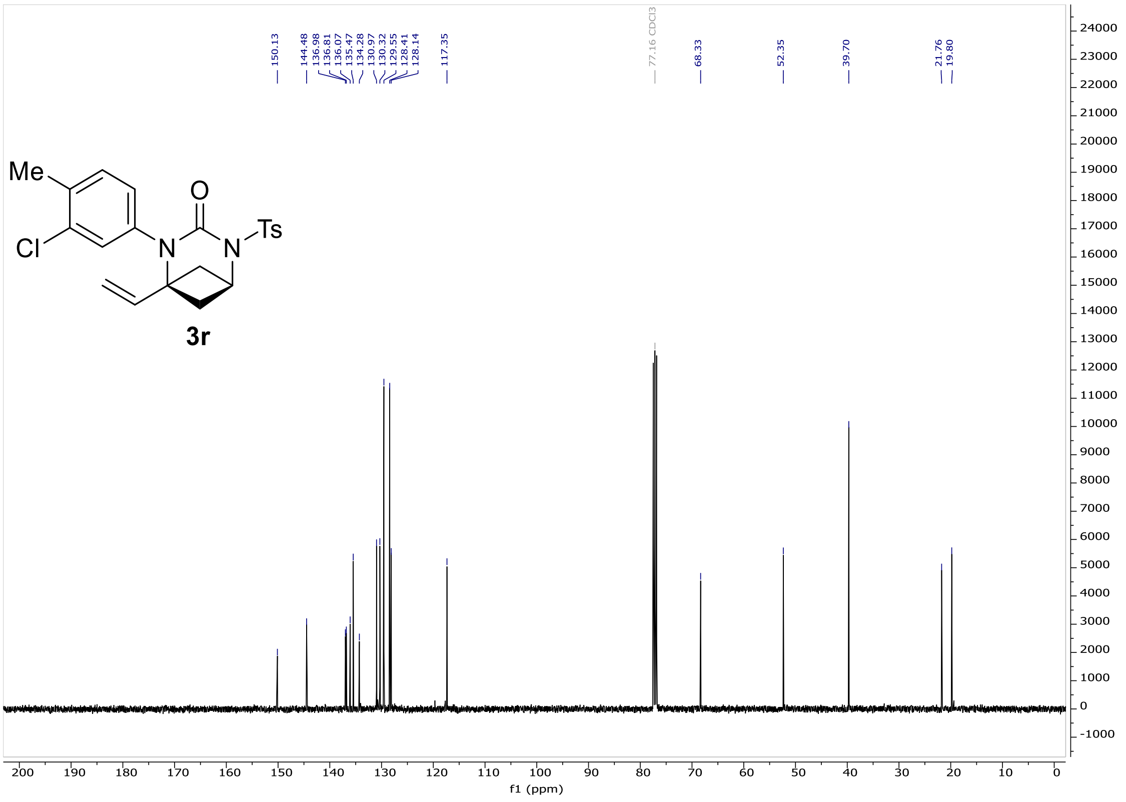

^{1}H NMR (**3s**, $CDCl_3$, 500 MHz)

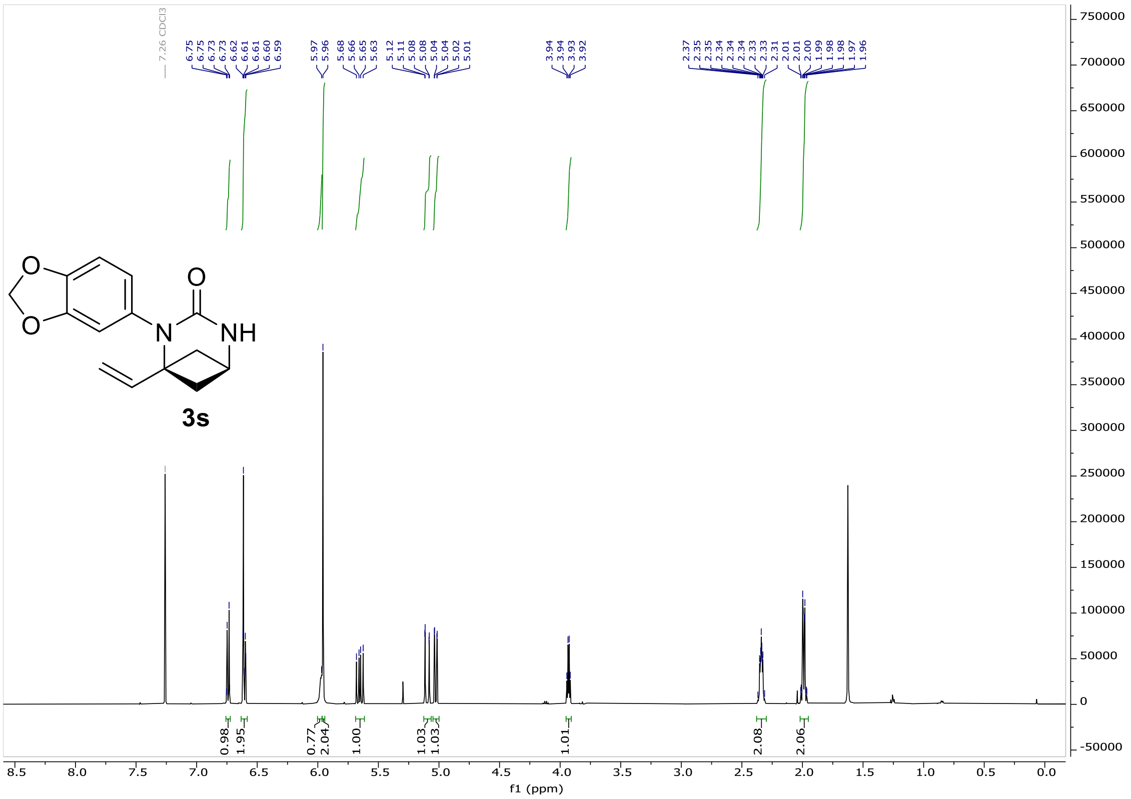

^{13}C NMR (**3s**, $CDCl_3$, 101 MHz)

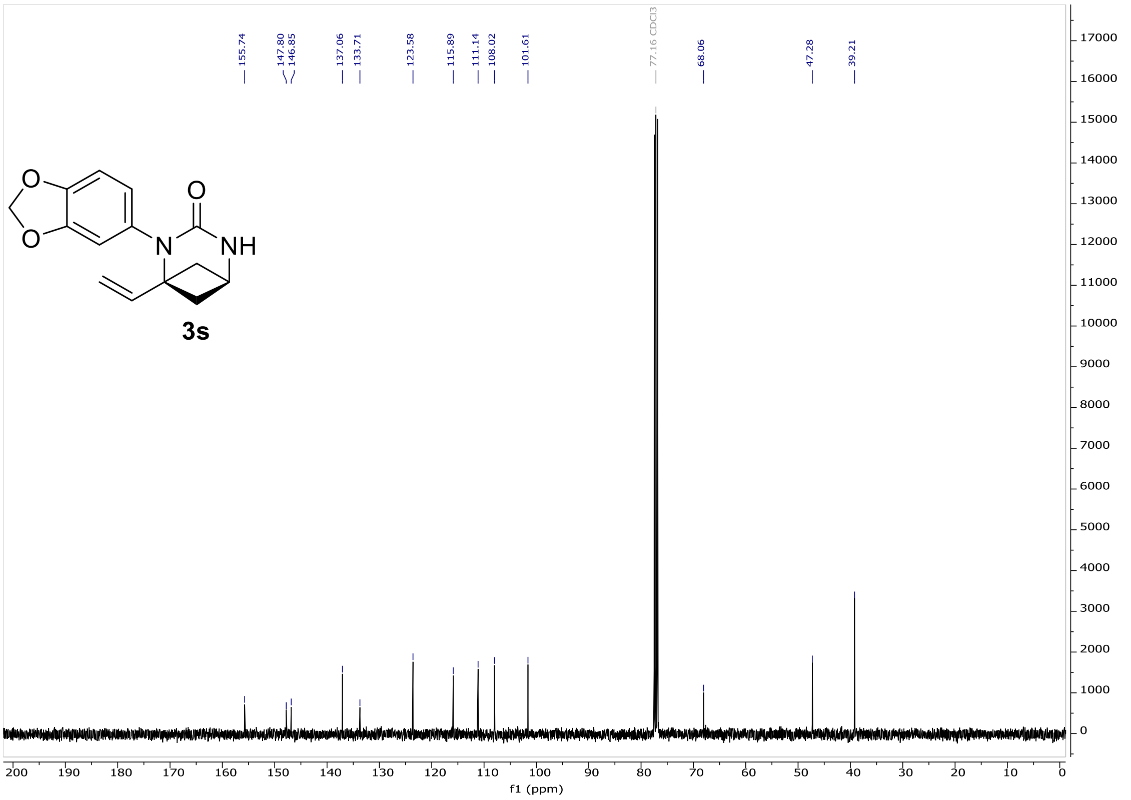

^{1}H NMR (**3t**, $CDCl_3$, 500 MHz)

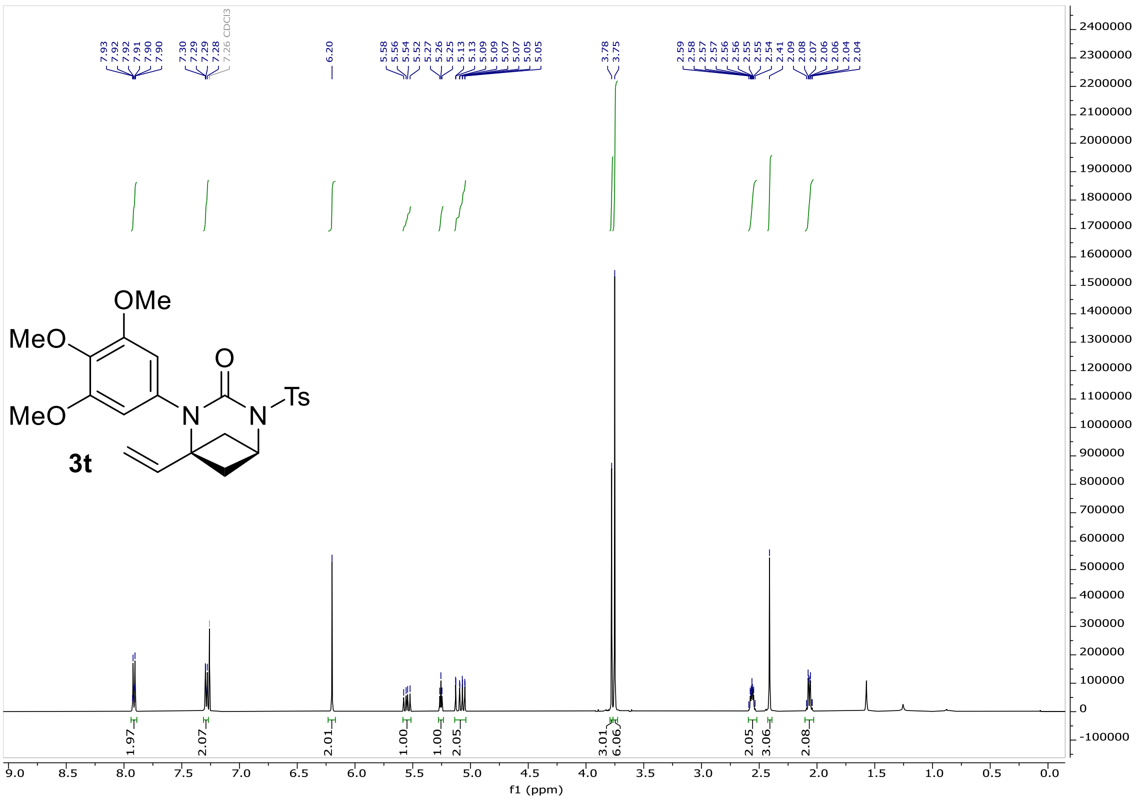

^{13}C NMR (**3t**, $CDCl_3$, 126 MHz)

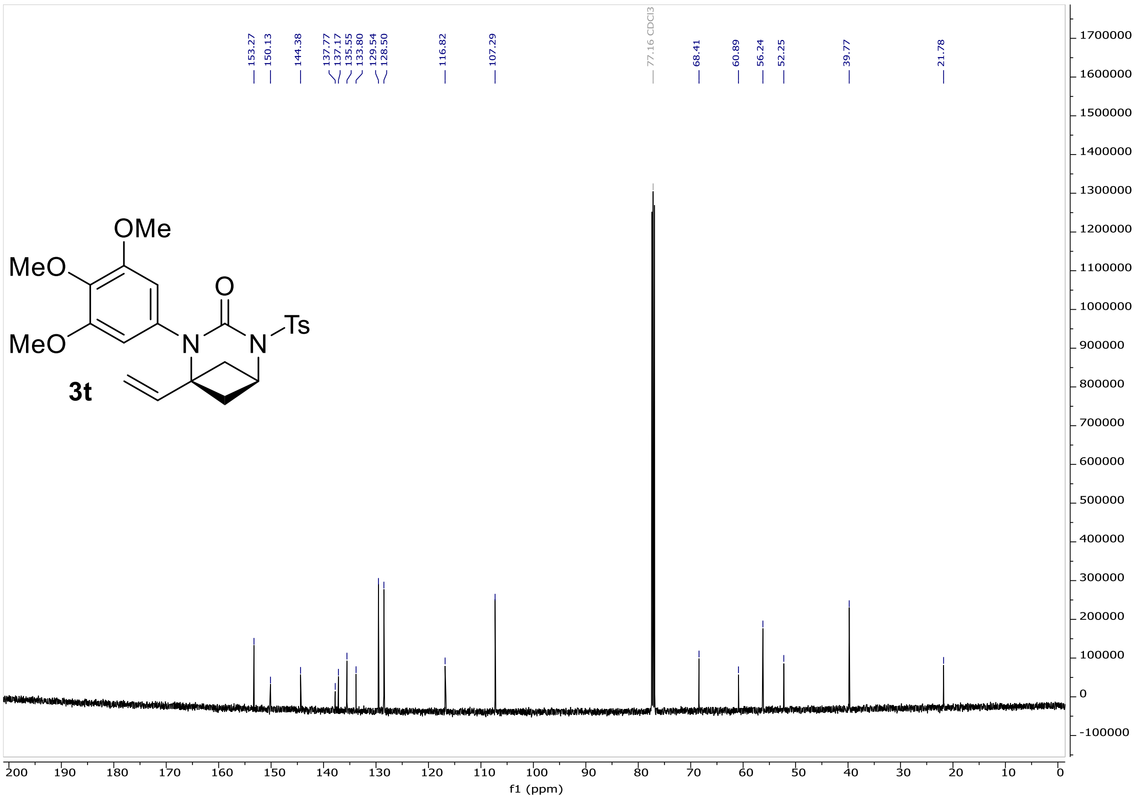

^{1}H NMR (**3u**, $CDCl_3$, 400 MHz)

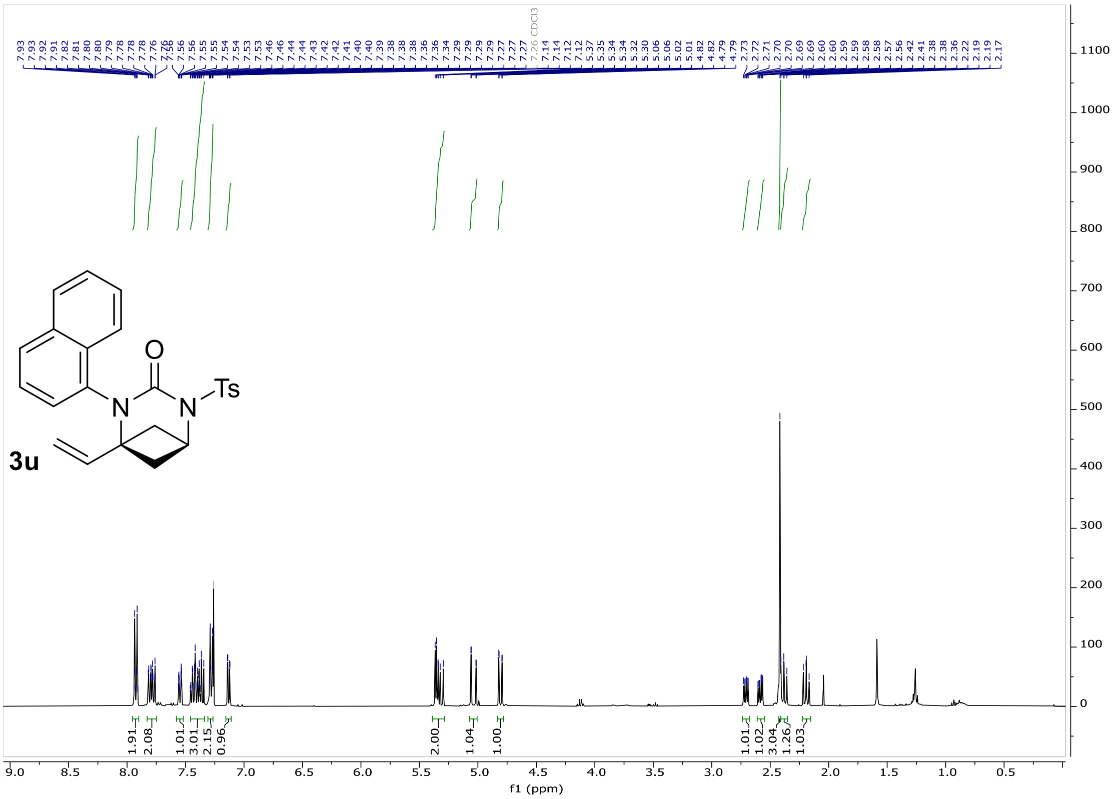

^{13}C NMR (**3u**, $CDCl_3$, 101 MHz)

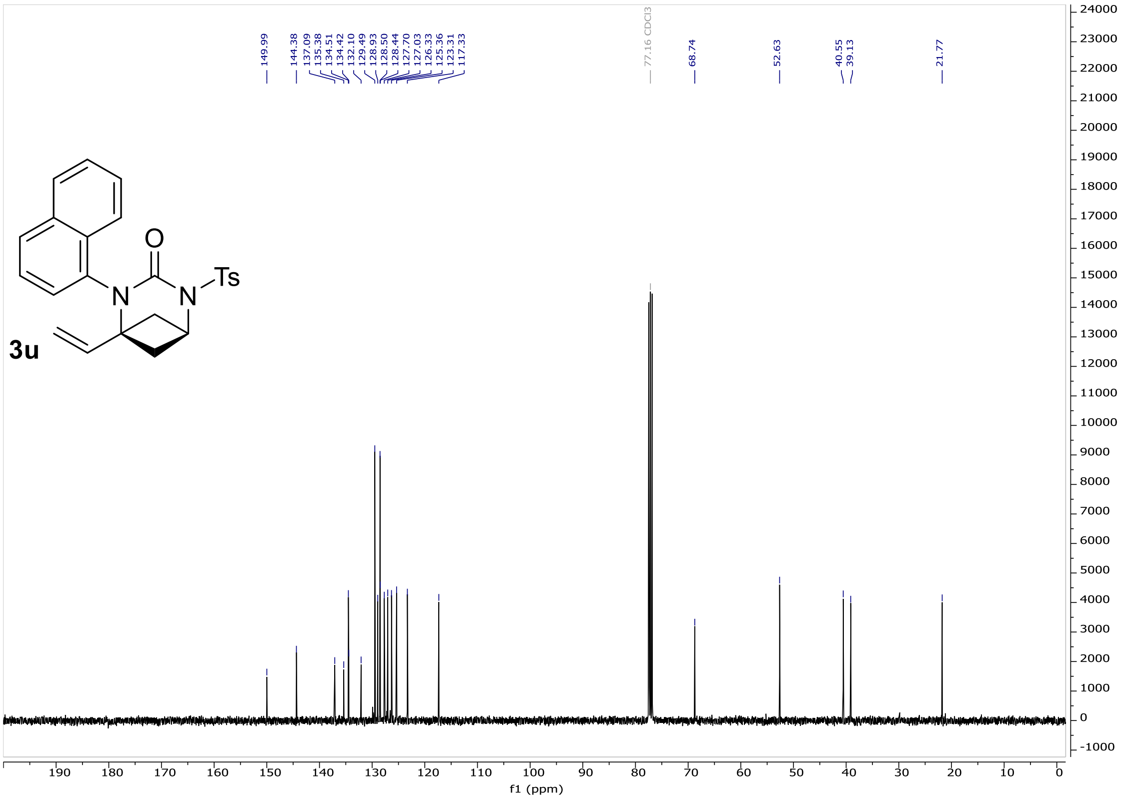

^{1}H NMR (**3v**, $CDCl_3$, 300 MHz)

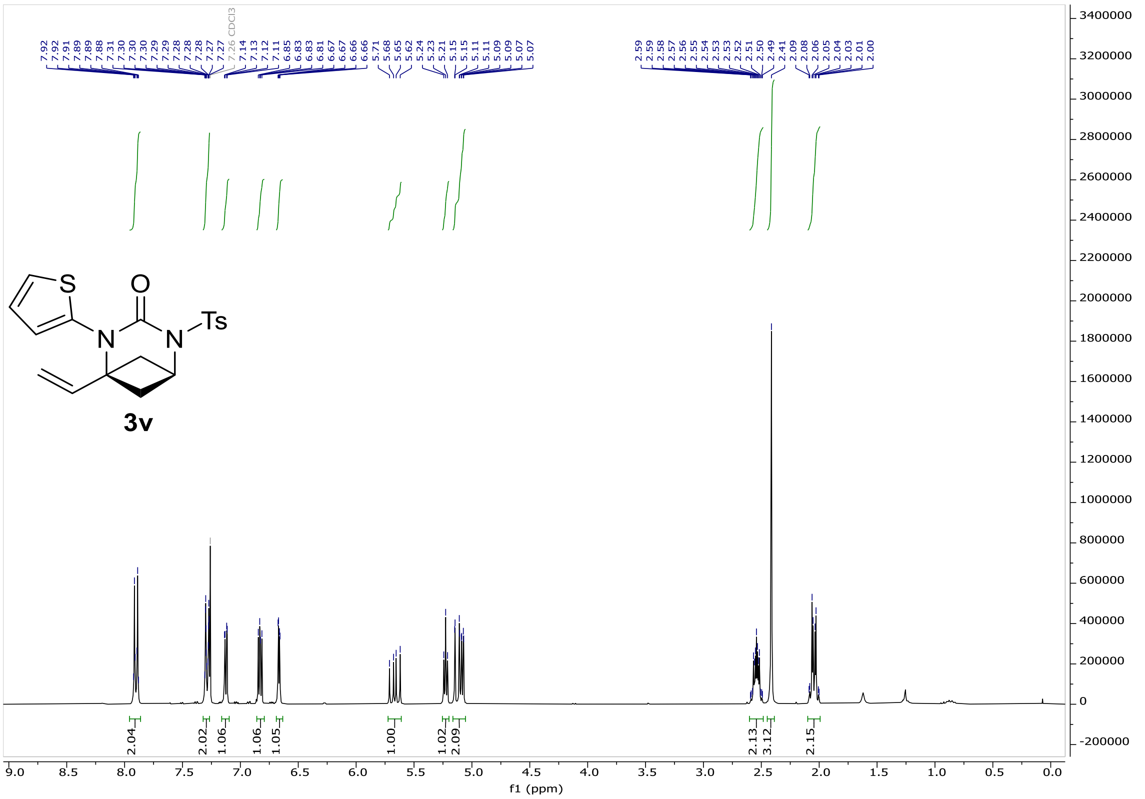

^{13}C NMR (**3v**, $CDCl_3$, 101 MHz)

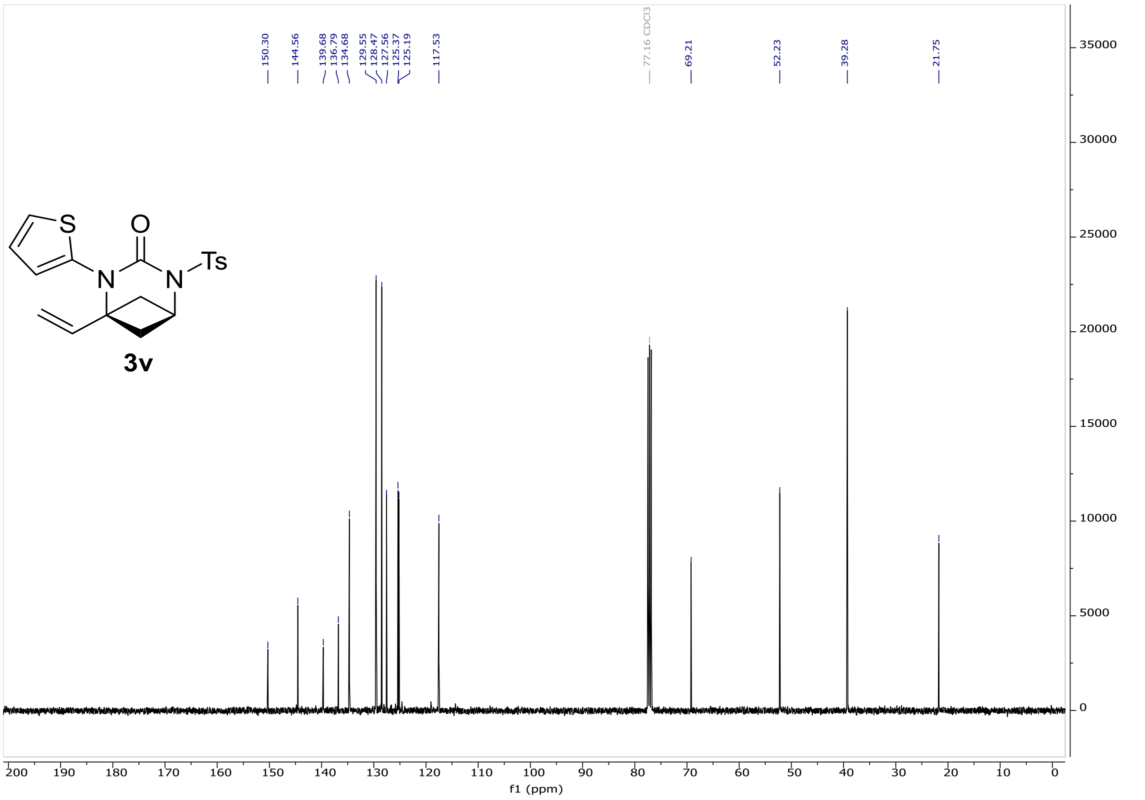

^{1}H NMR (**3w**, $CDCl_3$, 300 MHz)

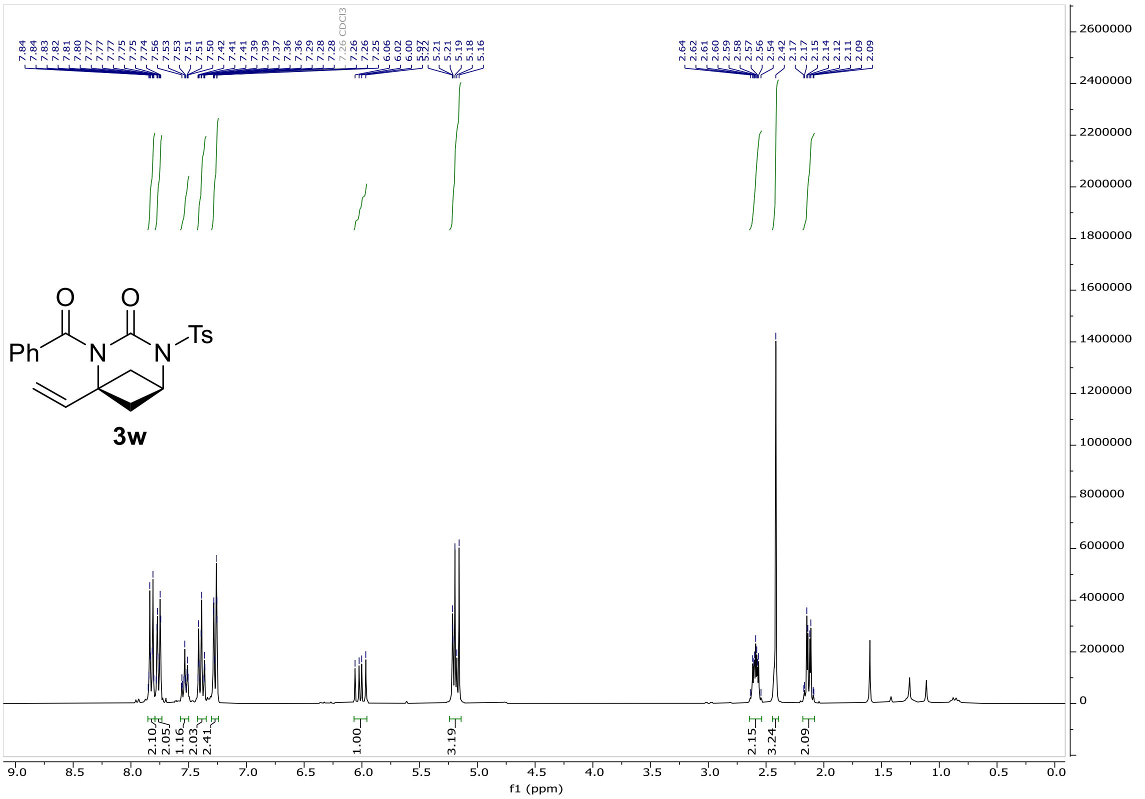

^{13}C NMR (**3w**, $CDCl_3$, 101 MHz)

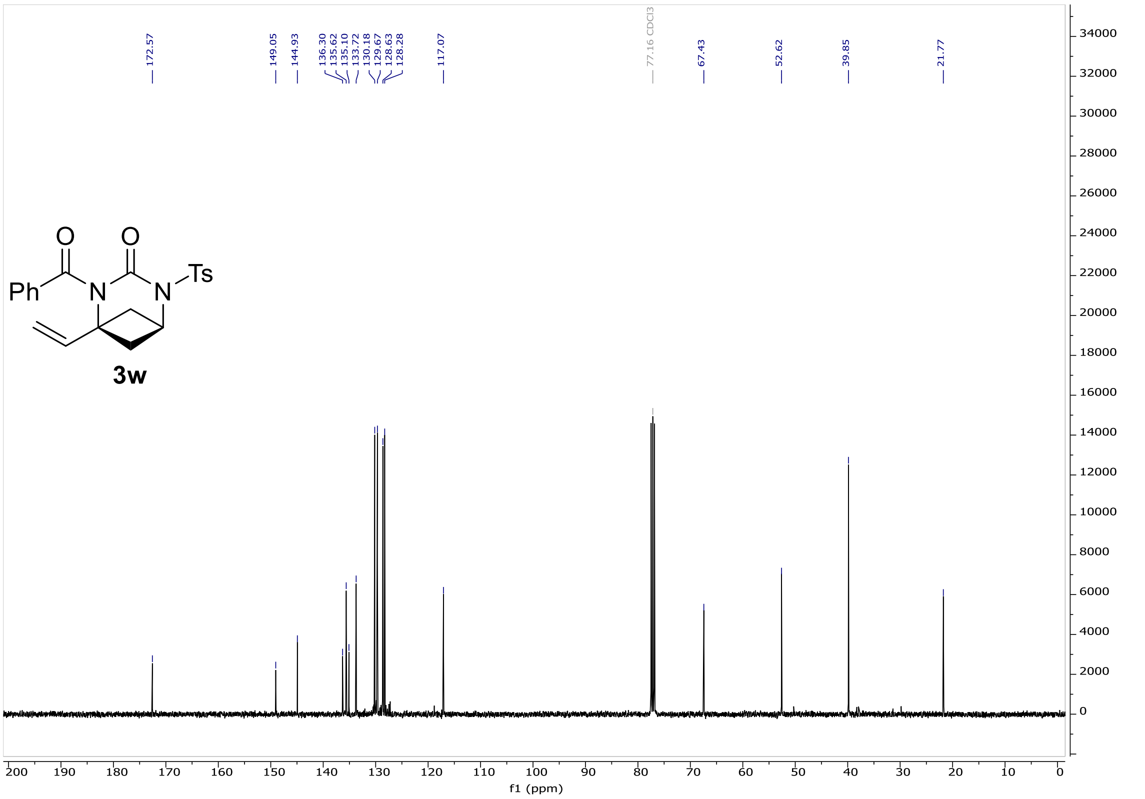

^{1}H NMR (**5a**, $CDCl_3$, 400 MHz)

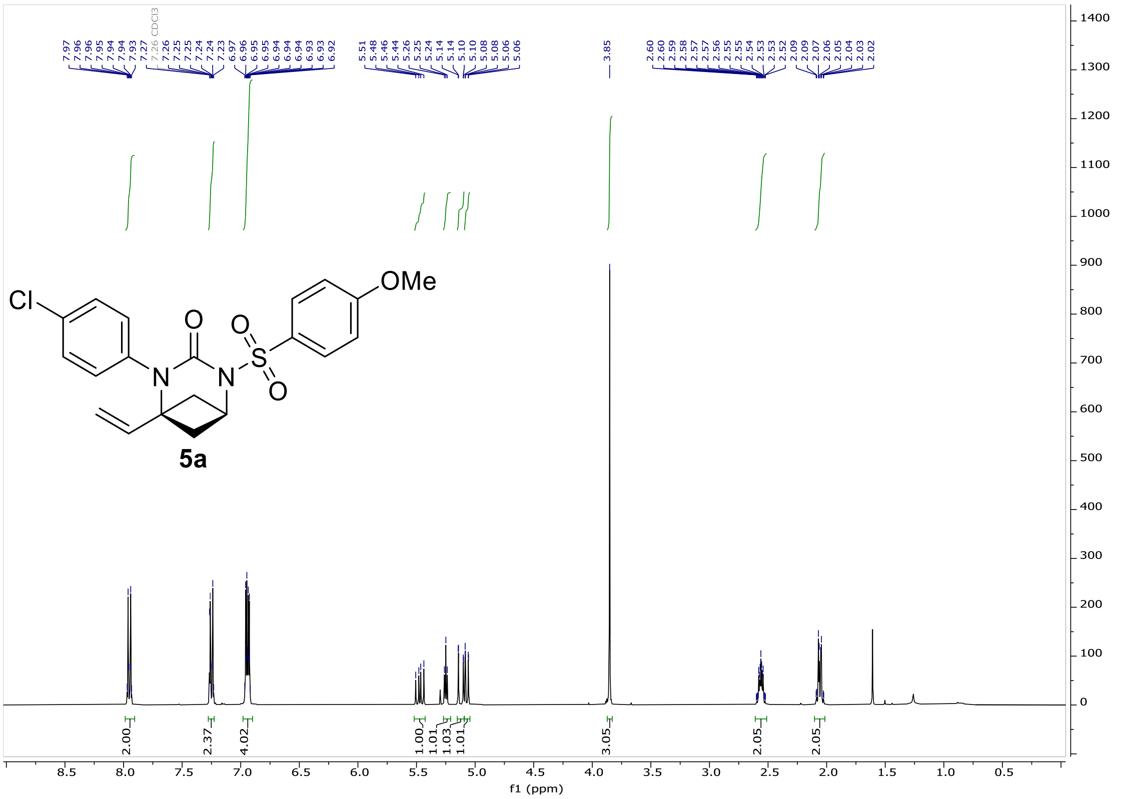

^{13}C NMR (**5a**, $CDCl_3$, 101 MHz)

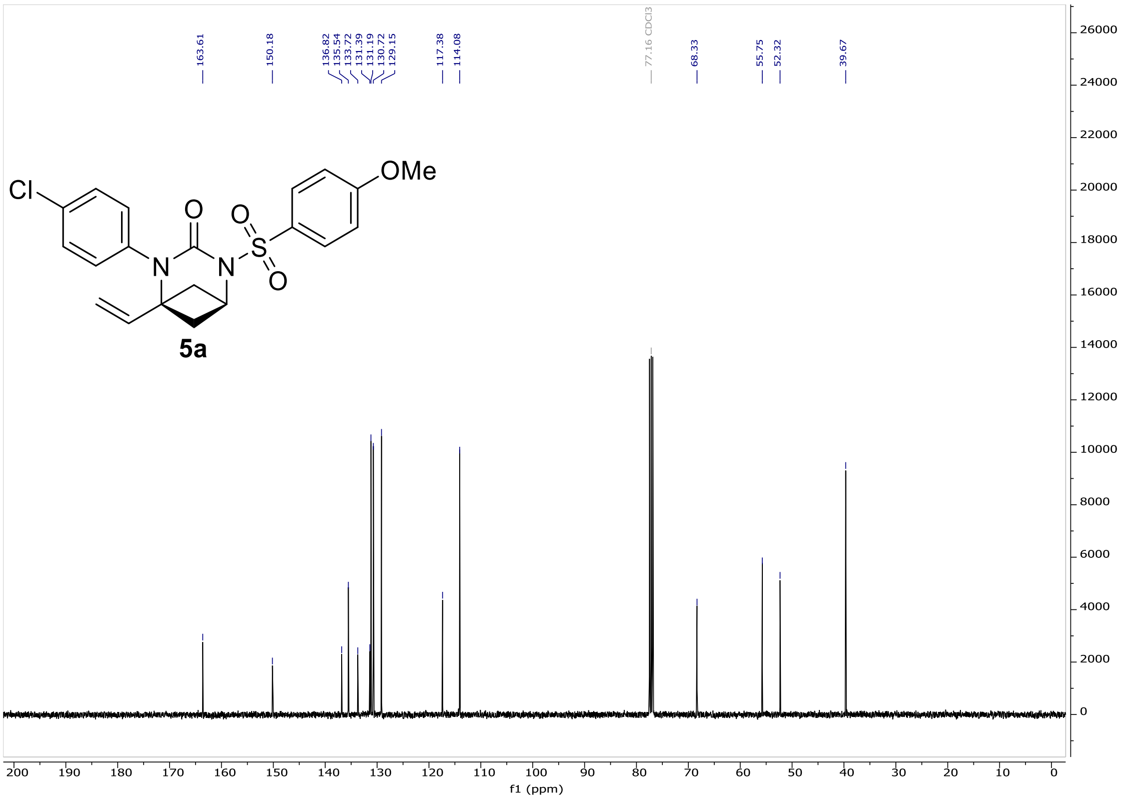

^{1}H NMR (**5b**, $CDCl_3$, 400 MHz)

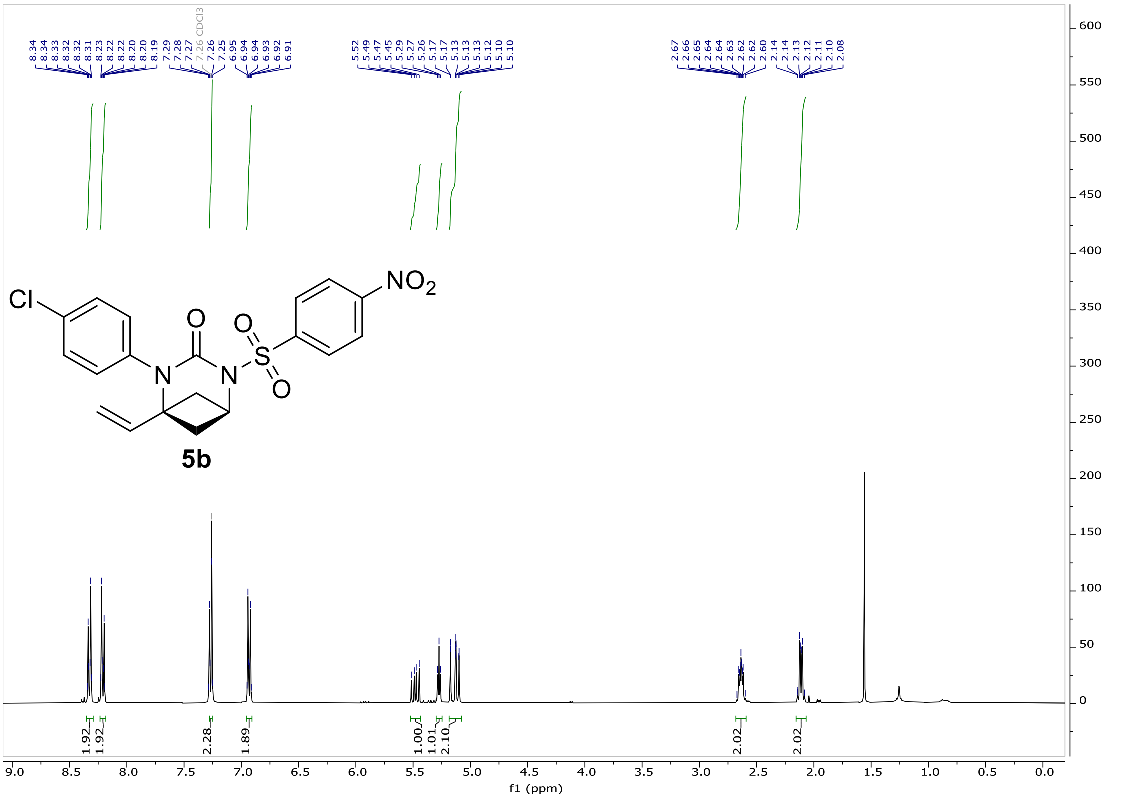

^{13}C NMR (**5b**, $CDCl_3$, 101 MHz)

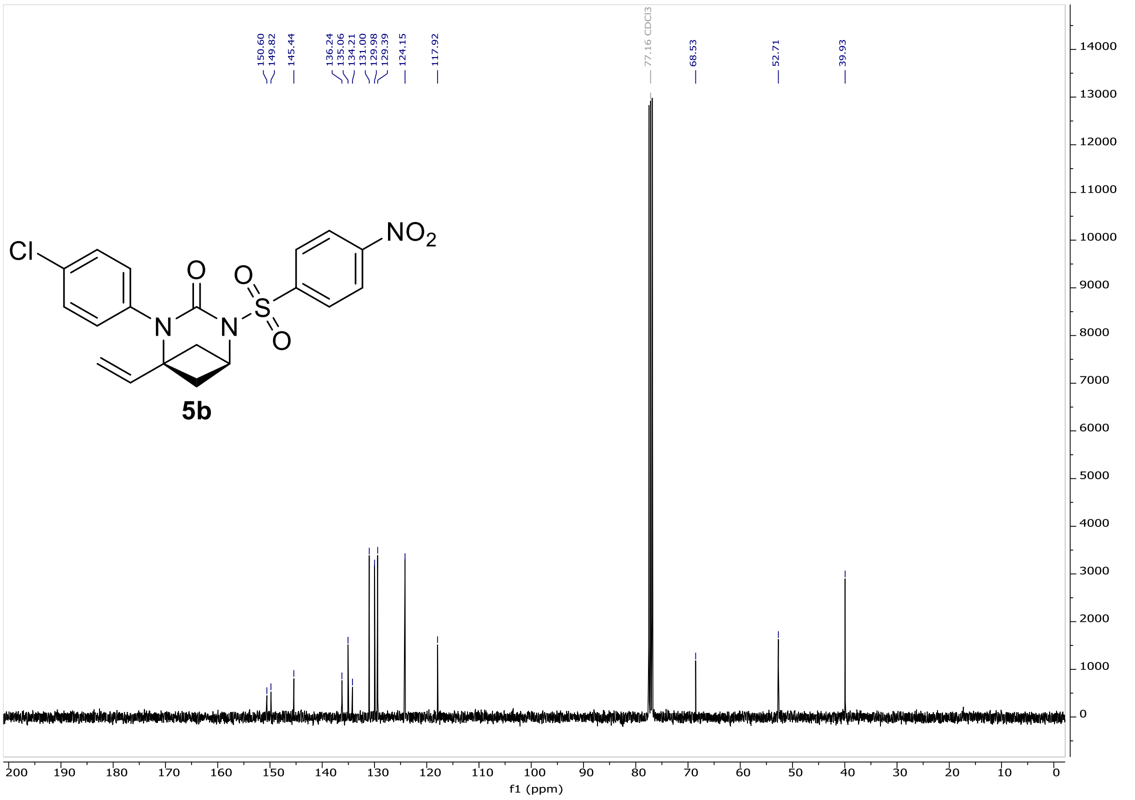

^{1}H NMR (**5c**, $CDCl_3$, 500 MHz)

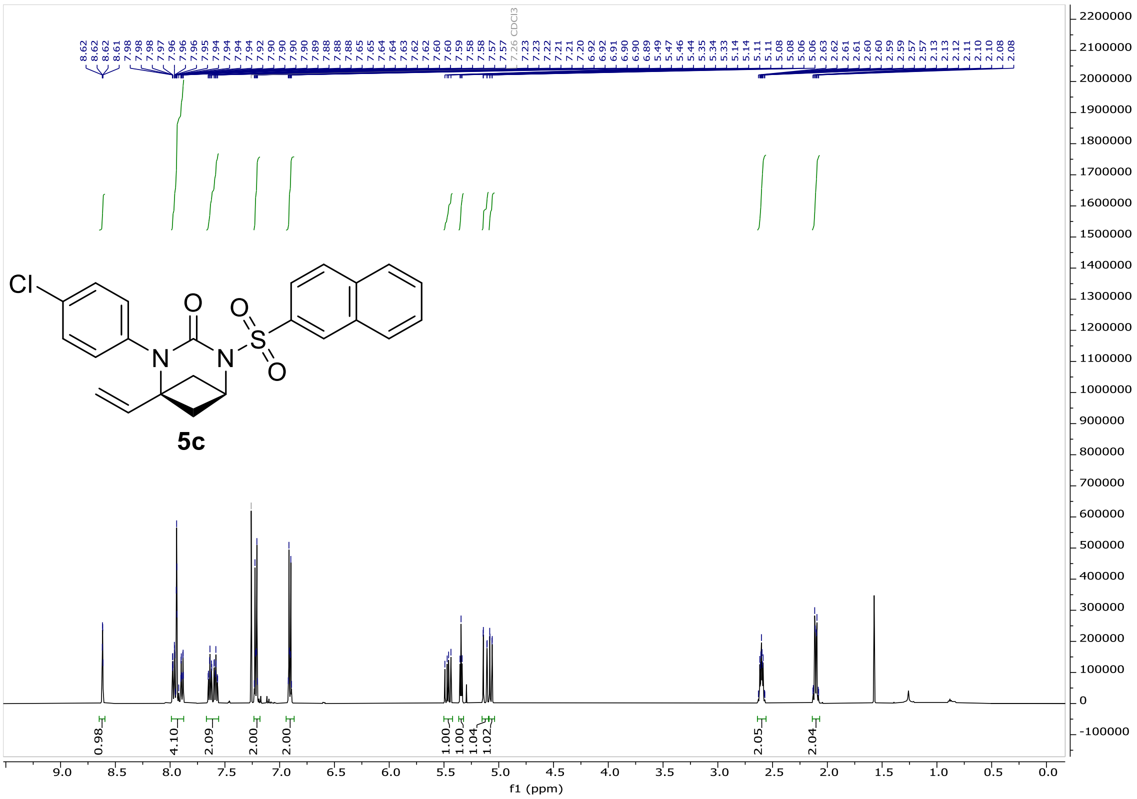

^{13}C NMR (**5c**, $CDCl_3$, 101 MHz)

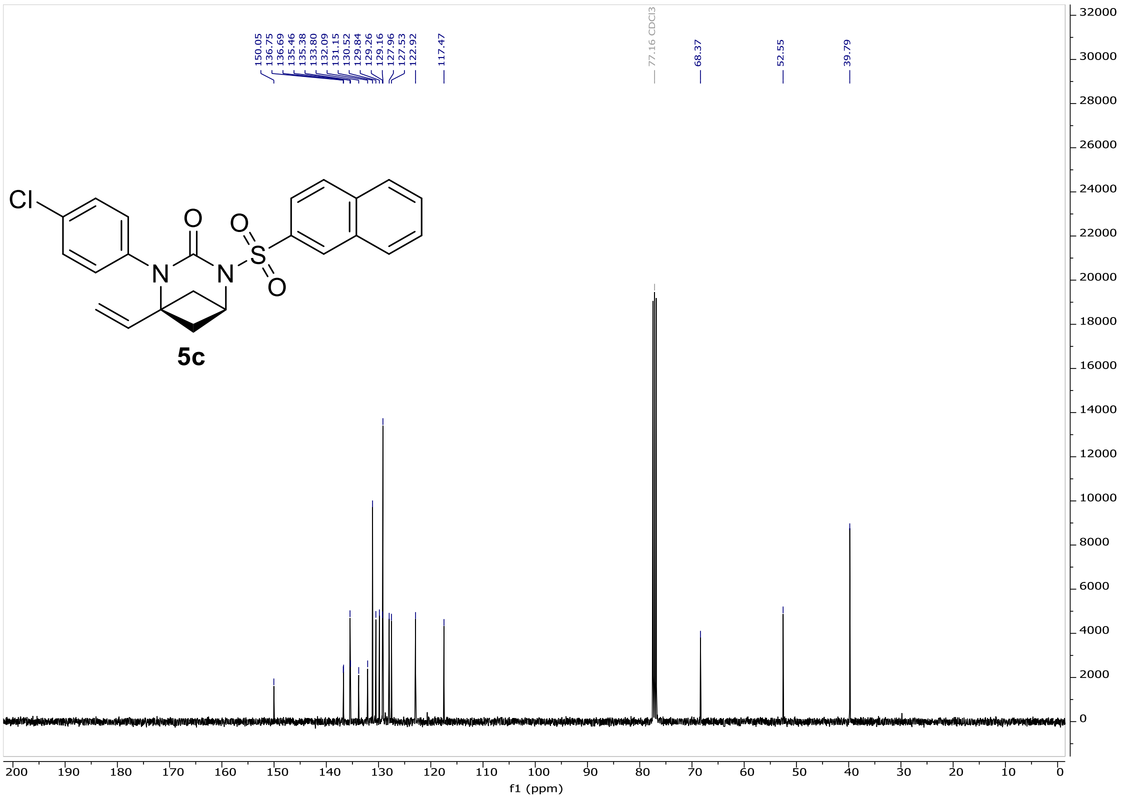

^{1}H NMR (**5d**, $CDCl_3$, 500 MHz)

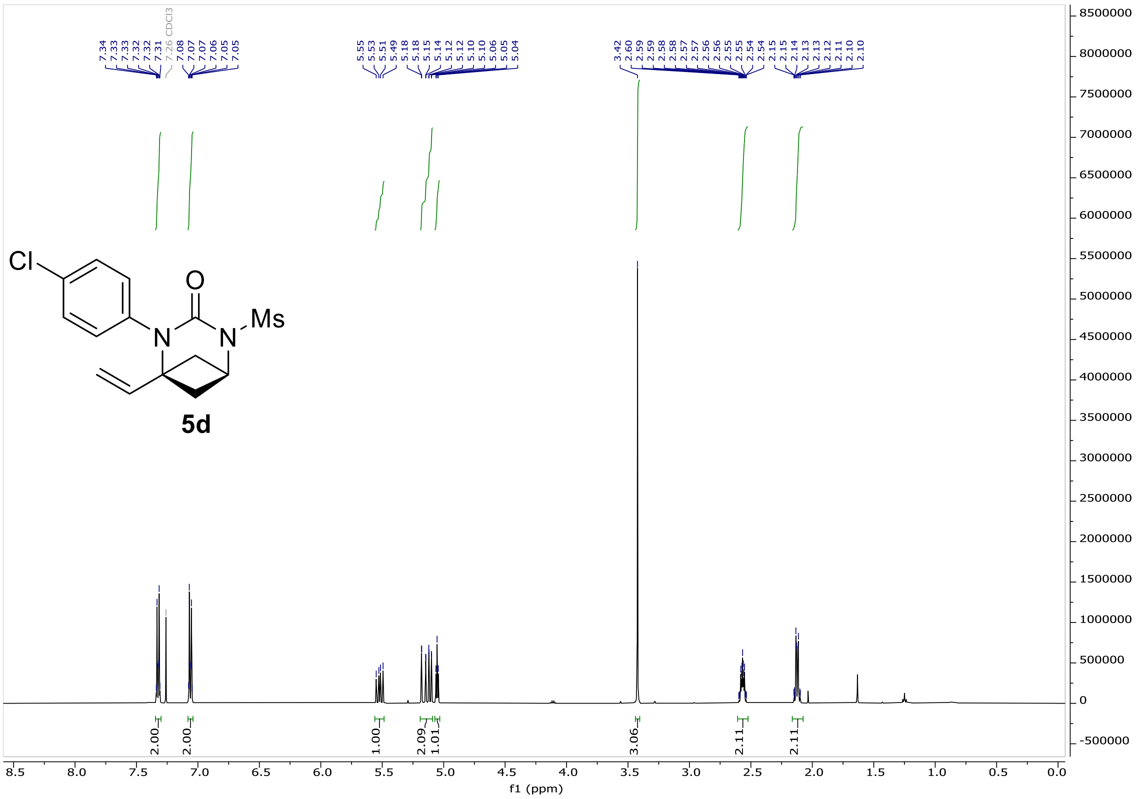

^{13}C NMR (**5d**, $CDCl_3$, 126 MHz)

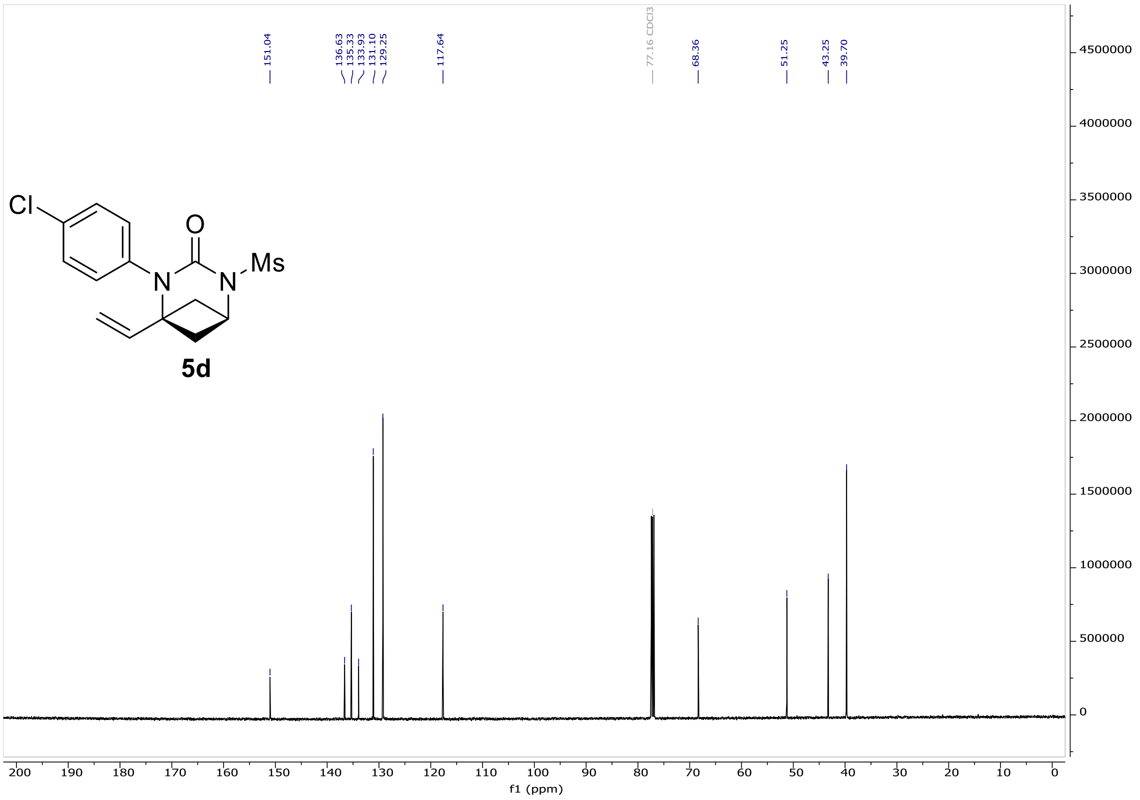

^{1}H NMR (**5e**, $CDCl_3$, 400 MHz)

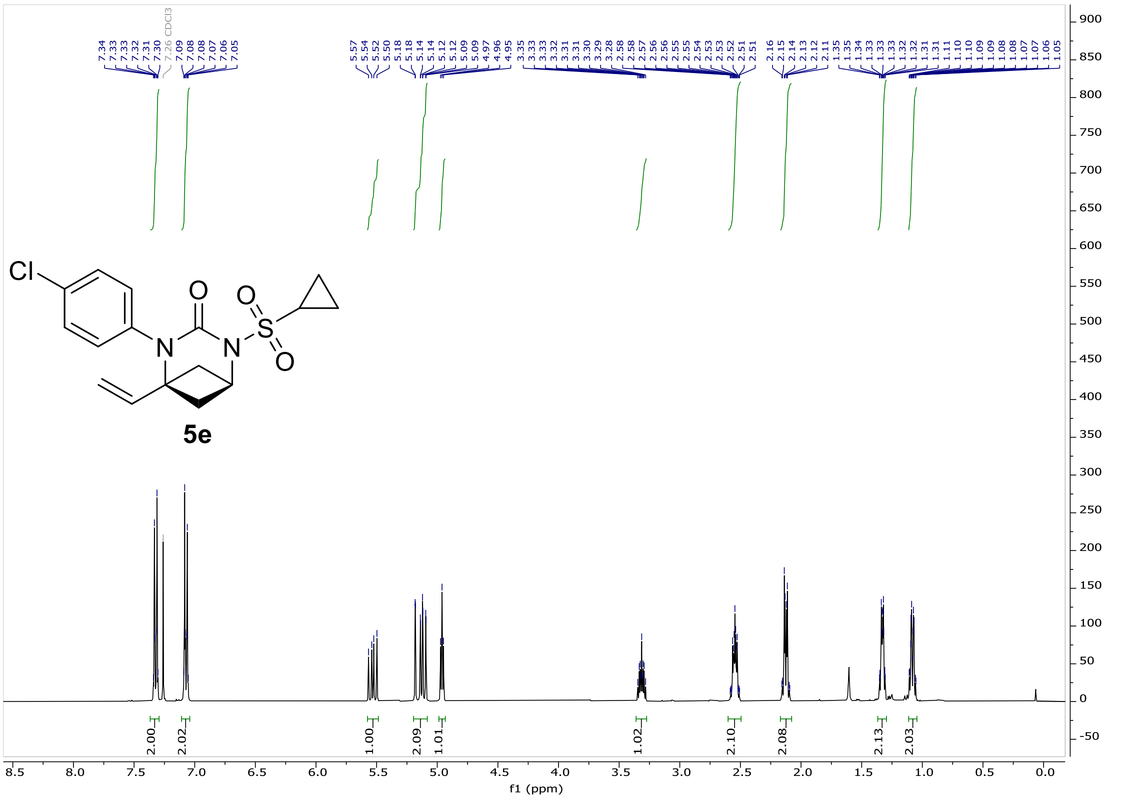

^{13}C NMR (**5e**, $CDCl_3$, 101 MHz)

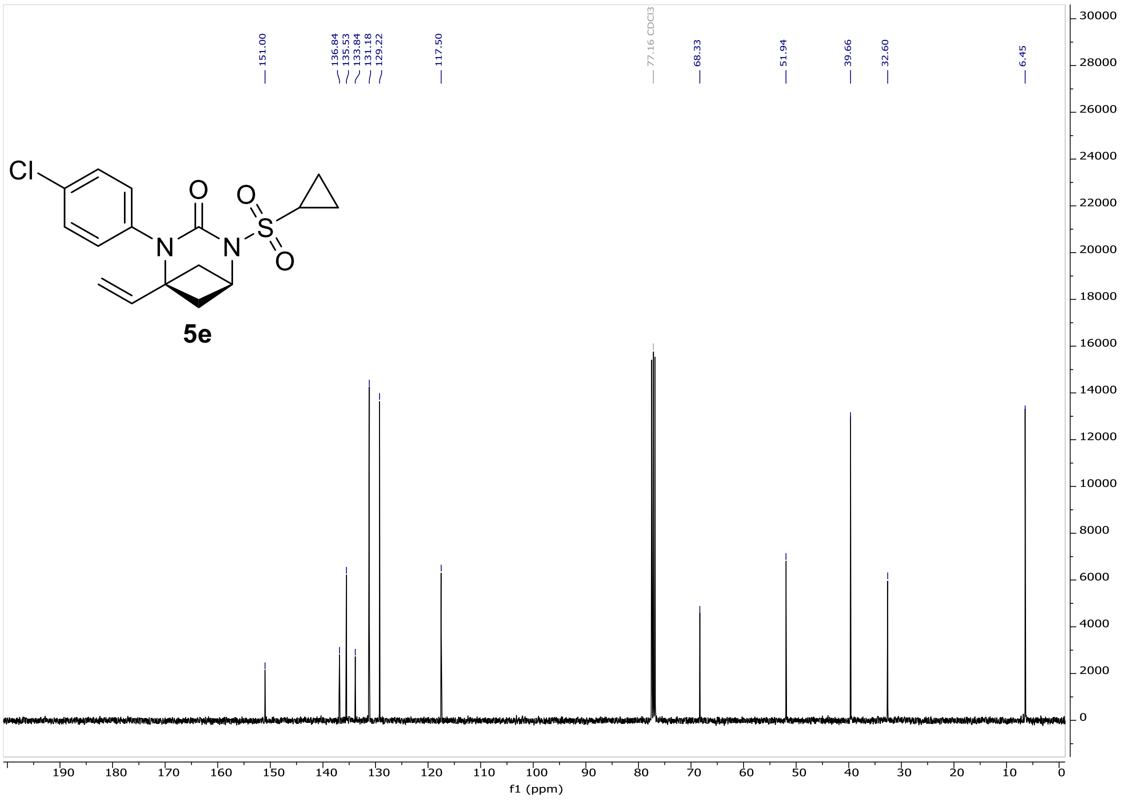

^{1}H NMR (**5f**, $CDCl_3$, 300 MHz)

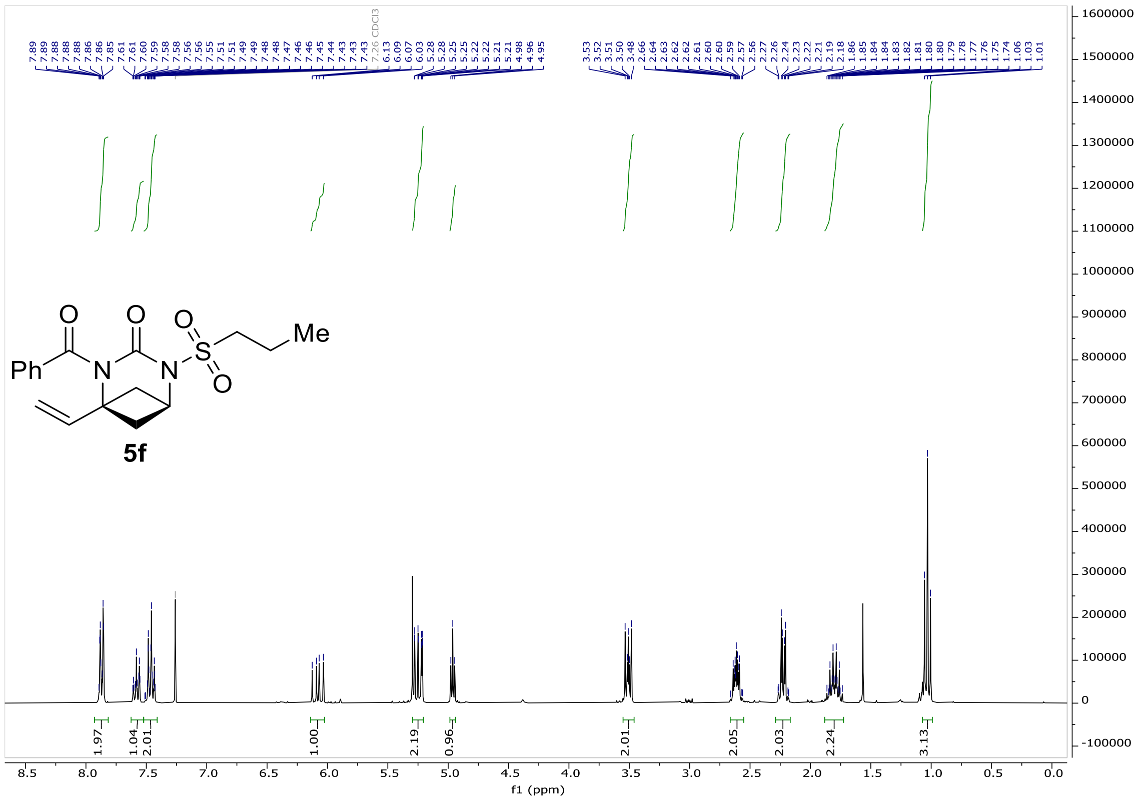

^{13}C NMR (**5f**, $CDCl_3$, 101 MHz)

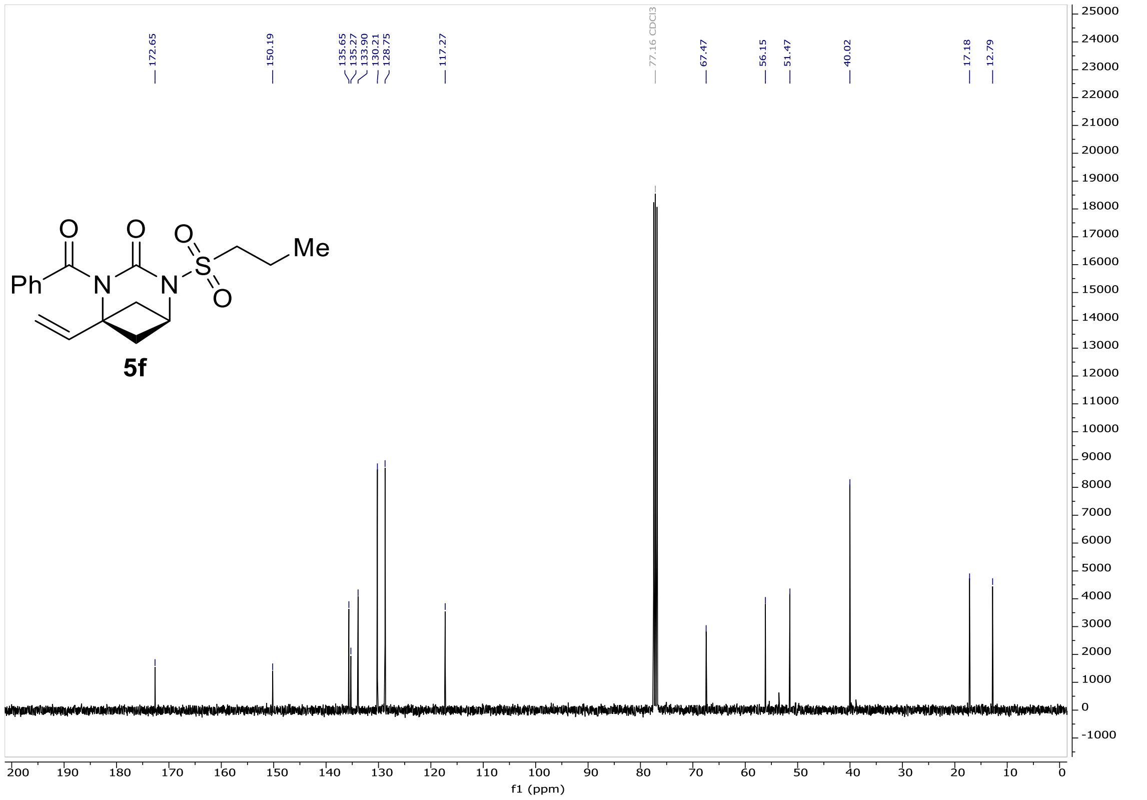

^{1}H NMR (**5g**, $CDCl_3$, 400 MHz)

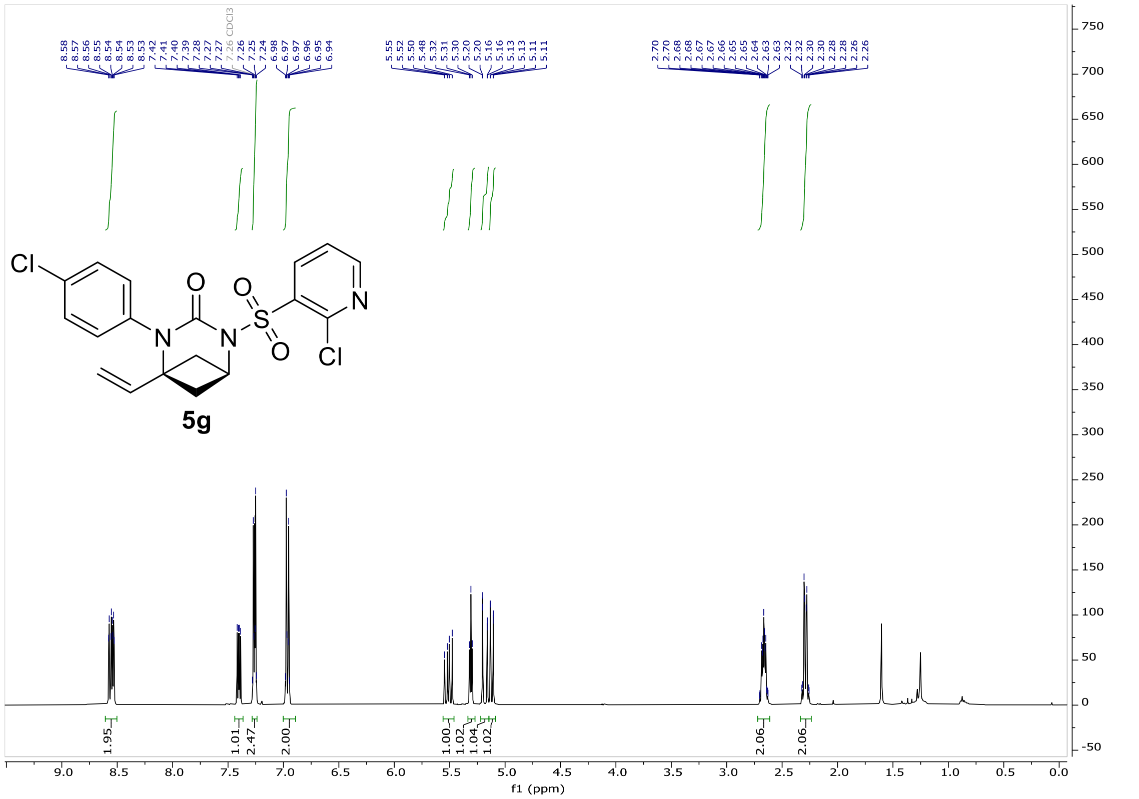

^{13}C NMR (**5g**, $CDCl_3$, 101 MHz)

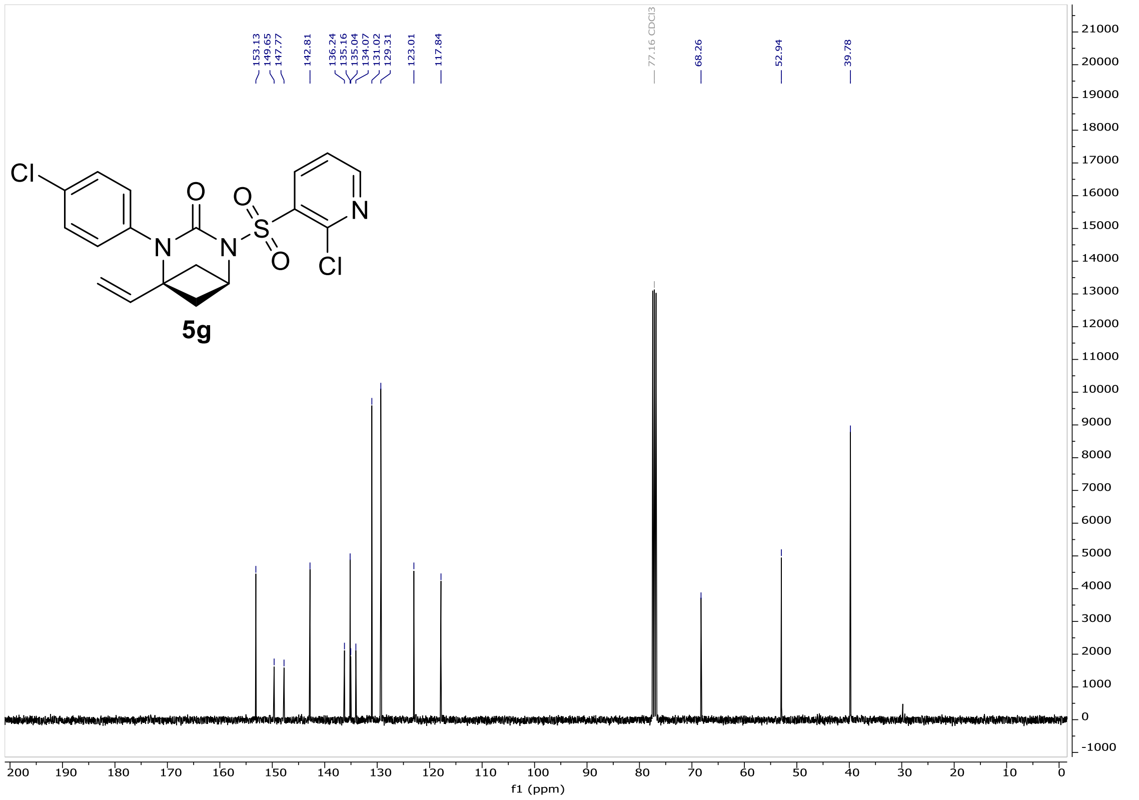

^{1}H NMR (**5h**, $CDCl_3$, 400 MHz)

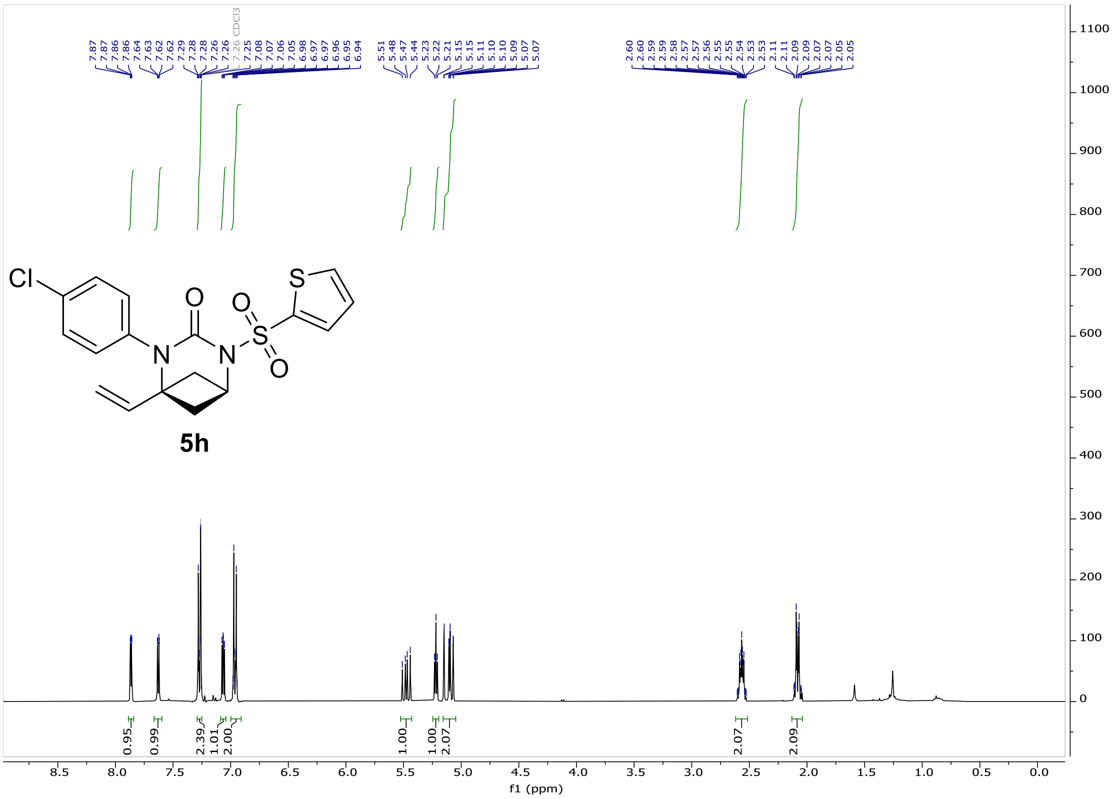

^{13}C NMR (**5h**, $CDCl_3$, 101 MHz)

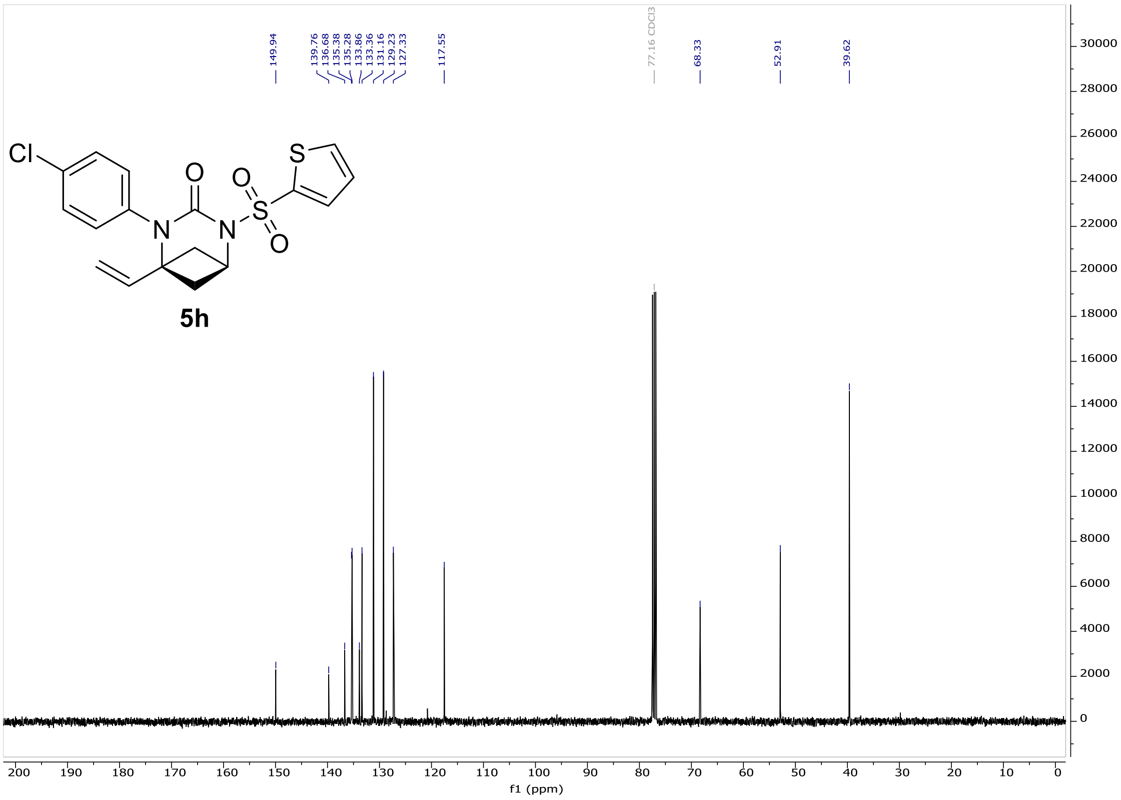

^{1}H NMR (**5i**, $CDCl_3$, 500 MHz)

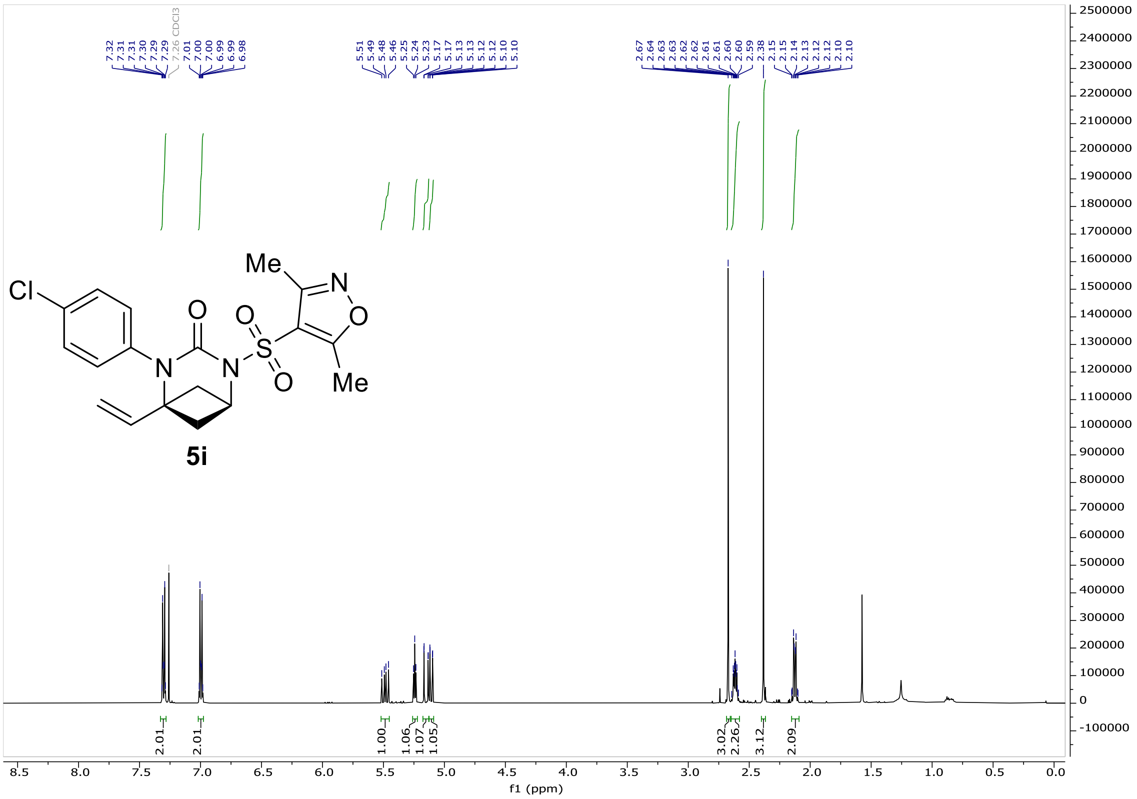

^{13}C NMR (**5i**, $CDCl_3$, 126 MHz)

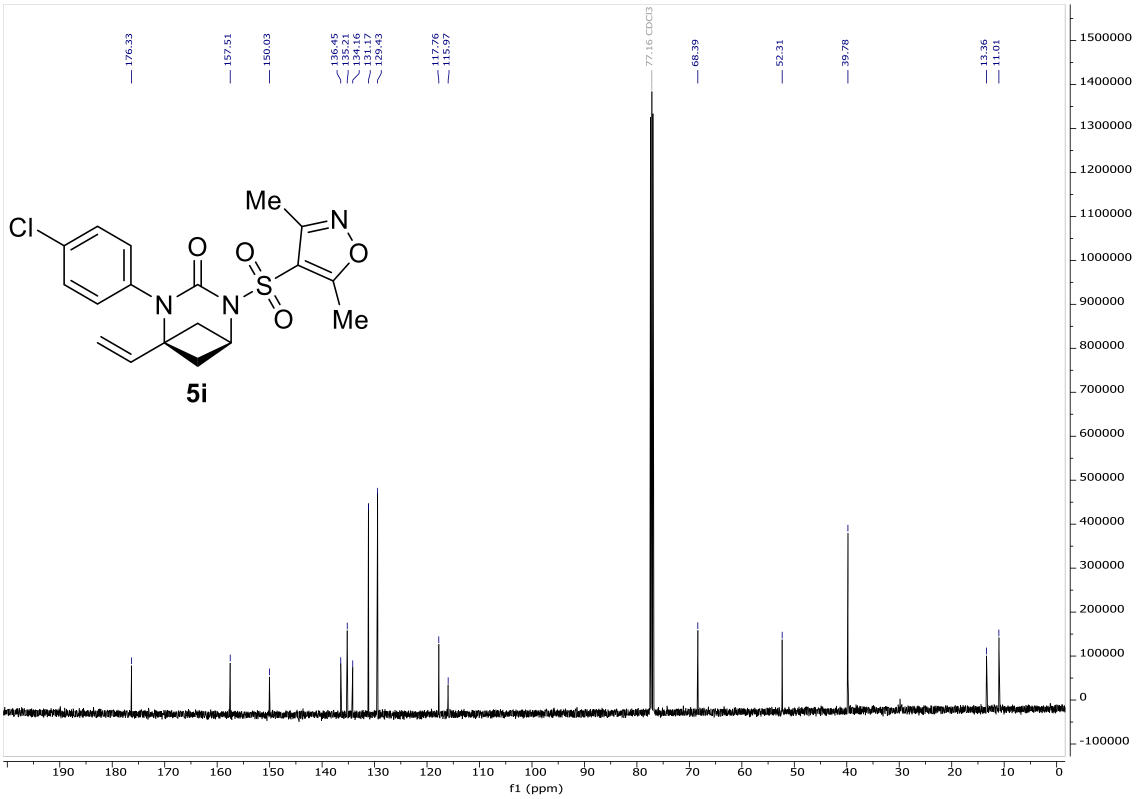

^{1}H NMR (**6**, $CDCl_3$, 400 MHz)

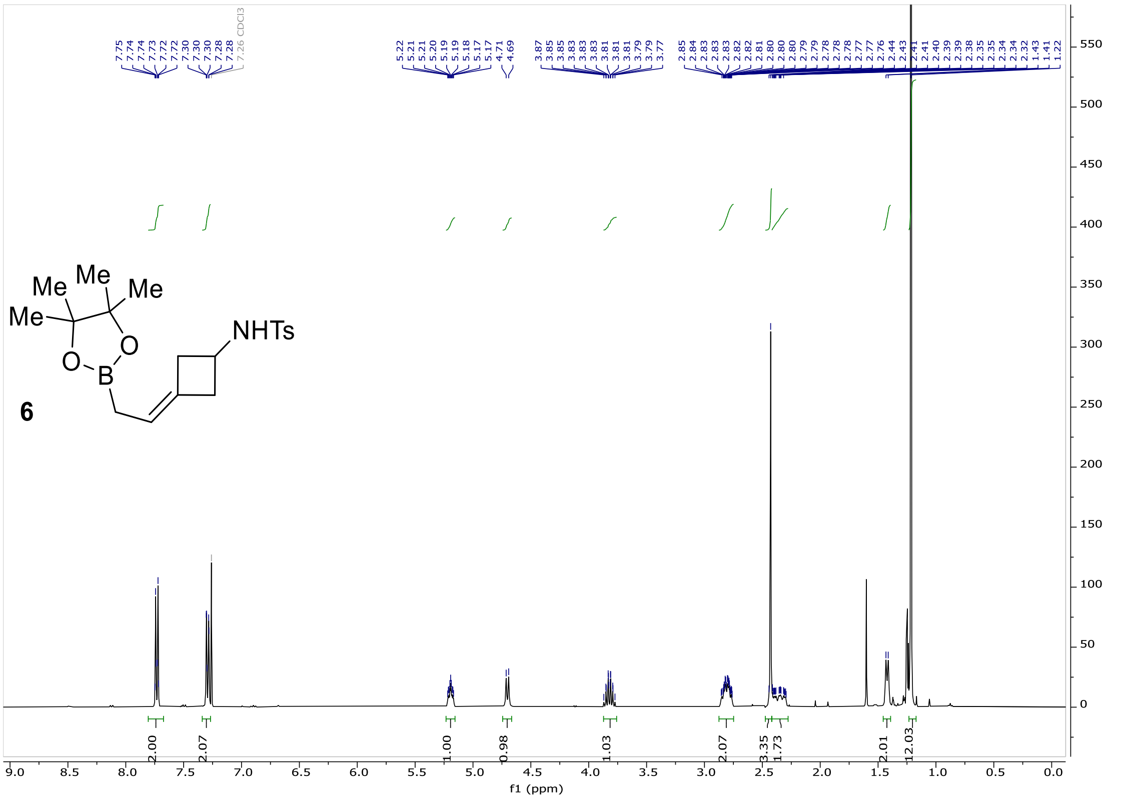

^{13}C NMR (**6**, $CDCl_3$, 101 MHz)

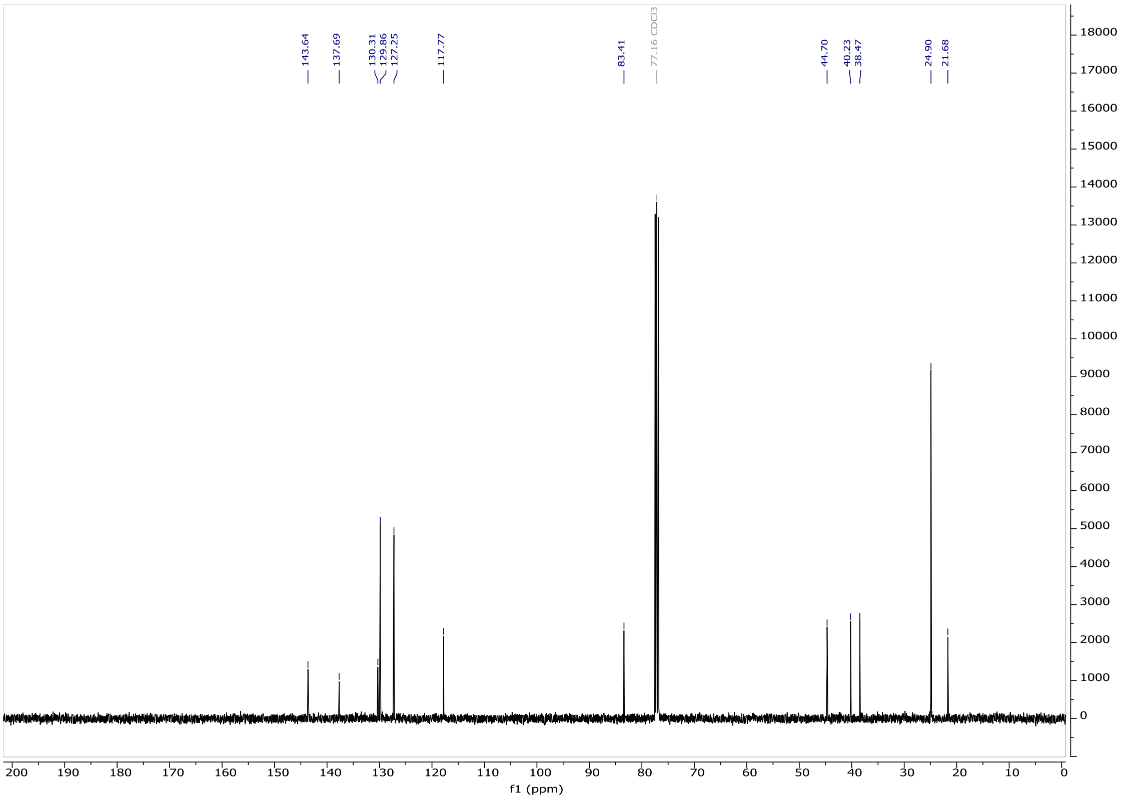

^{1}H NMR (**7**, $CDCl_3$, 400 MHz)

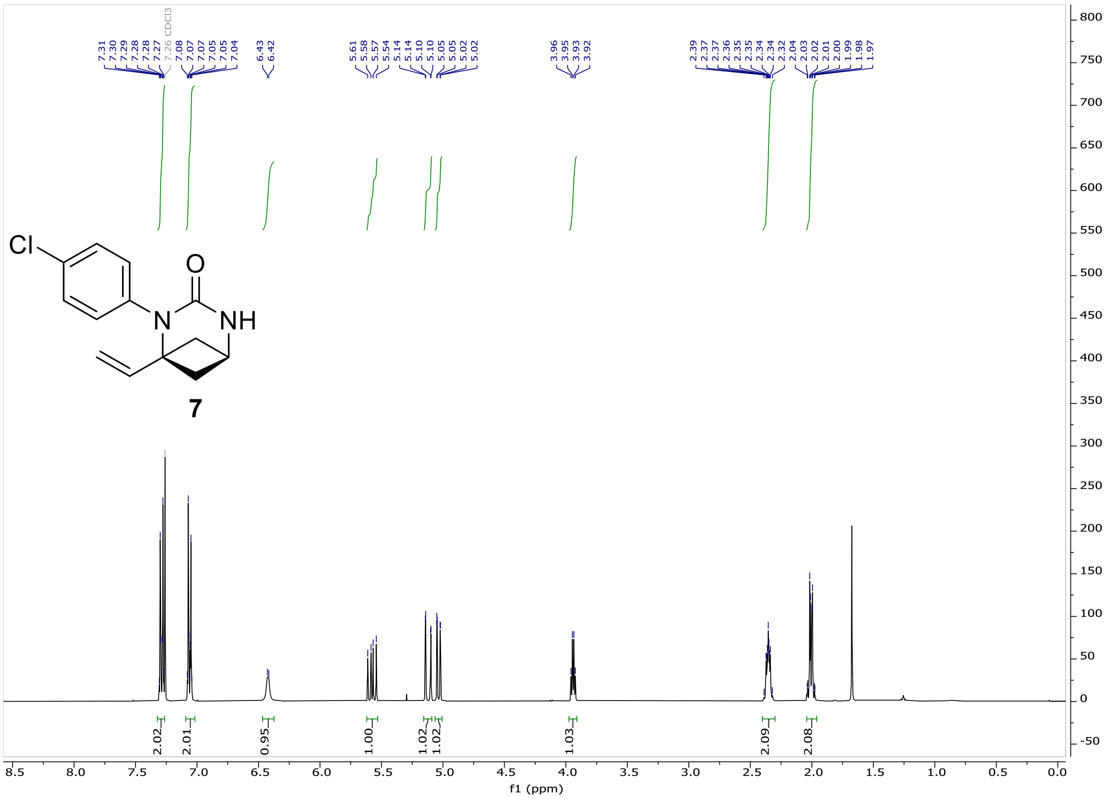

^{13}C NMR (**7**, $CDCl_3$, 101 MHz)

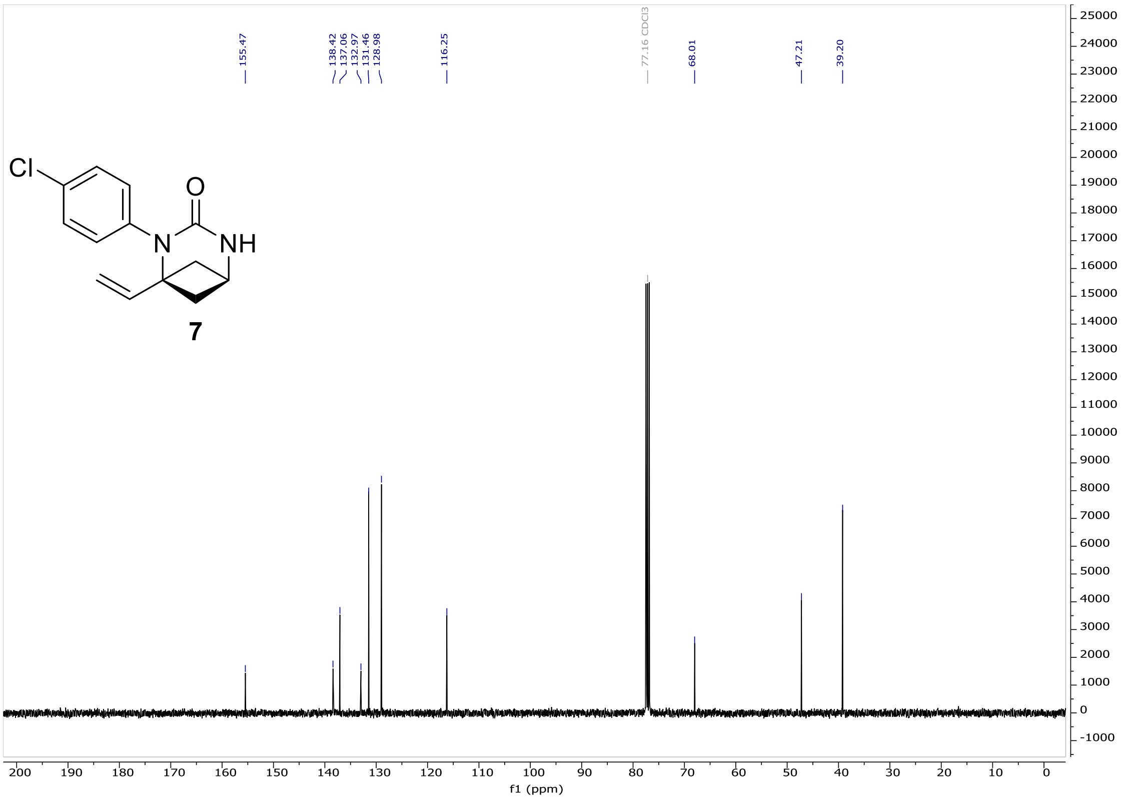

^{1}H NMR (**8**, $CDCl_3$, 500 MHz)

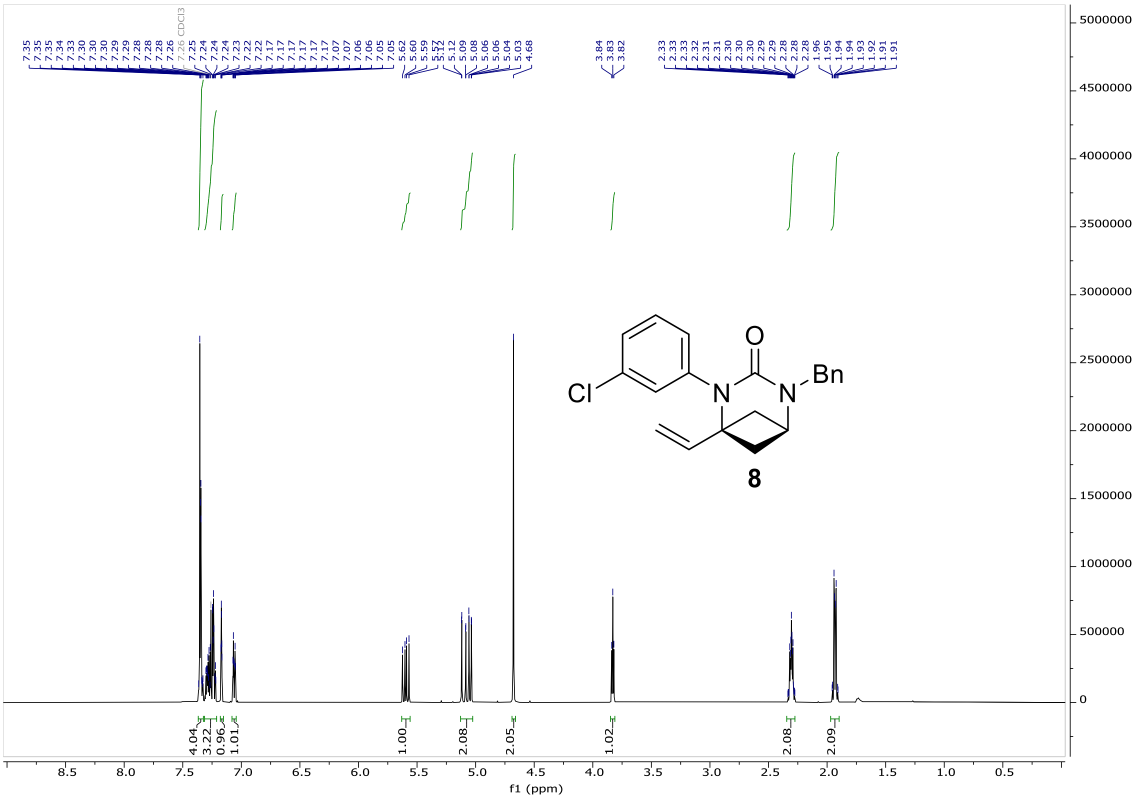

^{13}C NMR (**8**, $CDCl_3$, 101 MHz)

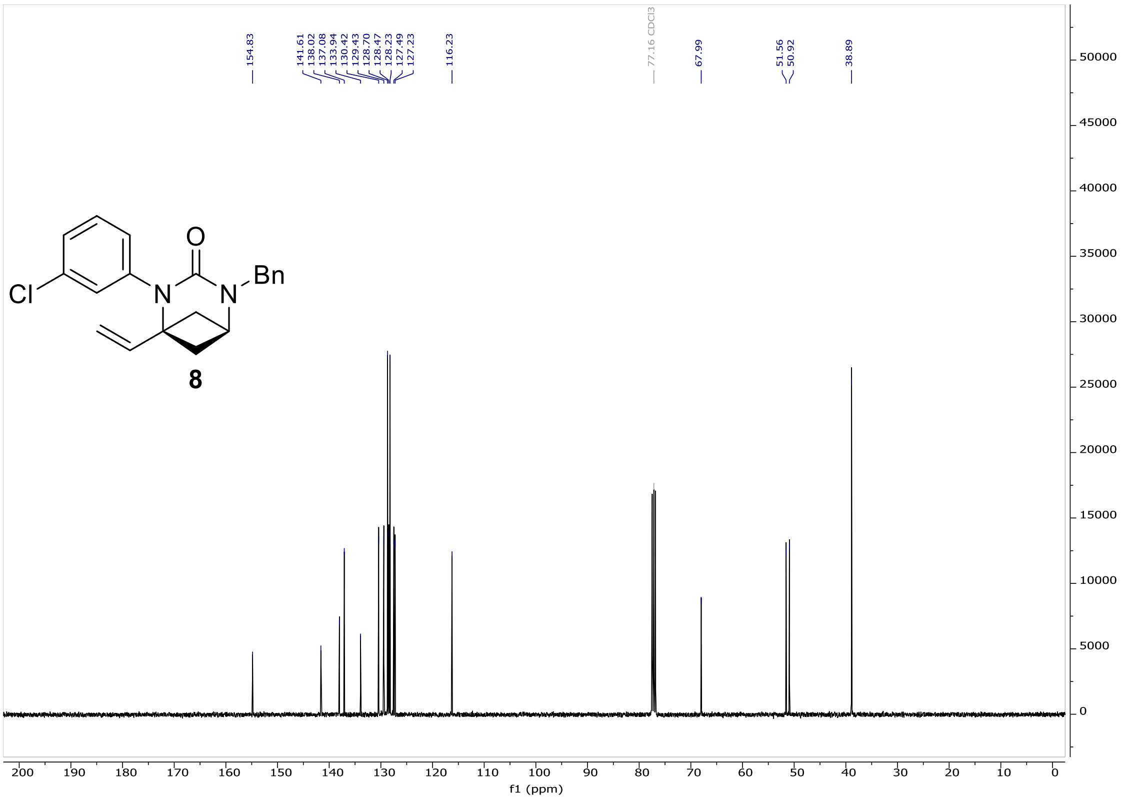

^{1}H NMR (**9a**, DMSO, 400 MHz)

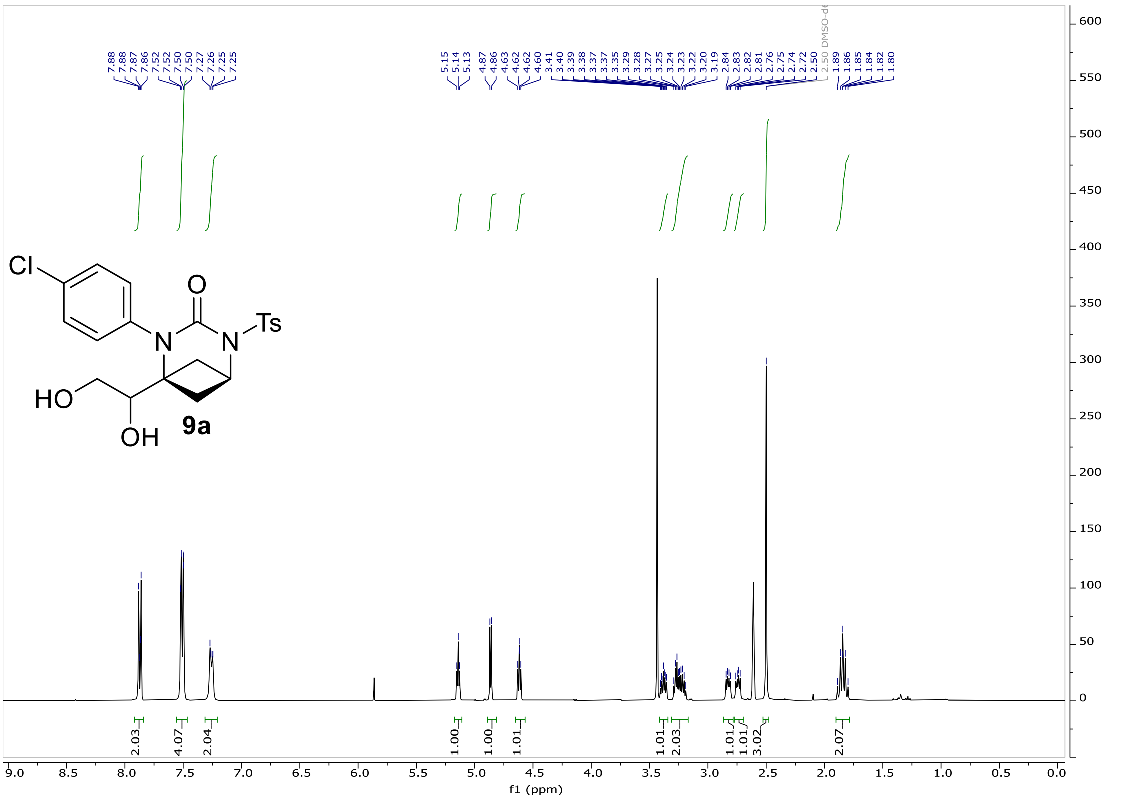

^{13}C NMR (**9a**, DMSO, 126 MHz)

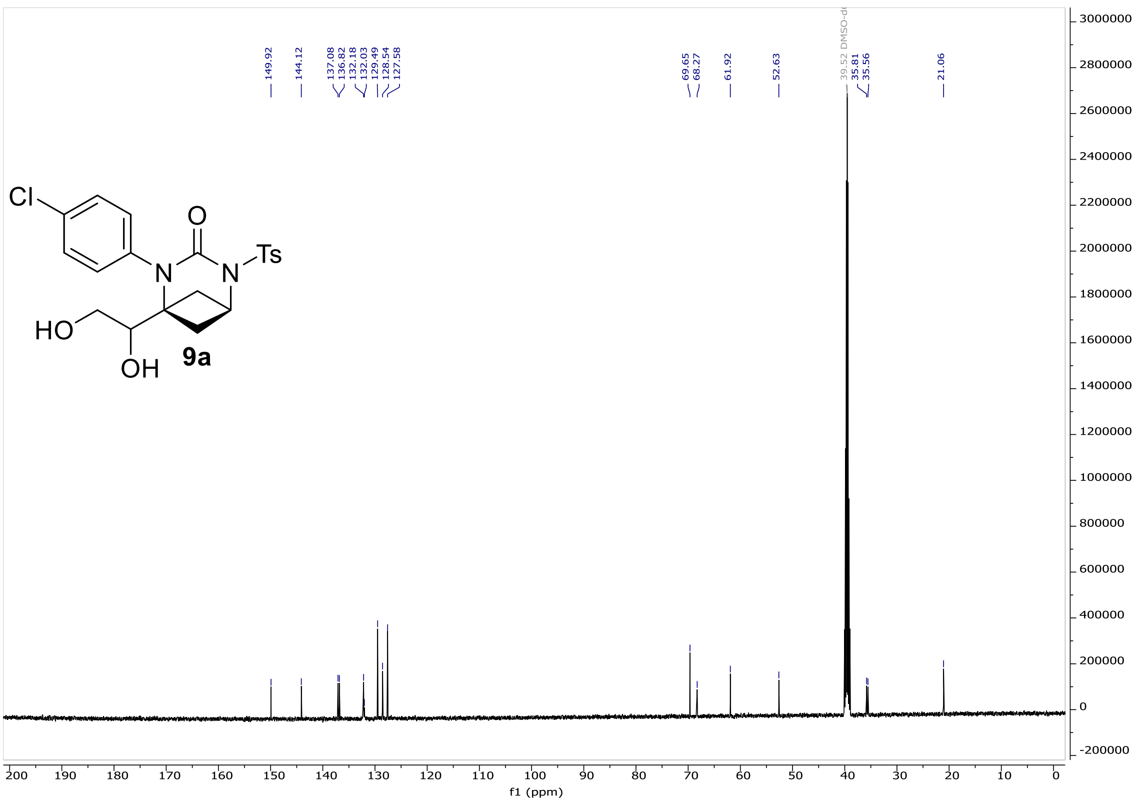

^{1}H NMR (**9b**, DMSO, 400 MHz)

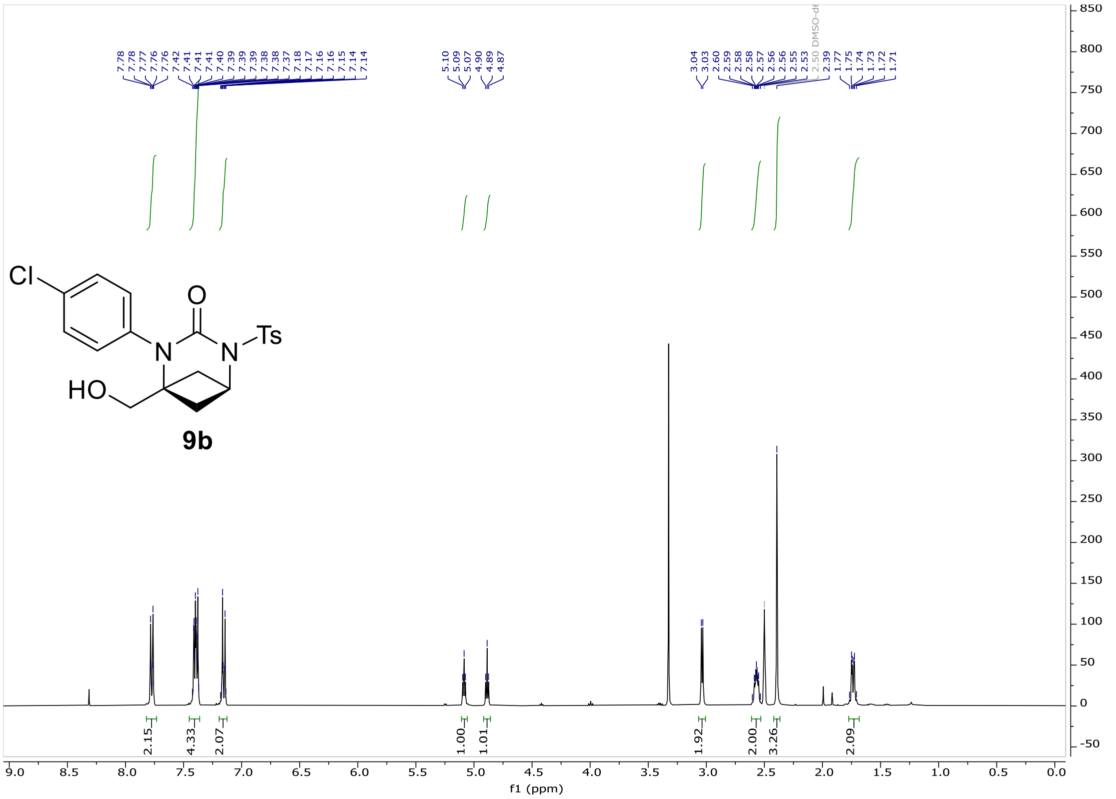

^{13}C NMR (**9b**, DMSO, 101 MHz)

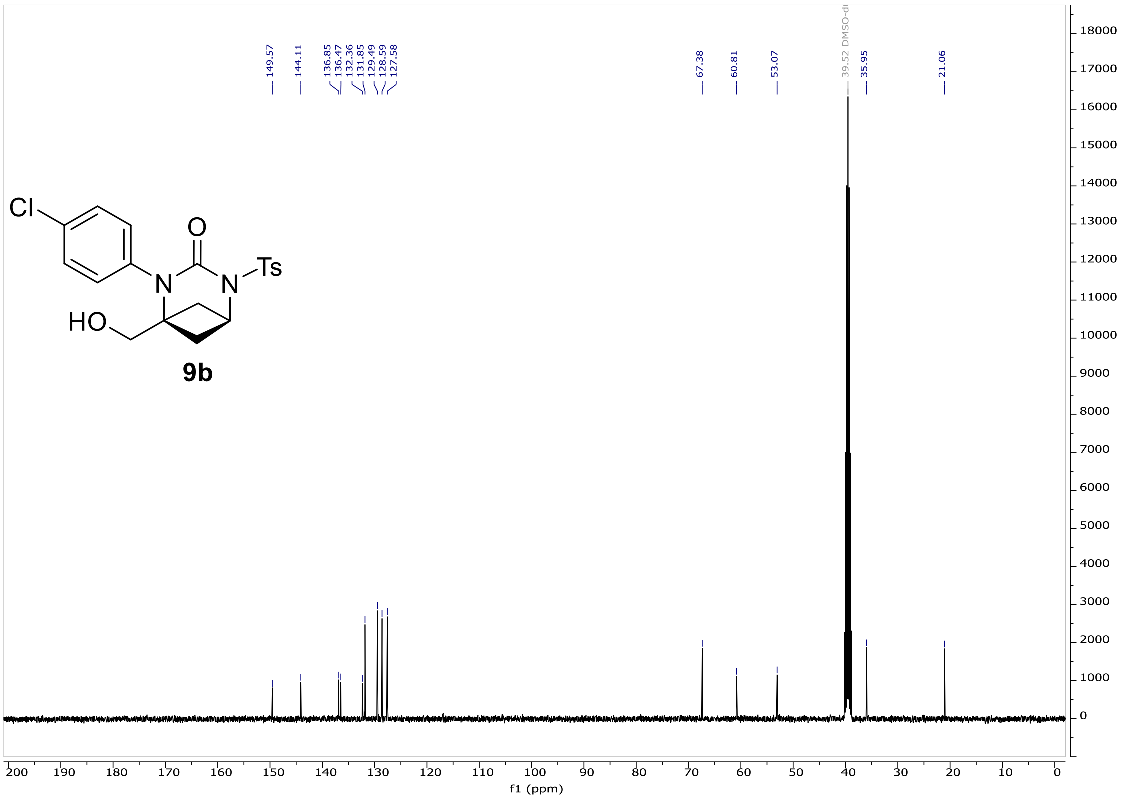

^{1}H NMR (**10**, DMSO, 500 MHz)

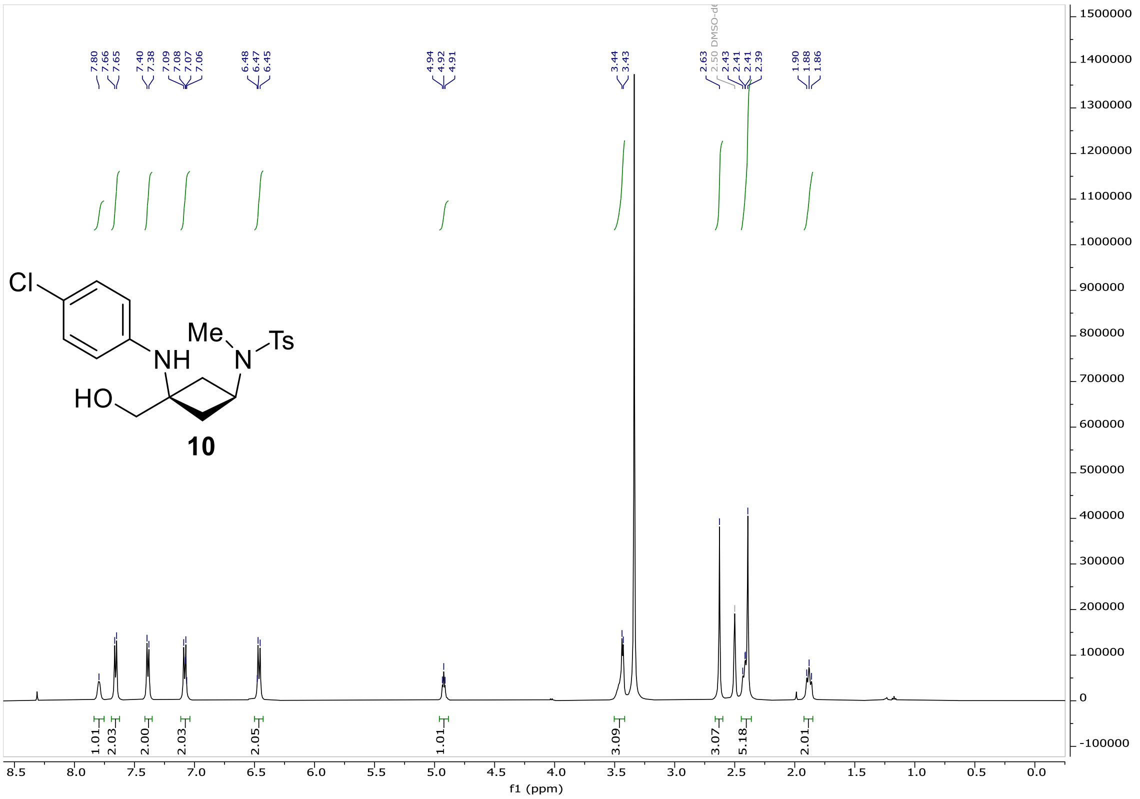

^{13}C NMR (**10**, DMSO, 126 MHz)

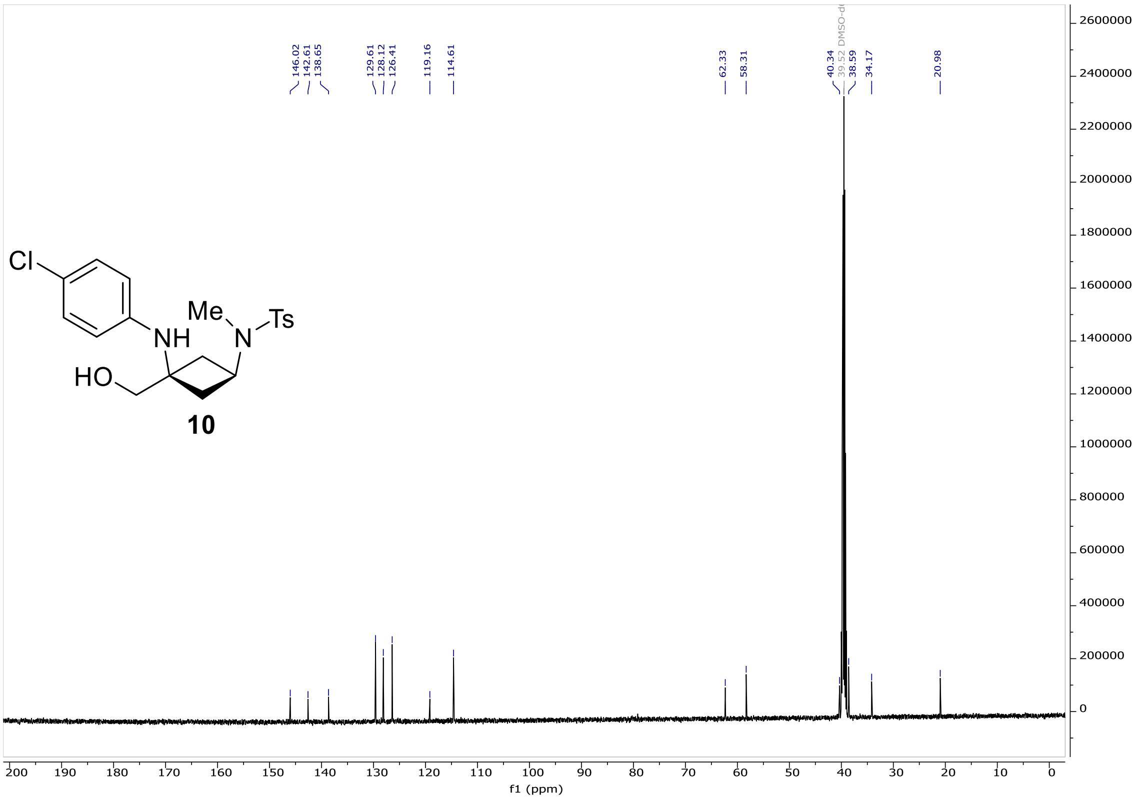

^{1}H NMR (**11**, $CDCl_3$, 300 MHz)

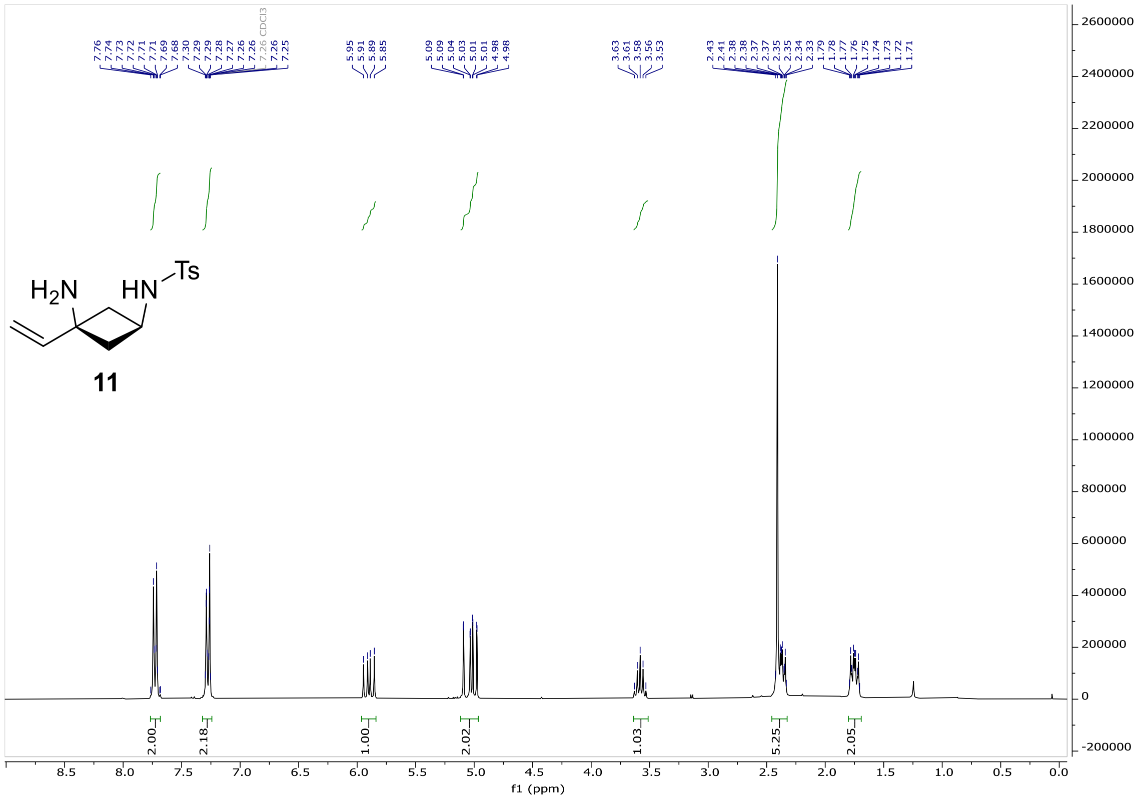

^{13}C NMR (**11**, $CDCl_3$, 101 MHz)

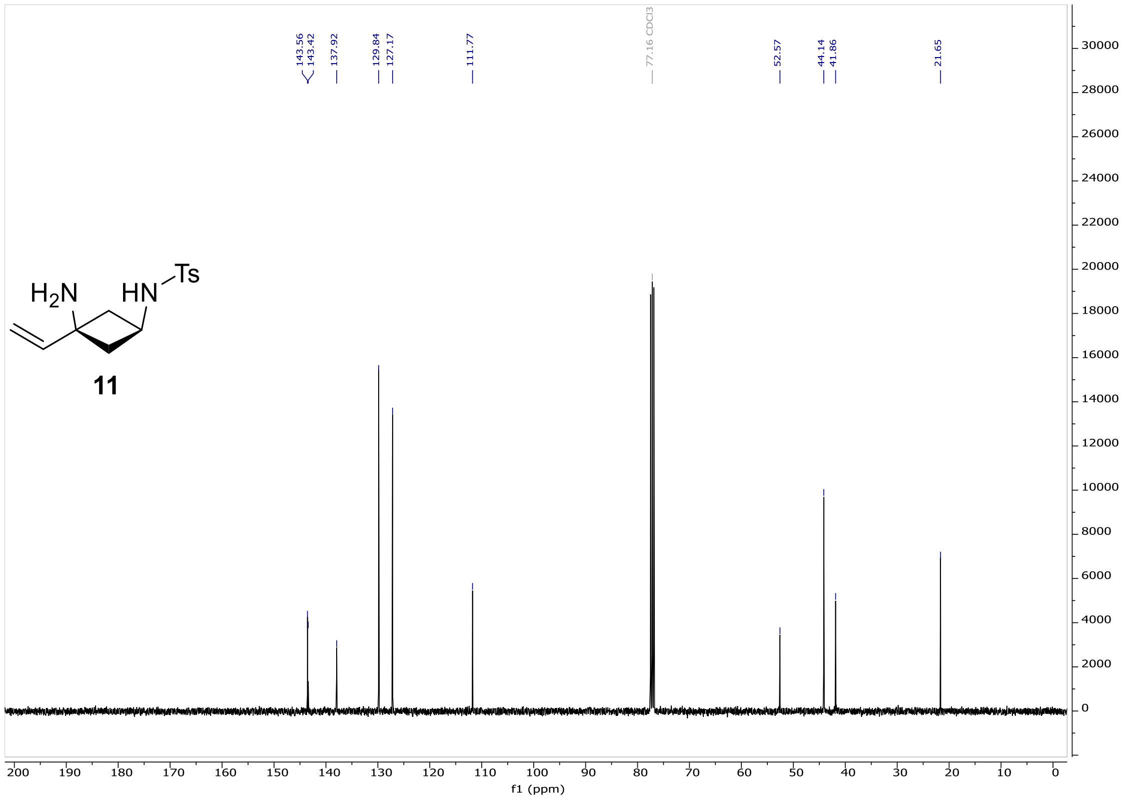

^{1}H NMR (**12**, $CDCl_3$, 300 MHz)

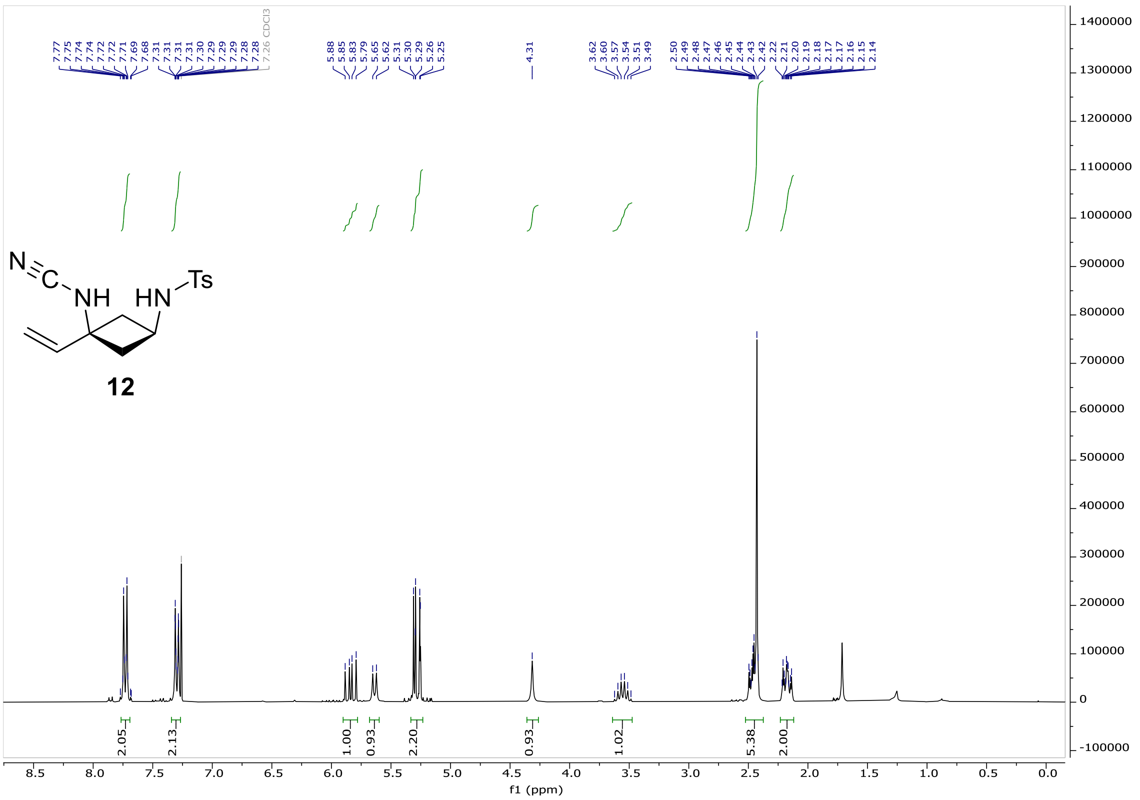

12

^{13}C NMR (**12**, $CDCl_3$, 126 MHz)

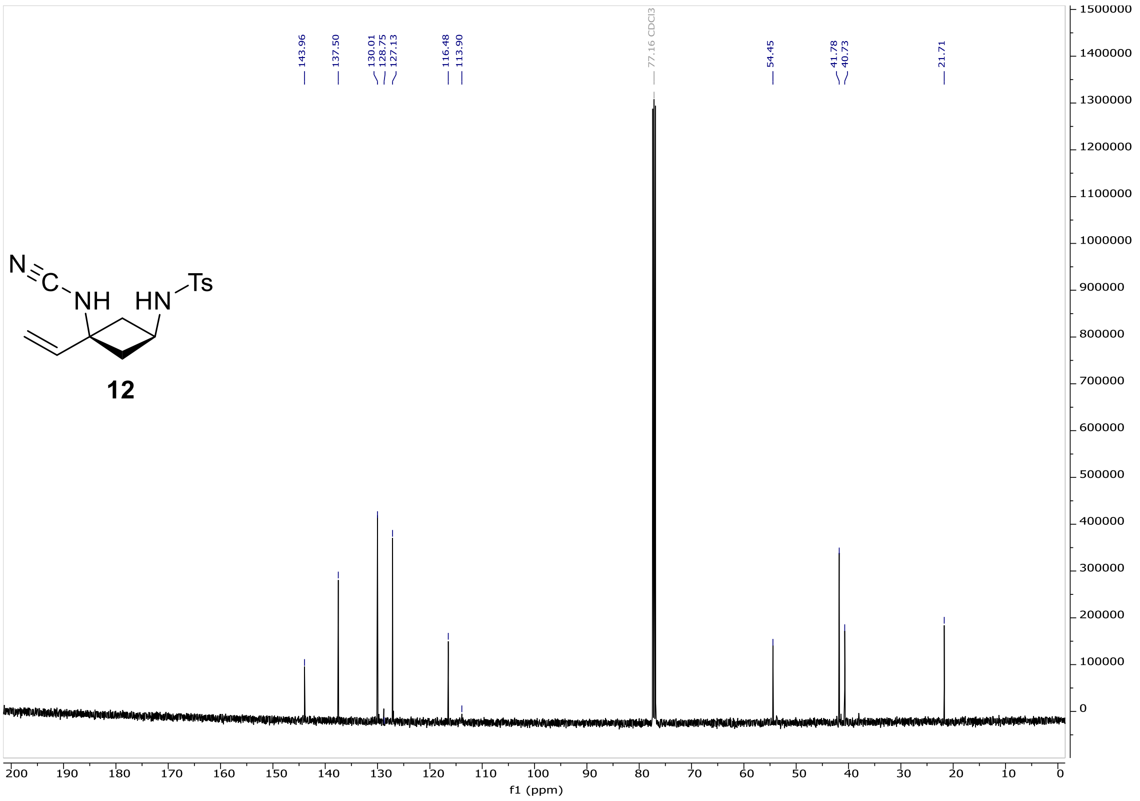

12

^{1}H NMR (**13**, $CDCl_3$, 500 MHz)

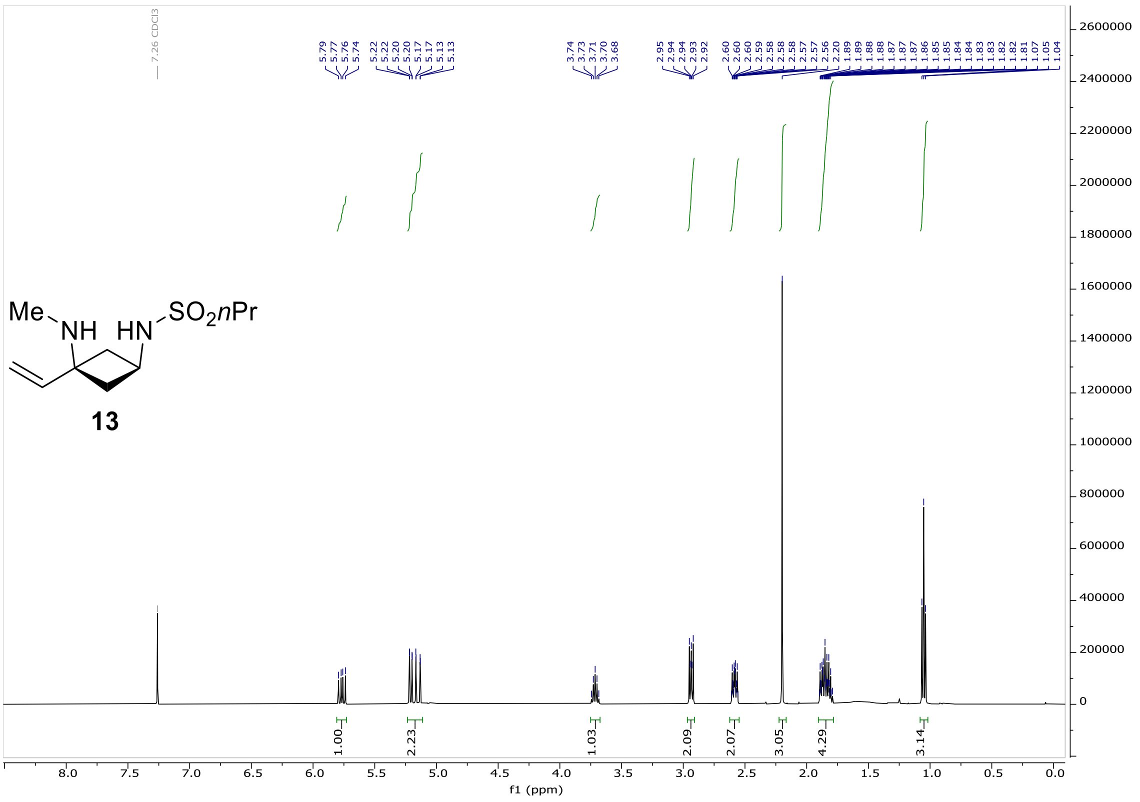

^{13}C NMR (**13**, $CDCl_3$, 126 MHz)

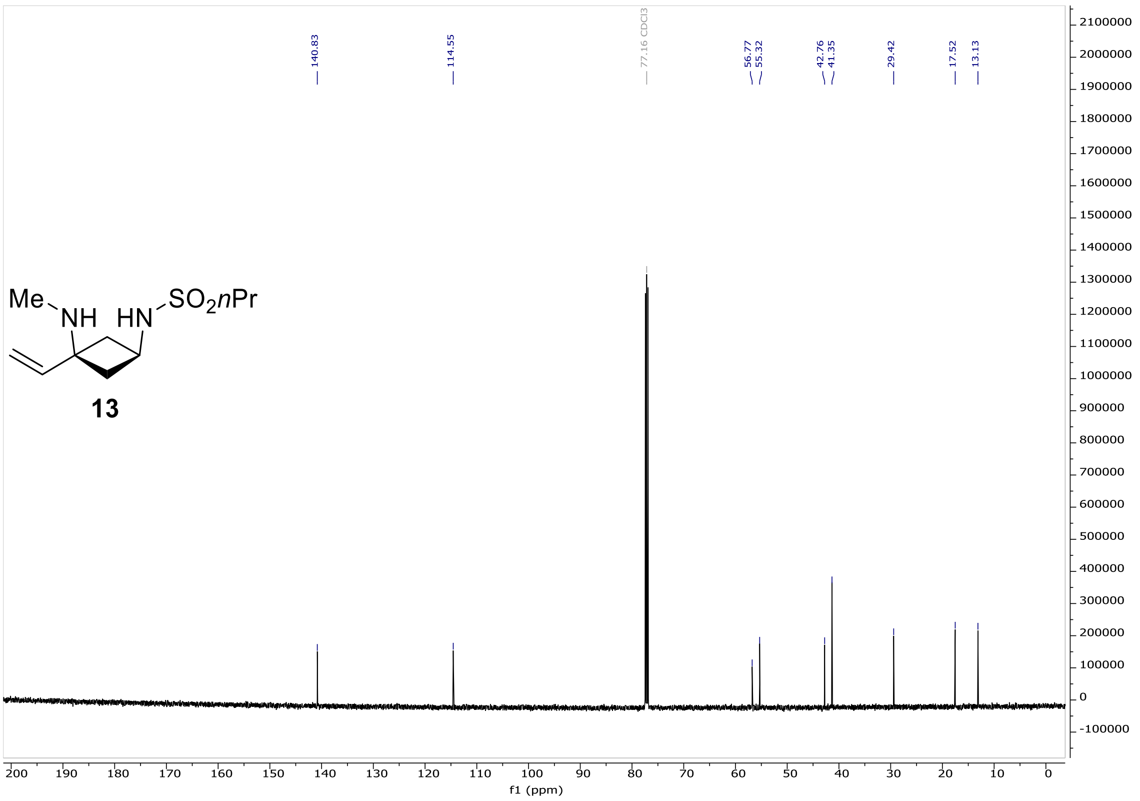

^{1}H NMR (**14**, DMSO, 300 MHz)

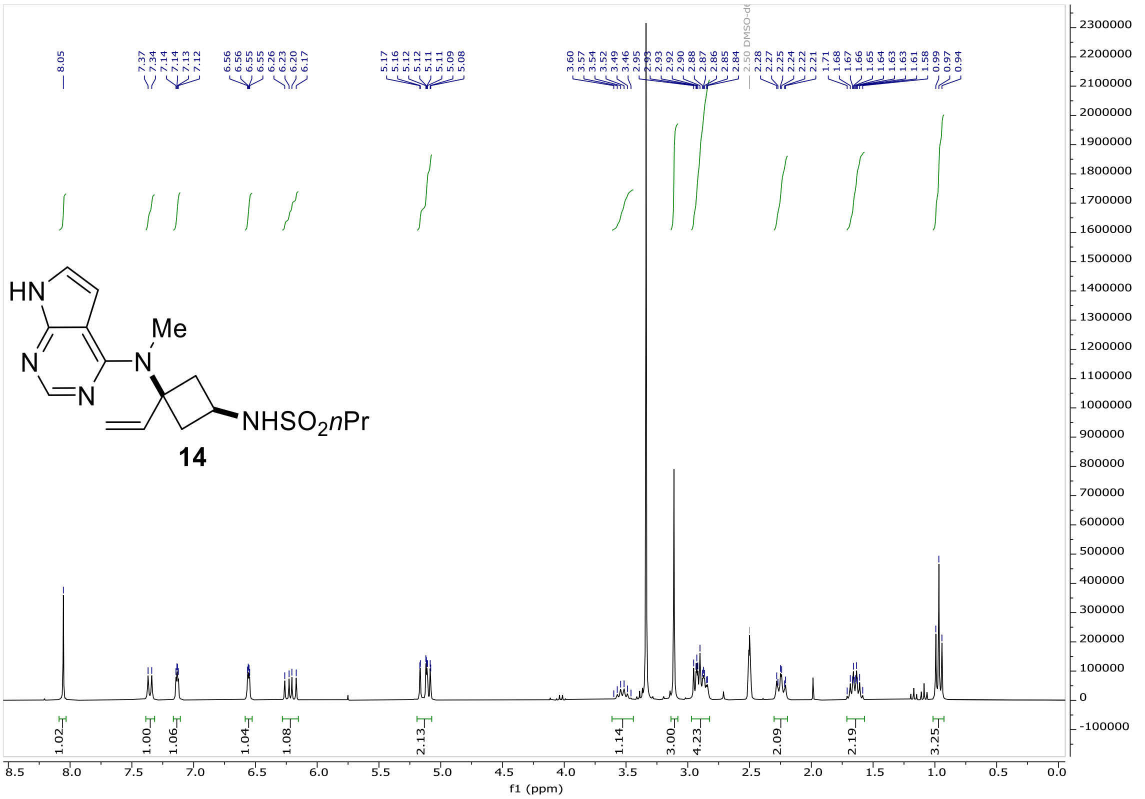

^{13}C NMR (**14**, DMSO, 126 MHz)

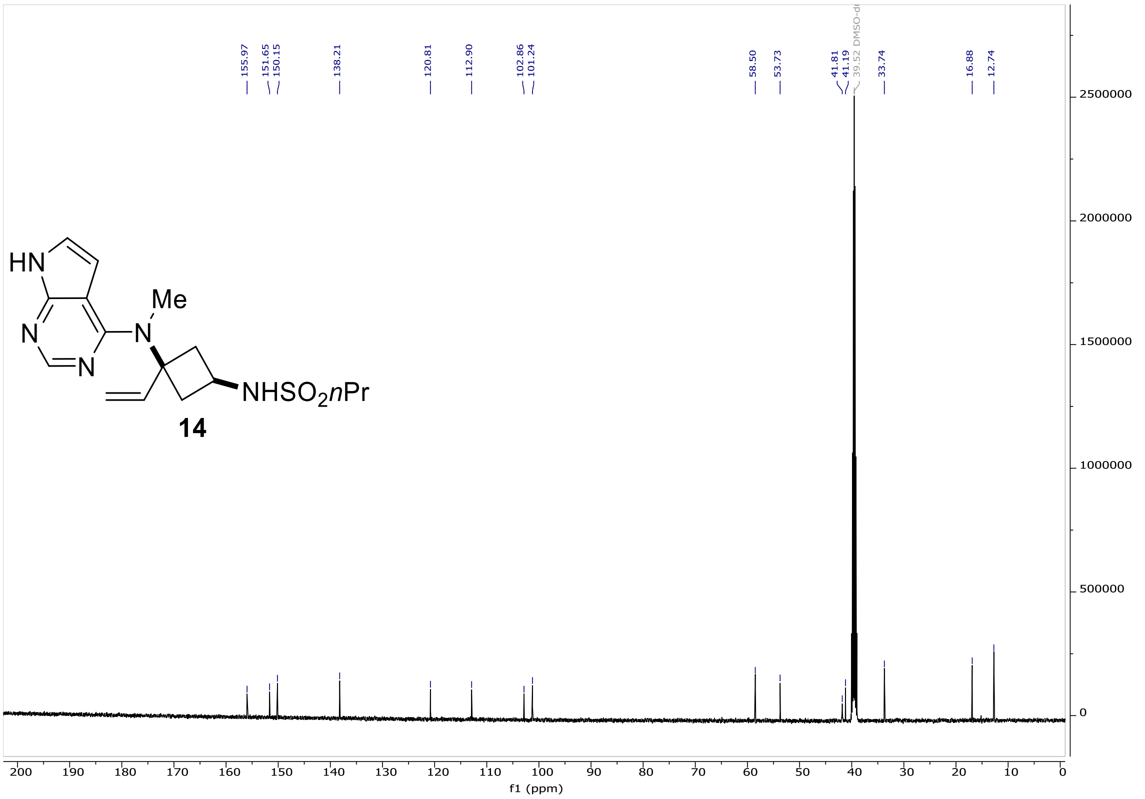

^{1}H NMR (**15**, $CDCl_3$, 400 MHz)

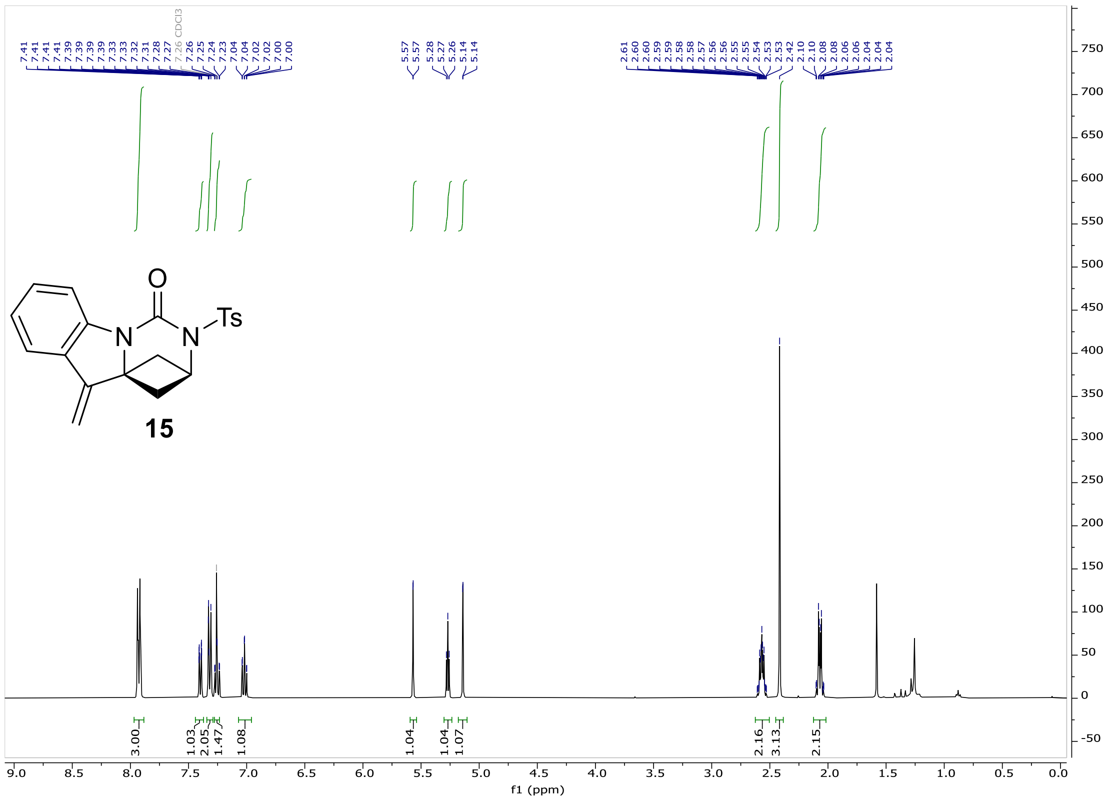

^{13}C NMR (**15**, $CDCl_3$, 101 MHz)

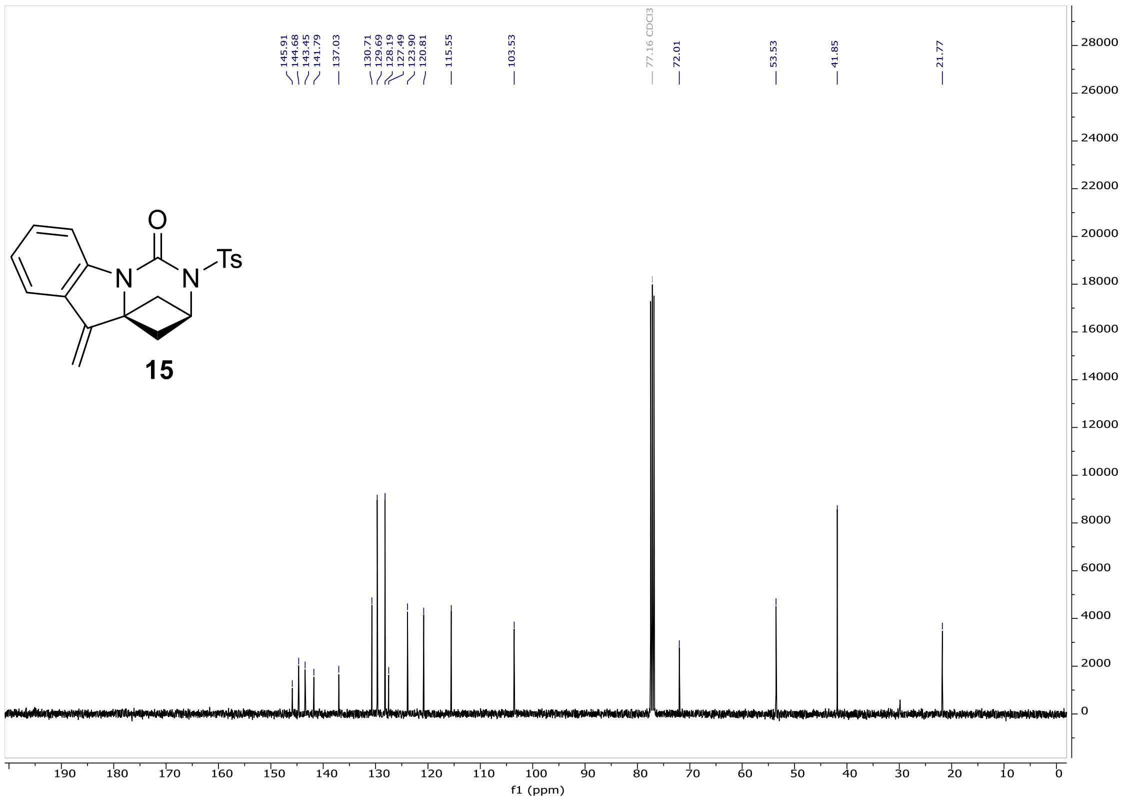

^{1}H NMR (**16**, $CDCl_3$, 400 MHz)

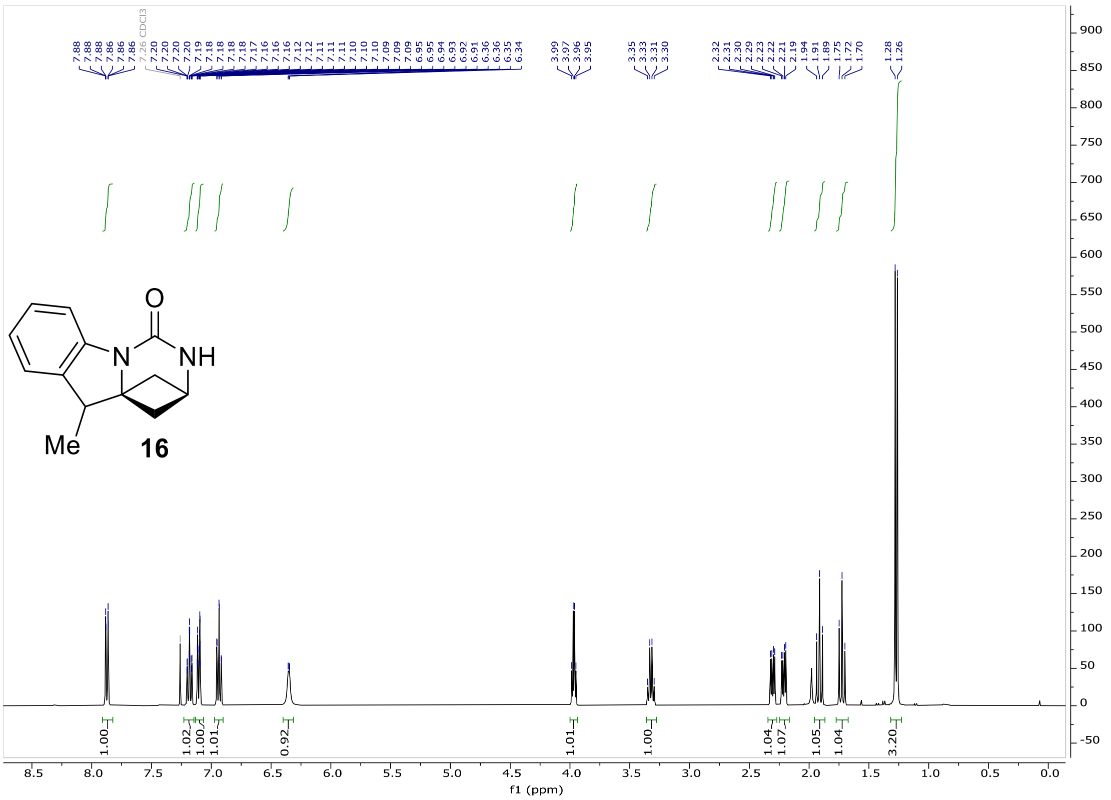

^{13}C NMR (**16**, $CDCl_3$, 101 MHz)

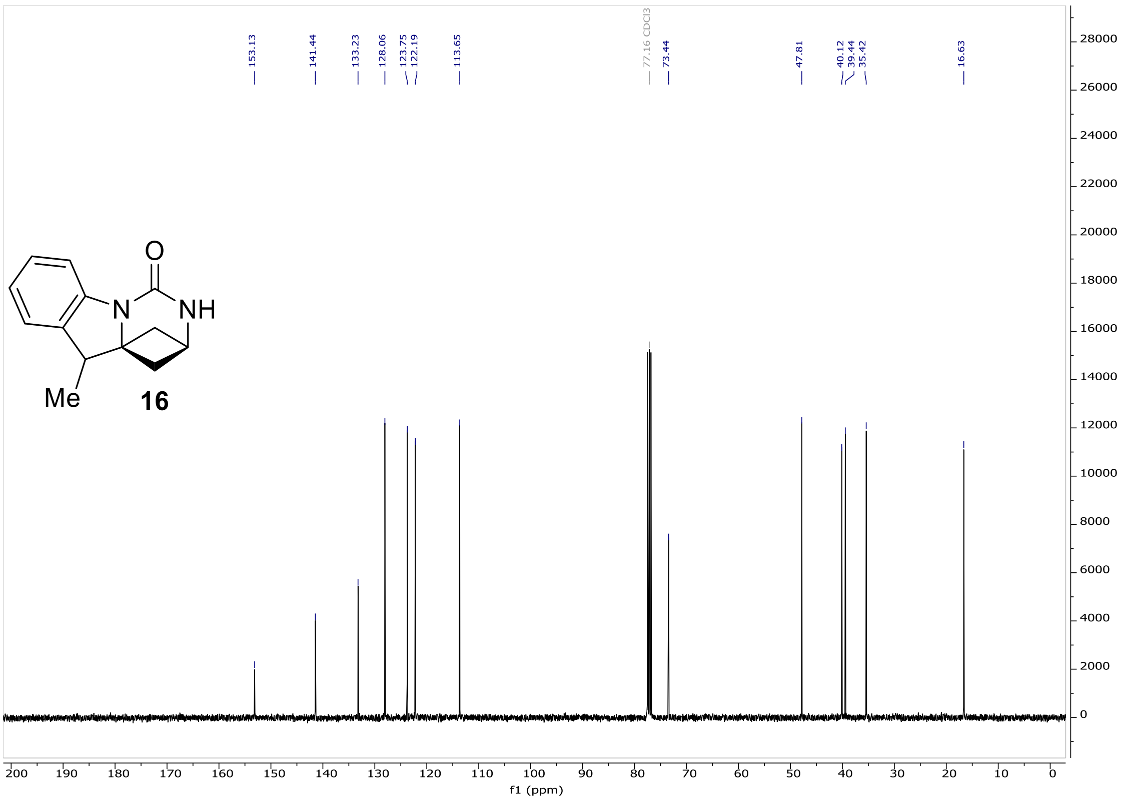

^{1}H NMR (**17**, $CDCl_3$, 300 MHz)

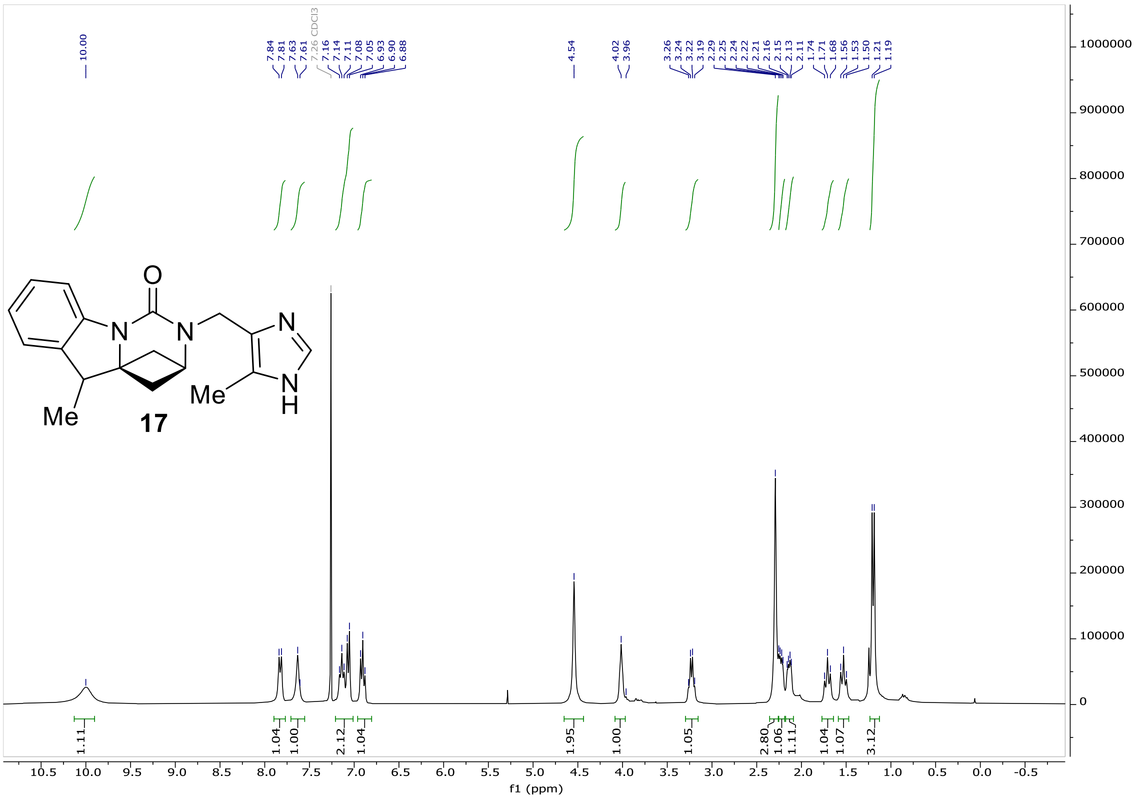

^{13}C NMR (**17**, $CDCl_3$, 126 MHz)

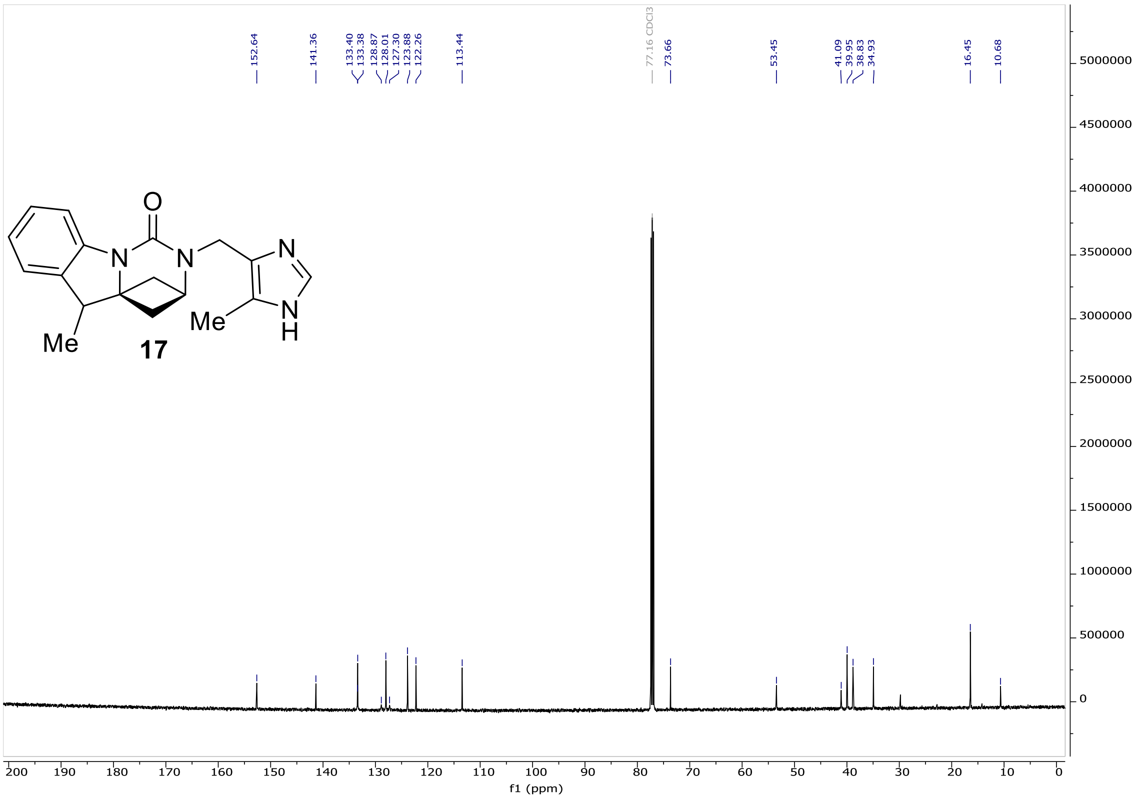

^{1}H NMR (**18**, $CDCl_3$, 400 MHz)

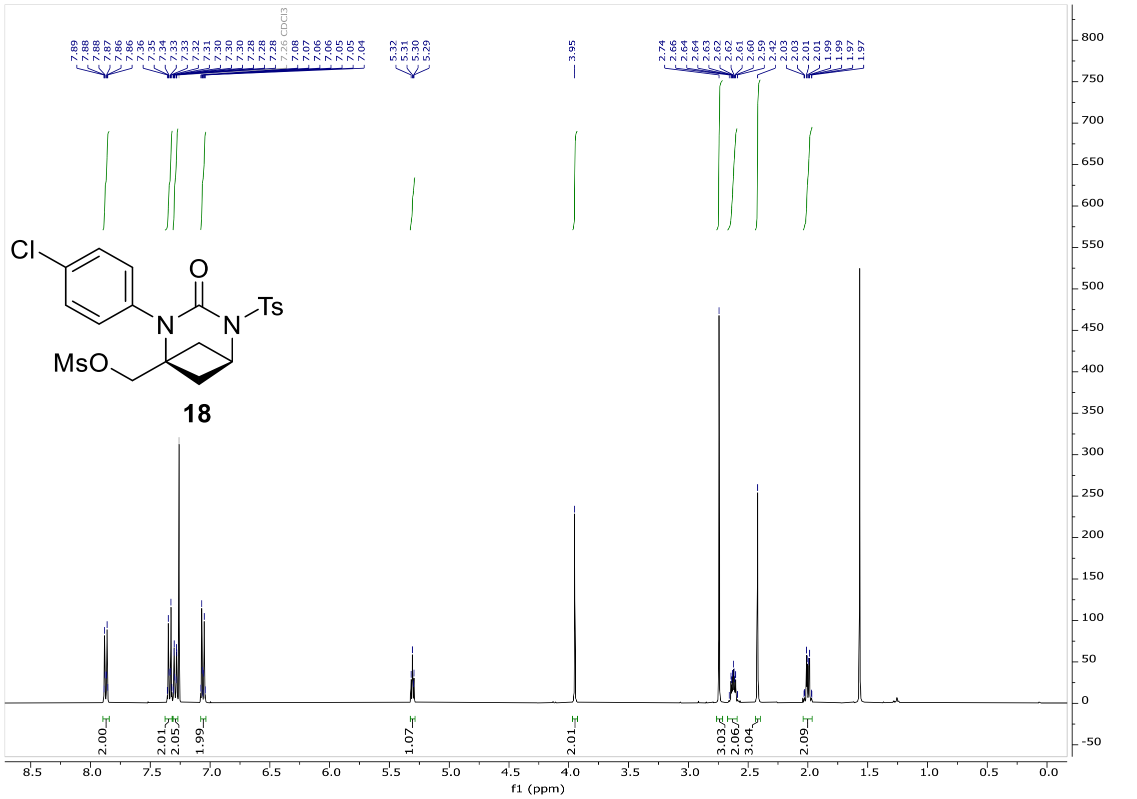

^{13}C NMR (**18**, $CDCl_3$, 101 MHz)

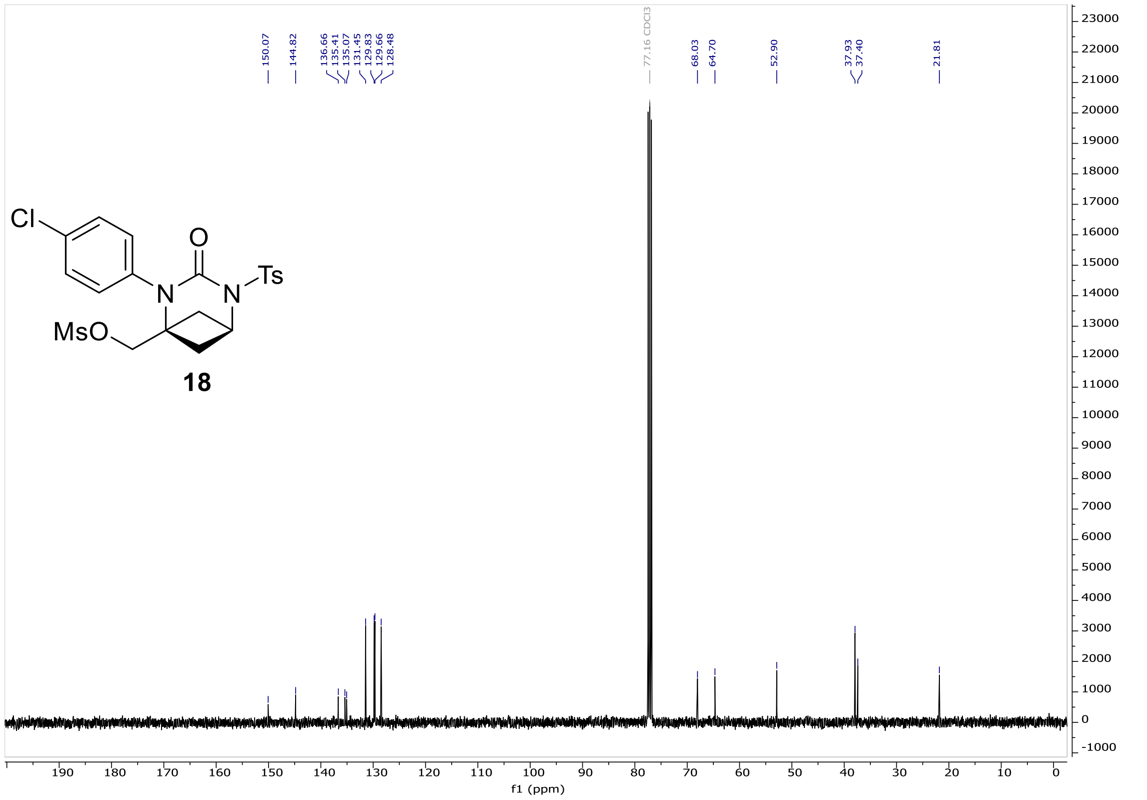

^{1}H NMR (**19**, $CDCl_3$, 500 MHz)

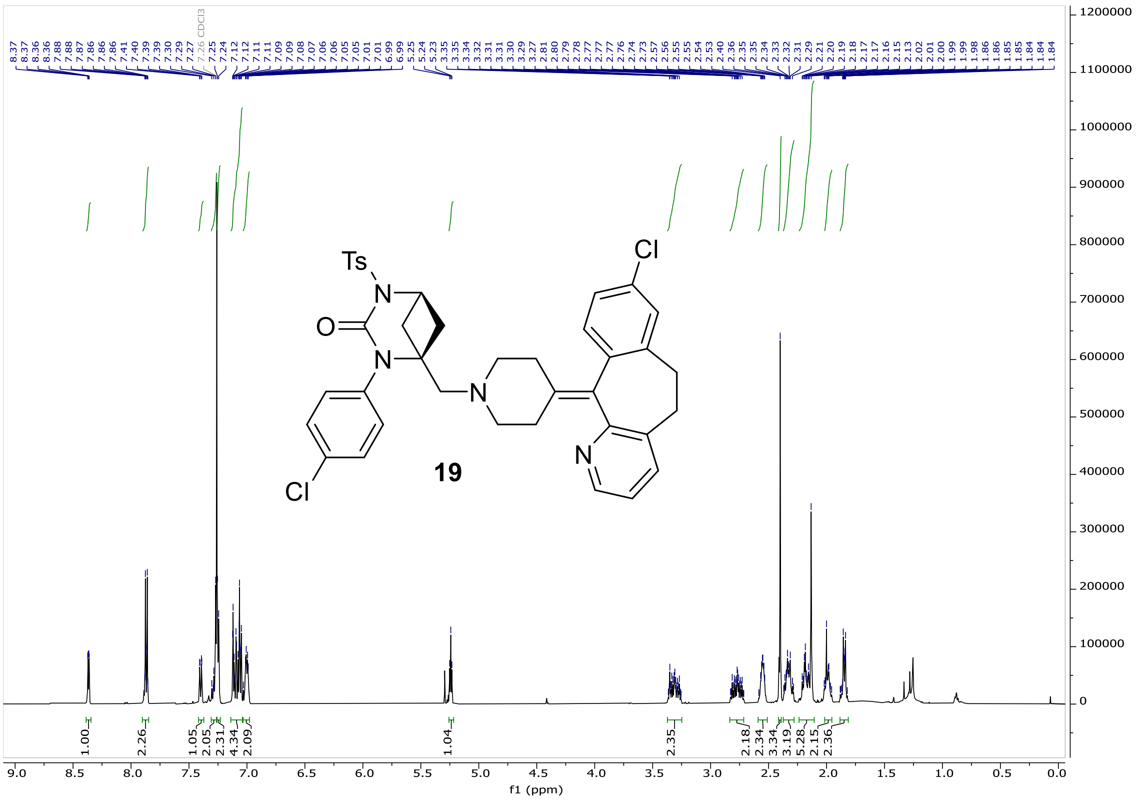

^{13}C NMR (**19**, $CDCl_3$, 126 MHz)

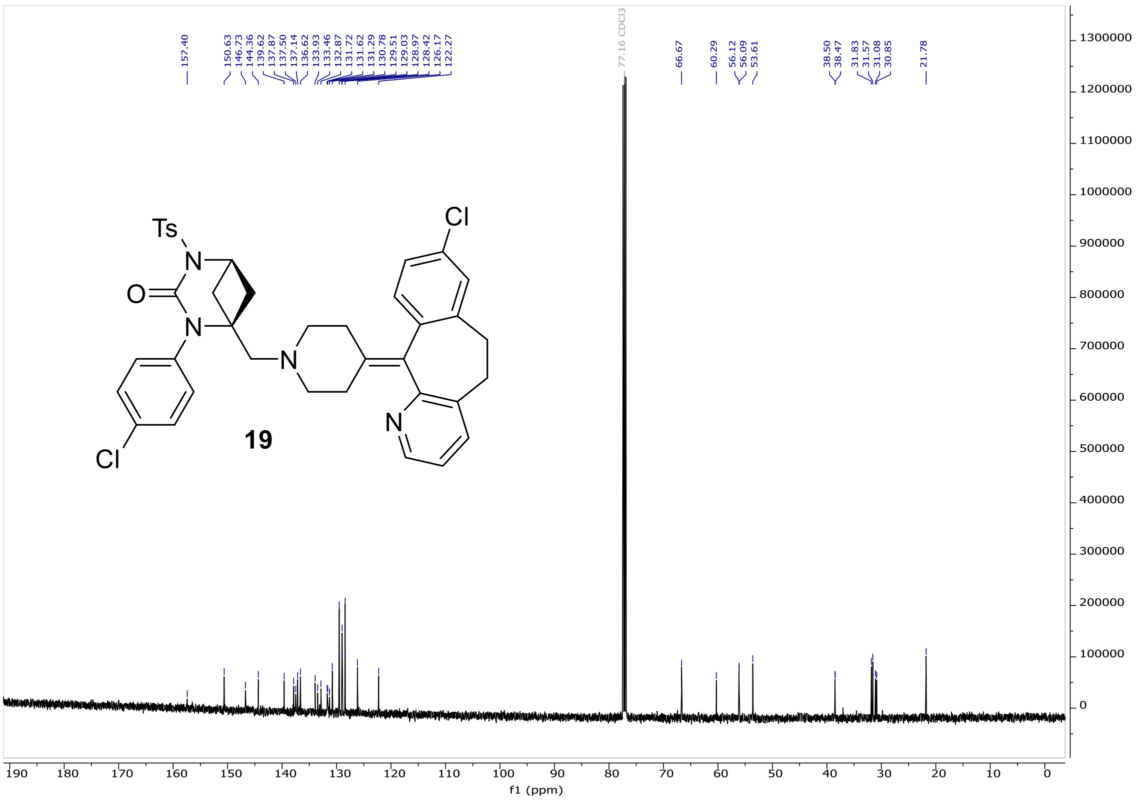

6. X-ray analysis and comments

Experimental. Single crystals of **3a** and **9a** were used as supplied. A suitable crystal was selected and mounted on a XtaLAB AFC11 (RCD3): quarter-chi single diffractometer. The crystal was kept at 100.00(10) K during data collection. Using Olex2,[5] the structure was solved with the SHELXT[6] structure solution program using Intrinsic Phasing and refined with the SHELXL[7] refinement package using Least Squares minimisation.

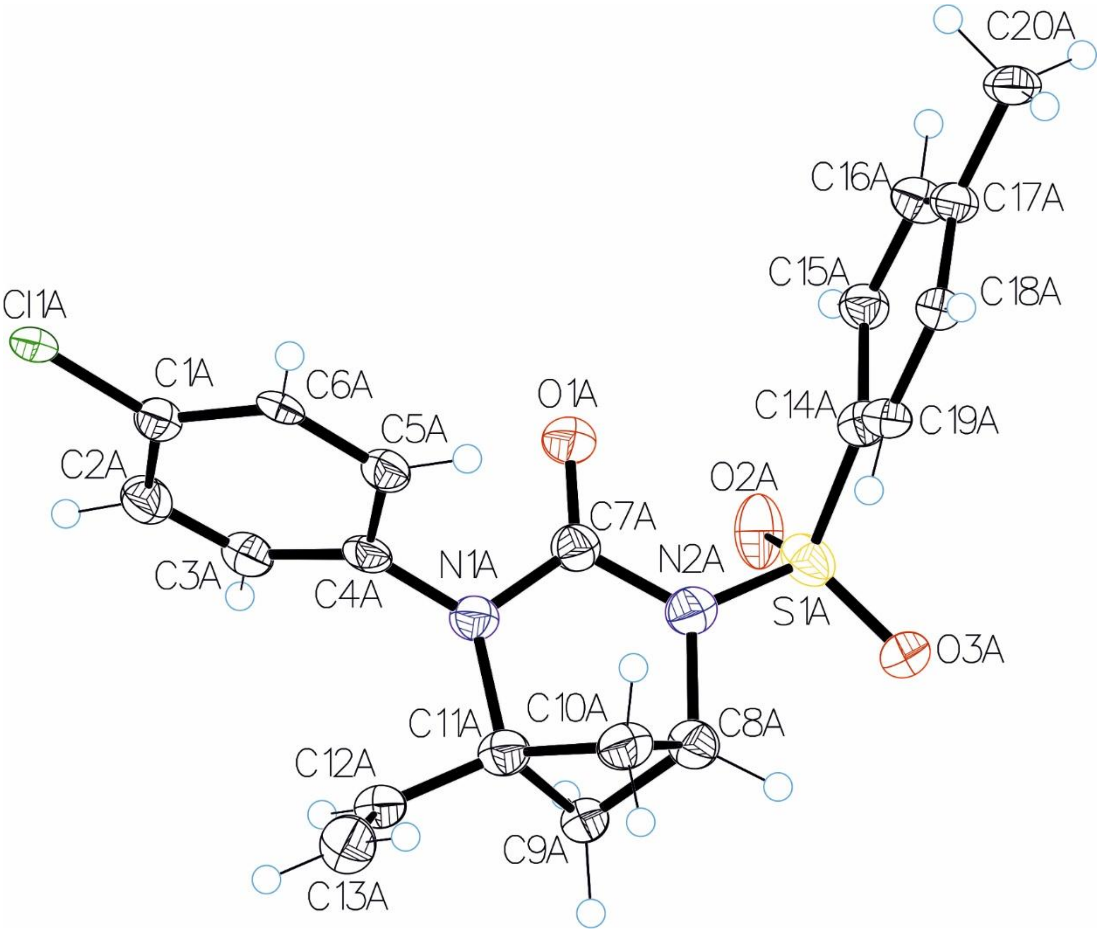

Displacement ellipsoid plot (50% probability level) of the molecular structure determined for compound **3a**. Details can be found in **CCDC-2488832**.

Sample preparation

Single crystals of **3a** were obtained by slow evaporation of a solution of **3a** in a hexane/ethyl acetate mixture (2:1) containing several drops of dichloromethane at room temperature, from which a suitable crystal was selected.

General comments:

Note: only weakly diffracting, low-quality multi-component crystals were available for this sample. Several attempts were made to collect a suitable dataset, and eventually one of them could be indexed and solved. Despite the poor crystal quality, the structure was deemed sufficient to confirm the ***identity*** and ***connectivity*** of the compound. The measured crystal turned out to be a racemic twin (TWIN LAW: -1.0, 0.0, 0.0, 0.0, -1.0, 0.0, 0.0, 0.0, -1.0; BASF [0.3(5)]). The asymmetric unit contains two independent molecules of the expected compound. The structure is of acceptable quality (no A-alerts and some commented B-alerts, see the CheckCif file for full details) with an R1 value of 15.96 %.

Commented B-alerts*:*

PROBLEM*: High R_1 Value, 0.16.*
RESPONSE*: Only weakly diffracting, low-quality multi-component crystals were available for this sample. Several attempts were made to collect a suitable dataset, and eventually one of them could be indexed and solved. Despite the poor crystal quality, the structure was deemed sufficient to confirm the identity and connectivity of the compound.*

PROBLEM*: High wR_2 Value (i.e. > 0.25) 0.41*
RESPONSE*: Only weakly diffracting, low-quality multi-component crystals were available for this sample. Several attempts were made to collect a suitable dataset, and eventually one of them could be indexed and solved. Despite the poor crystal quality, the structure was deemed sufficient to confirm the identity and connectivity of the compound.*

PROBLEM*: Low Bond Precision on C-C Bonds 0.035 Ang*
RESPONSE*: Only weakly diffracting, low-quality multi-component crystals were available for this sample. Several attempts were made to collect a suitable dataset, and eventually one of them could be indexed and solved. Despite the poor crystal quality, the structure was deemed sufficient to confirm the identity and connectivity of the compound.*

Table S2. Crystal data and structure refinement for **3a**

Empirical formula	$C_{40}H_{38}Cl_2N_4O_6S_2$	a/Å	14.176(2)
Formula weight	805.76	b/Å	5.8581(9)
Temperature/K	100.00(10)	c/Å	45.014(8)
Crystal system	orthorhombic	α/°	90
Space group	$Pna2_1$	β/°	90

γ/°	90
Volume/Å^3	3738.0(11)
Z	4
ρ_{calc} / g·cm^{-3}	1.432
μ/mm^{-1}	0.340
F(000)	1680.0
Crystal size/mm^3	0.3 × 0.2 × 0.05
Radiation	Mo Kα (λ = 0.71073)
2Θ range for data collection/°	5.748 to 50.456
Index ranges	-10 ≤ h ≤ 16, -5 ≤ k ≤ 7, -46 ≤ l ≤ 53
Reflections collected	19646
Independent reflections	6170 [R_{int} = 0.1138, R_{sigma} = 0.1457]
Data/restraints/parameters	6170/753/490
Goodness-of-fit on F^2	1.106
Final R indexes [I>=2σ (I)]	R_1 = 0.1596, wR_2 = 0.3871
Final R indexes [all data]	R_1 = 0.1881, wR_2 = 0.4058
Largest diff. peak/hole / e Å^{-3}	1.50/-1.18

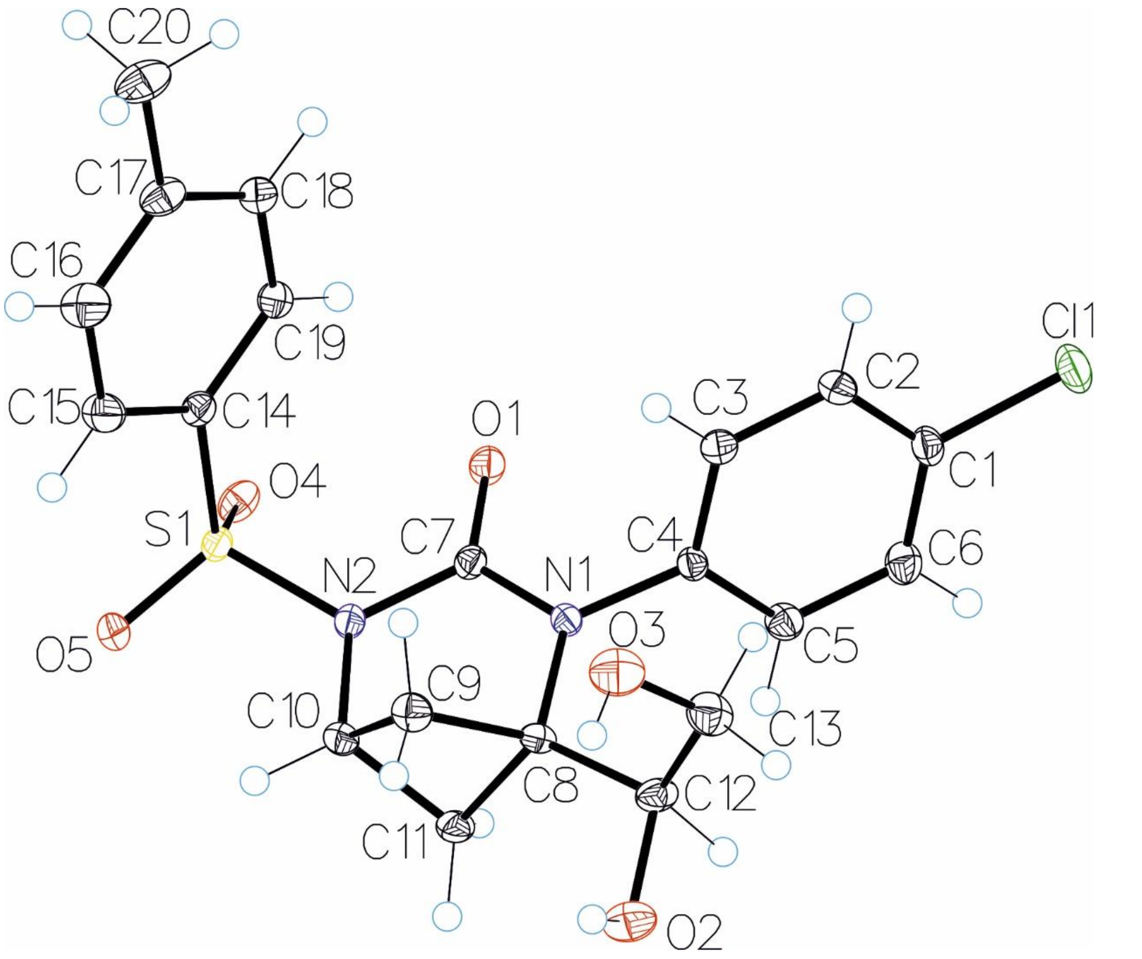

Displacement ellipsoid plot (50% probability level) of the molecular structure determined for compound **9a**. Details can be found in **CCDC-2486203**.

Sample preparation

Single crystals of **9a** were obtained by slow evaporation of a solution of **9a** in a hexane/ethyl acetate mixture (1:1) containing several drops of dichloromethane at room temperature, from which a suitable crystal was selected.

Table S3. Crystal data and structure refinement for **9a**

Empirical formula	$C_{20}H_{21}ClN_2O_5S$	a/Å	18.5596(4)
Formula weight	436.90	b/Å	7.37030(10)
Temperature/K	100.00(10)	c/Å	14.2784(3)
Crystal system	monoclinic	α/°	90
Space group	*P*2$_1$/c	β/°	100.917(2)

γ/°	90
Volume/Å^3	1917.79(6)
Z	4
ρ_{calc} / g·cm^{-3}	1.513
μ/mm^{-1}	0.345
F(000)	912.0
Crystal size/mm^3	0.3 × 0.3 × 0.2
Radiation	Mo Kα (λ = 0.71073)
2Θ range for data collection/°	4.47 to 61.804
Index ranges	-26 ≤ h ≤ 18, -10 ≤ k ≤ 10, -19 ≤ l ≤ 20
Reflections collected	16804
Independent reflections	5618 [R_{int} = 0.0275, R_{sigma} = 0.0273]
Data/restraints/parameters	5618/0/265
Goodness-of-fit on F^2	1.037
Final R indexes [I>=2σ (I)]	R_1 = 0.0410, wR_2 = 0.1162
Final R indexes [all data]	R_1 = 0.0464, wR_2 = 0.1195
Largest diff. peak/hole / e Å^{-3}	1.23/-0.57

7. References

(1) Zhou, J.-L.; Xiao, Y.; He, L.; Gao, X.-Y.; Yang, X.-C.; Wu, W.-B.; Wang, G.; Zhang, J.; Feng, J.-J. Palladium-Catalyzed Ligand-Controlled Switchable Hetero-(5+3)/Enantioselective [2σ+2σ] Cycloadditions of Bicyclobutanes with Vinyl Oxiranes. *J. Am. Chem. Soc*. **2024**, *146*, 19621–19628.

(2) Zhang, Z.; Sun, Y.; Gong, Y.; Tang, D.-L.; Luo, H.; Zhao, Z.-P.; Zhou, F.; Wang, X.; Zhou, J. Enantioselective propargylic amination and related tandem sequences to α-tertiary ethynylamines and azacycles. *Nat. Chem.* **2024**, *16*, 521–532.

(3) Connor, C. G.; DeForest, J. C.; Dietrich, P.; Do, N. M.; Doyle, K. M.; Eisenbeis, S.; Greenberg, E.; Griffin, S. H.; Jones, B. P.; Jones, K. N.; Karmilowicz, M.; Kumar, R.; Lewis, C. A.; McInturff, E. L.; McWilliams, J. C.; Mehta, R.; Nguyen, B. D.; Rane, A. M.; Samas, B.; Sitter, B. J.; Ward, H. W.; Webste, M. E. Development of a Nitrene-Type Rearrangement for the Commercial Route of the JAK1 Inhibitor Abrocitinib. *Org. Process Res. Dev.* **2021**, *25*, 608–615.

(4) Kato, M.; Nishino, S.; Ito, K.; Yamakuni, H.; Takasugi, H. New 5-HT3(Serotonin-3) Receptor Antagonists. II. Synthesis and Structure-Activity Relationships of Pyrimido[1,6-α]indoles. *Chem. Pharm. Bull*. **1994**, *42*, 2556-2564.

(5) Dolomanov, O. V.; Bourhis, L. J.; Gildea, R. J.; Howard, J. A. K.; Puschmann, H. OLEX2: a complete structure solution, refinement and analysis program. *J. Appl. Cryst*. **2009**, *42*, 339-341.

(6) Sheldrick, G. M. SHELXT – Integrated space-group and crystal structure determination. *Acta Cryst*. **2015**, *A71*, 3-8.

(7) Sheldrick, G. M. Crystal structure refinement with SHELXL. *Acta Cryst*. **2015**, *C71*, 3-8.
